# Quantitative proteomics analysis of permethrin and temephos-resistant *Ae*. *aegypti* revealed diverse differentially expressed proteins associated with insecticide resistance from Penang Island, Malaysia

**DOI:** 10.1371/journal.pntd.0011604

**Published:** 2023-09-18

**Authors:** Abubakar Shettima, Intan Haslina Ishak, Benjamin Lau, Hadura Abu Hasan, Noorizan Miswan, Nurulhasanah Othman

**Affiliations:** 1 Institute for Research in Molecular Medicine (INFORMM), Universiti Sains Malaysia, Gelugor, Malaysia; 2 Department of Microbiology, University of Maiduguri, Maiduguri, Nigeria; 3 School of Biological Sciences (SBS), Universiti Sains Malaysia, Gelugor, Malaysia; 4 Vector Control Research Unit (VCRU), Universiti Sains Malaysia, Gelugor, Malaysia; 5 Proteomics and Metabolomics (PROMET) laboratory, Malaysian Palm Oil Board (MPOB), Kajang, Malaysia; 6 Center for Chemical Biology (CCB), Universiti Sains Malaysia, Bayan Lepas, Malaysia; Kenya Agricultural and Livestock Research Organization, KENYA

## Abstract

Synthetic insecticides are the primary vector control method used globally. However, the widespread use of insecticides is a major cause of insecticide-resistance in mosquitoes. Hence, this study aimed at elucidating permethrin and temephos-resistant protein expression profiles in *Ae*. *aegypti* using quantitative proteomics. In this study, we evaluated the susceptibility of *Ae*. *aegypti* from Penang Island dengue hotspot and non-hotspot against 0.75% permethrin and 31.25 mg/l temephos using WHO bioassay method. Protein extracts from the mosquitoes were then analysed using LC–ESI–MS/MS for protein identification and quantification via label-free quantitative proteomics (LFQ). Next, Perseus 1.6.14.0 statistical software was used to perform differential protein expression analysis using ANOVA and Student’s t-test. The t-test selected proteins with≥2.0-fold change (FC) and ≥2 unique peptides for gene expression validation via qPCR. Finally, STRING software was used for functional ontology enrichment and protein-protein interactions (PPI). The WHO bioassay showed resistance with 28% and 53% mortalities in adult mosquitoes exposed to permethrin from the hotspot and non-hotspot areas. Meanwhile, the susceptibility of *Ae*. *aegypti* larvae revealed high resistance to temephos in hotspot and non-hotspot regions with 80% and 91% mortalities. The LFQ analyses revealed 501 and 557 (q-value <0.05) differentially expressed proteins in adults and larvae *Ae*. *aegypti*. The t-test showed 114 upregulated and 74 downregulated proteins in adult resistant versus laboratory strains exposed to permethrin. Meanwhile, 13 upregulated and 105 downregulated proteins were observed in larvae resistant versus laboratory strains exposed to temephos. The t-test revealed the upregulation of sodium/potassium-dependent ATPase β2 in adult permethrin resistant strain, H15 domain-containing protein, 60S ribosomal protein, and PB protein in larvae temephos resistant strain. The downregulation of troponin I, enolase phosphatase E1, glucosidase 2β was observed in adult permethrin resistant strain and tubulin β chain in larvae temephos resistant strain. Furthermore, the gene expression by qPCR revealed similar gene expression patterns in the above eight differentially expressed proteins. The PPI of differentially expressed proteins showed a p-value at <1.0 x 10^−16^ in permethrin and temephos resistant *Ae*. *aegypti*. Significantly enriched pathways in differentially expressed proteins revealed metabolic pathways, oxidative phosphorylation, carbon metabolism, biosynthesis of amino acids, glycolysis, and citrate cycle. In conclusion, this study has shown differentially expressed proteins and highlighted upregulated and downregulated proteins associated with insecticide resistance in *Ae*. *aegypti*. The validated differentially expressed proteins merit further investigation as a potential protein marker to monitor and predict insecticide resistance in field *Ae*. *aegypti*. The LC-MS/MS data were submitted into the MASSIVE database with identifier no: MSV000089259.

## Author summary

The study aimed to understand the protein expression profiles in *Ae*. *aegypti* mosquitoes resistant to permethrin and temephos insecticides using quantitative proteomics. The results revealed permethrin and temephos resistance in *Ae*. *aegypti* dengue hotspot and non-hotspot areas in Penang Island, Malaysia. The LC-MS/MS LFQ identified 501 and 577 differentially expressed proteins in adult and larvae *Ae*. *aegypti*. Furthermore, significantly enriched pathways of the differentially expressed proteins were involved in metabolic pathways, oxidative phosphorylation, carbon metabolism, biosynthesis of amino acids, glycolysis, and citrate cycle. A few differentially expressed proteins were validated as potential candidates for insecticide-resistant screening, prediction and monitoring in field strain *Ae*. *aegypti* mosquitoes in Malaysia.

## 1. Introduction

Insecticide resistance in the *Ae*. *aegypti* contributes to the dengue burden in Malaysia and other tropical countries. Apart from dengue, *Ae*. *aegypti* is also a vector for transmitting chikungunya, yellow fever, and Zika viral diseases. Chemical-based control is the primary control method used for dengue vectors in Malaysia and other countries. Some insecticides, including permethrin, deltamethrin, and malathion, are used against adult mosquitoes. Temephos is used to kill mosquitoes during their larval stage. Nonetheless, the widespread use of synthetic insecticides in chemical-based control methods deployed to fight vector-borne diseases has triggered insecticide resistance, particularly in mosquitoes [1]. *Ae*. *aegypti* has developed resistance to insecticides used for mosquito control in tropical and subtropical regions worldwide [2]. In many parts of Malaysia, insecticide resistance has been reported in the Aedes mosquitoes [3–7]. Ishak et al. [8] detected pyrethroids-resistant *Ae*. *aegypti* and *Ae*. *albopictus*, based on their resistance ratios and mortality percentages in Penang and Kuala Lumpur.

Mutations and modifications in the knockdown-resistant gene (*kdr*) of the voltage-gated sodium channel (VGSC) prompt pyrethroid insensitivity in mosquitoes. This mutation in VGSC diminishes the knockdown effect of the insecticide [9]. In Malaysia, F1534C and V1016G *kdr* gene sites confer resistance to pyrethroids [8]. Recently, a novel mutation, A1007G, was discovered in *Ae*. *aegypti* populations in Malaysia [10]. Certain detoxifying enzymes, such as Cytochrome P450 (CYP450), glutathione S-transferases (GST), and carboxylesterases (CCE), naturally degrade toxic compounds into non-toxic forms before removing them from the insect body [9]. This mechanism depends on the overproduction of detoxifying enzymes. Hence, gene expression studies using microarrays of detoxifying enzymes conducted in French territories in South America and the Caribbean have revealed a metabolic insecticide-resistant mechanism in *Ae*. *aegypti*. Microarray analysis demonstrated the involvement of CYP450 genes in conferring resistance [9]. Other genes involved in pyrethroid resistance are carboxylesterases and glutathione S-transferases (GST) [11–14]. Dusfour et al. [9] reported cytochrome gene variants CYP6BB2, CYP6M11(Q16WQ7), CYP6N12(Q16WR9), CYP9J9(Q174T1) CYP9J10, and carboxylesterase gene variant CCE3 were found to be overexpressed in *Ae*. *aegypti* deltamethrin-resistant strain identified in French Guiana, Guadeloupe, and New Caledonia. Furthermore, 3 carboxylesterases and 2 GST families gene variants were also overexpressed in the above region. Ishak et al. [15] confirmed the overexpression of CYP450 and CYP6P12 genes exerts pyrethroid resistance in Malaysian *kdr*-free *Ae*. *albopictus* populations.

Globally, there are limited proteomics studies on insecticide resistance in *Ae*. *aegypti*. Recent proteomics studies by Epelboin et al. [16] and Sun et al. [17] showed the abundance expression of one or more CYP450 genes in pyrethroid-resistant *Ae*. *aegypti*. These enzymes expression suggests the metabolic resistance mechanism triggered by the overexpression of esterase and CYP450 activity and detoxification. Furthermore, Epelboin et al. [16] also showed the mutations at the sodium voltage-gated channel gene associated with *kdr* in insecticide-resistant *Ae*. *aegypti*.

In Malaysia, *Ae*. *aegypti* is a primary vector in all urban settings with reported insecticide resistance [18,19]. However, there is much less insecticide resistance proteomics for the differentially expressed proteins study in Malaysia. Hence, we focused on *Ae*. *aegypti* adult and larvae stages to explain protein expression involved in insecticide resistance.

This study utilised LC–ESI–MS/MS for protein identification and quantification. Furthermore, we used the label-free quantitative proteomics (LFQ) approach to quantify differentially expressed proteins in permethrin and temephos-resistant *Ae*. *aegypti*. We utilised the electrospray ionisation (ESI) system coupled to a quadrupole-orbitrap MS to parallel accumulate a serial fragmentation acquisition. This model of tandem MS enhanced the speed and sensitivity by increasing the detection of overall mass intensity and resolution for better proteome coverage and quantification. The significant proteins identified in this study might be used as new biomarkers to predict and monitor *Ae*. *aegypti* insecticide resistance in the field.

## 2. Materials and methods

### 2.1. Mosquito strains

The sampling was done on Penang Island, a part of Penang state, located on the west coast of Peninsular Malaysia. The sampling areas were determined using the idengue website version 3.0 hosted by the Ministry of Science, Technology Innovation (MOSTI), Malaysia https://idengue.mysa.gov.my/ to distinguish between dengue hotspots and non-hotspot areas. Dengue hotspots are areas with continuous dengue fever outbreaks for over a month [20]. The hotspot and the non-hotspot areas selected in this study were the Taman Free School, George Town (5° 24′ 13.9″ N, 100° 18′ 34.96″ E), and Perumahan Medan Angsana Ayer Itam (5° 23′ 19″ N, 100° 16′ 58″ E). The sampling sites were chosen based on the insecticides spraying using thermal fogging areas declared dengue hotspots. The laboratory strain of *Ae*. *aegypti* was obtained at the Vector Control Research Unit (VCRU), Universiti Sains Malaysia, and used as a reference strain for standard susceptibility testing against permethrin and temephos.

### 2.2. Collection and rearing of *Ae*. *aegypti* mosquitoes

Sample collection was conducted using mosquito ovitraps. The ovitrap is a black, cylindrical plastic container filled with water [21]. A wooden paddle with a 15 cm in length was laid diagonally inside the ovitrap for female mosquitoes to lay eggs. The ovitraps were distributed five days after the fogging in the sampling area. Standard ovitraps containing 200 ml of dechlorinated water were used to collect the field *Ae*. *aegypti* mosquito strains. The ovitraps were placed randomly in each sample area two meters above the ground. All ovitraps were transported to the insectary facility at the School of Biological Sciences, Universiti Sains Malaysia (USM) Penang, after five days. The water containing the larvae and the wooden paddles with the eggs were transferred into an enamel tray filled with dechlorinated water.

### 2.3. Permethrin and temephos bioassays

About one hundred 1^st^ instar larvae were transferred to a 3-litre enamel tray containing 2 litres of water after 24-hour incubation, to decongest the enamel trays. Larvae food composed of dog biscuit powder, beef liver, powdered milk, and yeast in a 2:1:1:1 ratio was used to feed the larvae. The mosquitoes were reared under the optimum conditions of approximately 28°C with 75% relative humidity.

An adult bioassay was conducted according to WHO guidelines [22,23]. All the adult female *Ae*. *aegypti* strains were tested against 0.75% permethrin in five replicates of 20 non-blood-fed mosquitoes from 3 to 5 days old. Silicone oil-impregnated papers were used for pyrethroid control.

Mosquitoes were aspirated into the holding tubes and be rested for an hour. Dead, injured, or damaged mosquitoes were replaced with healthy ones after the one-hour resting time. After this pre-test period, the mosquitoes were blown into the exposure tubes lined with 0.75% permethrin insecticide test paper with a 12 cm x 15 cm dimension. The knockdown (*kd*) was assessed every 5 minutes during the 60-minute hold in the exposure chamber and then blown into the clean chamber for recovery assessment. A 10% sucrose solution-soaked cotton was provided to the mosquitoes as an energy source and kept at room temperature of 25°C ± 2°C with 80% ± 10% relative humidity for 24 hours. Once the recovery period was completed, the mortality was assessed at 24 hours was recorded for each test. Mortality percentages were assessed 24 hours after exposure in WHO tubes. The WHO criteria was used to determine the susceptibility status: (1) Mortality between 98 to 100% is considered susceptible, (2) Mortality < 98% indicates resistance suspect, (3) Mortality between 90 to 97% indicate the presence of resistance genes but must be confirmed by additional bioassay test or molecular methods, (4) Mortality < 90% indicate insecticide resistance [22].

The bioassays were performed in five replicates for each experiment. In total, 100 mosquitoes were used in each batch of the bioassay experiment. They were divided into 4–5 replicates of 20–25 mosquitoes per insecticide concentration as recommended by WHO [23]. This study used 20 mosquitoes per replicate unit to give a 0–100% mortality range. Based on the WHO definition, mosquitoes scored a knockdown when they could not stand or take off within 60 minutes. Meanwhile, mosquitoes were dead when they were immobile or unable to stand or take off at least 24 hours after exposure to the insecticide, respectively [22].

For the larval bioassay, a 31.25 mg/l temephos insecticide stock solution (Abate, Malaysia) obtained from the Vector control unit (VCRU), Universiti Sains Malaysia was diluted with water into eight different concentrations: 0.050 mg/l, 0.075 mg/l, 0.100 mg/l, 0.125 mg/l, 0.150 mg/l, 0.175 mg/l, 0.200 mg/l and ethanol as control. Then, they were tested on 20 mixed late 3^rd^ and early 4^th^ instar stages in five replicates. The mortality was calculated after 24 hours of exposure to the temephos. Larvae that could not swim up to the surface were scored dead, and the pupated larvae were removed from the test. The lethal concentration that kills 50% of the tested larvae (LC_50_) was calculated using Probit analysis with SPSS version 27 statistical software. For further analysis, resistant mosquitoes that survived the bioassay tests were kept at -80°C.

Based on the LC values, the Resistance Ratio (RR) was determined using the formula as shown below:

$$RR=\frac{LC50\;of\;field\;strain\;}{LC50\;of\;a\;laboratory\;strain\;}$$

RR values interpreted as susceptible, moderate resistance and highly resistance when the values are <5, 5–10, and >10, respectively.

### 2.4. Protein extractions

Twenty female adult mosquitoes and larvae from each group in triplicates were used, and details of the group classification are shown in Table 1. The extractions were performed on the field strain of the hotspot area and laboratory strain as a control. CytoBuster reagent was used to extract adult mosquito proteins by following Shettima et al. [24]. Meanwhile, the trichloroacetic acid (TCA) acetone precipitation method was used for the larvae proteins extraction [24]. About 600 μl CytoBuster reagent (Sigma, Germany) buffer was used for the mosquito homogenisation in a mini bead beater using 0.5 mm zirconia beads at 50 rpm for 5 minutes at room temperature. The pellets were centrifuged for 5 minutes at 16,000 × *g* at 4°C, and the supernatant was collected for analysis.

Proteins from twenty larvae of mixed late 3^rd^ instars to early 4^th^ instar *Ae*. *aegypti* larvae were utilised for the TCA acetone precipitation extraction method described by Wang et al. [25] and modified by Shettima et al. [26].

The larvae were homogenized in 10% TCA/cold acetone and 10 mM dithiothreitol (DTT), and incubated overnight at -20°C. Then it was centrifuged at 4°C, 15,000 × *g* for 5 minutes and the pellets were resuspended in lysis buffer (7M urea, 2M thiourea, 4% 3-[(3-cholamidopropyl) dimethylammonio)-1-propanesulfonate (CHAPS) containing 1 mM phenylmethylsulphonyl fluoride (PMSF), 2 mM ethylenediaminetetraacetic acid (EDTA) and 10 mM DTT. The mixture was sonicated for 1 minute, with a 10-second pulse and 10-second stop, and centrifuged at 4°C, 15,000 × *g*, for 5 minutes. The supernatant was reduced and alkylated with 10mM DTT and 55mM iodoacetamide (IAA). Then the sample was precipitated with chilled acetone (1:4) and incubated at -20°C overnight. Then, the precipitant was resuspended in 10 mM Tris- hydrochloric acid (HCL), sonicated for 1 minute, with a 10-second pulse and 10-second stop, and centrifuged at 4°C, 15,000 × *g* for 5 minutes.

The supernatants from both extraction methods were collected to determine protein concentration using Bio-Rad’s RC DC reagent. Approximately 1X final protease inhibitor concentration was added to the protein extracts and stored at -20°C.

### 2.5. In-gel protein digestion

About 40 μg of proteins from each group in triplicates were electrophoresed at 110V for 15 minutes. The electrophoresis was stopped when the protein complexes were trapped at the top of the separating gel. The gel was stained with RAMA stain on a rocker for 1 hour and subsequently rinsed with ddH_2_O until the trap protein band was visible. The thick protein band trapped in the gel was cut into small pieces and destained with a destaining solution made from ammonium bicarbonate, acetonitrile (ACN) and ultrapure water. The in-gel digestion was performed as Shettima et al. [24] described. First, the gel pieces were incubated at 37°C for 30 minutes with shaking at 300 rpm, and the solution mixture was discarded. The in-gel protein was reduced with 10 mM DTT at 60°C for 30 minutes and alkylated with 55 mM IAA for 60 minutes in the dark at RT. The proteins’ gel pieces were shrunk with ACN for 15 minutes at RT. In-gel digestion was performed using 12.5 ng/μL MS-grade trypsin (Promega, Madison, WI, USA) at 37°C overnight with shaking at 300 rpm. The gels were briefly centrifuged, and the peptides were extracted. The extracted peptides were pooled, and speed-vacuumed (Eppendorf, Germany) until dried to the desired volume and desalted using Zip-Tips (Merck Millipore, Burlington, MA, USA).

### 2.6. LC-ESI-MS/MS Analysis

The mass spectrometry was performed at the Proteomics and Metabolomics (PROMET) laboratory, Malaysian Palm Oil Board (MPOB). The digested peptides were reconstituted in 30 μl of 0.1% formic acid (FA) and 5% ACN. About 2 μl of the 0.2 μg/μl peptides of each group in triplicates were loaded onto an Acclaim PepMap 100 C18 column (2 μm, 0.075 ×150mm) (Thermo Scientific, MA, USA). The reverse-phase column was equilibrated with 0.1% FA (mobile phase A) and 80% ACN containing 0.1% FA (mobile phase B). The gradient mobile phase B of 5 to 35% in 75 minutes was applied for peptide elution at a 300 mL/minute flow rate. The peptides were separated using the EASY-nano liquid chromatography (EASY-nLC) 1200 System (Thermo Scientific, MA, USA). An online Q Exactive Plus Hybrid Quadrupole-Orbitrap mass spectrometer system (Thermo Scientific, MA, USA) generated the peptide ions with a spray voltage of 1900V in positive mode. A precursor ion scan was conducted with a resolution of 70,000 and a mass range of *m/z* 310–1800. Precursors containing charge states from 2 + to 8 + were further fragmented. The fragmentation was done via collision-induced and high-energy collision-induced (CID and HCD) at normalised energy of 28%. The resolution, isolation window, and ion injection time were set at 17,500, 0.7 Da, and 60 minutes. The scanned precursor mass range was set at *m/z* 110–1800.

Mass spectra of the peptides were acquired using Tune (Ver. 2.11 QF1 Build 3006) (Thermo Scientific, MA, USA) and deconvoluted with Proteome Discoverer (Ver. 2.4) (Thermo Scientific, MA, USA) to create the peptide mass list. SEQUEST HT search engine, incorporated in the Proteome Discoverer, matched the generated mass list against *Ae*. *aegypti* FASTA sequences obtained from UniProt released 2020–01 for mosquito proteins. The peptides’ mass tolerance and fragments were fixed at 10 ppm, and 0.02 Da. Trypsin was indicated as the digestion enzyme used, with up to two miscleavages allowed during the search. Carbamidomethylation on cysteine residues was set as a static modification. The variable amino acid modifications included deamidation (asparagine and glutamine residues) and oxidation (methionine residues). The mass list was also searched against a decoy database generated by randomised protein sequences. The identified proteins must have a Rank 1 peptide and a false discovery rate of <1% to be accepted. Spectra matching the sequences was further validated with the Percolator algorithm (Ver. 2.04) using *q* -value at a 1% false discovery rate.

### 2.7. Differential protein expression analysis

Statistical analysis was performed using Perseus 1.5.1.6., referring to a method described by Tyanova et al. [27]. The peptide ion intensity data were uploaded in triplicates of the groups mentioned in Table 1 for statistical analysis. The other non-statistical data were also uploaded, i.e., protein name, accession number, number of peptides, coverage, and functional ontologies. Differential protein expression analyses were performed for the adult and larval groups. A comparison was also performed to identify the number of differentially expressed proteins in each group.

**Table 1:** Classification of groups for differential protein expression analysis using ANOVA of *Ae*. *aegypti* field strain from the dengue hotspot area and laboratory strain.

Group No.	Description
**Adult**	
1	Adult field permethrin-resistant strain
2	Adult field strain that was not exposed to permethrin
3	Adult laboratory strain that was exposed to permethrin
4	Adult laboratory strain that was not exposed to permethrin
**Larvae**	
1	Larvae field temephos-resistant strain
2	Larvae field strain that was not exposed to temephos
3	Larvae laboratory strain that was exposed to temephos
4	Larvae laboratory strain that was not exposed to temephos

For the analysis of variance (ANOVA), all four groups in adult and larvae *Ae*. *aegypti* were compared (Table 1). The expression values, and the peptide ion intensity data, were matched and made logarithmic using the transformation formula log2(x). Unnecessary, incorrect, and inadequate protein identification from the primary data frame was filtered out using valid values percentage-based at 70%. Then, the columns were renamed accordingly. Annotations were specific for *Ae*. *aegypti* ontology was downloaded from https://vectorbase.org/vectorbase/app/, a VEuPathDB project release 53, 5 August 2021, installed onto Perseus annotation configuration for easy retrieval using the add annotations columns. The annotation included biological processes, molecular function, cellular component, and KEGG. Then categorical annotations were assigned to those rows, respectively. The imputation algorithm replaced missing values from the normal distribution with Perseus default settings of width 0.3 and downshifted 1.8. A histogram was plotted for each intensity column separately to verify whether the data were normally distributed. Quality checking was performed to determine the similarity of the same and different groups using multi-scatter plots. Pearson correlations were plotted to analyse the correlations between and within the groups. Also, the hierarchical clustering of the protein intensities was plotted for all samples. The analysis tab generated expression profile plots and principal component analysis (PCA) using protein intensities as a variable. Finally, Fisher’s exact functional enrichment test was performed from the ANOVA significant proteins. An ANOVA (q<0.05) and Fisher exact test functional enrichment at (q<0.05) were considered differentially significant.

In the student’s t-test analysis, the comparison groups were adult field permethrin-resistant strain versus adult laboratory strain exposed to permethrin and larvae field temephos-resistant strain versus larvae laboratory strain exposed to temephos. After transforming the expression values based on ion intensity and filtering out valid values based on percentage, missing values were replaced from the normal distribution. *Ae*. *aegypti* ontology annotation was added, and histogram and multi-scatter plots and Pearson correlations were plotted for group similarities, differences, and correlations. A two-sample t-test was used to identify the interactors using s0 = 0 and FDR = 0.01. In this analysis, the s0:0 and FDR.: 0.01 parameters of the volcano plots were used to show the cut-off curve, indicating the significantly different proteins in abundance for groups. The volcano plot was generated with a t-test difference on the y-axis against the -log t-test p-value on the x-axis. Proteins with a t-test difference of >2.0-fold changes and q value < 0.05 were considered significant.

### 2.8. RNA extraction and cDNA synthesis

The RNA extraction was performed using NucleoSpin RNA Plus (Macherey-Nagel, Germany) using eight *Ae*. *aegypti* female adult mosquitoes from each permethrin and larvae temephos resistant from the hotspot and non-hotspot areas. Furthermore, the laboratory strains exposed to permethrin and temephos were used as controls. The cDNA conversion kit ReverTra Ace qPCR RT Master Mix with gDNA Remover (Toyobo CO., Ltd, Osaka, Japan) was used in this study. The RNA template was incubated at 65°C for 5 minutes and kept on ice. About 2 μl of 4x DN Master Mix, 1 μg RNA template and Nuclease free water were added to make a total of 8 μl. The mixture was then incubated at 37°C for 5 minutes. Then 2 μl of 5x room temperature Master Mix II was added to the 8 μl mixture and incubated at 37°C for 15 minutes, 50°C for 5 minutes, and 98°C for 5 minutes.

### 2.9. Primer design

For the validation, proteins with ≥2-fold change from the t-test (q<0.05) (Table 2), showed significant expression from the ANOVA (q<0.05), and having unique peptide ≥2 were selected for this analysis. Coding sequences (CDS) of these proteins in FASTA format were obtained from the VEuPathDB vector base https://vectorbase.org/vectorbase/app/. Primers ACT and RPS17 were previously described by Dzaki et al. [28] and used as reference genes for qPCR in *Ae*. *aegypti*. The remaining primers were designed using the Primer 3 software https://bioinfo.ut.ee/primer3-0.4.0/. Restrictive parameters for primer selection were melting temperatures (T_m_) between 59°C and 68°C, with an optimal T_m_ between 63°C and 64°C. GC content between 40 and 60%, primer length between 18 and 24 bp, and amplicon length between 80 to 200 bases.

**Table 2:** Primer sequences of the selected and reference genes.

Protein	Primer sequence	Product size
ACT (reference) [28]	FW 5´-CGTTCGTGACATCAAGGAAA-3´	175 bp
	RV 5´-GAACGATGGCTGGAAGAGAG-3´	
RPS17 (reference) [28]	FW 5´-AAGAAGTGGCCATCATTCCA-3´	225 bp
	RV 5´-GGTCTCCGGGTCGACTTC-3’	
Enolase phosphatase E1 (Q17Q32)	FW 5´-ACGCGCTGAAACACGTCGAA-3´	82 bp
	RV 5´-TCGCGCAGAGCAGCAACAAC-3´	
Na^+^/K^+^ ATPase β2 (Q16RY8)	FW 5´-CGTTGAAGCCTGGACGACGC-3´	99 bp
	RV 5´-TCTGGCGGTGGGTTGTCGAA-3´	
Glucosidase 2 subunit β (Q16M80)	FW 5´-GGTCCAGGCGAGGCAGAGAA-3´	94 bp
	RV 5´-CGAGGACGAGGCAACTCCGT-3’	
Troponin I (Q16RS3)	FW 5´-AGCGGAACGTAGGCGCATCA-3’	104 bp
	RV 5´-TGGCGTGATAGTCGCGGAGG3-’	
H15 domain containing protein (A0A6I8TV54)	FW 5´-GCAAGCCGAAGAAGCCGTCG-3´	132 bp
	RV 5´-CGCATTTGTAGTTGGCGGCGA-3´	
60S ribosomal protein L21(Q1HRN4)	FW 5´-CGCGCTGCCGTGAGGATTTC-3’	120 bp
	RV 5´-CGCGAGGTGCTTGTGGCTTG-3´	
AAEL01756-PB (J9HFM9)	FW 5´-GCTGGTGCTTCCGTCGATGC-3’	97 bp
	RV 5´-CCGCTGTTGGCTTCTTGGCT-3´	
Tubulin β chain (A0A1S4F2Y4)	FW 5´-GCCAAACCCAAGCTACGGCG-3´	138 bp
	RV 5´-AGACGTGGGAACGGCACCAT-3’	

Note: Oligo dt primers were used reverse transcription. The primers for the PCR are those listed in this table.

### 2.10. Gene expression

The PCR mixture was made for 20 μl per reaction using Thunderbird Next SYBR qPCR mix (Toyobo CO., Ltd, Osaka, Japan). Standard PCR reaction set up containing a 10 μl Thunderbird Next SYBR qPCR Mix, 0.6 μl each of forward and reverse primer with the final concentration of 0.3 μM, 7.8 μl of PCR water, and 1 μl of cDNA. The qPCR (Rotor-Gene Q, Qiagen, Hilden Dusseldorf, Germany) was run using a thermal parameter with an initial denaturation at 95°C for 2.30 minutes, followed by 40 cycles of denaturation at 95°C for 20 seconds, annealing at 59°C for 20 seconds and extension at 72°C for 15 seconds. After a final extension at 72°C for 20 seconds, the melting curve analysis was performed automatically for quality checking to verify that only a single PCR product amplified. All samples were amplified in triplicates. ACT and RPS17 were used as internal control/reference genes [28]. The significant gene fold-change (Fc) > 2.

An equation from Vandesompele et al., [29] was used for the relative gene expression calculation using combination of two reference genes. The equation provides the fold change difference between the untreated and the treated samples.

When the C_T_ value showed a down-regulation pattern, the formula below was used to determine the fold change.


$$\frac{-1}{2-\Delta \Delta \;Ct}.$$

### 2.11. Prediction of protein-protein interaction (PPI) network and functional ontology enrichment

The STRING software database version 11.0b, released on 17 October 2020, was used to predict PPI network associations of the differentially expressed proteins of ANOVA analyses in permethrin- and temephos-resistant *Ae*. *aegypti*. This study selected the network edge by ‘evidence’, where the coloured lines indicated the type of interaction evidence. The sources of active interactions included ‘text mining’, ‘experiment’, ‘databases’, ‘neighbourhood’, ‘gene fusion’, ‘co-occurrence’, ‘co-expression’, and ‘protein homology’. The medium confidence of 40% was selected as the minimum required interaction score. A Markov clustering algorithm (MCL) with three inflation parameters was applied with 5% FDR stringency in a complete STRING network. The functional ontology enrichments in the protein network were retrieved using the analysis tool incorporated in the software. The analysis also contains the enriched KEGG pathways. STRING uses the protein accession number from the VectorBase database VEuPathDB for analysis. In addition, this accession number is related to the accession number of the UniProt database, referring to the same protein description.

## 3. Results

### 3.1. Susceptibility status and resistant pattern of *Ae*. *aegypti*

The *Ae*. *aegypti* strain from the field hotspot indicated KT_50_ of 29.041 and KT_99_ of 54.567 minutes (Table 3). Meanwhile, field strain from the non-hotspot area showed KT_50_ and KT_99_ 151.225 and 501.375 minutes, respectively (Table 3). The highest KT was observed in the field strain from the non-hotspot site. The overall mortality was 28%, and 53% in the field strains from the hotspot and non-hotspot regions, respectively (Table 4). The laboratory strain exhibited the lowest knockdown time KT_50_ of 7.9 minutes and KT_99_ of 17.138 minutes, and the exposure effect showed 100% mortality (Tables 3 and 4). Overall, the resistance ratio (RR) indicated resistance towards permethrin in the field strain compared to the laboratory strain (Table 3). Additional bioassay data can be found in S1 Table (a-c).

The larvae of *Ae*. *aegypti* field strain from the hotspot area showed the highest LC_50_ and LC_99_ of 0.153mg/l and 0.543mg/l (Table 3). Meanwhile, 0.148mg/l and 0.405mg/l were obtained from LC_50_ and LC_99_ of the non-hotspot field strain, respectively (Table 3). The discriminating concentrations were 1.086mg/l and 0.810mg/l in the field strains from the hotspot and non-hotspot areas. The result also indicated the lowest lethal concentration (LC) in the laboratory strain with LC_50_ and LC_99_ of 0.004mg/l and 0.009mg/l, with the discriminating concentration being 0.018mg/l (Table 3). The mortality percentages of the *Ae*. *aegypti* larvae from the field hotspot and non-hotspot areas indicated temephos resistance by 80% and 91% total mortality (Table 4). Also, the resistant ratio (RR) revealed 38.25 and 37.00 for the larvae field strains from the hotspot and non-hotspot areas (Table 3). Overall, the temephos resistance was higher in mosquito larvae from the dengue hotspot area than in the non-hotspot area. Additional bioassay data can be found in S1 Table (d-i).

**Table 3:** Knockdown time (min) KT_50_ and KT_99_ against 0.75% permethrin and 31.25mg/l temephos of *Ae*. *aegypti* female adults and larval from field and laboratory strains.

Strain	Insecticide	KT_50(mins)_/LC_50(mg/l)_ [95% CI]	KT_99_/LC_99_ [95% CI]	Discriminating Concentration	RR	Regression ± SD
Field hotspot	Permethrin	29.042 [2.2,3.1]	151.225mins [4.40,7.70]	-	4.00	4.479±0.431
	Temephos	0.153 [0.00,0.21]	0.543mg/l [0.30,0.63]	1.086mg/l	38.25	3.451±0.262
Field non-hotspot	Permethrin	54.567 [1.6,1.8]	503.375mins [3.10,3.90]	-	7.00	4.195±0.383
	Temephos	0.148 [0.15,0.16]	0.405mg/l [0.28,0.31]	0.810mg/l	37.00	4.408±0.293
Laboratory	Permethrin	7.909 [0.82,0.89]	17.138mins [1.23,1.36]	-	1.00	1.994±0.8755
	Temephos	0.004 [0.00,0.006]	0.009mg/l [0.07,0.01]	0.018mg/l	1.00	16.583±1.237

KT_50_: Time duration required to knockdown 50% of the population after exposure to insecticide. KT_99_: Time duration required to knockdown 99% of the population after exposure to insecticide.

**Table 4:** Insecticide susceptibility status of *Ae*. *aegypti* female adults and larvae against 0.75% permethrin and 31.25mg/l temephos.

Strain	Insecticide	% Mortality	Standard deviation	Status
Field hotspot	Permethrin	28	2.07	Resistant
	Temephos	80	1.26	Resistant
Field non-hotspot	Permethrin	53	3.57	Resistant
	Temephos	91	0.40	Resistant
Laboratory	Permethrin	100	0.00	Susceptible
	Temephos	100	0.00	Susceptible

### 3.2. Differential protein expression analyses

**Fig 1:**
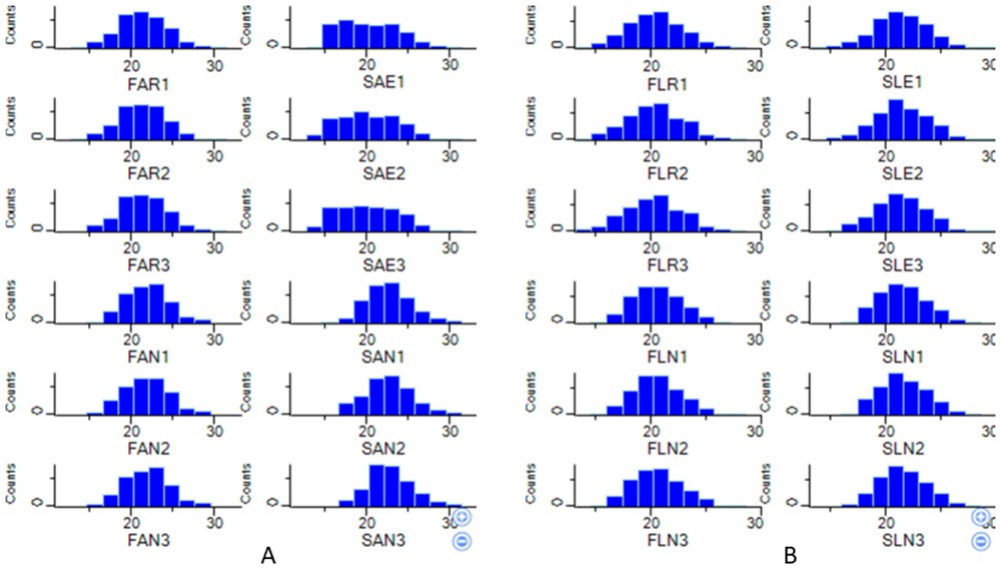
Histograms present three replicates of *Ae. aegypti* identified proteins. A. Adults. B. Larvae. Note: FAR/FLR: Adult/Larvae *Ae*. *aegypti* field permethrin resistant strain, FAN/FLN: Adult/Larvae *Ae*. *aegypti* field strain that was not exposed to permethrin, SAE/SLE: Adult/Larvae *Ae*. *aegypti* laboratory strain that was exposed to permethrin. SAN/FLN: Adult/Larvae *Ae*. *aegypti* laboratory strain that was not exposed to permethrin.

**Fig 2:**
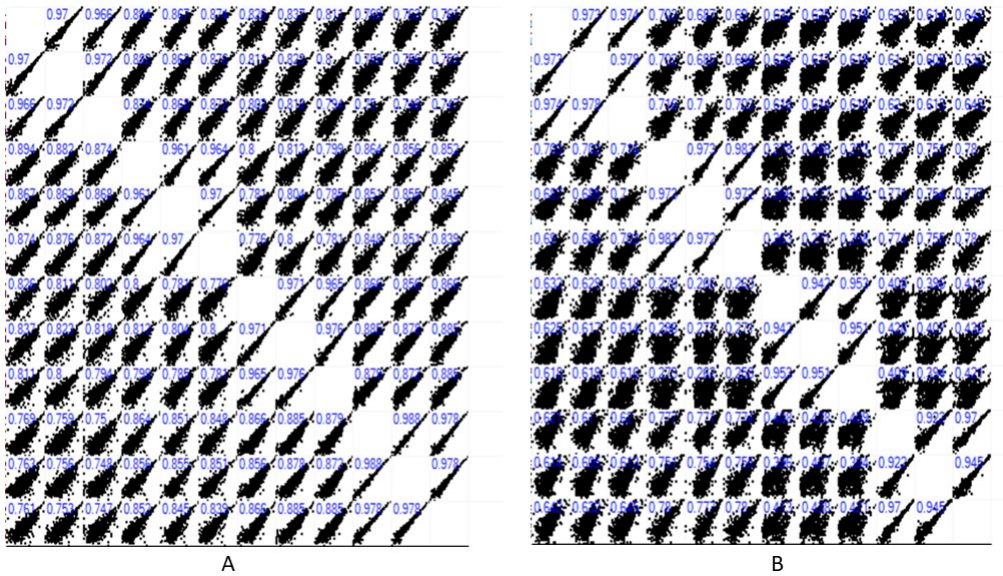
Multi-scatter plot and Pearson correlation coefficient of *Ae*. *aegypti* identified proteins. A. Adults B. Larvae.

**Fig 3:**
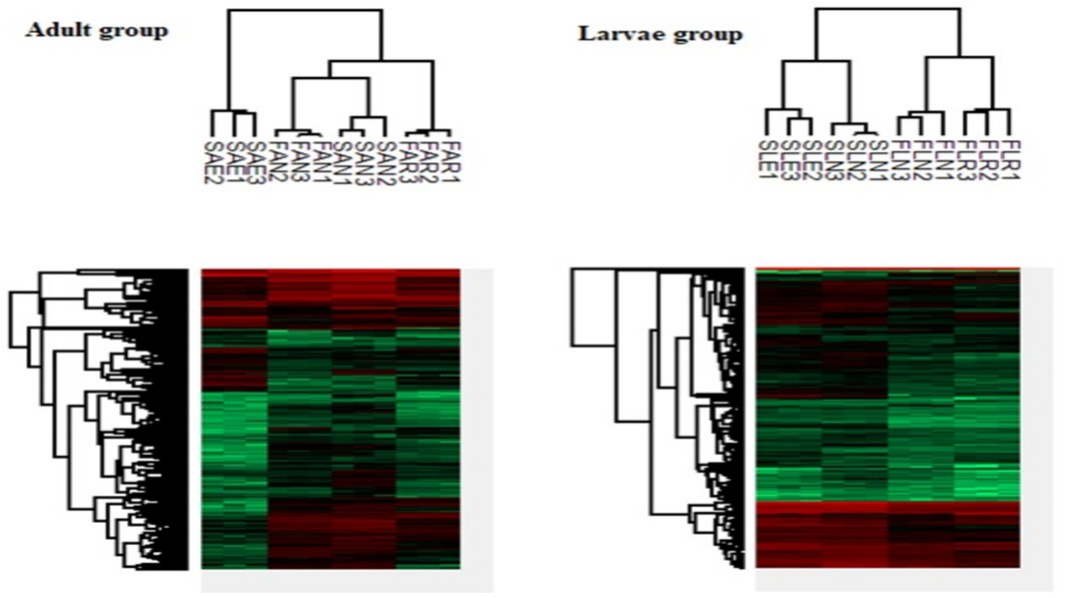
Hierarchical clustering of the identified proteins in adults and larvae *Ae*. *aegypti*. Red black, and green bars represent high: normal, and low protein expressions. Note: FAR; Field strain adult *Ae*. *aegxpti* permethrin resistant. FAN: Field strain adult *Ae*. *aegxpti* not exposed to permethrin. SAE: Laboratory strain adult *Ae*. *aegxpti* exposed to permethrin. SAN: Laboratory strain adult *Ae*. *aegxpti* not exposed to permethrin. FLR: Field strain *Ae*. *aegxpti* larvae temephos resistant. FLN: Field strain *Ae*. *aegxpti* larvae not exposed to temephos. SLE: Laboratory strain *Ae*. *aegxpti* larvae exposed to temephos. SLN: Laboratory strain *Ae*. *aegxpti* larvae not exposed to temephos. The numbers (1,2,3)that follow the initial three letters indicate the replicate.

**Fig 4:**
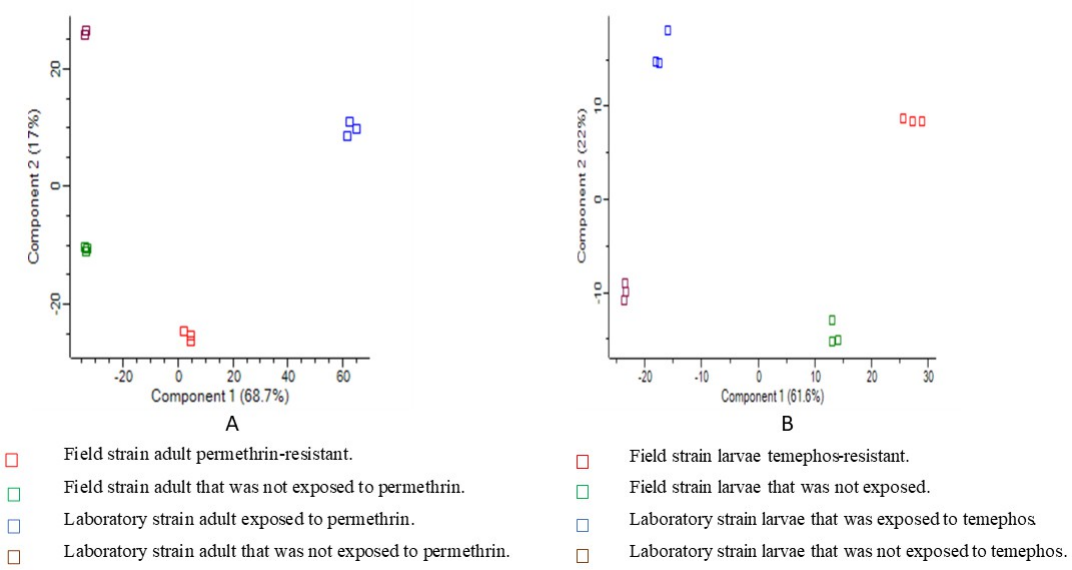
PCA of *Ae*. *aegypti* identified proteins. A. Adults B. Larvae.

In this study, the imputation revealed the normal distribution of the data for subsequent analysis, as shown in the histograms (Fig 1A and 1B). A significant Pearson correlation coefficient was observed for the protein quantification. This correlation showed comparable performance and reproducibility in the experimental samples used in this study (Fig 2A and 2B). Meanwhile, the clustering and the Principal Component Analysis (PCA) also showed different protein expression intensities in the insecticide-treated groups and the control groups that were not exposed to any insecticide treatment (Figs 3, 4A, and 4B). All the group replicates components belong to each appropriate cluster. The differential protein expression analyses were carried out using the hotspot strains for permethrin and temephos bioassay, respectively.

A total of 501 and 557 differentially expressed proteins were identified in the *Ae*. *aegypti* adult permethrin-resistant and larvae temephos-resistant from the analysis of variance (ANOVA), respectively. Details of the differentially expressed proteins can be found in S2 and S3 Tables. Among the differentially expressed proteins, we identified cytochrome complex proteins, GST proteins, motor-related proteins, insect cuticle proteins, ribosomal proteins, and heat shock proteins that might confer insecticide resistance in *Ae*. *aegypti* (S1 File, S4 Table).

**Fig 5:**
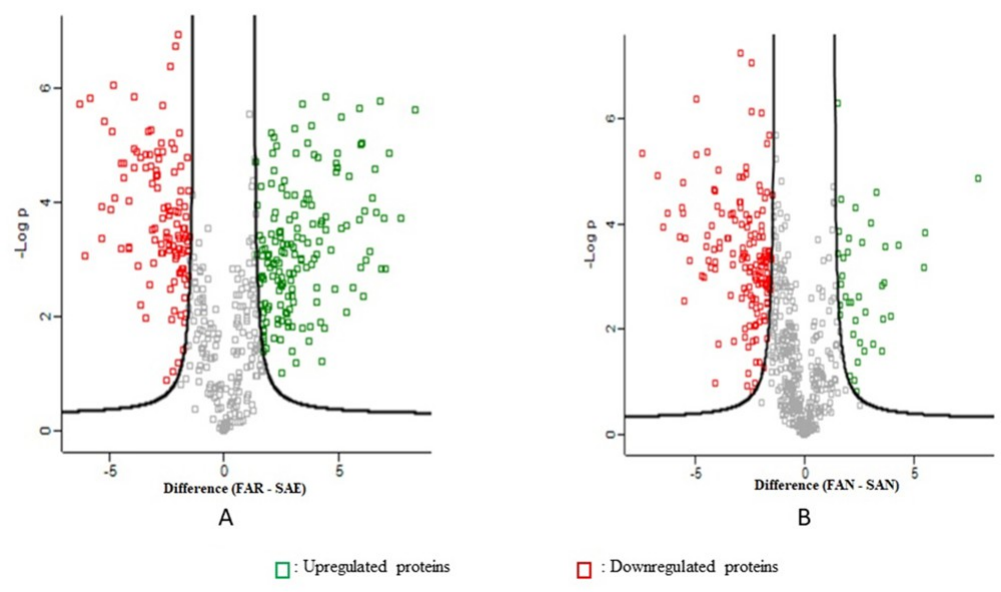
Volcano plots show differentially expressed proteins in adults *Ae*. *aegypti*. A. Field permethrin-resistant strain versus laboratory strain that was exposed to permethrin B. Field strain versus laboratory strain that were not exposed to permethrin. **Notes**: **FAR**: Field adult permethrin resistant strain **SAE**: Adult laboratory strain that was exposed to permethrin **FAN**: Field adult strain that was not exposed to permethrin. **SAN**: Laboratory adult strain that was not exposed to permethrin.

The t-test result showed 188 proteins with significant differential expression in *Ae*. *aegypti* permethrin-resistant strain from the hotspot area versus laboratory strain exposed to permethrin. The analysis revealed 114 upregulated and 74 downregulated proteins (Fig 5A) (Tables 5 and S5). In addition, a total of 113 proteins were differentially expressed in *Ae*. *aegypti* field strain versus laboratory strain that was not exposed to permethrin with 24 upregulated and 89 downregulated proteins (Fig 5B) (S5 Table).

**Table 5:** Example of the up-regulated and down-regulated proteins in adult *Ae*. *aegypti*.

**Up-regulated proteins of hotspot adult *Ae*. *aegypti* permethrin-resistant strain versus adult laboratory strain exposed to permethrin (q-value <0.05).**							
**No.**	**Accession no.**	**Description**	**SUM PEP**	**Sequence coverage (%)**	**Matched peptides**	**Unique Peptides**	**Fold change** **FC**
1	Q16NG1	Fumarylacetoacetase	108.09	48	15	15	8.35
2	Q16J86	Catalase	175.06	50	22	15	7.72
3	Q16TC0	Aliphatic nitrilase, putative	66.35	34	9	9	7.20
4	Q17N03	Fumarylacetoacetate hydrolase	78.93	50	12	6	7.04
5	Q16U43	Phosphoglucomutase	159.77	52	22	15	4.90
6	Q1HRM4	Cytochrome c oxidase subunit VIA, putative	9.93	24	2	2	3.84
7	J9HHL7	Glutathione S transferase	39.74	34	6	4	3.02
8	Q16RY8	Sodium/potassium dependent beta 2 subunit	41.11	27	6	6	2.96
9	Q16KF6	Cytochrome c oxidase subunit 4	99.90	54	9	9	2.46
10	Q1HQZ5	Heat shock cognate 70	72.74	21	11	2	2.45
**Down-regulated proteins of hotspot adult *Ae*. *aegypti* permethrin-resistant strain versus adult laboratory strain exposed to permethrin (q-value <0.05).**							
**No.**	**Accession no.**	**Description**	**SUM PEP**	**Sequence coverage (%)**	**Matched peptides**	**Unique Peptides**	**Fold change** **FC**
1	Q17H74	Tropomyosin invertebrate	92.7	34	10	2	6.28
2	Q173N8	Far upstream (fuse) binding protein	9.4	3	2	2	5.82
3	Q16XP0	Apolipophorin-III, putative	9.6	12	2	2	5.31
4	Q17Q32	Enolase-phosphatase E1	21.9	5	5	5	3.18
5	Q16M80	Glucosidase 2 subunit β	20.4	10	4	4	2.88
6	Q0IGD5	Pupal cuticle protein 78E, putative	7.4	8	2	2	2.86
7	Q17H80	Tropomyosin invertebrate	338.9	69	30	2	2.34
8	Q17BT9	Superoxide dismutase	83.9	42	8	8	2.44
9	Q16RS3	Troponin I	61.8	32	9	5	2.01
10	W0FUL2	Myosin heavy chain	951.9	47	104	2	2.12

**Fig 6:**
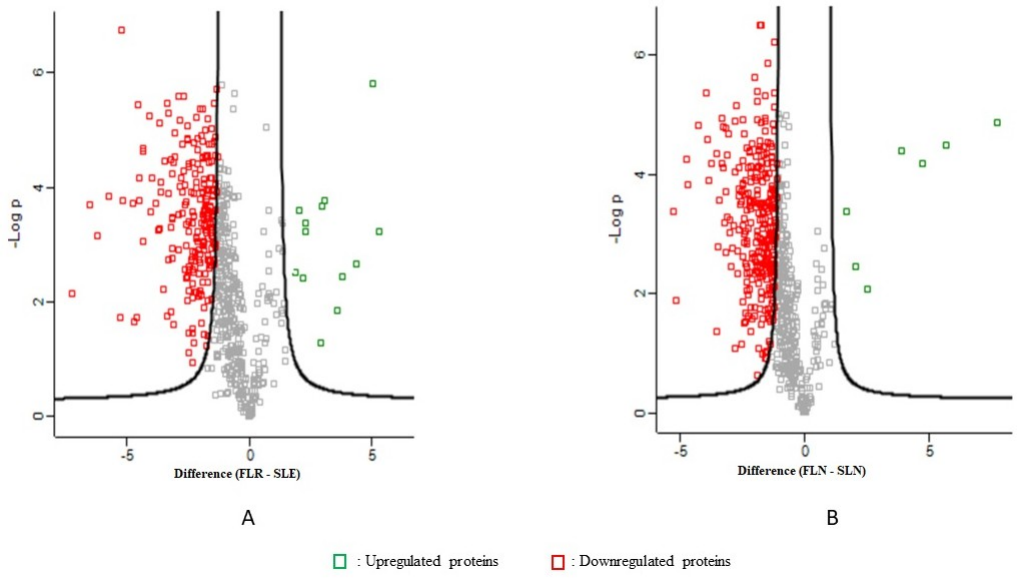
Volcano plots show differentially expressed proteins in larvae *Ae*. *aegypti*. A. Field temephos-resistant strain versus laboratory strain that was exposed to temephos B. Field strain versus laboratory strain that were not exposed to temephos. **Notes**: **FLR**: Field larvae temephos resistant strain **SLE**: Laboratory larvae strain that was exposed to temephos **FLN**: Field larvae strain that was not exposed to temephos **SLN**: Laboratory larvae strain that was not exposed to temephos.

There were 118 proteins with significant differential expression in the field strain larvae temephos-resistant strain versus laboratory strains exposed to temephos with 13 upregulated and 105 downregulated proteins (Fig 6A) (Table 6) (S6 Table). A total of 91 differentially expressed proteins of the *Ae*. *aegypti* larvae field strain versus laboratory strains not exposed to temephos indicated 7 upregulated and 84 downregulated proteins (Fig 6B) (S6 Table).

**Table 6:** Example of the up-regulated and down-regulated proteins in larvae *Ae*. *aegypti*.

**Up-regulated proteins of the hotspot *Ae*. *aegypti* larvae temephos- resistant versus laboratory strain exposed to temephos (q-value <0.05).**							
**No.**	**Accession no.**	**Description**	**SUM PEP**	**Sequence coverage [%]**	**Matched Peptides**	**Unique Peptides**	**Fold Change** **FC**
1	Q16JT5	Ribosomal protein	3.73	10	2	2	5.28
2	Q16ZH3	60S ribosomal protein L6	10.13	10	3	3	5.02
3	Q0IFL5	Argininosuccinate synthase	6.37	6	3	3	3.57
4	Q1HRN4	60S ribosomal protein L21	9.58	16	3	3	3.05
5	Q1HRP1	60S ribosomal protein L14	13.75	16	3	3	2.97
6	A0A6I8TV54	H15 domain-containing protein	66.86	30	5	5	2.92
7	Q17IE1	AAEL002372-PA	5.45	15	2	2	2.31
8	J9HFM9	AAEL017516-PB S	70.68	27	11	4	2.31
9	Q16QN5	AAEL011230-PA	20.99	18	4	4	2.20
10	Q16FB1	60S ribosomal protein L27	11.64	21	3	3	2.04
11	A0A023EIH3	Putative 60s ribosomal protein l11	9.98	12	2	2	3.80
**Down-regulated proteins of the hotspot *Ae*. *aegypti* larvae temephos resistant versus laboratory strain exposed to temephos (q-value <0.05).**							
**No.**	**Accession no.**	**Description**	**SUM PEP**	**Sequence Coverage [%]**	**Matched Peptides**	**Unique Peptides**	**Fold Change** **FC**
1	Q17D30	Eukaryotic translation initiation factor 3 subunit M	6.03	5	2	2	6.51
2	A0A6I8T2V8	Peroxisome assembly factor-2 (peroxisomal-type ATPase 1)	7.54	2	2	2	5.23
3	Q1EGF0	Lysosomal cathepsin B	7.04	6	2	2	5.17
4	A0A1S4FX57	5-aminoimidazole-4-carboxamide ribonucleotide formyltransferase	7.57	4	2	2	4.70
5	Q179I6	Trypsin	34.11	28	4	2	4.59
6	A0A1S4F2Y4	Tubulin beta chain	75.02	24	9	2	4.34
7	A0A1S4F6S3	Acyl-coa dehydrogenase	5.65	5	2	2	4.02
8	Q6Q9G8	Elongation factor 1 beta	22.23	23	5	4	3.72
9	Q175J7	Acetyl-coa acetyltransferase	30.84	20	8	8	3.67
10	J9HII6	AAEL017177-PA	7.40	3	2	2	3.62

Functional enrichment analyses by Fisher exact test revealed 80 and 4 proteins in the adult and larval hotspot strain, respectively. The functional enrichment in the adult permethrin-resistant *Ae*. *aegypti* was in the cellular component ontology (Table 7). The enriched proteins were involved in the endoplasmic reticulum/Golgi category performing protein synthesis and facilitating the delivery of the proteins to their appropriate destinations. Another enriched cellular component in the permethrin-resistant group was the “other membranes” category.

On the other hand, four functionally enriched proteins belong to the biological process, the cellular component, and the molecular function ontologies found in the hotspot temephos-resistant *Ae*. *aegypti* larvae. The four proteins were involved in the lipid metabolic process, the extracellular region, phospholipase activity, phosphatidylcholine 1-acetylhydrolase activity, lipase activity, carboxylic ester hydrolase activity (Table 7).

**Table 7:** Example of significant functional enrichment proteins in adult and larvae *Ae*. *aegypti*.

**Adult *Ae*. *aegypti* hotspot permethrin-resistant cellular component by Fisher exact test (q-value <0.05)**					
**No.**	**Accession no.**	**Description**		**Cellular component**	
1	Q1HRQ7	ATP synthase subunit alpha		Proton-transporting ATP synthase complex, catalytic core F(1) GO:0045261	
2	A0A1S4FE53	Calcium-transporting ATPase		Sarcoplasmic reticulum membrane GO:0033017	
3	Q16N74	Sodium/potassium-transporting ATPase subunit alpha		Plasma membrane GO:0005886	
4	O16109	V-type proton ATPase catalytic subunit A		Proton transporting V-type ATPase, V1domain GO:0033180	
5	Q1HR57	VDAC		Mitochondrial outer membrane GO:0005741	
6	Q16PI3	Transferrin		Extracellular space GO:0005615	
7	Q16RY8	Sodium/potassium-dependent ATPase beta-2 subunit		Sodium/potassium-exchanging ATPase complex GO:0005890	
8	Q17FM7	NADPH—cytochrome P450 reductase		Endoplasmic reticulum membrane GO:0005789	
9	Q16XG8	Cytochrome c oxidase subunit		Mitochondrion GO:0005739	
10	Q16KF6	Cyt c oxidase subunit 4		Mitochondrial respiratory chain complex IV GO:0005751	
**Larvae *Ae. aegypti* hotspot temephos-resistant biological process, cellular component, and molecular function by Fisher exact test (q-value <0.05)**					
**No.**	**Accession no.**	**Description**	**Biological process**	**Cellular component**	**Molecular function**
1	Q16TS7	Enoyl-CoA hydratase	Fatty acid beta-oxidation GO:0006635	Mitochondrial fatty acid beta-oxidation multienzyme complex GO:0016507	enoyl-CoA hydratase activity GO:0004300
2	Q16YD0	Acyl-coenzyme A oxidase	Biosynthetic activity GO:0009058	Peroxisome GO:0005777	Acyl-CoA oxidase activity GO:0003997
3	Q173Q8	Lipase	Lipid metabolic process GO:0006629	Extracellular region GO:0005576	Triglyceride lipase activity GO:0004806
4	Q16LH5	AAEL012645-PA	Insignificant	Extracellular region GO:0005576	Chitin binding GO:0008061

### 3.3. Gene expression analyses

Overall, the expression patterns revealed by the gene expression analysis corresponded to the protein expression t-test results. Enolase phosphatase E1, glucosidase 2 β, and troponin I were downregulated in the adult *Ae*. *aegypti* permethrin-resistant strain (Table 8). The sodium/potassium-dependent ATPase β2 subunit upregulation from the non-hotspot area corresponded to the protein expression t-test analysis. However, this gene showed insignificant upregulation from the adult *Ae*. *aegypti* permethrin-resistant strain of the hotspot area (Table 8).

**Table 8:** Gene expression fold change (FC) of *Ae*. *aegypti*.

**Adult *Ae*. *aegypti* permethrin-resistant strain**			
**Gene/protein/accession no.**	**Hotspot**	**Non-hotspot**	**T-test**
	**Gene expression (Fc)**		**Protein Expression (Fc)**
Sodium/potassium-dependent ATPase β2 subunit (Q16RY8)	0.45	3.76	2.96
Enolase phosphatase E1 (Q17Q32)	-2.5	-3.4	-3.18
Glucosidase 2 β (Q16M80)	-2.3	-3.8	-2.88
Troponin I (Q16RS3)	-2.6	-2.0	-2.01
**Larvae *Ae*. *aegypti* temephos-resistant strain**			
**Gene/protein/accession no.**	**Hotspot**	**Non-hotspot**	**T-test**
	**Gene expression (Fc)**		**Protein expression (Fc)**
H15 domain containing protein (A0A6I8TV54)	0.54	0.43	2.92
60S ribosomal protein L21 (Q1HRN4)	1.82	2.02	3.05
AAEL01756-PB (J9HFM9)	0.84	2.72	2.31
Tubulin β chain (A0A1S4F2Y4)	-142.9	-10.0	-4.43

Note:—(minus): downregulated proteins.

The gene expression pattern in the larvae *Ae*. *aegypti* temephos-resistant strain showed the upregulation of H15 domain-containing protein, 60S ribosomal protein L21, AAEL01756-PB, and the downregulation of tubulin β chain from the hotspot and non-hotspot areas (Table 8). This gene expression analysis in the temephos-resistant *Ae*. *aegypti* also confirms similar protein expression patterns by t-test (Table 8) except for the H15 domain containing proteins showed an insignificant upregulation, while 60S ribosomal protein L21, AAEL01756-PB were upregulated.

### 3.4. Predictive PPI and functional ontology enrichment

**Fig 7:**
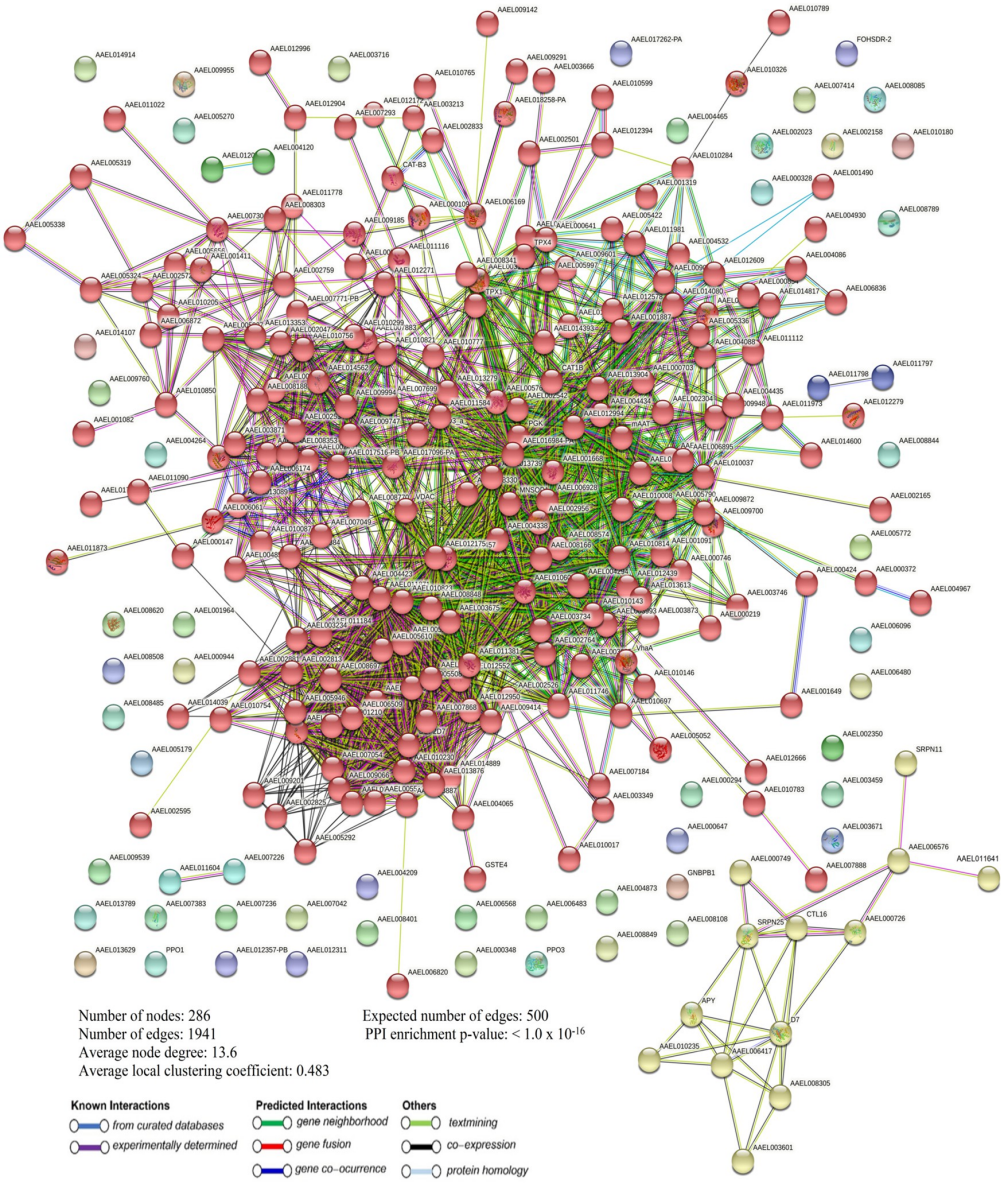
Differentially expressed proteins PPI in permethrin-resistant *Ae*. *aegypti*.

**Fig 8:**
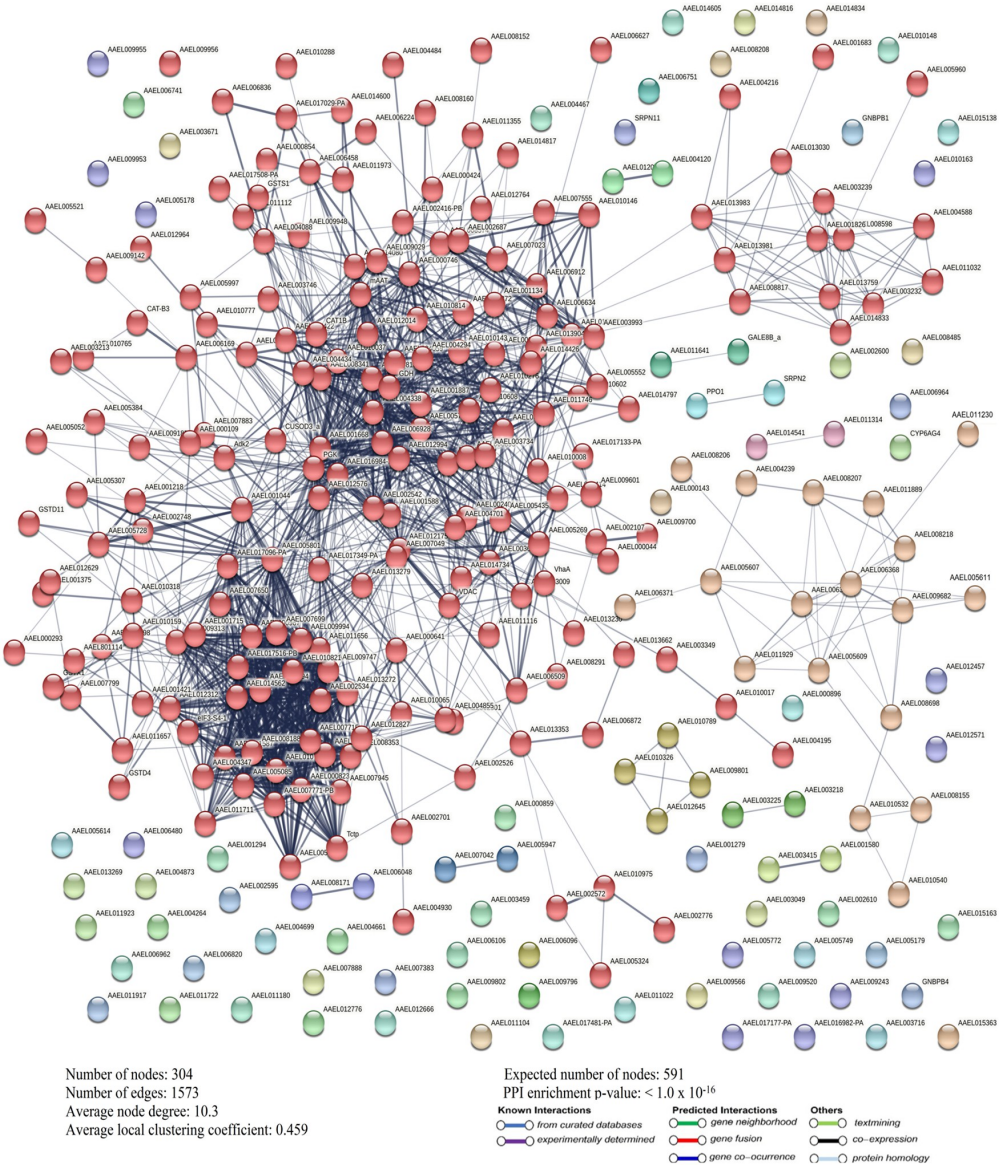
Differentially expressed proteins PPI in temephos-resistant *Ae*. *aegypti*.

STRING analysis of the differentially expressed proteins from the permethrin-resistant adult *Ae*. *aegypti* strain revealed 286 nodes, 1941 edges, an average node degree, and a local coefficient of 13.6 and 0.483 (Fig 7). In the temephos-resistant larvae *Ae*. *aegypti* strain, the differentially expressed proteins showed 304 nodes, 1573 edges, and an average node degree and local coefficient of 10.3 and 0.459 (Fig 8). A significant PPI was observed in permethrin, and temephos-resistant differentially expressed proteins with a p-value of <1.0 x 10^−16^ (Figs 7 and 8), suggesting that the network displayed significantly more interactions than expected for the differentially expressed proteins.

STRING analyses revealed functional ontology enrichment in the permethrin-resistant *Ae*. *aegypti* differentially expressed proteins, including biological processes and molecular function. The biological process showed three categories: the metabolic drug process (GO:0017144), the small molecule metabolic process (GO:0044281), and the nucleobase-containing small molecule metabolic process (GO:0055086). The small molecule metabolic process category revealed the highest gene counts of six, followed by the drug metabolic process category with five gene counts and four gene counts in the nucleobase containing the small molecule metabolic process category. Furthermore, the molecular function ontology also showed three categories: catalytic activity (GO:0003824), hydrolase activity (GO:0016787), and hydrolase activity acting on ester bonds (GO:0016788) (Table 9). The catalytic activity revealed eight gene counts, followed by five and three gene counts in hydrolase activity and hydrolase activity acting on ester bonds.

**Table 9:** Functional ontology enrichment of differentially expressed proteins in adult *Ae*. *aegypti* permethrin-resistant strain.

No.	Term ID	Description	Gene count	FDR	Matching proteins
	**Biological process**				
1	GO:0017144	Drug metabolic process	5	0.0064	AAEL000109, AAEL002956, AAEL003161, VhaA, AAEL012172
2	GO:0044281	Small molecule metabolic process	6	0.0064	AAEL000109, AAEL002956, AAEL003161, APY, VhaA, AAEL012172
3	GO:0055086	Nucleobase-containing small molecule metabolic process	4	0.0323	AAEL003161, APY, VhaA, AAEL012172
**No.**	**Molecular function**				
1	GO:0003824	Catalytic activity	8	0.03	AAEL000109, AAEL002956, AAEL003161, AAEL006169, APY, VhaA, c, AAEL012172
2	GO:0016787	Hydrolase activity	5	0.03	AAEL000109, AAEL006169, APY, VhaA, AAEL010326
3	GO:0016788	Hydrolase activity, acting on ester bonds	3	0.03	AAEL000109, APY, AAEL010326

Notes: AAEL000109- enolase-phosphatase E1, AAEL002956-probable citrate synthase 1, AAEL003161-adenylosuccinate synthetase, VhaA-V-type proton ATPase catalytic subunit A, AAEL012172-methylthioadenosine phosphorylase, APY-apyrase, AAEL006169-lysosomal aspartic protease, AAEL010326-phosphodiesterase-related protein.

The functional enrichment of the temephos-resistant larvae differentially expressed proteins indicated seventeen biological processes and one cellular component ontology (S7 Table). Noteworthy, the top five functional enrichment of the protein network were metabolic process (GO:0008152) with eleven gene counts, organonitrogen compound metabolic process (GO:1901564) with ten gene counts, primary metabolic process (GO:0044238) with ten gene counts, cellular metabolic process (GO:0044237) with nine gene counts, and cellular nitrogen compound metabolic process (GO:0034641) with eight gene counts. The enriched cellular component was cytoplasm (GO:0005737) with nine gene counts.

The enriched KEGG pathways in the permethrin-resistant differentially expressed proteins revealed 45 pathways (S9 Table). The pathways with the highest gene counts included metabolic pathways with 102 gene counts, oxidative phosphorylation with 35 gene counts, carbon metabolism with 30 gene counts, biosynthesis of amino acids with 16 gene counts, and glycolysis with 14 gene counts. The pathways with the least gene count with 2 genes were phenylalanine metabolism, nitrogen metabolism, sulphur metabolism, vitamin B6 metabolism, selenocompound metabolism, ascorbate, and aldarate metabolism. Other enriched KEGG pathways included the citrate cycle, valine leucine, isoleucine, and histidine (S9 Table).

The enriched KEGG pathways in the temephos-resistant differentially expressed proteins showed 43 enriched pathways (S9 Table). Metabolic pathways revealed the highest gene count of 76, followed by carbon metabolism with 32 gene counts, ribosome with 19 gene counts, biosynthesis of amino acid with 16 gene counts, glycolysis with 15 gene counts, citrate cycle and valine, leucine, and isoleucine degradation with 11 gene counts each, fatty acid degradation and pyruvate metabolism with 10 gene counts in each category. Phenylalanine, tyrosine, and tryptophan biosynthesis, synthesis and degradation of ketone bodies, sulphur metabolism, and retinol metabolism revealed 2 gene counts. Other pathways included protein processing in the endoplasmic reticulum, insect hormone biosynthesis, oxidative phosphorylation, phenylalanine metabolism, lysosome and cysteine, and methionine metabolism (S9 Table).

The local cluster in the protein-protein interaction network (PPI) showed 112 and 120 clusters in differentially expressed proteins of permethrin and temephos-resistant strains, respectively. Examples of the local clusters of the differentially expressed proteins were shown in S10 Table. The clusters with the highest gene count in the permethrin and temephos- resistant strains was the mixed, inclusive carbon metabolism and aldehyde dehydrogenase domain with 45 and 43 gene counts, respectively. At the same time, the clusters with the highest strength were the glycolysis and enolase in differentially expressed proteins of permethrin- resistant strain and disulphide isomerase and heat shock protein 70 family clusters in differentially expressed proteins of temephos-resistant strain.

## 4. Discussion

Our study revealed a similar trend to previous studies where both *Ae*. *aegypti* field strains from dengue hotspot and non-hotspot areas showed resistance against permethrin based on mortality rate and resistance ratio (RR) (Table 4). Furthermore, the mortality rates showed more resistance in the hotspot strain than the non-hotspot strain. The differences in the resistance may indicate differences in the underlying resistance mechanism in the field strains. In the previous study, Rong et al. [6] conducted biweekly surveillance of field *Ae*. *aegypti* in two dengue-endemic areas of Shah Alam, Selangor, Malaysia. The field study reported permethrin resistance with an extremely low mortality percentage of less than 10%. Also, they showed that laboratory permethrin-selected strains of *Aedes* species exhibited an increasing resistant development pattern of permethrin throughout three generations of Aedes mosquitoes.

On the other hand, recent studies conducted in dengue hotspot areas of Selangor emphasised possible risks to the success of insecticides presently implemented in dengue vector control. Rasli et al. [18] studied the largest dengue hotspot areas, with about 24 different dengue hotspot areas. They showed high and significant insecticide-resistant to pyrethroid and organophosphate. In Penang Island, Zuharah et al. [19] reported the risk of developing insecticide resistance towards pyrethroid with around 87% mortality rates towards permethrin in the Northern district of Penang. Rasli et al. [18] and Zuharah et al. [19] indicated insecticide resistance in *Ae*. *aegypti* in the dengue hotspot areas similar to the current study.

The larval bioassay from the field strains showed both dengue hotspot and non-hotspot *Ae*. *aegypti* strains indicated resistance against temephos based on mortality rates and resistance ratio (RR). On the other hand, a higher concentration of temephos was required to attain 100% mortality, which was indicated by discriminating concentrations of 1.086mg/l and 0.810mg/l in the hotspot and non-hotspot field strains (Table 4). These discriminating concentrations in the field *Ae*. *aegypti* exceeded the established concentration of 0.012mg/l temephos required to eliminate larval stages [22,30]. The development of temephos resistance in *Ae*. *aegypti* larvae have been reported in Malaysia since the late 1980s [4]. Ishak et al. [8] showed moderate resistance to temephos in Kuala Lumpur, Johor Bharu, and Kota Bharu. Chen et al. [3,4] reported high temephos resistance status *Ae*. *aegypti* field strains from Kuala Lumpur and Selangor.

The resistance development in permethrin and temephos could be attributed to selection criteria due to the continuing use of insecticides to control dengue vectors. The insecticide use is sometimes extensive, where vector control officers performed about twenty circles of thermal fogging in the dengue-endemic area of Shah Alam in five months [6]. The resistance in the non-hotspot could be due to the area being once a dengue hotspot. These areas received attention from vector control management and private contractors, which led to insecticide selection pressure in Aedes mosquitoes and produced offspring carrying insecticide-resistant genes [31].

This study utilised LC–ESI–MS/MS and a label-free quantitative proteomics approach for protein identification and quantification, known as tandem mass spectrometry (MS). This system is the electrospray ionisation (ESI) coupled to a quadrupole-orbitrap MS for a parallel accumulation of a serial fragmentation acquisition via collision-induced and high-energy collision-induced dissociation. This model of tandem MS enhanced the speed and sensitivity by increasing overall mass-ion intensity detection and resolution for better proteome coverage and quantification [32]. The advantage of the orbitrap analyser is due to its high-resolution power. The high resolution has given an edge to the orbitrap in analysing proteins characterised by high molecular weight [33].

Furthermore, the label-free quantitative proteomics (LFQ) approach was used to identify and quantify differentially expressed proteins in the insecticide-resistant *Ae*. *aegypti*. The advantage of using the LFQ approach is that it does not require tedious sample preparation, while the labelled approach requires additional pre-treatments and expensive labelling reagents. Thus, the LFQ approach is less likely than labelled techniques to cause errors in sample preparations. This approach can be applied to nearly all numbers and types of samples and enable comparison across many experimental conditions [25], allowing greater study design flexibility.

In contrast, isotopic labelling approaches are limited to the number of samples or experiments that may be directly evaluated [34]. Therefore, we studied the differentially expressed proteins in permethrin and temephos-resistant *Ae*. *aegypti* based on the advantages of the LFQ and the tandem mass spectrometry LC–ESI–MS/MS.

Previously, we evaluated the proteome of adult female *Ae*. *aegypti* using two protein extraction methods via LC-ESI-MS/MS indicated that CytoBuster protein extraction reagent was superior to TCA in terms of the protein yield, proteome coverage and extraction speed [24]. Furthermore, TCA acetone, precipitation extraction methods performed better in larvae than adult proteins [26].

To elucidate the proteome changes in insecticide resistance Aedes mosquitoes, we compared the proteome of *Ae*. *aegypti* field permethrin- and temephos-resistant with laboratory strains. Using the statistical PERSEUS software, the MS data showed normal distribution after each group’s logarithmic transformation of the peptide intensities (Fig 1A and 1B). The correlation coefficient also showed a high relationship in the data from groups that received the same treatment (Fig 2A and 2B). No outlier was observed from the hierarchical clustering data from the heatmap. The above information showed that the MS data is suitable for the subsequent analysis’s accuracy [27,35].

Differential protein expression analysis from ANOVA revealed 501 differentially expressed proteins from the adult *Ae*. *aegypti* and 557 differentially expressed proteins in larval *Ae*. *aegypti*. The differentially expressed proteins identified in this study were similar to those identified in the preliminary proteomics studies of permethrin-resistant and susceptible *Ae*. *aegypti* by Rosilawati et al. [36] in Malaysia. The proteins such as HSP70, phosphoglucomutase, ribosomal proteins and ferritin.

This study also showed proteins like fumarylacetoacetate, fumarylacetoacetate hydrolase, tubulin beta chain, apyrase, ferritin and D7 similar to those studies by Epelboin et al. in Latin America [16] and Wang et al. [25] in Shandon Province, China, on the pyrethroids resistance *Ae*. *aegypti* and *Cx*. *pipiens pallens*. Furthermore, Zhang et al. [37] revealed the differentially expressed proteins in insecticide resistance *Cx*. *pipiens pallens coquillett* in Shandong Province, China, similar to differentially expressed proteins found in our study, i.e., cytochromes-related, motor-related, ribosomal, GST, cuticle, and HSPs (S1 File).

There were 26 shared proteins between this study and Epelboin et al. [16]. We found CYP450 and GSTs families’ proteins among differentially expressed proteins. However, none of these proteins was observed in Epelboin et al. [16] study. The differences in the identified proteins of our studies to those of Epelboin et al. [16] may be due to the type of samples used, where Epelboin et al. focused on mid-gut tissue compared to the total whole-body proteins in this study.

This study identified cytochrome-related proteins among differentially expressed proteins, and this finding relates to that permethrin Fig A in S1 File and temephos Fig B in S1 File resistant *Ae*. *aegypti* required additional ATP to accomplish different cellular functions, including respiration and activation of detoxification systems [38].

The t-test of this study also revealed the upregulation of cytochrome c oxidase subunit VIA and cytochrome c oxidase subunit 4 with 3.84- and 2.46-fold changes (Table 5). Also, Rosilawati et al. [36] also found these two cytochromes in the Malaysia permethrin resistant-Aedes mosquitoes.

Cytochrome b 5 (Cytb5) is an essential enzyme in CYP450-mediated metabolism because it acts as the second electron donor or required cofactor [39]. Kasai et al. [40] have reported the overexpression of cytb5 more than 3-fold in field-collected and laboratory-selected permethrin-resistant strains *Ae*. *aegypti*, after ten generations of adult selection. Furthermore, the overexpression with a 1.5-fold change of cytb5 in permethrin-resistant strain was confirmed by Sun et al. [17] using RNA sequence and proteomics-based approaches. The cytb5 expression in *Ae*. *aegypti* permethrin and temephos-resistant strains in this study showed a possible association between CYP450 enzymes and increased insecticide detoxification in mosquitoes as shown in Fig A-B in S1 File.

This study also identified differentially expressed NADPH cytochrome P450 reductase in permethrin and temephos-resistant *Ae*. *aegypti*. The NADPH cytochrome P450 reductase is also an electron transfer protein found in the endoplasmic reticulum of cells. NADPH cytochrome P450 reductase and cytb5 have been co-expressed in some instances [41]. Marcombe et al. [42] have reported the overexpression of NADPH cytochrome P450 reductase in insecticide-resistant *Ae*. *aegypti* population in Martinique. They used qPCR to investigate the transcription levels of the NADPH cytochrome P450 reductase.

This study identified CYP9J9 and CYP9J27 differentially expressed proteins in permethrin-resistant strain, in concordance with Bariami et al. [12], who demonstrated the differential expression of CYP9J9 and CYP9J27 in pyrethroid-resistant *Ae*. *aegypti* from the Cayman Islands and Cuba. Furthermore, a transcriptome sequencing analysis indicated the significant-high transcript levels of the CYP9J family in pyrethroid-resistant versus susceptible *Ae*. *aegypti* in Vietnam [43]. We also observed that CYP9J27 and CYP9J9 were the only differentially expressed proteins of the larvae temephos-resistant strain. Therefore, we postulate the expression of CYP450 family proteins, mainly CYP9J27 and CYP9J9 may relate to the detoxification of organophosphate-based insecticides.

Differentially expressed glutathione transferase enzymes (GST) in permethrin Fig C and temephos Fig D in S1 File resistant adult and larvae *Ae*. *aegypti* identified in this study using ANOVA included GSTD1 (J9HHL7) and GSTD1 (A0A1S4EXN8). Other exclusively determined GSTs in permethrin-resistant adults were GST (A0A6I8TLA6), GST(A0A6I8TL57), GSTe4 (Q5PY78), and glutathione synthetase (Q0IEN8) (Table 5). In the temephos-resistant larvae, the unique GSTs were GST (Q16P79), glutathione peroxidase (A0A6I8TLR0), S-(hydroxymethyl) glutathione dehydrogenase (Q176A6), GST1 (Q17MB8), GSTd4 (Q0C791), and GSTD1-1 (Q16SH7). The t-test analyses of the permethrin-resistant strain versus the laboratory strain exposed to permethrin also showed the upregulation of GST (J9HHL7) with a 3.02-fold change (Table 5).

GSTs belong to the detoxifying enzyme systems in *Ae*. *aegypti*. These enzymes transform xenobiotic compounds into harmless ones via increased expression or gene alteration and substitution [44]. Hamzah & Alias, [45] examined GST expression in Aedes mosquitoes using affinity chromatography and proteomics analysis. Their study compared susceptible strain, field strain, and laboratory permethrin-selected strain. They confirmed the overexpression of sixteen isoforms GSTS1-1, GSTS1-4, GSTS1-3, GSTS1-4, GSTD1-3, GSTD1-2, GSTD1-5, GSTD1-6, GSTD1-7, GSTD1-8, GSTD1-9, GSTD1-10, GSTD1-11, GSTT, GSTD1-12, and GSTD1-13 in the field strain (p<0.05) than the susceptible strain. This study identified two differentially expressed GSTD-1s in the permethrin-resistant *Ae*. *aegypti*, included J9HHL7 and A0A1S4EXN8 isoforms. The differential expression of GSTD-1 in permethrin-resistant *Ae*. *aegypti* supporting that GST delta class might play a role in the glutathione S-transferase detoxification part of the metabolic resistance mechanism against permethrin.

Our study showed GSTD11 and GSTD4 were also differentially expressed in the temephos-resistant larvae. The differentially expressed GSTs in this study indicated that metabolic resistance mechanisms might trigger temephos despite permethrin resistance in Aedes mosquitoes.

Previously, many gene amplification products like myosin were expressed in insecticide-resistant mosquitoes [46]. The identification of differentially expressed proteins of motor-related proteins Fig E-F in S1 File like myosin heavy chain, non-muscle, or smooth muscle (Q179E8), (Q17L97), myosin heavy chain (W0FUL2), myosin regulatory light chain 2 (Q17HX1), myosin light chain alkali (Q16MS5), and myosin light chain kinase (Q17AF2) suggested that increased muscle contraction in the permethrin resistant *Ae*. *aegypti* was likely associated with resistant development (Table 5). Furthermore, the t-test revealed the downregulation of myosin heavy chain (W0FUL2) and troponin I (Q16RS3) (Table 5). In the temephos-resistant larvae Fig F in S1 File, proteins such as troponin t, invertebrate (A0A6I8T582), troponin I (Q16RS5), paramyosin, long-form (Q16RF4), myosin regulatory light chain 2 (mlc-2) (Q17HX1), paramyosin (A0A6I8TKE6), myosin light chain alkali (Q16MS5) were differentially expressed in this study (Table 6).

Myosin is a superfamily group of enzymes and contractile proteins known as motor proteins. This motor protein is actin-dependent and accelerates contraction in muscles and cell division [47,48]. Previously Yang et al. [49] have reported the involvement of the myosin regulatory light chain (MRLC) gene in the deltamethrin-resistant *Cx*. *pipiens pallens*. To unravel if the myosin regulatory light chain was involved in the deltamethrin resistance, Yang et al. [49] cloned the full-length MRLC from *Cx*. *pipiens pallens* into a mosquito expression plasmid DNA pIB/V5-His-TOPO MRLC. Then, the recombinant plasmid was transfected into *Ae*. *albopictus* C6/C3 cell lines. The expression was evaluated with the control plasmid transfected cells to observe their proliferation with deltamethrin treatments. The RT-qPCR analysis demonstrated that the MRLC transcription level in the deltamethrin-resistant strain was 4.08-fold higher than in the deltamethrin susceptible strain. The outcome indicated MRLC as a possible trigger of deltamethrin resistance in *Cx*. *pipiens pallens* [49]. Therefore, the differential expression of myosin regulatory light chain, myosin regulatory light chain 2, myosin light chain alkali, and kinase in resistant adult and larvae *Ae*. *aegypti* in this study suggests these proteins’ potential involvement in regulating permethrin and temephos resistance in *Ae*. *aegypti* in Penang Island.

The mosquito cuticle, also known as the exoskeleton, is the outermost part of the insect body. The functions of insect cuticles involve the sensory perception of the environment, locomotion, physical structure maintenance, and shielding from desiccation. The cuticle is the foremost and significant obstacle against exterior harmful chemical penetration. Thus, the cuticle is an effective insecticide penetration channel in insects. Insect cuticles are composed of cuticular protein, chitin, and lipids. The chitin sclerotisation, structure, and hydration manipulate the insect cuticle’s properties, including permeability to pyrethroids [50].

Furthermore, cuticle proteins and their assembly pattern influenced the integrity of the cuticle [37,51]. This study identified several insect cuticle proteins differentially expressed in ANOVA and t-test analyses, including pupal cuticle protein, putative (A0A1S4FZ80), cuticle protein 2 (Q17LN8), cuticle protein 5 (Q16I56) in the permethrin and temephos resistant adults and larvae *Ae*. *aegypti* Fig G-H in S1 File. The downregulation of pupal cuticle protein 78E, putative of the permethrin-resistant strain revealed by the t-test with a fold change of 2.86 (Table 5). Wang et al. [25] also showed the downregulation of pupal cuticle protein 78E, putative in *Cx*. *pipiens pallens* in pyrethroid-resistant compared to susceptible strain. Furthermore, Fang et al. [52] indicated that cuticle proteins identified by Wang et al. [25] were associated with deltamethrin resistance in *Culex* mosquitoes. Fang et al. [52] used RT-qPCR to measure the expression levels of the cuticle proteins from deltamethrin-resistant and laboratory strains after short-term exposure to the insecticide. The cDNA of the CpCPLCG5 gene that encodes the cuticle proteins was cloned from *Cx*. *pipiens pallens*. The outcome indicated higher expression of the CpCPLCG5 gene level in the deltamethrin-resistant strain.

In the temephos-resistant larvae Fig H in S1 File, we identified pupal cuticle protein 78E, putative (Q0IGD5), pupal cuticle protein 36 (Q17G24), pupal cuticle protein, putative (Q17FX9), cuticle protein, putative (Q16E70, Q16UU3, Q16UU5, Q16EH6, Q16UU4), cuticle protein (Q17KI1), larval cuticle protein lcp-30 isoform x1 (Q17G21), and larval/pupal cuticle protein h1c (Q17MT9). The differential expression of cuticle proteins in both adult and larval *Ae*. *aegypti* in permethrin and temephos-resistant strains might suggest the reduction of pyrethroids and temephos penetration in the mosquito body was due to cuticle thickening and alteration of cuticle composition. However, the notion that permethrin and temephos involvement in cuticle remodelling reduces insecticide penetration into the insects’ nervous systems is not fully understood. It needs further study to elucidate the complicated mechanism.

Ribosomal proteins are known for maintaining cell growth and death and their functions in protein biosynthesis and translation [37]. We observed a range of ribosomal proteins was differentially expressed, revealed by ANOVA Fig L-J in S1 File and t-test analyses in the permethrin- (Table 5) and temephos- (Table 6) resistant *Ae*. *aegypti* of this study. Ribosomal protein (Q16JT5) and 60S ribosomal protein L6 (Q16ZH3) were the proteins with Fold change of 5 in temephos-resistant larvae compared to the laboratory strains exposed to temephos (Table 6).

The differential expressions of these ribosomal protein’s changes in protein biosynthesis and translation of mRNA into protein during the insecticide pressure in *Ae*. *aegypti* [37]. Previously, Zhang et al. [37] reported several ribosomal proteins differentially expressed in insecticide resistance *Cx*. *pipens pallens*. Several ribosomal proteins identified in Zhang et al. [37] studies correspond to the ones identified in this study. These include acidic ribosomal protein P1, ribosomal protein L28, 60S ribosomal protein L10, and 60S ribosomal protein L37. This expression of ribosomal proteins in this study highlights the possible involvement of these proteins in conferring permethrin and temephos resistance in *Ae*. *aegypti*.

Heat shock proteins (HSP) are expressed in varying degrees in response to stress and are a highly conserved superfamily of proteins. They are essentially present in all organisms, from prokaryotes to eukaryotes [53]. This study identified four heat shock differentially expressed proteins Fig K in S1 File, including heat shock cognate 70 (Q1HQZ5), in the adult *Ae*. *aegypti* permethrin-resistant strain, heat shock cognate 70 (Q1HR69), heat shock cognate 70 (Q1HQZ5), and HSP 83 (Q16KZ2) in the larvae temephos-resistant strain Fig L in S1 File. The t-test analysis showed the heat shock cognate (Q1HQZ5) was upregulated (Table 6). HSP identification in this study corresponded to the preliminary proteomic analysis of permethrin-resistant and susceptible *Ae*. *aegypti* in Malaysia by Rosilawati et al. [36]. Rosilawati et al. [36] reported that HSP70 was among the essential proteins associated with protecting and defence against permethrin. Djegbe et al. [54] have identified HSP83 significantly overexpressed in the salivary glands of acetylcholinesterase (ace-1^R^ allele) resistant *Culex* mosquitoes. They focused on a quantitative comparison of salivary proteins from two strains of *Cx*. *quninquefasciatus* with the same genetic makeup but carrying either an ace-1^R^ allele or not. The identification of HSPs in this study suggests their function to protect and enhance the survival chances of the *Ae*. *aegypti* against permethrin and temephos insecticides.

Among the upregulated proteins identified in permethrin resistance compared to laboratory strains exposed to permethrin, proteins with the highest fold change (FC) included Fumarylacetoacetase (Q16NG1), Catalase (Q16J86), Aliphatic nitrilase, putative (Q16TC0), Fumarylacetoacetate hydrolase (Q17N03), with FC>6 (Table 5). The upregulation of catalase (Q16J86) with FC 7.7 is vital in permethrin-resistant *Ae*. *aegypti*. Catalase has been identified as a metabolic gene associated with pyrethroid-resistant [46,55]. It is an antioxidant enzyme that facilitates the catalysis of H_2_O_2_ into H_2_O and oxygen (O_2_) [56]. *Ae*. *aegypti* exposure to permethrin can trigger the production of reactive oxygen species such as hydrogen peroxide (H_2_O_2_) in the mitochondria. Reactive oxygen species are deadly to living cells, leading to oxidative damage in proteins, lipids, and DNA. Thus, reactive oxygen species are associated with longevity. Other upregulated antioxidants in this category include GST (J9HHL7).

Fisher’s exact test identified most of the differentially expressed proteins enrichment in a cellular component in the permethrin-resistant *Ae*. *aegypti* to be associated with oxidative phosphorylation, oxidoreductase activity electron transport, and ATP synthase proteins (Table 7). Those proteins were involved in the protein complexes driving the electron transport chain and ATP generation. This study highlighted sodium/potassium-transporting ATPase subunit alpha and sodium/potassium-dependent ATPase beta-2 subunit. Zhang et al. [37] have reported the expression of the above two proteins in pyrethroid-resistant *Culex* mosquitoes. Voltage-dependent anion channel VDAC increases the permeability and metabolite passage between the mitochondria and the cytosol [31]. This study showed a significant expression of VDAC (Q1HR57) might be associated with pyrethroid passage because VDAC is a vital gatekeeper. The GO term molecular function of VDAC reveals that VDAC facilitates energy-dependent transportation of toxins throughout the membrane outside of the cell. Further research is required to disclose the potential involvement of the VDAC gene in pumping out pyrethroid outside the cell.

In this study, enoyl-CoA hydratase and acyl-coenzyme A oxidase functioned in the fatty acid metabolism, lipase involved in phospholipase activity, and AAEL012645-PA, chitin-binding proteins were the only significant proteins identified by Fisher exact test in the temephos- resistant *Ae*. *aegypti* larvae. The functions of these proteins need to be further elucidated as no reports about these proteins in mosquitoes’ insecticide-resistant studies.

The heterogeneity of the differentially expressed proteins linked to multiple resistance in the field *Ae*. *aegypti* strains were observed in various studies [16,17]. This study also found differentially expressed proteins related to metabolic and cuticle resistance, like cytochrome B and cuticle proteins. Therefore, functional studies are required to understand further the roles of the differentially expressed proteins related to multiple resistance mechanisms in providing additional information which may help manage insecticide resistance in Aedes mosquitoes.

The validation of differentially expressed proteins in this study focused on proteins that were not previously validated in permethrin/temephos-resistant *Ae*. *aegypti*. Based on the available reports, this is the first study in Malaysia to validate sodium/potassium-dependent ATPase β2 subunit, enolase phosphatase E1, glucosidase 2 β and troponin I in permethrin -resistant *Ae*. *aegypti*, H15 domain-containing protein, 60S ribosomal protein L21, AAEL01756-PB, and tubulin β chain in temephos-resistant *Ae*. *aegypti*, though some of the differentially expressed proteins were previously reported in permethrin-resistant *Ae*. *aegypti*, like the troponin and sodium/potassium-dependent ATPase β2 subunit (S7 Table).

The qPCR gene expression confirmed the protein expression analyses by revealing the upregulation of sodium/potassium-dependent ATPase β2 in *Ae*. *aegypti* permethrin-resistant strain from the non-hotspot area (Table 8). The upregulation of 60S ribosomal protein L21 in the temephos-resistant strain of the hotspot and the non-hotspot regions suggests this protein involvement in regulating insecticide resistance in *Ae*. *aegypti* field strain compared to the laboratory strain (Table 8).

The membrane protein sodium/potassium-dependent ATPase β2 maintains the electrochemical gradient essential for the normal functioning of cells by the active transport of sodium and potassium ions and membrane potentials [57]. The importance of the pump created by the electrochemical gradient of sodium/potassium ions is the transport of organic and inorganic molecules from and within the cells, including excitable and in the nervous system [57]. This action may be related to the action potentials created by sodium-gated channels with electrical signalling functions in excitable cells [57]. In insects, sodium/potassium ATPase targets several xenobiotic compounds [57]. This condition might explain the upregulation of sodium/potassium-dependent ATPase β 2 in permethrin-resistant *Ae*. *aegypti* strain. The validation of H15 domain-containing protein and 60S ribosomal protein L21 in the temephos-resistant *Ae*. *aegypti* and AAEL01756-PB, a structural constituent of ribosomes in temephos-resistant *Ae*. *aegypti* also suggest associations of genes in the insecticide resistance mechanism, although their function did not correlate to enzymes associated with detoxification systems.

Troponin is part of the motor-related proteins responsible for muscle contraction and maintains the nervous system [58]. Also, Troponin is involved in the cytoskeleton apparatus. The downregulation of troponin I in permethrin-resistant *Ae*. *aegypti* might hint increase muscle activity. After all, permethrin exerts its function through nerve conduction blocking and muscle paralysis [59–61], countering the immediate action of troponin to contract muscle cells.

The outcome of the gene expression may highlight the potential use of the differentially expressed proteins as biomarkers for monitoring and predicting insecticide resistance in field populations of *Ae*. *aegypti* as the analyses used two conditions of field strains: exposed to (resistant) and not exposed to the insecticides.

Overall, the high PPI network <1.0 x 10^−16^ of the differentially expressed proteins in permethrin (Fig 7) temephos (Fig 8) resistant *Ae*. *aegypti* indicated that the interactions among the proteins were at least partially biologically connected as an entity. The significant PPI in the permethrin- and temephos-resistant differentially expressed proteins strongly suggests that the interaction is likely due to the oxidative stress of permethrin and temephos exposure. This is because several oxidative stress-producing proteins were differentially expressed, for example, the GSTs, catalase, H_2_O_2_ and thioredoxin.

The PPI network showed the highest interaction in the biological process ontology associated with probable citrate synthase 1 and VhaA in the metabolic drug process with high strength interaction of 1.12 and FDR of <0.05. Probable citrate synthase 1 (AAEL002956) has been enriched in the metabolic drug process, small molecule metabolic process, and catalytic activity. V-type proton ATPase catalytic subunit A (VhaA) (AAEL008787-PA) were enriched in all ontology categories except hydrolase activity acting on ester bonds. VhaA is an essential electrogenic pump with ATPase activity. It can transport protons from the cytoplasm to the extracellular fluid, thereby acidifying organelles to generate negative cell membrane voltages. The membrane voltage drives ion transport through specific ion channels, creating electrochemical proton potential. In addition, the electrochemical proton potential can facilitate secondary active transportation such as cation/H+ exchange or anion/H+ co-transport [62,63]. The active participation of VhaA proteins in all the enriched ontologies suggests the proteins’ versatility in the metabolic and small molecule metabolic processes (Table 9).

Enolase phosphatase E1 was enriched in all the identified biological process ontologies except in the nucleobase-containing small molecule metabolic process category and molecular function ontologies in permethrin-resistant differentially expressed proteins. Enolase phosphatase E1 was also enriched in several of the enriched functional ontologies in the temephos-resistant differentially expressed proteins like metabolic process, primary metabolic process, cellular metabolic process and organonitrogen compound metabolic process (S8 Table). The enrichment of enolase phosphatase E1 in the metabolic drug process and small molecule metabolic process validates by the student’s t-test and the gene expression analysis in this study. Enolase phosphatase E1 is a bifunctional enzyme catalyses 2,3-diketo-5-methylthiopentyl-1-phosphate enolization to form a transitional 2-hydroxy-3-keto-5-methylthiopentenyl-1-phosphate necessary to produce acireductone 1,2-dihydroxy-3-keto-5-methylthiopentene. Enolase phosphatase E1 is essential for the methionine salvage pathway, where methylthioadenosine is converted into L-methionine [64]. The methionine salvage pathway plays a critical role in stress responses by increasing the levels of polyamine protein, which is essential for normal cell function and growth [64]. The PPI of enolase phosphatase E1 interacted with chaperonin (AAEL011584), an HSP60 family, and electron transport oxidoreductase (AAEL013739). We postulate its interaction with HSP60 may be linked to conferring insecticide resistance [36,53].

Phophotriesterases have been associated with insecticide resistance in insects [65]. Phophotriesterase carries the esterase gene, a hydrolase enzyme. The enzymes function in hydrolysing compounds with ester bonds, splitting the ester bonds into acid and alcohol in H_2_O [66]. The enrichment of phosphotriesterases in the metabolic process of the temephos-resistant differentially expressed proteins (S8 Table) suggests the involvement of this protein in sequestering organophosphate compounds or direct hydrolysis of the insecticide leading to metabolic insecticide resistance [67]. Most of the proteins mentioned in the metabolic process are also involved in other biological process categories like organonitrogen compound metabolic process, organonitrogen compound biosynthetic process, small molecule metabolic process, cellular nitrogen compound metabolic process, etc.

The KEGG pathways annotation of the permethrin and temephos-resistant *Ae*. *aegypti* differentially expressed proteins (S9 Table) revealed several enriched pathways that might be involved in insecticide resistance. The number of genes/proteins involved in the metabolic process might indicate the active participation of those proteins in the permethrin and temephos metabolism resulting in insecticide resistance. Oxidative phosphorylation pathway in the permethrin-resistant *Ae*. *aegypti* suggests the energy requirement need to drive the metabolic drug process and other cellular activities. Zhang et al. [37] also showed that oxidative phosphorylation was activated in the differentially expressed proteins of pyrethroid-resistant *Cx*. *pipiens pallens* compared to the susceptible strains. They concluded this through annotated comparison of the KEGG pathways via functional ontology analysis.

The STRING analyses revealed the glycolysis and enolase clusters (CL:7698) with the highest strength of 1.66, followed by CF [1] ATPase, oligomycin sensitivity conferral protein/delta subunit cluster (CL:8954) with 1.65 strength enrichment in the permethrin resistant strain differentially expressed proteins. The glycolysis and enolase clusters involved five proteins such as enolase (AAEL001668-PA), phosphoglycerate kinase (AAEL004988-PA), Triosephosphate isomerase (AAEL002542-PA), fructose-bisphosphate aldolase (AAEL005766), and AAEL016984-PA (S9 Table). Although no available study associates the above proteins with permethrin resistance, Chowdhury et al. [68] have reported the upregulation of enolase, phosphoglycerate kinase, and fructose-bisphosphate aldolase in the salivary glands of *Ae*. *aegypti* infected with dengue virus related to metabolic proteins. The glycolysis and enolase clusters in the temephos-resistant differentially expressed proteins also revealed the highest enrichment strength of 1.65 (S9 Table). The enrichment of glycolysis and enolase clusters in permethrin- and temephos- resistant differentially expressed proteins might suggest their involvement in regulating pyrethroid and organophosphate resistance. However, further functional study is required to understand the association of the above proteins in insecticide resistance *Ae*. *aegypti*.

CF ATPase, oligomycin sensitivity conferral protein/delta subunit cluster was associated with four proteins, including mitochondrial ATP synthase b chain (AAEL005610-PA), ATP synthase gamma subunit (AAEL008848-PA), ATP synthase delta chain, and ATP synthase subunit alpha (S10 Table). Previous ATP synthase proteins have been reported by Wang et al. [25] and Zhang et al. [37] to be present in pyrethroid-resistant mosquitoes. Zhang et al. [37] have validated the presence of ATP synthase delta chain in insecticide-resistant *Culex* using parallel reaction monitoring. Meanwhile, the disulphide isomerase and heat shock protein 70 family cluster (CL:3606) had the highest enrichment strength of 1.72 in the temephos-resistant differentially expressed proteins. The proteins were AAEL017349-PA, protein disulphide isomerase (AAEL00641-PA), protein disulphide isomerase A6 precursor (AAEL010065-PA), endoplasmin (AAEL012827-PA) and AAEl017263-PA. The result concurs with the previous report, where HSP and endoplasmin proteins are associated and differentially expressed in insecticide-resistant mosquitoes [54].

Other important clusters in permethrin and temephos-resistant differentially expressed proteins were the peroxidase and thioredoxin/glutathione reductase selenoprotein clusters (CL:8413) with 1.34 and 1.32 strengths, respectively. This cluster comprised thioredoxin and catalase, suggesting the regulation of oxidative stress created by the insecticide. The two proteins function as antioxidants in the cellular process. Epelboin et al. [16] also reported some antioxidants in the pyrethroid-resistant *Ae*. *aegypti*, like superoxide dismutase and catalase, suggested their interaction with other antioxidants like GST. The mixed, inclusive glutathione metabolism and AhpC/TSA (alkyl hydroperoxide reductase/thiol specific antioxidant) clusters were also enriched in permethrin, and temephos-resistant differentially expressed proteins. These clusters might suggest a survival strategy by the *Ae*. *aegypti* against permethrin and temephos insecticides. This cluster also shared antioxidant action with peroxidase and thioredoxin/glutathione reductase selenoprotein clusters (CL:8413). Antioxidants were reported to be associated with insecticide-resistant mosquitoes previously [16,37].

In conclusion, this study has provided new insight and supplemented data *Ae*. *aegypti* permethrin and temephos resistance mosquitoes are based mainly on the differential protein expression and predictive functions of the differentially expressed proteins in Malaysia. The validated differentially expressed proteins merit further investigation to be used as biomarkers for prediction and monitoring insecticide resistance *Ae*. *aegypti* mosquitoes in the field.

## Supporting information

S1 Table Additional bioassay data. (DOCX)

S2 Table Permethrin resistant DEPs. (XLSX)

S3 Table Temephos resistant DEPss. (XLSX)

S4 Table Set of DEPs. (XLSX)

S5 Table T test analyses for permethrin. (XLSX)

S6 Table T test for temephos. (XLSX)

S7 Table Average C_T_ values. (DOCX)

S8 Table Functional ontology enrichment. (DOCX)

S9 Table Enriched KEGG pathways. (XLSX)

S10 Table Clusters in Permethrin and Temephos. (XLSX)

S1 File Figure A-L. (DOCX)
