# Supplementary material for: Quantitative proteomics analysis of permethrin and temephos-resistant Ae. aegypti revealed diverse differentially expressed proteins associated with insecticide resistance from Penang Island, Malaysia
Source: PLoS Negl Trop Dis. 2023 Sep 18;17(9):e0011604. doi: 10.1371/journal.pntd.0011604 (PMC10538732; doi:10.1371/journal.pntd.0011604)
Supplement: S1 Table — (DOCX) [file pntd.0011604.s001.docx]

**Supplementary S1 (a) Adult bioassay of the hotspot strain against permethrin.**

| **Time/Rep** | **1** | **2** | **3** | **4** | **5** | **Control** | **Control** | **Knockdown** | **SD** |
| --- | --- | --- | --- | --- | --- | --- | --- | --- | --- |
| 0 | 00 | 00 | 00 | 00 | 00 | 00 | 00 | 00 | 0.00 |
| **05 mins** | **01** | **01** | **02** | **02** | **01** | **00** | **00** | **07** | **0.54** |
| 10 mins | 01 | 02 | 02 | 03 | 02 | 00 | 00 | 10 | 0.70 |
| 15 mins | 05 | 04 | 03 | 03 | 02 | 00 | 00 | 17 | 1.14 |
| 20 mins | 05 | 05 | 04 | 04 | 02 | 00 | 00 | 20 | 1.22 |
| 25 mins | 05 | 06 | 06 | 04 | 02 | 00 | 00 | 23 | 1.67 |
| 30 mins | 05 | 06 | 06 | 04 | 02 | 00 | 00 | 23 | 1.67 |
| 35 mins | 05 | 06 | 06 | 04 | 02 | 00 | 00 | 23 | 1.67 |
| 40 mins | 06 | 06 | 06 | 04 | 02 | 00 | 00 | 24 | 1.78 |
| 45 mins | 06 | 06 | 06 | 04 | 02 | 00 | 00 | 24 | 1.78 |
| 50 mins | 06 | 07 | 07 | 04 | 03 | 00 | 00 | 27 | 1.81 |
| 55 mins | 06 | 07 | 07 | 04 | 03 | 00 | 00 | 27 | 1.81 |
| **60 mins** | **06** | **08** | **07** | **04** | **03** | **00** | **00** | **28** | **2.07** |

**Notes:**

Rep: Replicate - the number of replicates in the experiment. There were 20 mosquitoes in each replicate.

SD: Standard deviation – measures how dispersed the data is in relation to the mean.

**Supplementary S1 (b) Adult bioassay of the non-hotspot strain against permethrin.**

| **Time/Rep** | **1** | **2** | **3** | **4** | **5** | **Control** | **Control** | **Knockdown** | **SD** |
| --- | --- | --- | --- | --- | --- | --- | --- | --- | --- |
| 0 | 00 | 00 | 00 | 00 | 00 | 00 | 00 | 00 | 0.00 |
| **5 mins** | **00** | **00** | **02** | **02** | **02** | **00** | **00** | **06** | **1.09** |
| 10 mins | 00 | 01 | 05 | 06 | 04 | 00 | 00 | 16 | 2.58 |
| 15 mins | 01 | 02 | 08 | 07 | 09 | 00 | 00 | 27 | 3.64 |
| 20 mins | 02 | 03 | 08 | 08 | 10 | 00 | 00 | 31 | 3.49 |
| 25 mins | 03 | 03 | 09 | 09 | 10 | 00 | 00 | 34 | 3.49 |
| 30 mins | 04 | 04 | 10 | 11 | 10 | 00 | 00 | 39 | 3.49 |
| 35 mins | 04 | 05 | 10 | 11 | 10 | 00 | 00 | 40 | 3.24 |
| 40 mins | 05 | 06 | 11 | 12 | 10 | 00 | 00 | 44 | 3.11 |
| 45 mins | 05 | 06 | 12 | 13 | 10 | 00 | 00 | 46 | 3.56 |
| 50 mins | 05 | 07 | 12 | 13 | 11 | 00 | 00 | 48 | 3.43 |
| 55 mins | 06 | 07 | 14 | 14 | 11 | 00 | 00 | 52 | 3.78 |
| **60 mins** | **06** | **08** | **14** | **14** | **11** | **00** | **00** | **53** | **3.57** |

**Supplementary S1 (c) Adult bioassay of the laboratory strain against permethrin**

| **Time/Rep** | **1** | **2** | **3** | **4** | **5** | **Control** | **Control** | **Knockdown** | **SD** |
| --- | --- | --- | --- | --- | --- | --- | --- | --- | --- |
| 0 mins | 00 | 00 | 00 | 00 | 00 | 00 | 00 | 00 | 0.00 |
| **5 mins** | **03** | **04** | **04** | **03** | **04** | **00** | **00** | **18** | **0.54** |
| 10 mins | 14 | 17 | 17 | 14 | 17 | 00 | 00 | 79 | 1.64 |
| 15 mins | 19 | 19 | 20 | 18 | 19 | 00 | 00 | 95 | 0.70 |
| 20 mins | 20 | 20 | 20 | 19 | 20 | 00 | 00 | 99 | 0.44 |
| **25 mins** | **20** | **20** | **20** | **20** | **20** | **00** | **00** | **100** | **0.00** |
| 30 mins | 20 | 20 | 20 | 20 | 20 | 00 | 00 | 100 | 0.00 |
| 35 mins | 20 | 20 | 20 | 20 | 20 | 00 | 00 | 100 | 0.00 |
| 40 mins | 20 | 20 | 20 | 20 | 20 | 00 | 00 | 100 | 0.00 |
| 45 mins | 20 | 20 | 20 | 20 | 20 | 00 | 00 | 100 | 0.00 |
| 50 mins | 20 | 20 | 20 | 20 | 20 | 00 | 00 | 100 | 0.00 |
| 55 mins | 20 | 20 | 20 | 20 | 20 | 00 | 00 | 100 | 0.00 |
| 60 mins | 20 | 20 | 20 | 20 | 20 | 00 | 00 | 100 | 0.00 |

**Supplementary S1 (d) Larval bioassay of the hotspot strain against temephos**

| **Con/Rep** | **1** | **2** | **3** | **4** | **5** | **Control** | **Control** | **Mortality%** | **SD** |
| --- | --- | --- | --- | --- | --- | --- | --- | --- | --- |
| 0.050mg/l | 00 | 00 | 00 | 00 | 00 | 00 | 00 | 00 | 0.00 |
| **0.075mg/l** | **02** | **02** | **01** | **02** | **00** | **00** | **00** | **07** | **0.80** |
| 0.100mg/l | 07 | 06 | 08 | 07 | 03 | 00 | 00 | 31 | 1.72 |
| 0.125mg/l | 09 | 08 | 10 | 08 | 04 | 00 | 00 | 39 | 2.03 |
| 0.150mg/l | 10 | 09 | 11 | 08 | 05 | 00 | 00 | 43 | 2.05 |
| 0.175mg/l | 13 | 13 | 12 | 10 | 09 | 00 | 00 | 57 | 1.62 |
| 0.200mg/l | 16 | 15 | 14 | 13 | 13 | 00 | 00 | 71 | 1.16 |
| **0.250mg/l** | **18** | **17** | **15** | **15** | **15** | **00** | **00** | **80** | **1.26** |

**Notes:**

Con: Concentrations - the concentration of temephos used in the bioassay test.

Rep: Replicate - the number of replicates in the experiment, there were 20 larvae in each replicate.

SD: Standard deviation – measures how dispersed the data is in relation to the mean.

**Supplementary S1 (e) Diagnostic concentration (LC_99_x2) results of the hotspot strain against temephos**

| **Time/Rep** | **1** | **2** | **3** | **4** | **5** | **Control** | **Control** | **Mortality %** | **SD** |
| --- | --- | --- | --- | --- | --- | --- | --- | --- | --- |
| 0 | 00 | 00 | 00 | 00 | 00 | 00 | 00 | 00 | 0.00 |
| **30 mins** | **15** | **14** | **15** | **13** | **15** | **00** | **00** | **72** | **0.80** |
| 40 mins | 19 | 18 | 18 | 19 | 18 | 00 | 00 | 92 | 0.48 |
| **50 mins** | **20** | **20** | **20** | **20** | **20** | **00** | **00** | **100** | **0.00** |
| 60 mins | 20 | 20 | 20 | 20 | 20 | 00 | 00 | 100 | 0.00 |
| 2 hours | 20 | 20 | 20 | 20 | 20 | 00 | 00 | 100 | 0.00 |
| 3 hours | 20 | 20 | 20 | 20 | 20 | 00 | 00 | 100 | 0.00 |
| 6 hours | 20 | 20 | 20 | 20 | 20 | 00 | 00 | 100 | 0.00 |
| 12 hours | 20 | 20 | 20 | 20 | 20 | 00 | 00 | 100 | 0.00 |
| 24 hours | 20 | 20 | 20 | 20 | 20 | 00 | 00 | 100 | 0.00 |
| 36 hours | 20 | 20 | 20 | 20 | 20 | 00 | 00 | 100 | 0.00 |
| 48 hours | 20 | 20 | 20 | 20 | 20 | 00 | 00 | 100 | 0.00 |

**Supplementary S1 (f) Larval bioassay of the non-hotspot strain against temephos.**

| **Con/Rep** | **1** | **2** | **3** | **4** | **5** | **Control** | **Control** | **Mortality %** | **SD** |
| --- | --- | --- | --- | --- | --- | --- | --- | --- | --- |
| **0.050mg/l** | **00** | **01** | **00** | **01** | **01** | **00** | **00** | **03** | **0.48** |
| 0.075mg/l | 01 | 01 | 02 | 02 | 02 | 00 | 00 | 08 | 0.48 |
| 0.100mg/l | 01 | 02 | 03 | 03 | 03 | 00 | 00 | 12 | 0.80 |
| 0.125mg/l | 05 | 04 | 05 | 08 | 07 | 00 | 00 | 29 | 1.46 |
| 0.150mg/l | 09 | 09 | 10 | 11 | 11 | 00 | 00 | 50 | 0.89 |
| 0.175mg/l | 12 | 13 | 15 | 16 | 12 | 00 | 00 | 68 | 1.62 |
| 0.200mg/l | 14 | 15 | 16 | 18 | 15 | 00 | 00 | 78 | 1.35 |
| **0.250mg/l** | **18** | **18** | **18** | **19** | **18** | **00** | **00** | **91** | **0.40** |

**Supplementary S1 (g) Diagnostic concentration (LC_99_x2) results of the non-hotspot strain against temephos.**

| **Time/Rep** | **1** | **2** | **3** | **4** | **5** | **Control** | **Control** | **Mortality %** | **SD** |
| --- | --- | --- | --- | --- | --- | --- | --- | --- | --- |
| 0 | 00 | 00 | 00 | 00 | 00 | 00 | 00 | 00 | 0.00 |
| **30 mins** | **18** | **19** | **18** | **15** | **15** | **00** | **00** | **85** | **1.66** |
| 40 mins | 19 | 20 | 19 | 19 | 19 | 00 | 00 | 96 | 0.40 |
| 50 mins | 19 | 20 | 20 | 20 | 20 | 00 | 00 | 99 | 0.40 |
| **60 mins** | **20** | **20** | **20** | **20** | **20** | **00** | **00** | **100** | **0.00** |
| 2 hours | 20 | 20 | 20 | 20 | 20 | 00 | 00 | 100 | 0.00 |
| 3 hours | 20 | 20 | 20 | 20 | 20 | 00 | 00 | 100 | 0.00 |
| 6 hours | 20 | 20 | 20 | 20 | 20 | 00 | 00 | 100 | 0.00 |
| 12 hours | 20 | 20 | 20 | 20 | 20 | 00 | 00 | 100 | 0.00 |
| 24 hours | 20 | 20 | 20 | 20 | 20 | 00 | 00 | 100 | 0.00 |
| 36 hours | 20 | 20 | 20 | 20 | 20 | 00 | 00 | 100 | 0.00 |
| 48 hours | 20 | 20 | 20 | 20 | 20 | 00 | 00 | 100 | 0.00 |

**Supplementary S1 (h) Larval bioassay of the laboratory strain against temephos.**

| **Con/Rep** | **1** | **2** | **3** | **4** | **5** | **Control** | **Control** | **Mortality %** | **SD** |
| --- | --- | --- | --- | --- | --- | --- | --- | --- | --- |
| **0.004mg/l** | **06** | **06** | **10** | **09** | **11** | **00** | **00** | **42** | **2.05** |
| 0.005mg/l | 09 | 12 | 13 | 14 | 11 | 00 | 00 | 59 | 1.72 |
| 0.0054mg/l | 16 | 16 | 16 | 17 | 17 | 00 | 00 | 82 | 0.48 |
| 0.006mg/l | 19 | 19 | 19 | 18 | 18 | 00 | 00 | 93 | 0.48 |
| 0.007mg/l | 18 | 17 | 19 | 18 | 19 | 00 | 00 | 91 | 0.74 |
| 0.008mg/l | 19 | 18 | 20 | 19 | 20 | 00 | 00 | 96 | 0.74 |
| 0.0085mg/l | 19 | 19 | 20 | 20 | 20 | 00 | 00 | 98 | 0.48 |
| 0.009mg/l | 20 | 20 | 20 | 20 | 19 | 00 | 00 | 99 | 0.40 |
| 0.010mg/l | 20 | 20 | 20 | 19 | 20 | 00 | 00 | 99 | 0.40 |
| **0.011mg/l** | **20** | **20** | **20** | **20** | **20** | **00** | **00** | **100** | **0.00** |

**Supplementary S1 (i) Diagnostic concentration (LC_99_x2) results of the laboratory strain against temephos.**

| **Time/Rep** | **1** | **2** | **3** | **4** | **5** | **Control** | **Control** | **Mortality %** | **SD** |
| --- | --- | --- | --- | --- | --- | --- | --- | --- | --- |
| 0 | 00 | 00 | 00 | 00 | 00 | 00 | 00 | 00 | 0.00 |
| 30 mins | 00 | 00 | 00 | 00 | 00 | 00 | 00 | 00 | 0.00 |
| **40 mins** | **05** | **03** | **04** | **03** | **02** | **00** | **00** | **17** | **1.01** |
| 50 mins | 05 | 03 | 04 | 03 | 02 | 00 | 00 | 17 | 1.01 |
| 60 mins | 06 | 03 | 04 | 03 | 02 | 00 | 00 | 18 | 1.35 |
| 2 hours | 08 | 05 | 06 | 05 | 06 | 00 | 00 | 30 | 1.09 |
| 3 hours | 15 | 10 | 13 | 15 | 11 | 00 | 00 | 64 | 2.03 |
| 6 hours | 15 | 13 | 15 | 15 | 15 | 00 | 00 | 73 | 0.80 |
| 12 hours | 19 | 18 | 18 | 18 | 19 | 00 | 00 | 92 | 0.48 |
| **24 hours** | **20** | **20** | **20** | **20** | **20** | **00** | **00** | **100** | **0.00** |
| 36 hours | 20 | 20 | 20 | 20 | 20 | 00 | 00 | 100 | 0.00 |
| 48 hours | 20 | 20 | 20 | 20 | 20 | 00 | 00 | 100 | 0.00 |
