# Supplementary material for: Quantitative proteomics analysis of permethrin and temephos-resistant Ae. aegypti revealed diverse differentially expressed proteins associated with insecticide resistance from Penang Island, Malaysia
Source: PLoS Negl Trop Dis. 2023 Sep 18;17(9):e0011604. doi: 10.1371/journal.pntd.0011604 (PMC10538732; doi:10.1371/journal.pntd.0011604)
Supplement: S7 Table — (DOCX) [file pntd.0011604.s007.docx]

**S6 Average C_T_ values of the (A). adult *Ae. aegypti* permethrin-resistant from the hotspot, non-hotspot areas and laboratory strain.**

| **Sodium/potassium-dependent ATPase β2 subunit (Q16RY8)** Na^+^/K^+^ ATPase β2 | | |
| --- | --- | --- |
| **Mosquito strain** | **Primer set** | **Average C_T_ ±SD** |
| Laboratory strain | Na^+^/K^+^ ATPase β2 | 30.49 **±** 0.14 |
|  | ACT | 26.27 **±** 0.43 |
|  | RSP17 | 24.51 **±** 0.45 |
| Resistant field strain hotspot | Na^+^/K^+^ ATPase β2 | 34.12 **±** 4.40 |
|  | ACT | 27.76 **±** 0.28 |
|  | RSP17 | 27.97 **±** 0.34 |
| Resistant field strain from non-hotspot | Na^+^/K^+^ ATPase β2 | 27.64 **±** 0.19 |
|  | ACT | 25.20 **±** 0.14 |
|  | RSP17 | 23.70 ± 0.14 |
| **Enolase phosphatase E1(Q17Q32)** | | |
| **Mosquito strain** | **Primer set** | **Average C_T_ ±SD** |
| Laboratory strain | Enolase phosphatase E1 | 26.58 ± 0.34 |
|  | ACT | 28.29 ± 0.55 |
|  | RSP17 | 27.44 ± 1.70 |
| Resistant field strain from hotspot | Enolase phosphatase E1 | 29.75 ± 0.56 |
|  | ACT | 29.37 ± 1.17 |
|  | RSP17 | 30.11 ± 0.21 |
| Resistant field strain from non-hotspot | Enolase phosphatase E1 | 26.85 ± 0.10 |
|  | ACT | 27.15 ± 0.08 |
|  | RSP17 | 25.57 ± 0.18 |
| **Glucosidase 2 β (Q16M80)** | | |
| **Mosquito strain** | **Primer set** | **Average C_T_ ±SD** |
| Laboratory strain | Glucosidase 2 β | 27.63 ± 0.39 |
|  | ACT | 23.11 ± 0.22 |
|  | RSP17 | 23.22 ± 0.68 |
| Resistant field strain from hotspot | Glucosidase 2 β | 36.01 ± 0.59 |
|  | ACT | 29.47 ± 0.33 |
|  | RSP17 | 31.28 ± 2.76 |
| Resistant field strain from non-hotspot | Glucosidase 2 β | 32.65 ± 0.62 |
|  | ACT | 27.25 ± 0.10 |
|  | RSP17 | 25.44 ± 0.19 |
| **Troponin I (Q16RS3)** | | |
| **Mosquito strain** | **Primer set** | **Average C_T_ ±SD** |
| Laboratory strain | Troponin I | 30.45 ± 0.20 |
|  | ACT | 27.74 ± 0.21 |
|  | RSP17 | 26.15 ± 0.23 |
| Resistant field strain from hotspot | Troponin I | 33.94 ± 0.90 |
|  | ACT | 28.52 ± 0.17 |
|  | RSP17 | 29.63 ± 0.29 |
| Resistant field strain from non-hotspot | Troponin I | 30.39 ± 0.25 |
|  | ACT | 26.57 ± 0.35 |
|  | RSP17 | 24.76 ± 0.36 |

**(B). Average C_T_ values of the *Ae. aegypti* larvae temephos-resistant strain from the hotspot, non-hotspot areas and laboratory strain.**

| **H15 domain containing protein(A0A6I8TV54) – H15** | | |
| --- | --- | --- |
| **Mosquito strain** | **Primer set** | **Average C_T_ ±SD** |
| Laboratory strain | H15 | 27.59 ± 0.28 |
|  | ACT | 25.61 ± 0.50 |
|  | RSP17 | 28.68 ± 0.54 |
| Resistant field strain hotspot | H15 | 23.14 ± 0.59 |
|  | ACT | 22.69 ± 0.56 |
|  | RSP17 | 20.90 ± 0.35 |
| Resistant field strain from non-hotspot | H15 | 26.04 ± 2.23 |
|  | ACT | 25.60 ± 0.17 |
|  | RSP17 | 23.19 ± 0.57 |
| **60S ribosomal protein L21(Q1HRN4) 60SRPL21** | | |
| **Mosquito strain** | **Primer set** | **Average C_T_ ±SD** |
| Laboratory strain | 60SRPL21 | 28.50 ± 0.23 |
|  | ACT | 28.73 ± 0.08 |
|  | RSP17 | 28.58 ± 0.13 |
| Resistant field strain from hotspot | 60SRPL21 | 22.28 ± 0.12 |
|  | ACT | 23.79 ± 0.15 |
|  | RSP17 | 22.82 ± 0.65 |
| Resistant field strain from non-hotspot | 60SRPL21 | 24.25 ± 0.08 |
|  | ACT | 27.35 ± 0.11 |
|  | RSP17 | 23.49 ± 0.05 |
| **AAEL01756-PB(J9HFM9) – a structural constituent of ribosome** | | |
| **Mosquito strain** | **Primer set** | **Average C_T_ ±SD** |
| Laboratory strain | AAEL01756-PB | 27.75 ± 0.08 |
|  | ACT | 25.41 ± 0.08 |
|  | RSP17 | 28.23 ± 0.24 |
| Resistant field strain from hotspot | AAEL01756-PB | 24.00 ± 0.15 |
|  | ACT | 23.53 ± 0.32 |
|  | RSP17 | 22.09 ± 0.12 |
| Resistant field strain from non-hotspot | AAEL01756-PB | 24.82 ± 0.04 |
|  | ACT | 27.13 ± 0.58 |
|  | RSP17 | 23.50 ± 0.07 |
| **Tubulin β chain(A0A1S4F2Y4)** | | |
| **Mosquito strain** | **Primer set** | **Average C_T_ ±SD** |
| Laboratory strain | Tubulin β chain | 29.47 ± 0.38 |
|  | ACT | 26.32 ± 0.19 |
|  | RSP17 | 27.44 ± 0.43 |
| Resistant field strain from hotspot | Tubulin β chain | 33.89 ± 0.27 |
|  | ACT | 26.55 ± 0.38 |
|  | RSP17 | 21.67 ± 0.17 |
| Resistant field strain from non-hotspot | Tubulin β chain | 30.62 ± 0.26 |
|  | ACT | 26.41 ± 0.23 |
|  | RSP17 | 22.96 ± 0.30 |
