# Supplementary material for: Quantitative proteomics analysis of permethrin and temephos-resistant Ae. aegypti revealed diverse differentially expressed proteins associated with insecticide resistance from Penang Island, Malaysia
Source: PLoS Negl Trop Dis. 2023 Sep 18;17(9):e0011604. doi: 10.1371/journal.pntd.0011604 (PMC10538732; doi:10.1371/journal.pntd.0011604)
Supplement: S8 Table — (DOCX) [file pntd.0011604.s008.docx]

**S7.** **Functional ontology enrichment of DEPs in larvae *Ae*. *aegypti* temephos-resistant strain.**

| **No.** | **Term ID** | **Description** | **Gene Count** | **FDR** | **Matching proteins** |
| --- | --- | --- | --- | --- | --- |
|  | **Biological process** | | | | |
| 1 | GO:1901564 | Organonitrogen compound metabolic process | 10 | 0.0014 | AAEL000109, AAEL001134, AAEL004347, AAEL004701, AAEL006169, eIF3-S4-1, AAEL007945, AAEL013694, VhaA, Adk2 |
| 2 | GO:1901566 | Organonitrogen compound biosynthetic process | 7 | 0.0088 | AAEL000109, AAEL004347, AAEL004701, eIF3-S4-1, AAEL007945, AAEL013694, Adk2 |
| 3 | GO:0008152 | Metabolic process | 11 | 0.0244 | AAEL000109, AAEL001134, AAEL004347, AAEL004701, AAEL006169, eIF3-S4-1, AAEL007945, AAEL013694, VhaA, AAEL010326, Adk2 |
| 4 | GO:0043604 | Amide biosynthetic process | 5 | 0.0244 | AAEL004347, AAEL004701, eIF3-S4-1, AAEL007945, AAEL013694 |
| 5 | GO:0044238 | Primary metabolic process | 10 | 0.0244 | AAEL000109, AAEL001134, AAEL004347, AAEL004701, AAEL006169, eIF3-S4-1, AAEL007945, AAEL013694, VhaA, Adk2 |
| 6 | GO:0044281 | Small molecule metabolic process | 5 | 0.0244 | AAEL000109, AAEL001134, AAEL004701, VhaA, Adk2 |
| 7 | GO:1901605 | Alpha-amino acid metabolic process | 3 | 0.0244 | AAEL000109, AAEL001134, AAEL004701 |
| 8 | GO:0034641 | Cellular nitrogen compound metabolic process | 8 | 0.0275 | AAEL001134, AAEL004347, AAEL004701, eIF3-S4-1, AAEL007945, AAEL013694, VhaA, Adk2 |
| 9 | GO:0006412 | Translation | 4 | 0.0387 | AAEL004347, eIF3-S4-1, AAEL007945, AAEL013694 |
| 10 | GO:0006413 | Translational initiation | 3 | 0.0387 | AAEL004347, eIF3-S4-1, AAEL007945 |

**S7. continuation.**

| **No.** | **Term ID** | **Description** | **Gene Count** | **FDR** | **Matching proteins** |
| --- | --- | --- | --- | --- | --- |
| 11 | GO:0044237 | Cellular metabolic process | 9 | 0.0387 | AAEL000109, AAEL001134, AAEL004347, AAEL004701, eIF3-S4-1, AAEL007945, AAEL013694, VhaA, Adk2 |
| 12 | GO:0044249 | Cellular biosynthetic process | 7 | 0.0387 | AAEL000109, AAEL004347, AAEL004701, eIF3-S4-1, AAEL007945, AAEL013694, Adk2 |
| 13 | GO:0044271 | Cellular nitrogen compound biosynthetic process | 6 | 0.0387 | AAEL004347, AAEL004701, eIF3-S4-1, AAEL007945, AAEL013694, Adk2 |
| 14 | GO:0019538 | Protein metabolic process | 5 | 0.0425 | AAEL004347, AAEL006169, eIF3-S4-1, AAEL007945, AAEL013694 |
| 15 | GO:0055086 | Nucleobase-containing small molecule metabolic process | 3 | 0.0425 | AAEL001134, VhaA, Adk2 |
| 16 | GO:0008652 | Cellular amino acid biosynthetic process | 2 | 0.0461 | AAEL000109, AAEL004701 |
| 17 | GO:1901607 | Alpha-amino acid biosynthetic process | 2 | 0.0461 | AAEL000109, AAEL004701 |
| **No.** | **Cellular component** | | | | |
| 1 | GO:0005737 | Cytoplasm | 9 | 0.0254 | AAEL000109, AAEL001134, Tctp, AAEL004347, AAEL006169, eIF3-S4-1, AAEL007945, AAEL013694, Adk2 |

**Notes: AAEL000109- enolase-phosphatase E1, AAEL002956-probable citrate synthase 1, AAEL003161-adenylosuccinate synthetase, VhaA-V-type proton ATPase catalytic subunit A, AAEL012172-methylthioadenosine phosphorylase, APY-apyrase, AAEL006169-lysosomal aspartic protease, AAEL010326-phosphoesterase-related protein, AAEL001134-malonate-semialdehyde dehydrogenase, AAEL004701-argininosuccinate synthase, eIF3-S4-1-eukaryotic translation initiation factor 3, AAEL007945-eukaryotic translation initiation factor 3 subunit H, AAEL013694-small subunit ribosomal protein, Adk2-adenylate kinase, Tctp-translationally controlled tumour protein homolog**
