# Supplementary material for: Quantitative proteomics analysis of permethrin and temephos-resistant Ae. aegypti revealed diverse differentially expressed proteins associated with insecticide resistance from Penang Island, Malaysia
Source: PLoS Negl Trop Dis. 2023 Sep 18;17(9):e0011604. doi: 10.1371/journal.pntd.0011604 (PMC10538732; doi:10.1371/journal.pntd.0011604)
Supplement: S1 File — (DOCX) [file pntd.0011604.s011.docx]

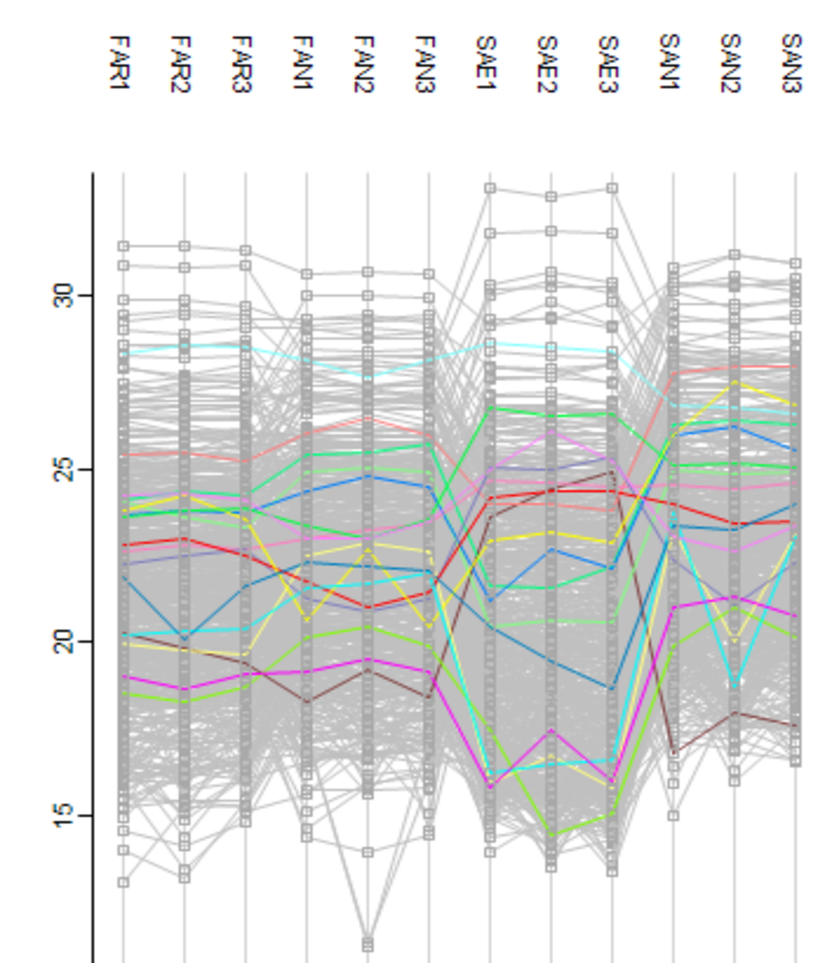


**S Fig A. Differentially expressed set of Cytochrome proteins identified by ANOVA in adult *Ae*. *aegypti* permethrin-resistant strain (q-value <0.05).** Notes: FAR: Field strain adult *Ae. aegypti* permethrin resistant. FAN: Field strain adult *Ae. aegypti* not exposed to permethrin. SAE: Laboratory strain adult *Ae. aegypti* exposed to permethrin. SAN: Laboratory strain adult *Ae. aegypti* not exposed to permethrin.

**Notes:**

| 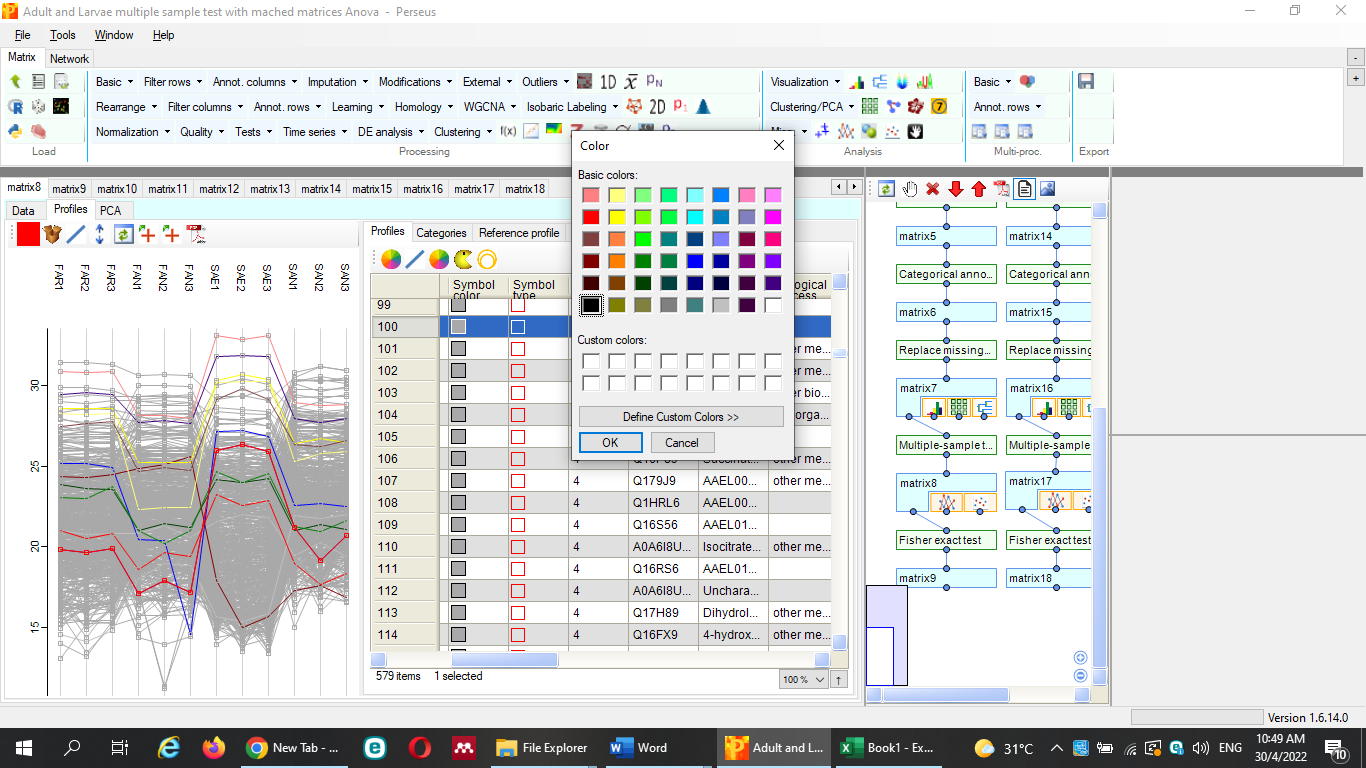 | Ubiquinol-cytochrome c reductase complex core protein (A0A6I8TBV7) | 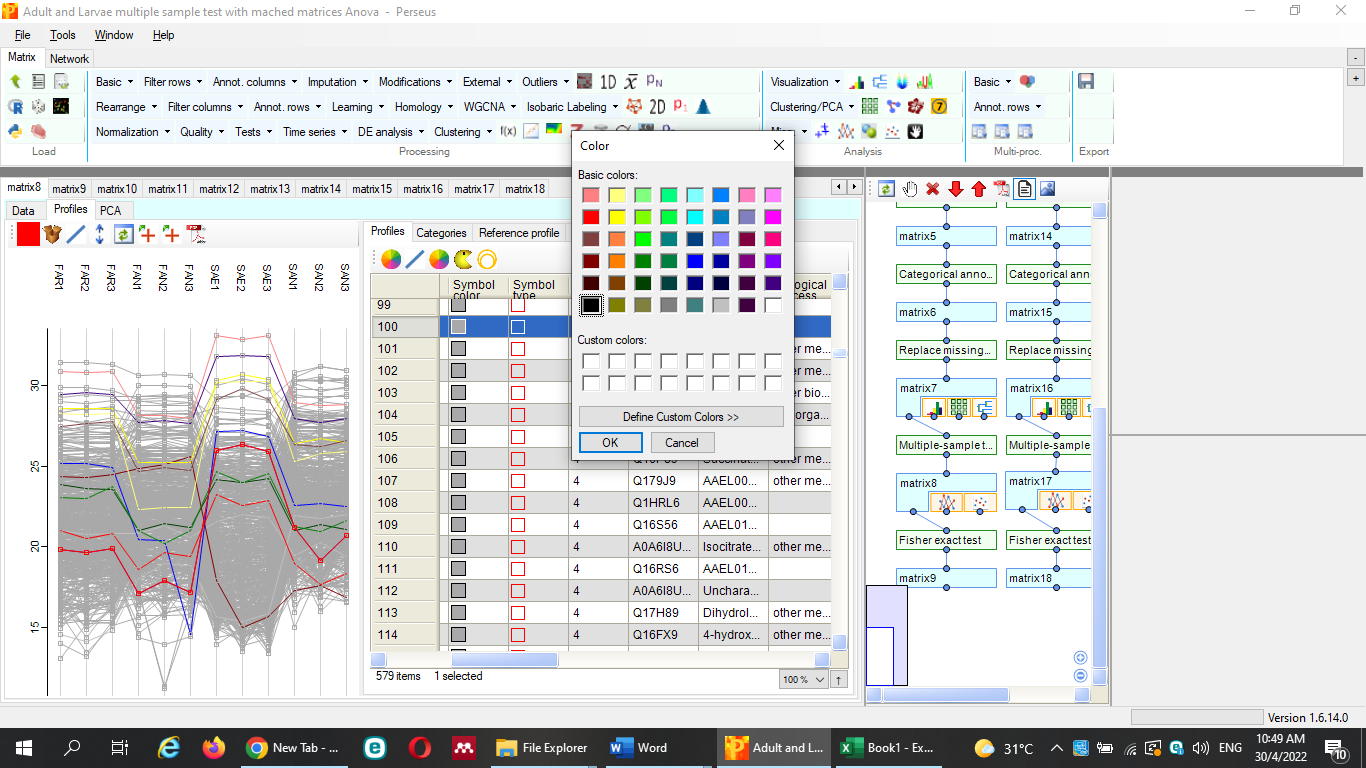 | Ubiquinol-cytochrome c reductase complex core protein (Q17AK0) |
| --- | --- | --- | --- |
| 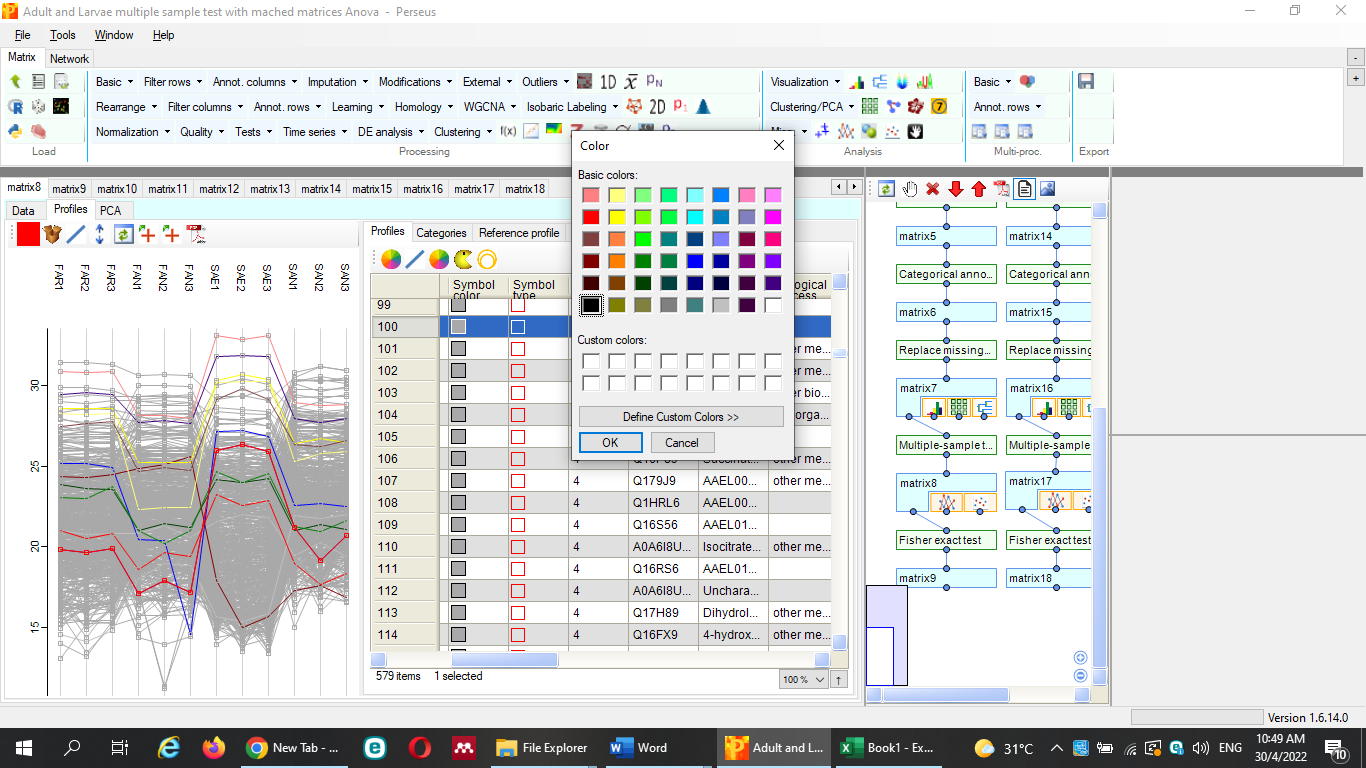 | Cytochrome C1 (Q16NS5) | 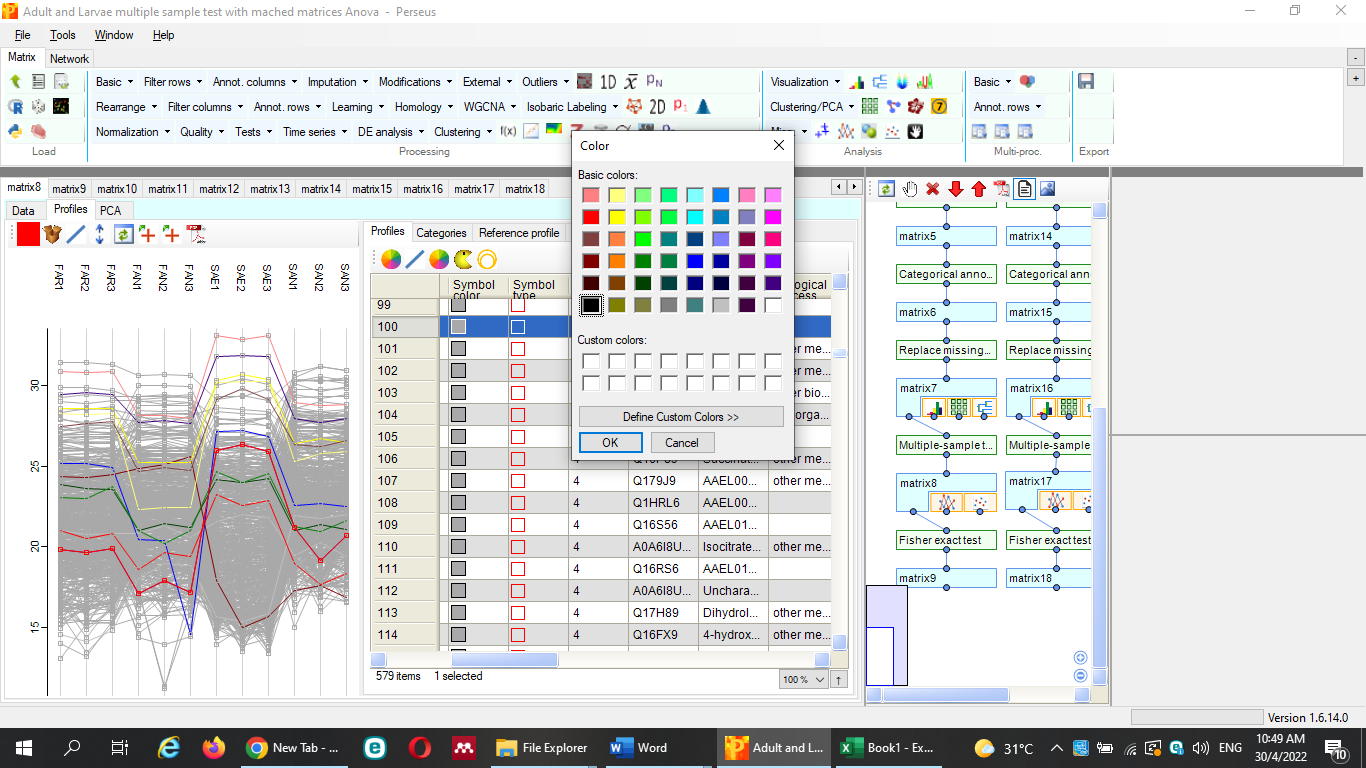 | Cytochrome c oxidase subunit 4 (Q16KF6) |
| 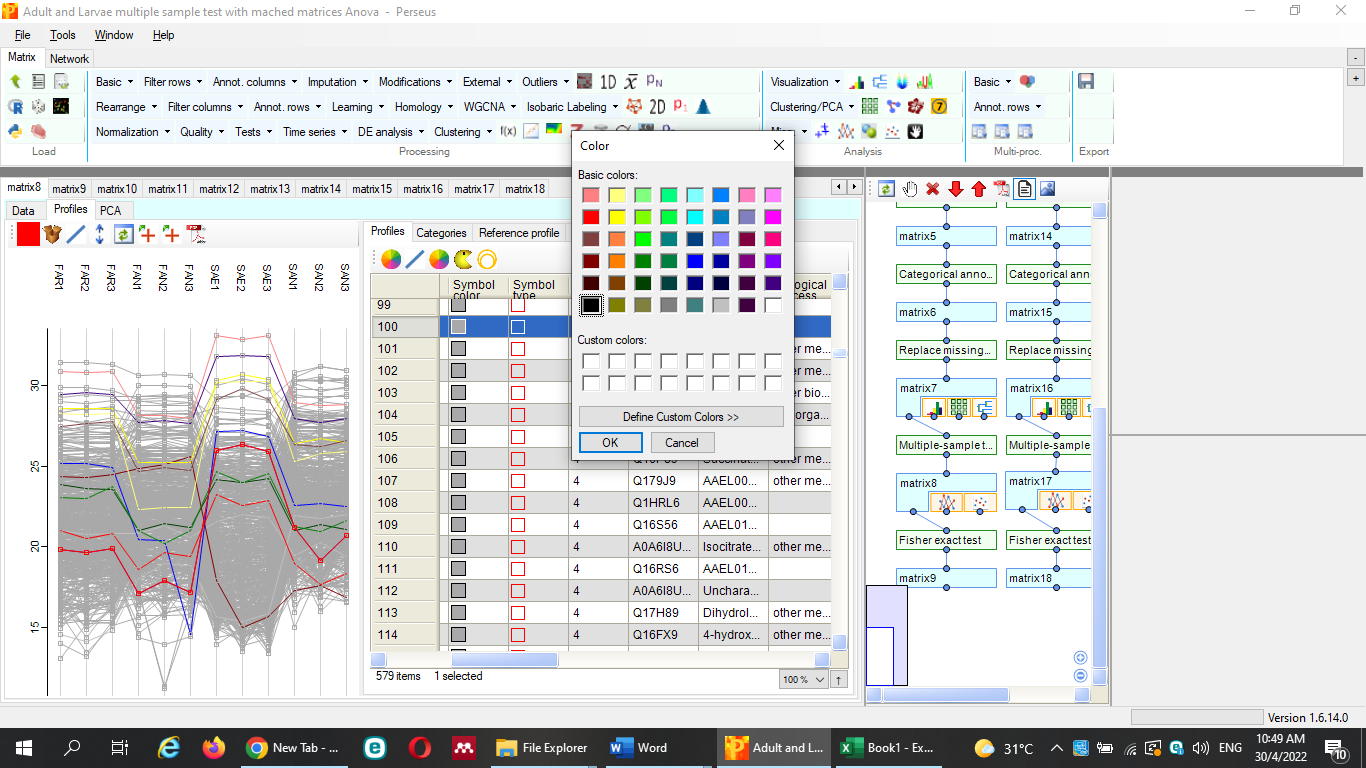 | Mitochondrial cytochrome c (Q1HRI9) | 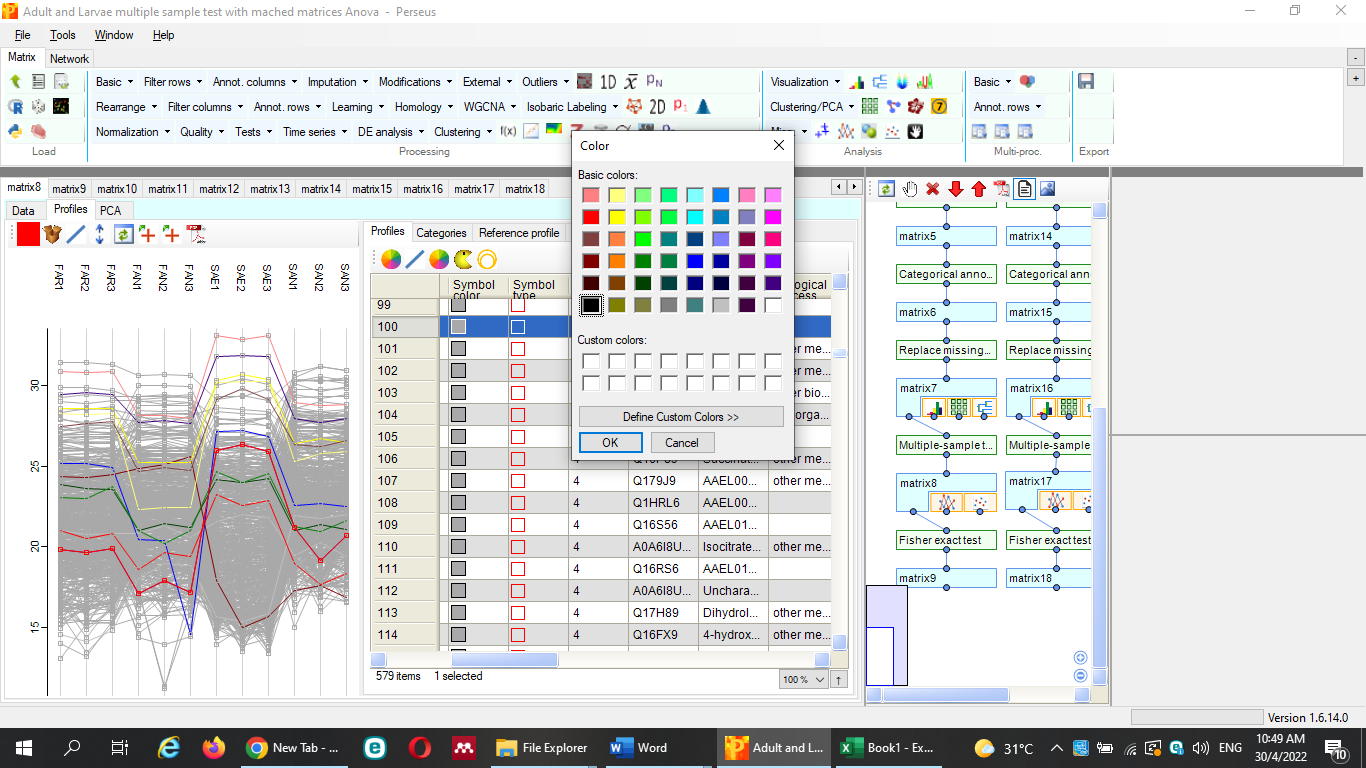 | Cytochrome b-c1 complex subunit Rieske, mitochondrial (Q17EQ1) |
| 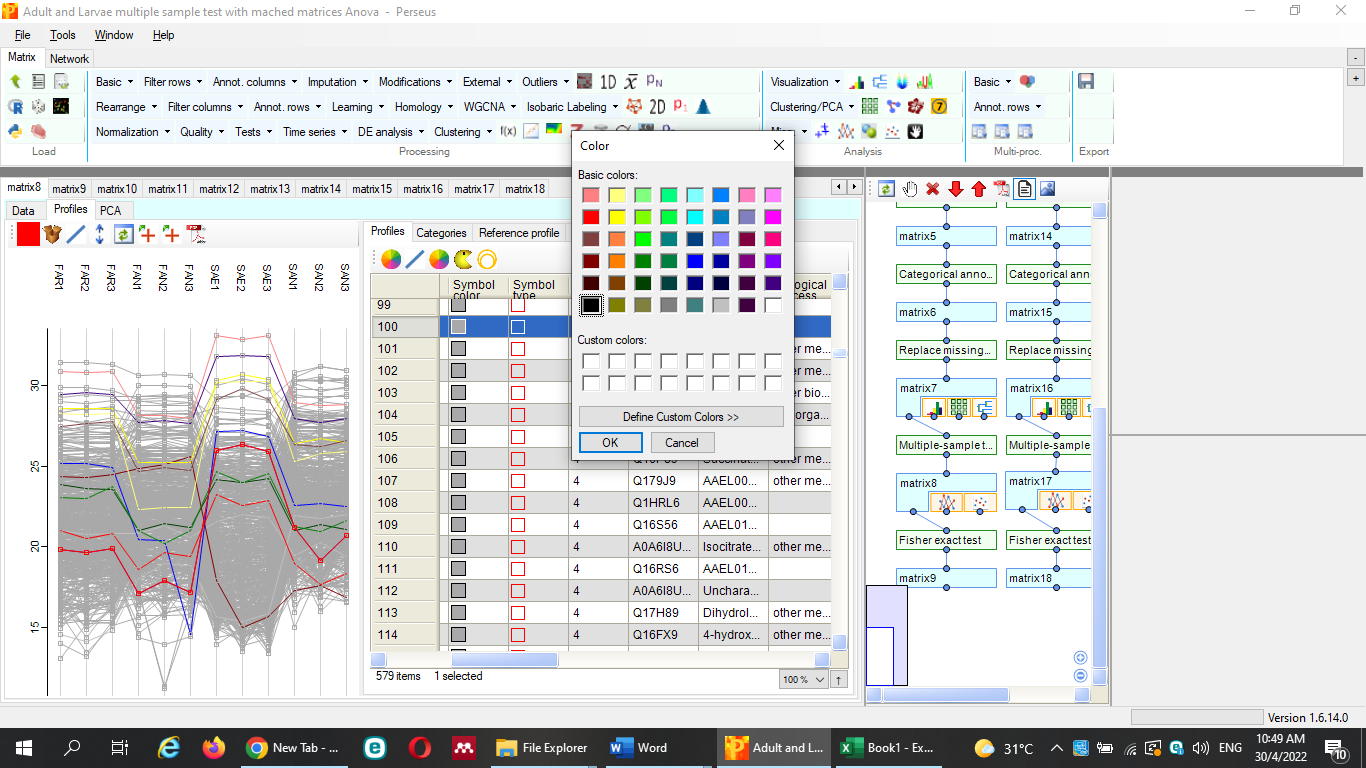 | Cytochrome b-c1 complex subunit 7 (Q5MM88) | 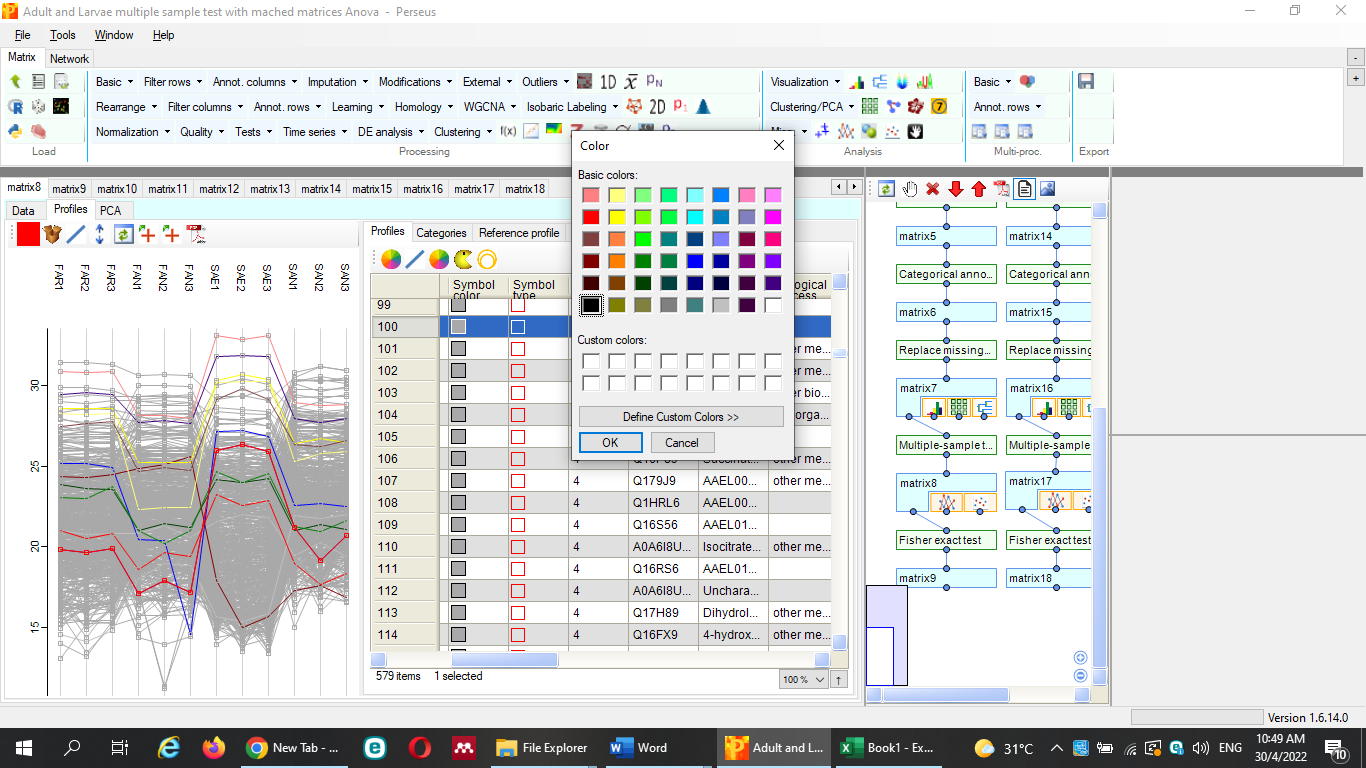 | Cytochrome c oxidase polypeptide Va (Q1HRJ0) |
| 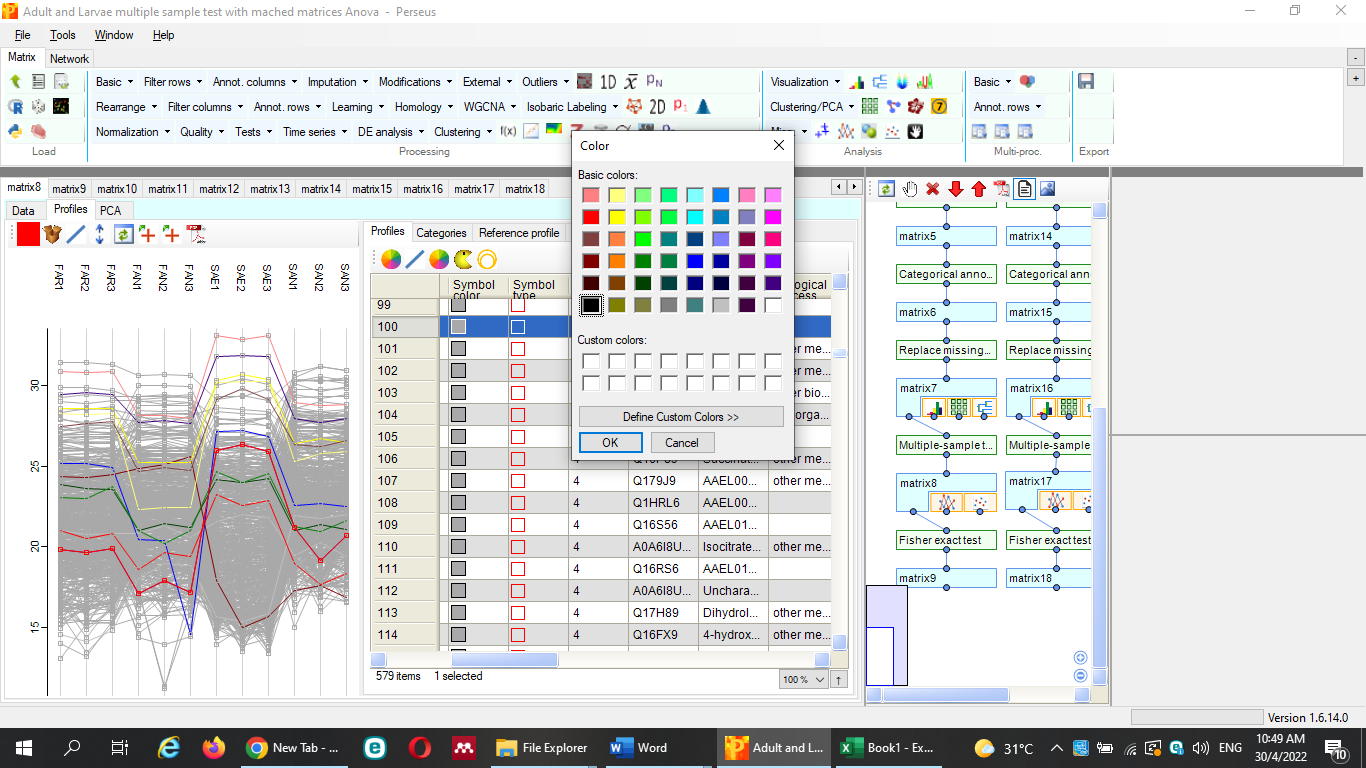 | Cytochrome c oxidase (Q16KF9) | 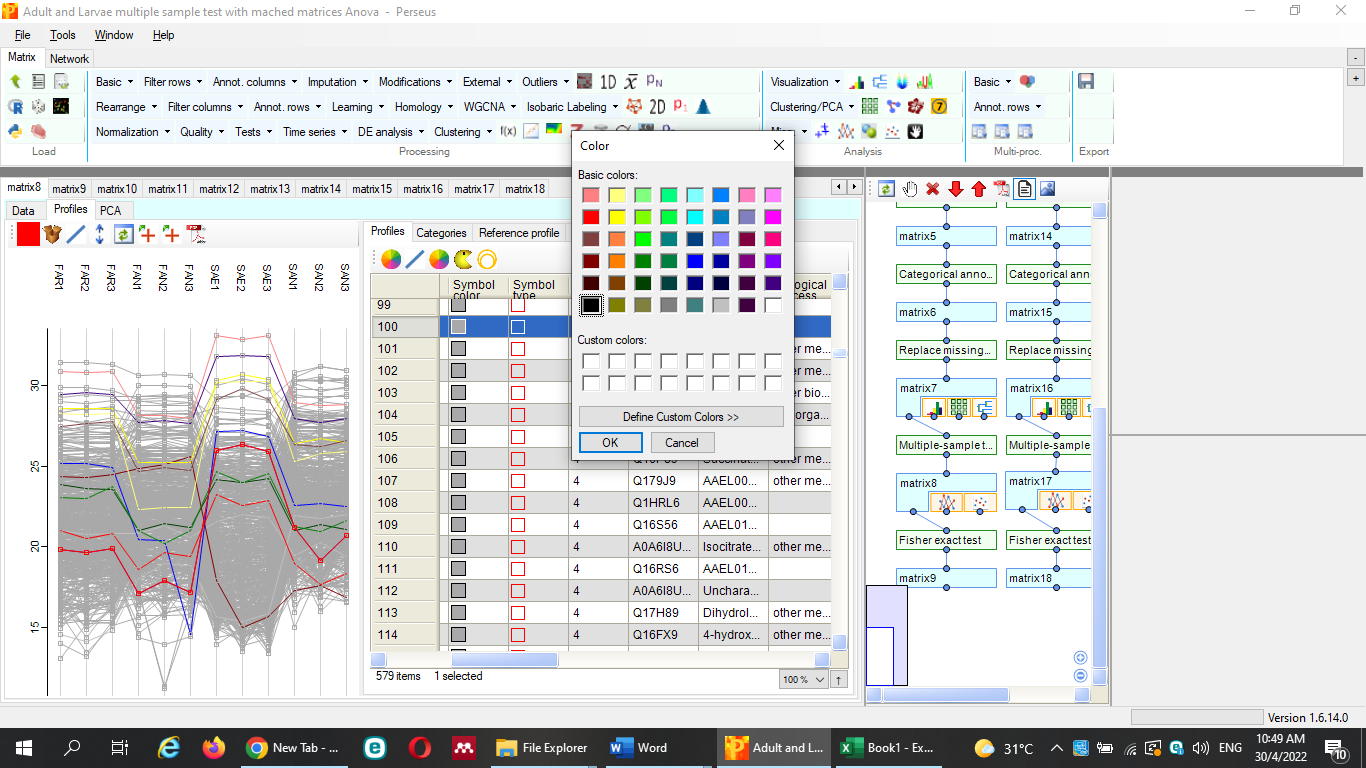 | Cytochrome b5 (cytb5) (Q16U40) |
| 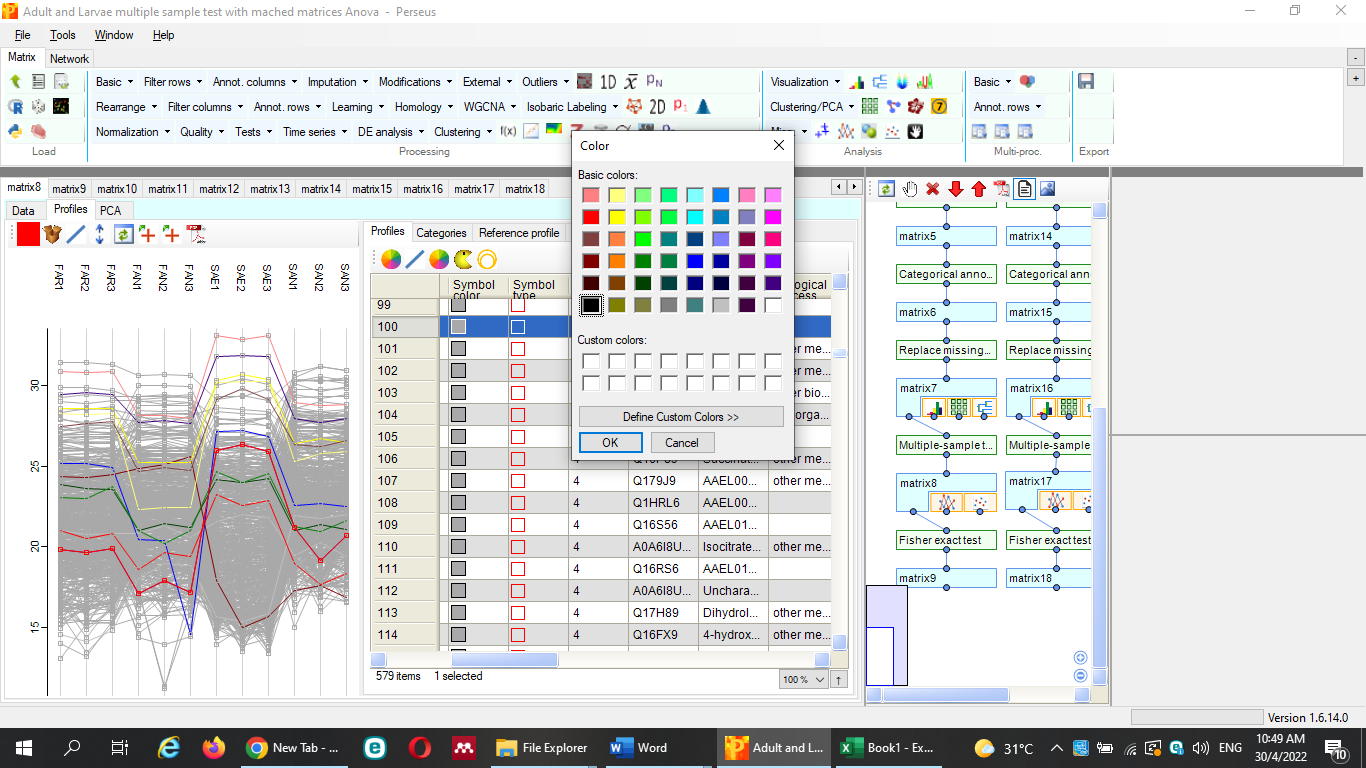 | NADPH--cytochrome P450 reductase (Q17FM7) | 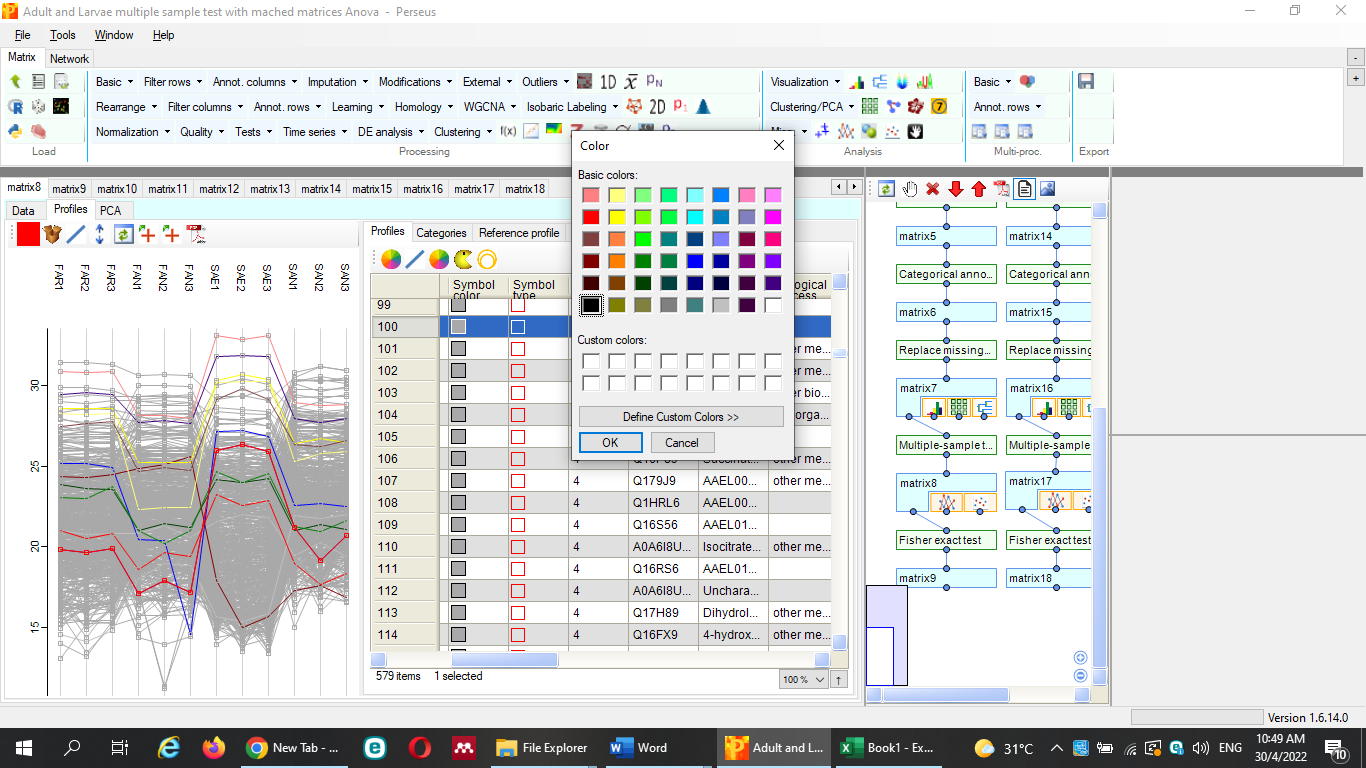 | Cytochrome c oxidase subunit (Q16XG8) |
| 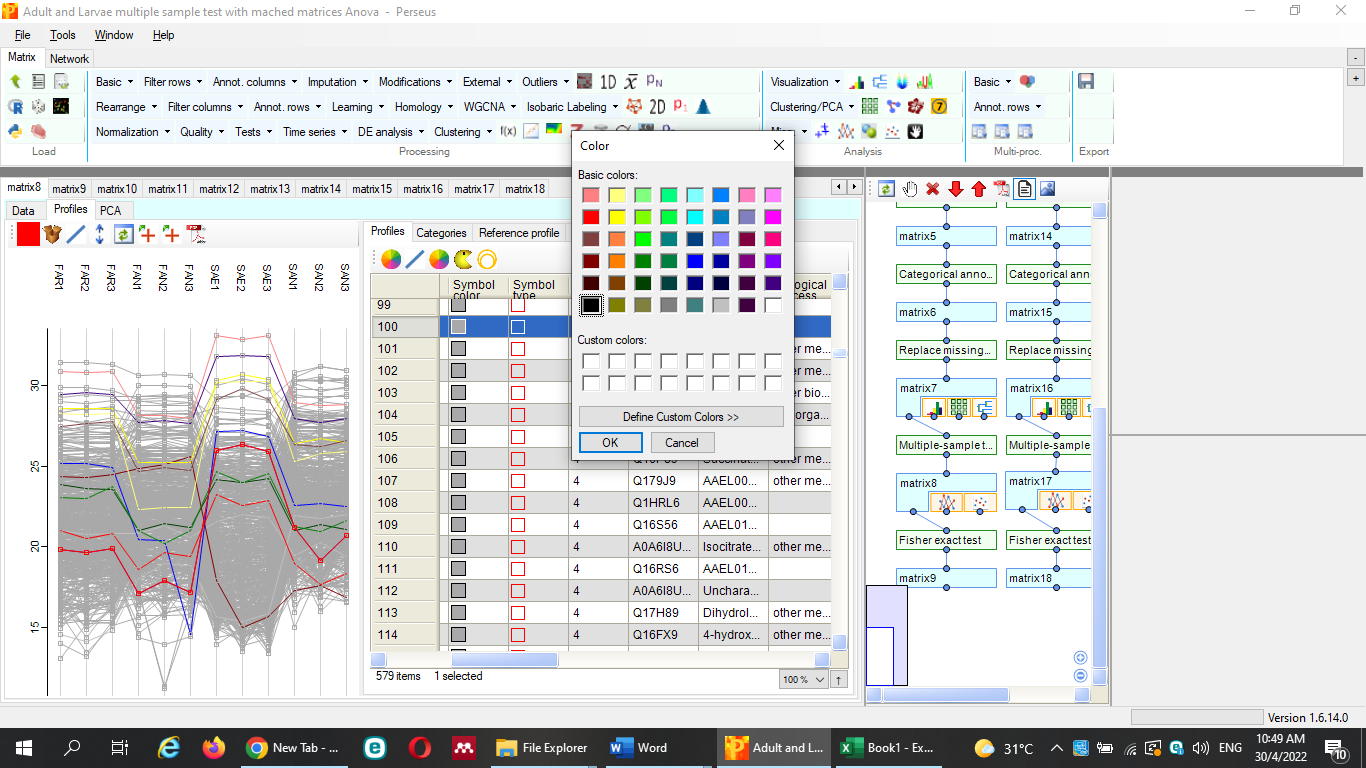 | Cytochrome c oxidase, subunit VIA, putative (Q1HRM4) | 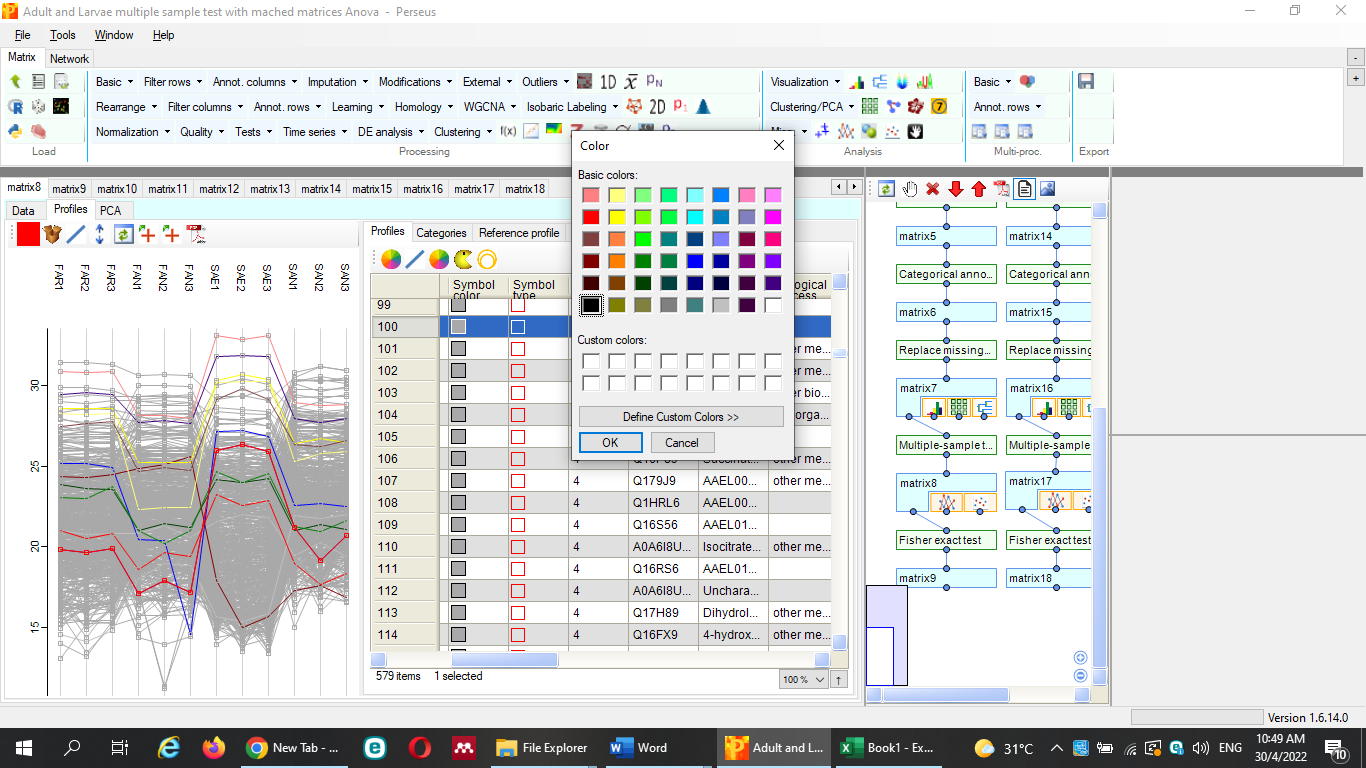 | Mitochondrial cytochrome c oxidase subunit VIC (Q1HQE0) |
| 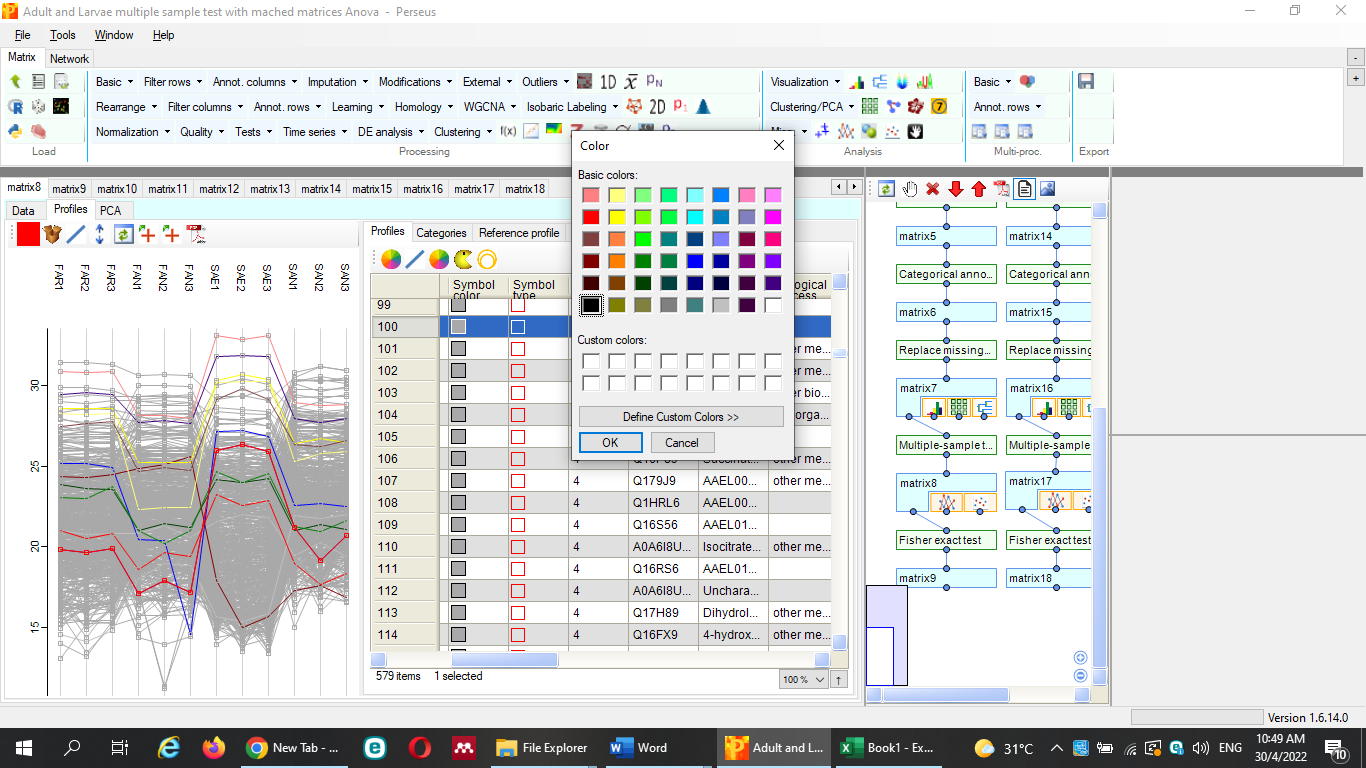 | Cytochrome c oxidase subunit (Q16XG7) | 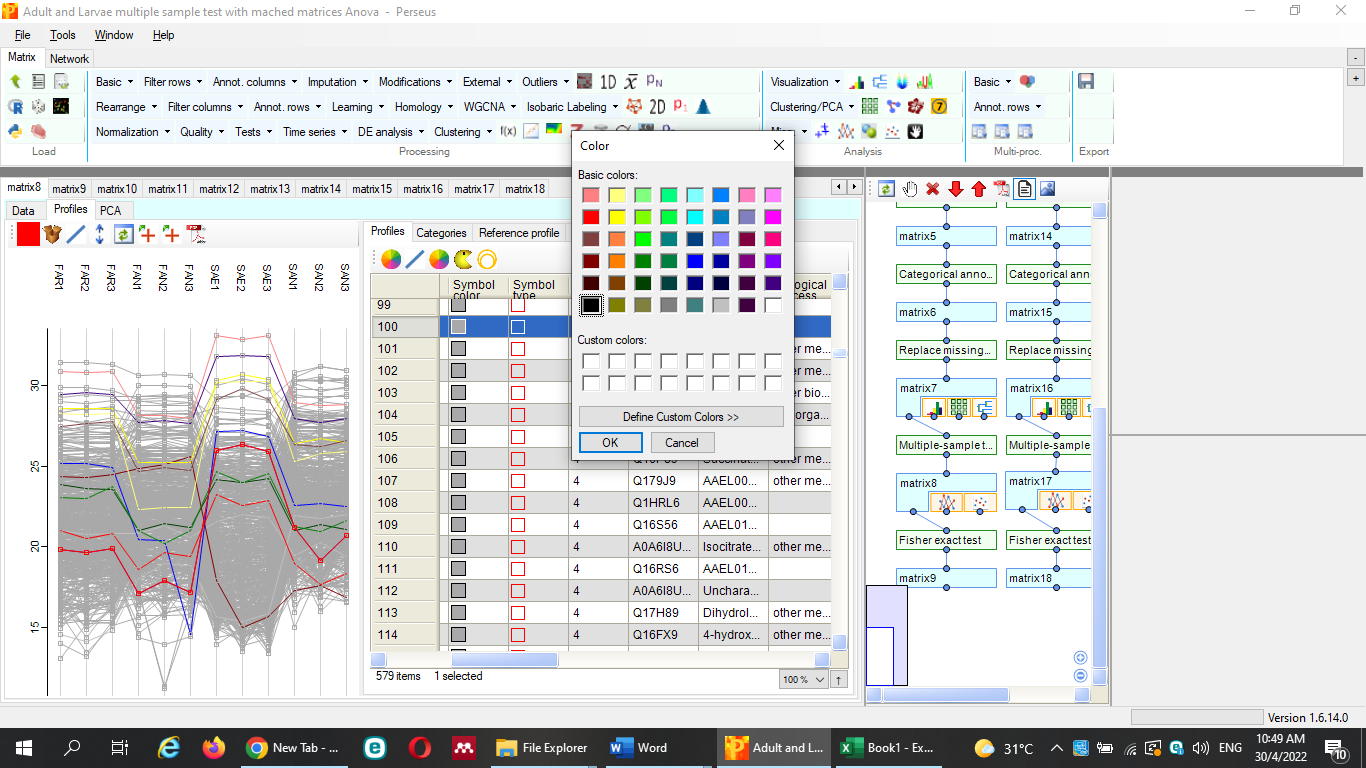 | Succinate dehydrogenase cytochrome b small subunit (A0A023EI91) |
| 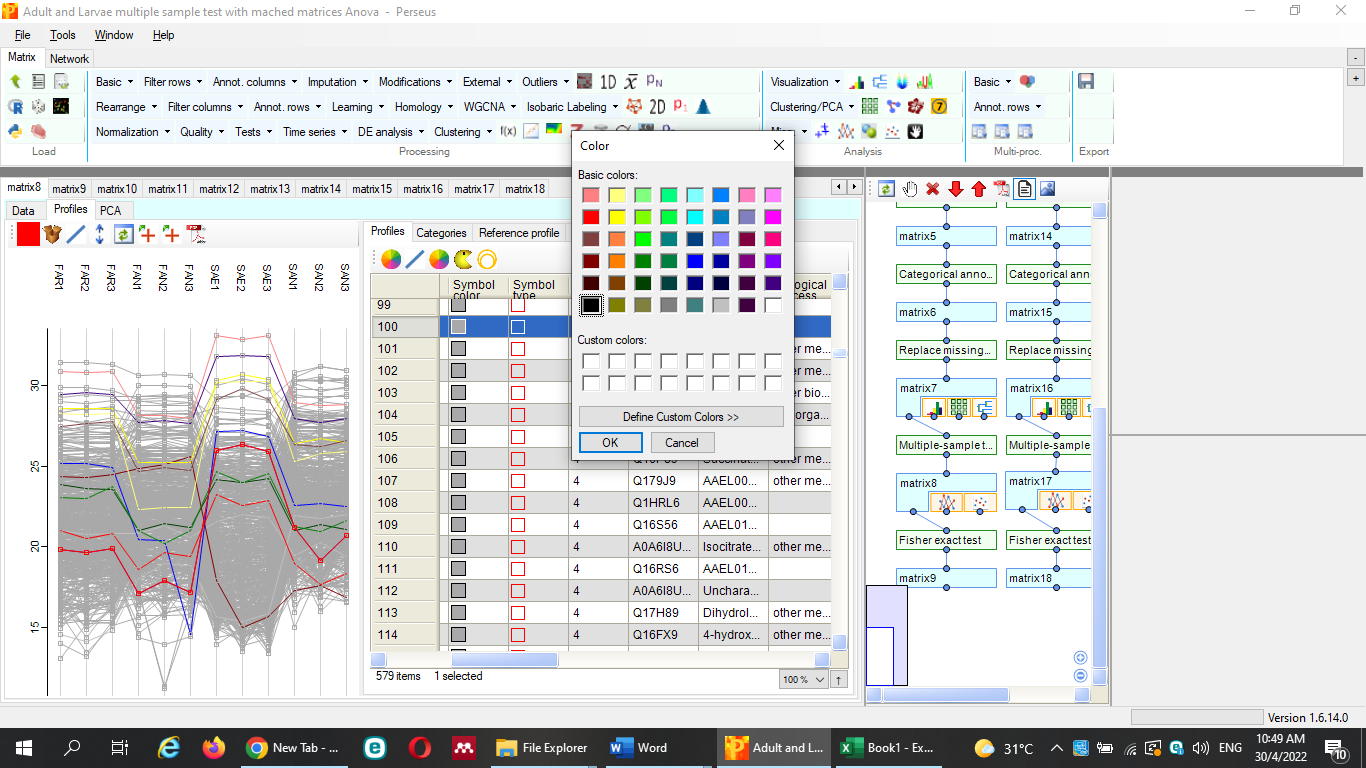 | Putative mitochondrial ubiquinol-cytochrome c reductase hinge protein (A0A023EDZ6) |  |  |


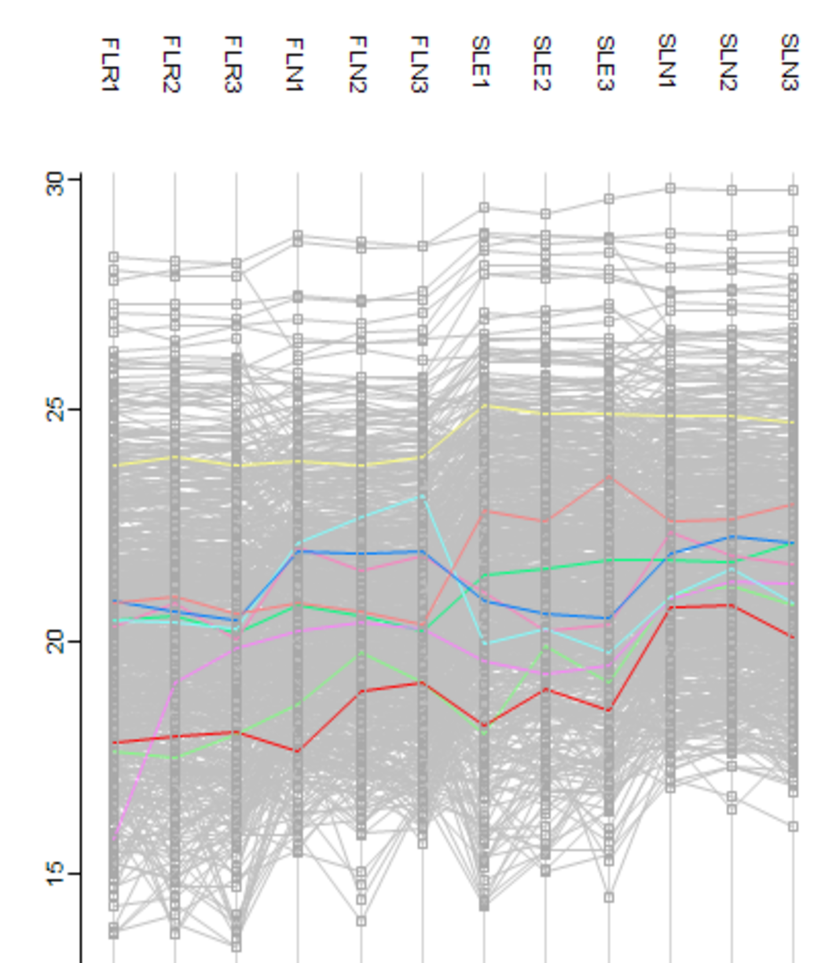


**S Fig B. Cytochrome proteins identified by ANOVA in *Ae*. *aegypti* larvae temephos resistant strain (q-value <0.05).** Notes: FLR: Field strain *Ae. aegypti* larvae temephos resistant. FLN: Field strain *Ae. aegypti* larvae not exposed to temephos. SLE: Laboratory strain *Ae. aegypti* larvae exposed to temephos. SLN: Laboratory strain *Ae. aegypti* larvae not exposed to temephos.

**Notes:**

| 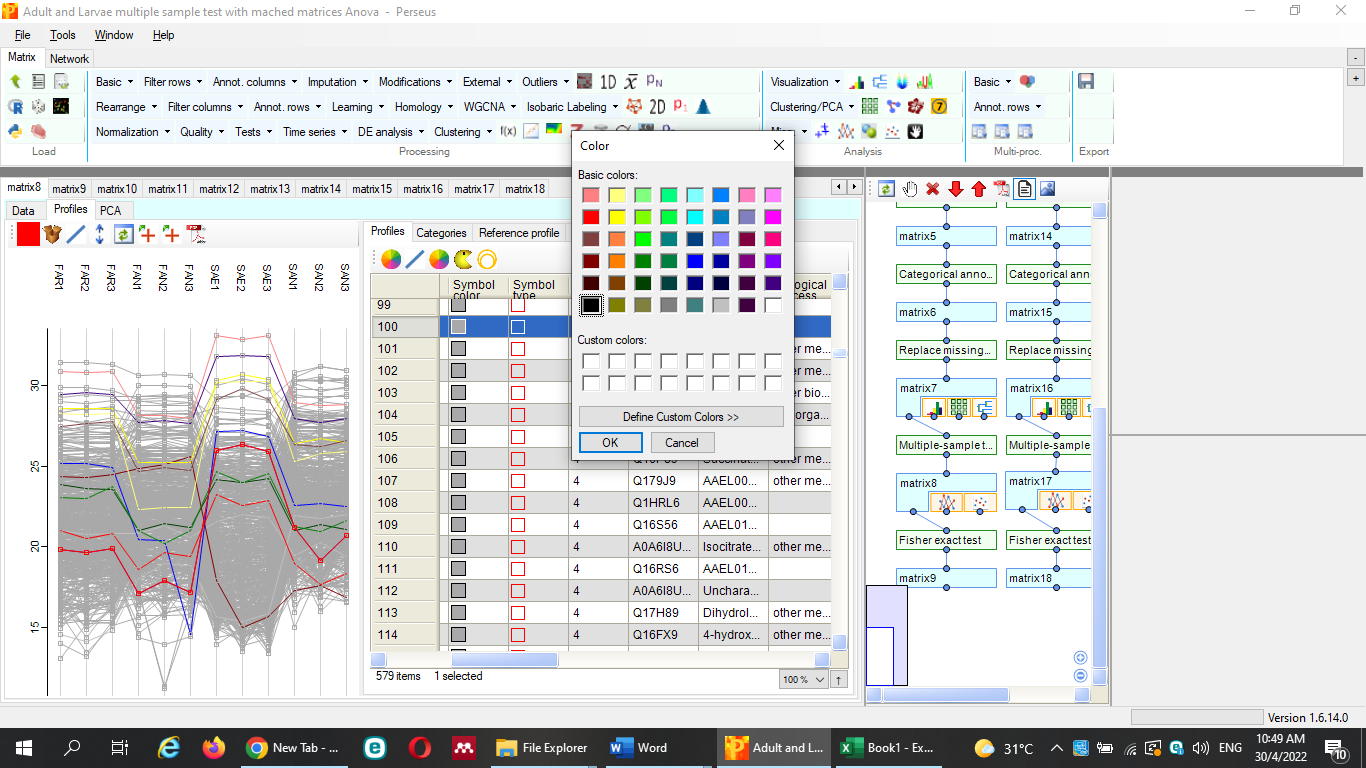 | Ubiquinol-cytochrome c reductase complex core protein (A0A6I8TBV7) | 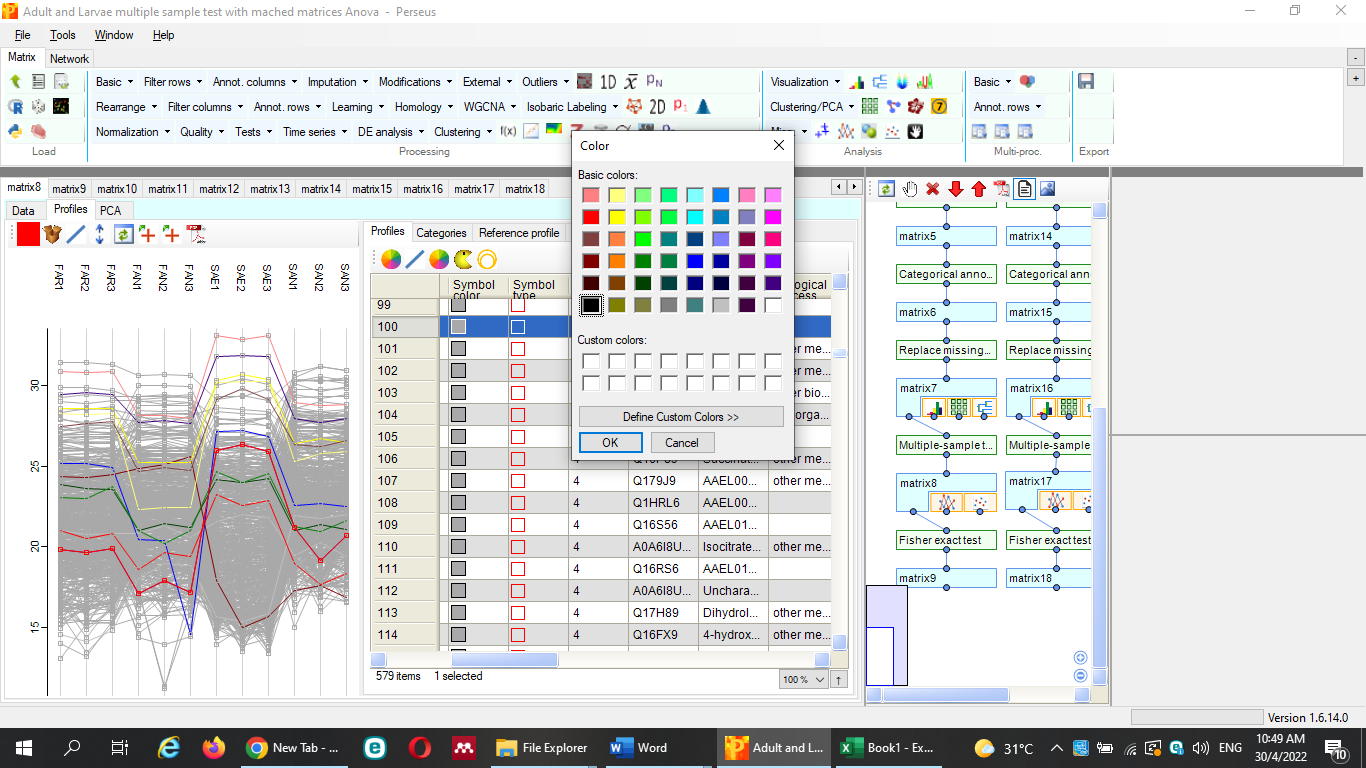 | Ubiquinol-cytochrome c reductase complex core protein (Q17AK0) |
| --- | --- | --- | --- |
| 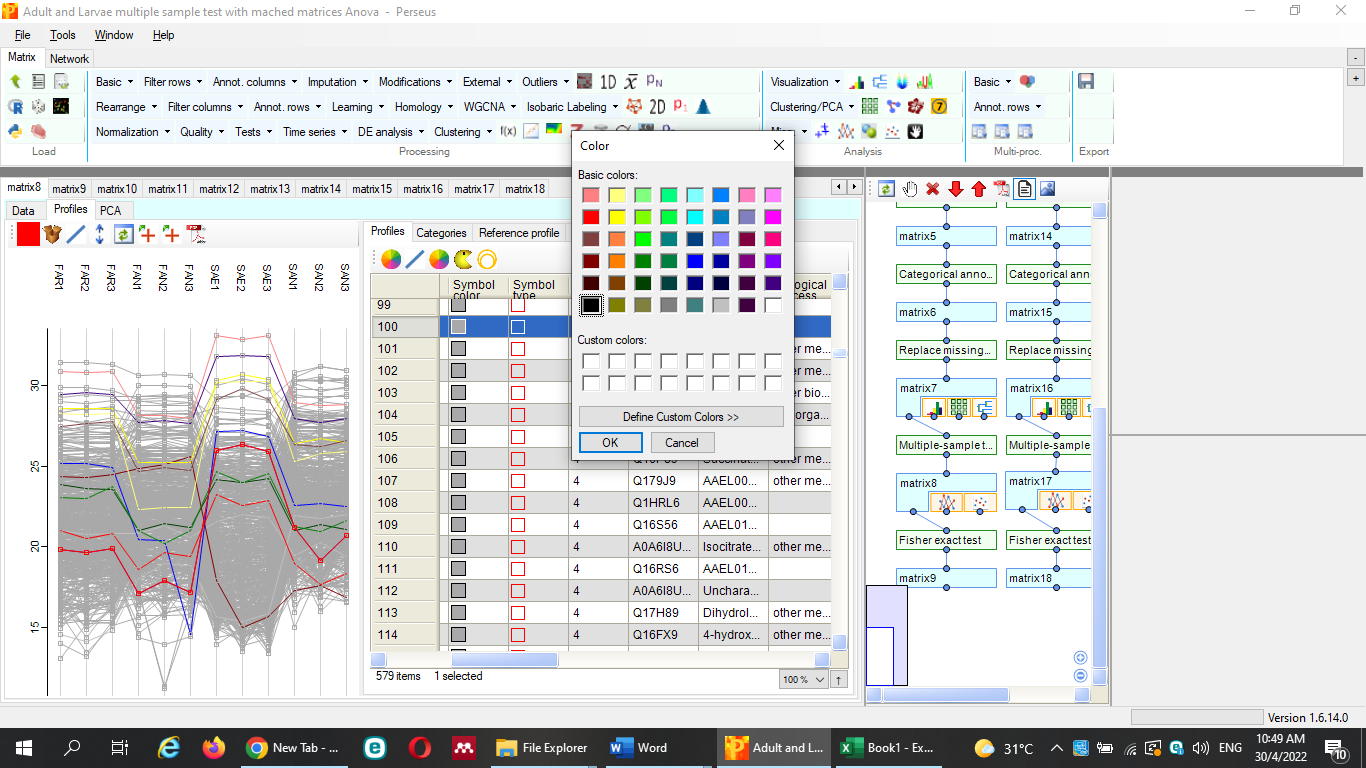 | Cytochrome c oxidase subunit 4 (Q16KF6) | 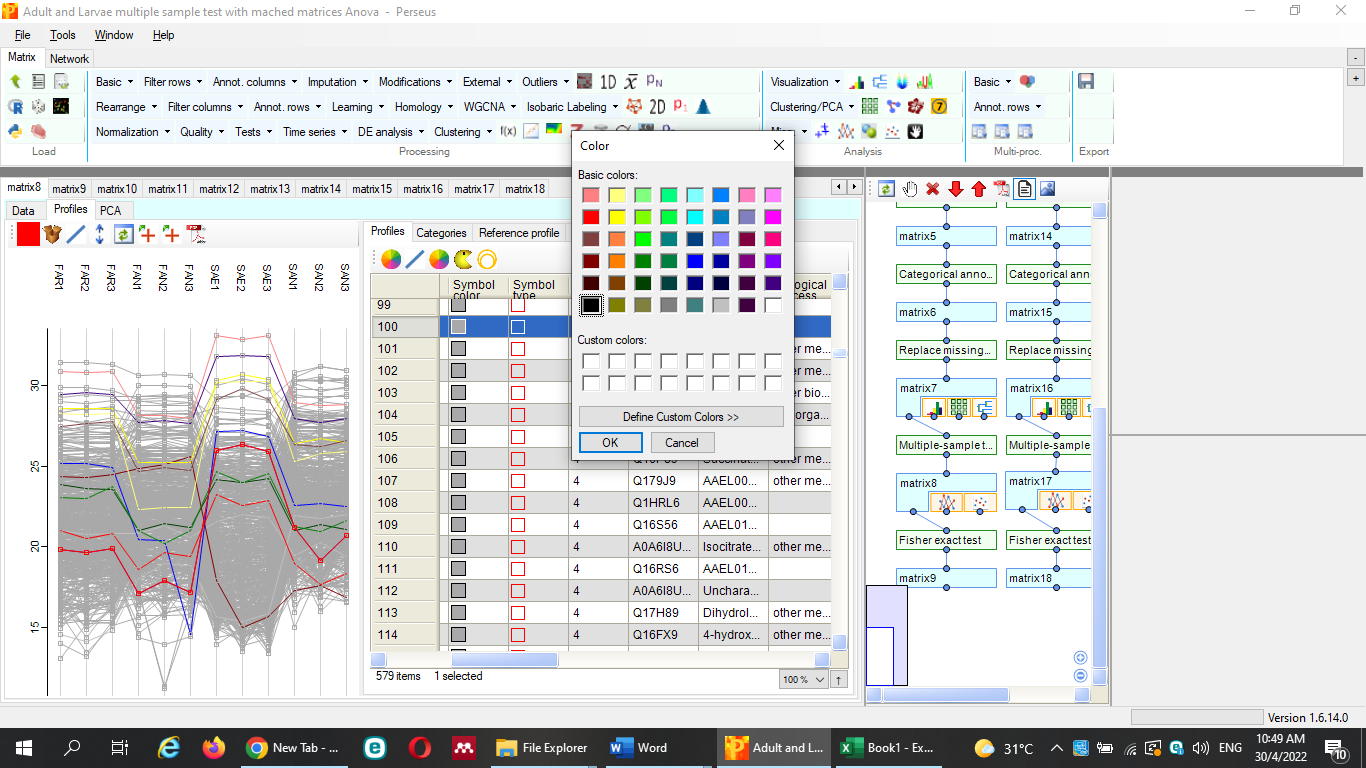 | Cytochrome b-c1 complex subunit Rieske, mitochondrial (Q17EQ1) |
| 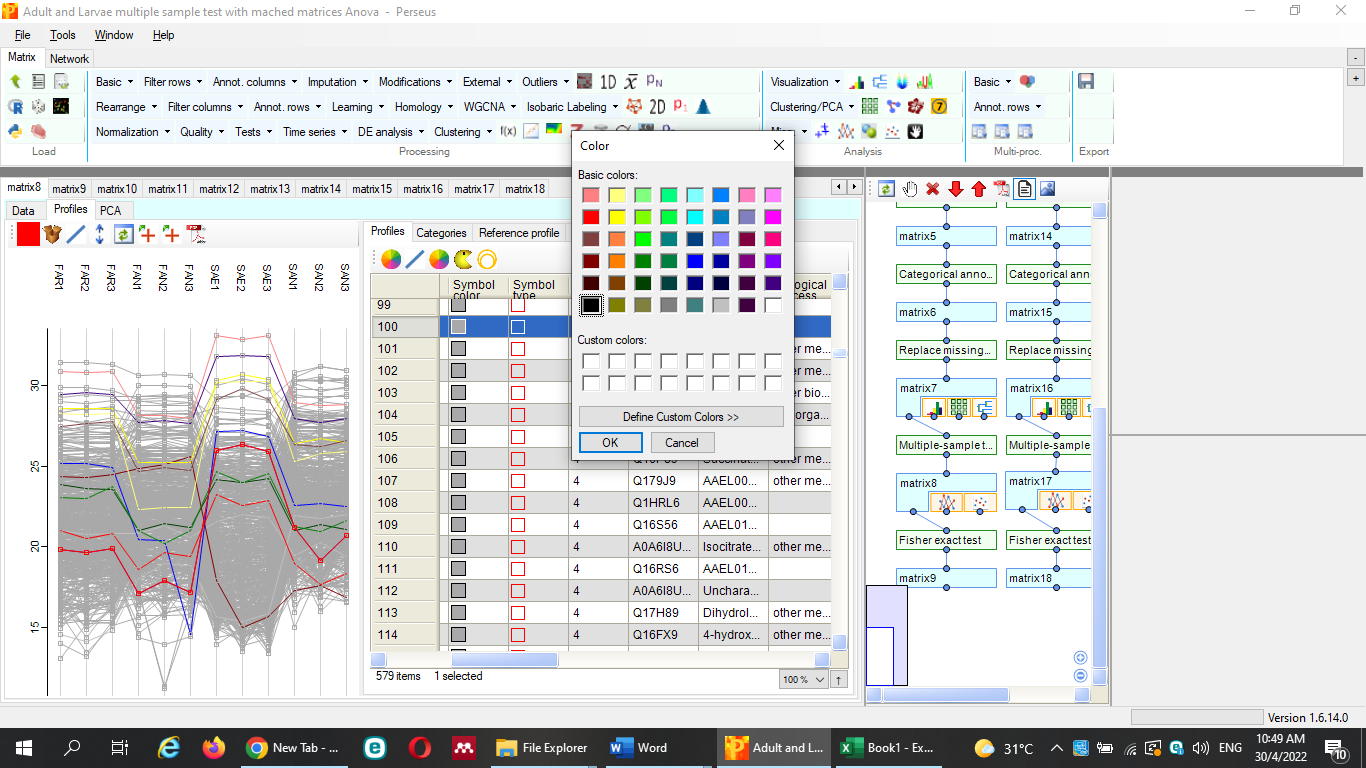 | Cytochrome b5 (cytb5) (Q16U40) | 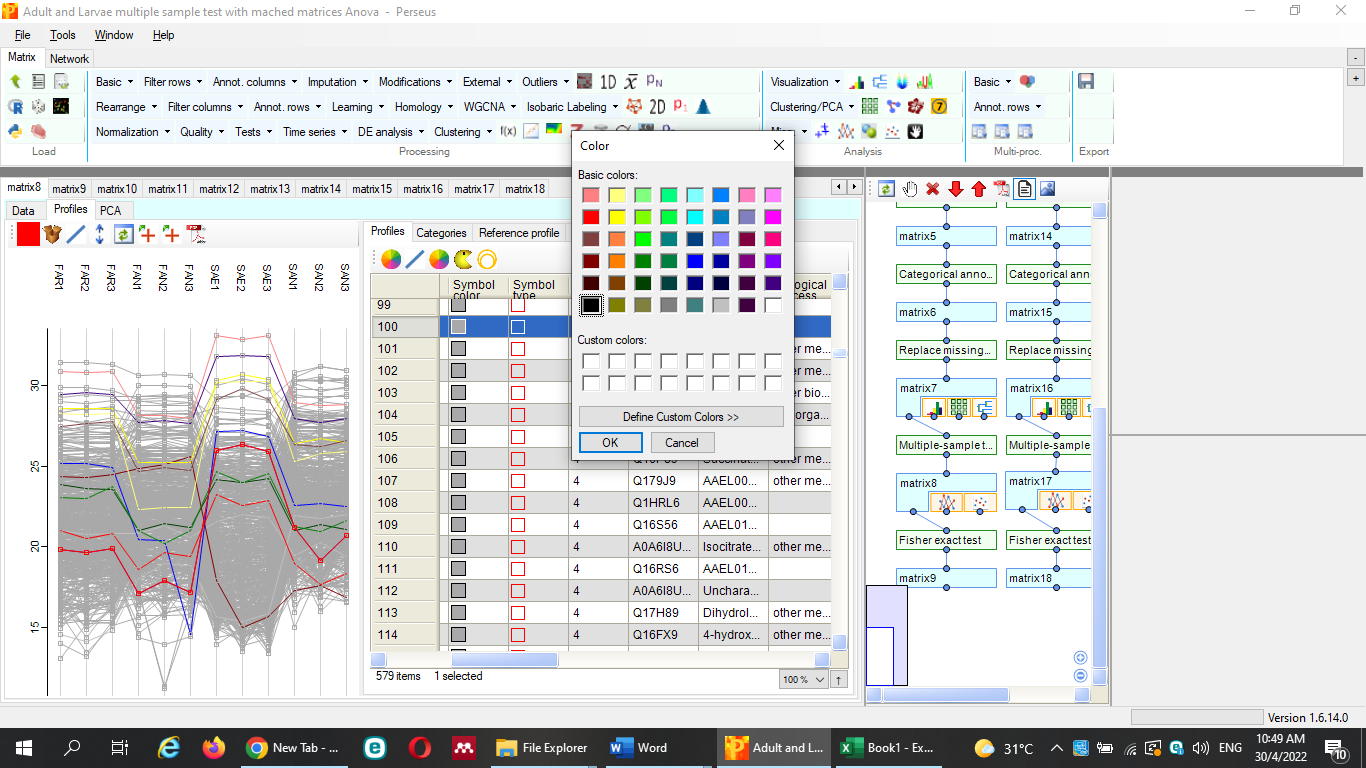 | NADPH--cytochrome P450 reductase (Q17FM7) |
| 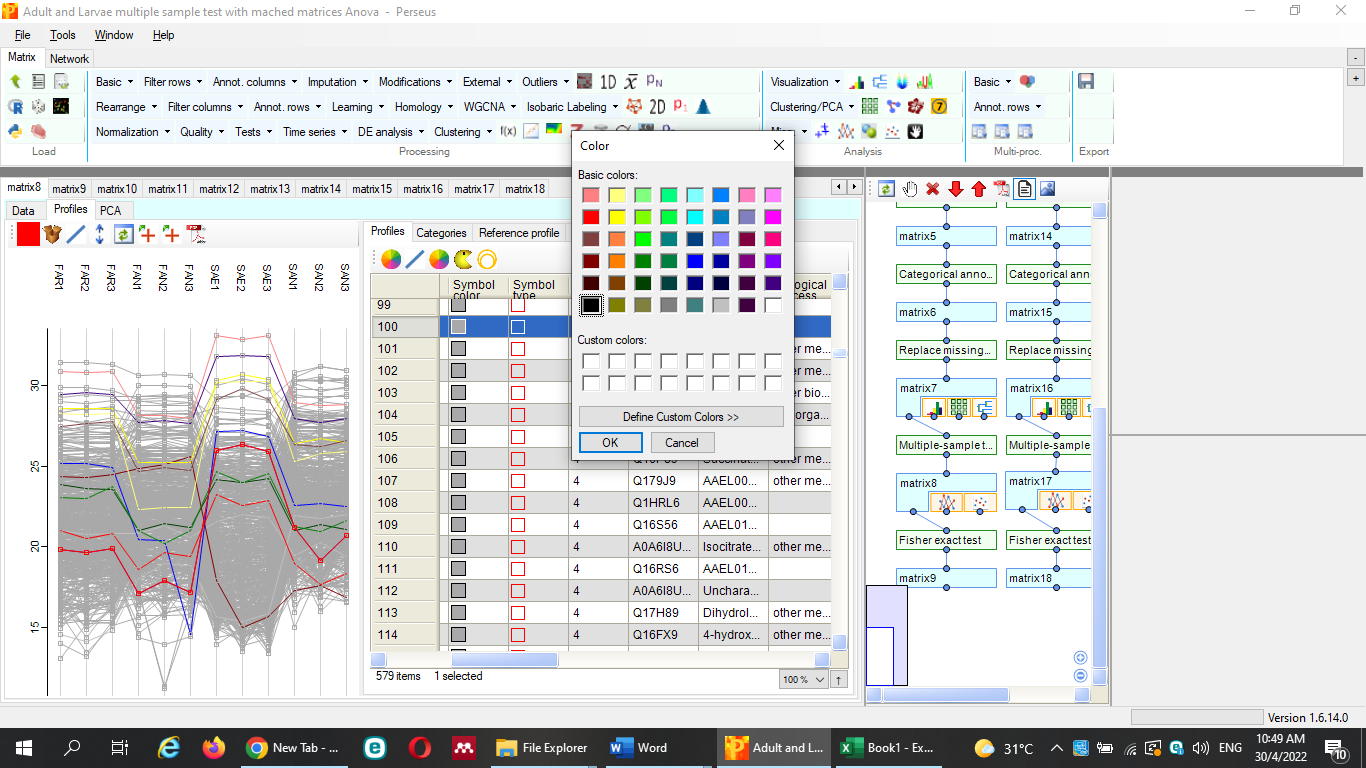 | CYP9J27 (A0A1D8GRB8) | 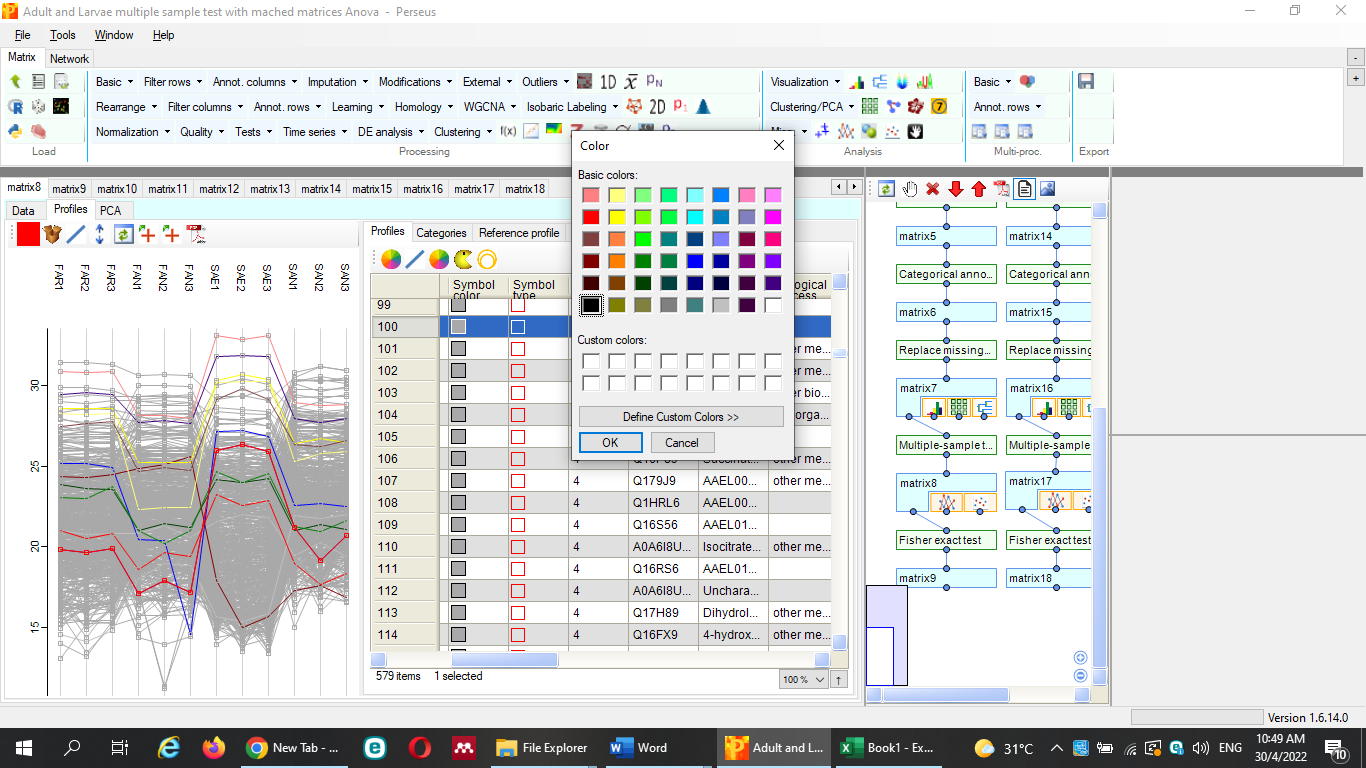 | CYP9J9 (Q16FV9) |
| 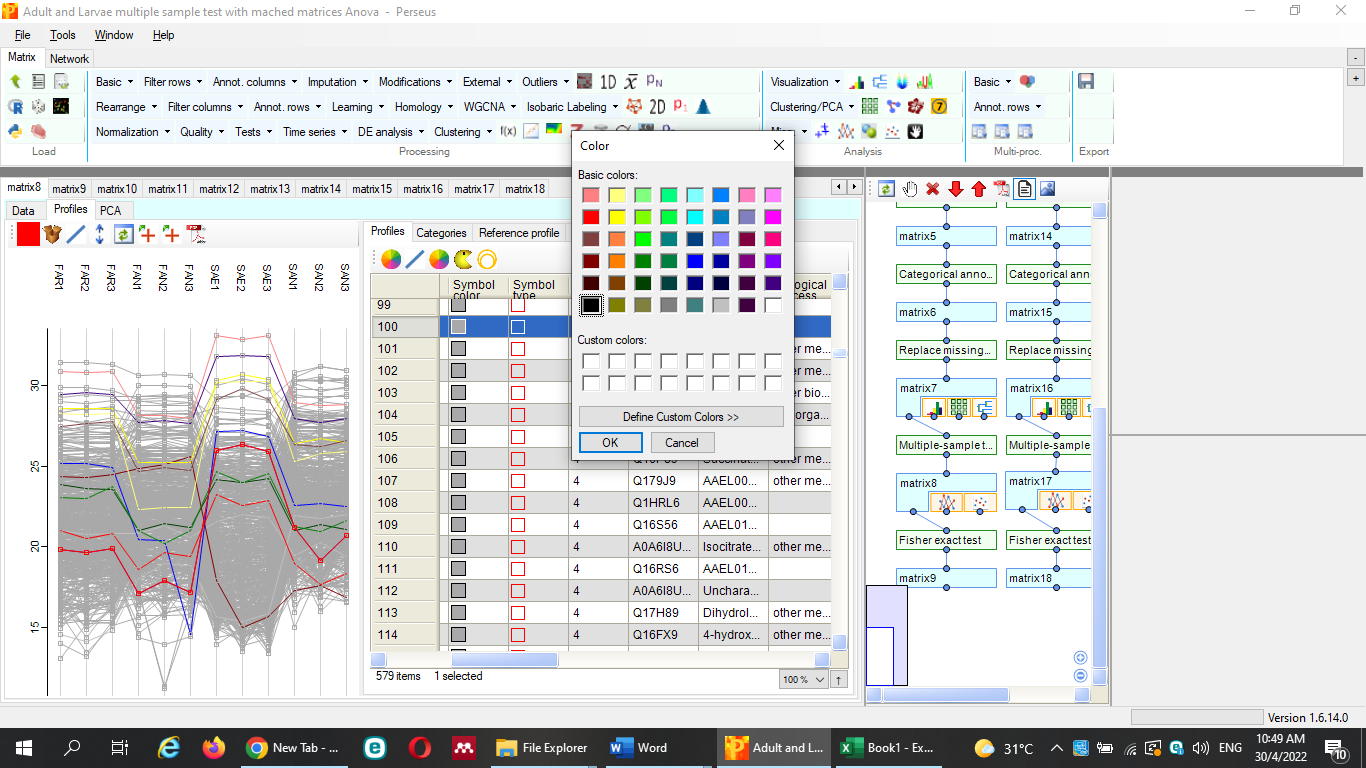 | CYP6AG4 (Q173V1) |  |  |


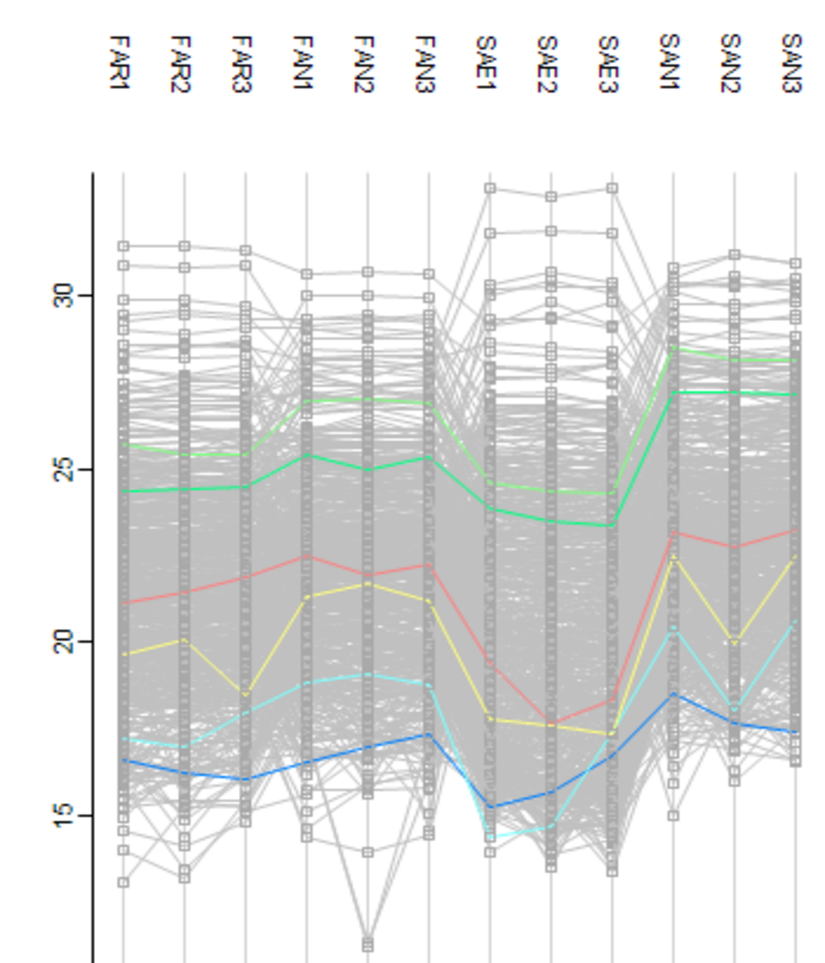


**S Fig C. GST proteins identified by ANOVA in adult *Ae*. *aegypti* permethrin-resistant strain (q-value <0.05).** Notes: FAR: Field strain adult *Ae. aegypti* permethrin resistant. FAN: Field strain adult *Ae. aegypti* not exposed to permethrin. SAE: Laboratory strain adult *Ae. aegypti* exposed to permethrin. SAN: Laboratory strain adult *Ae. aegypti* not exposed to permethrin.

**Notes:**

| 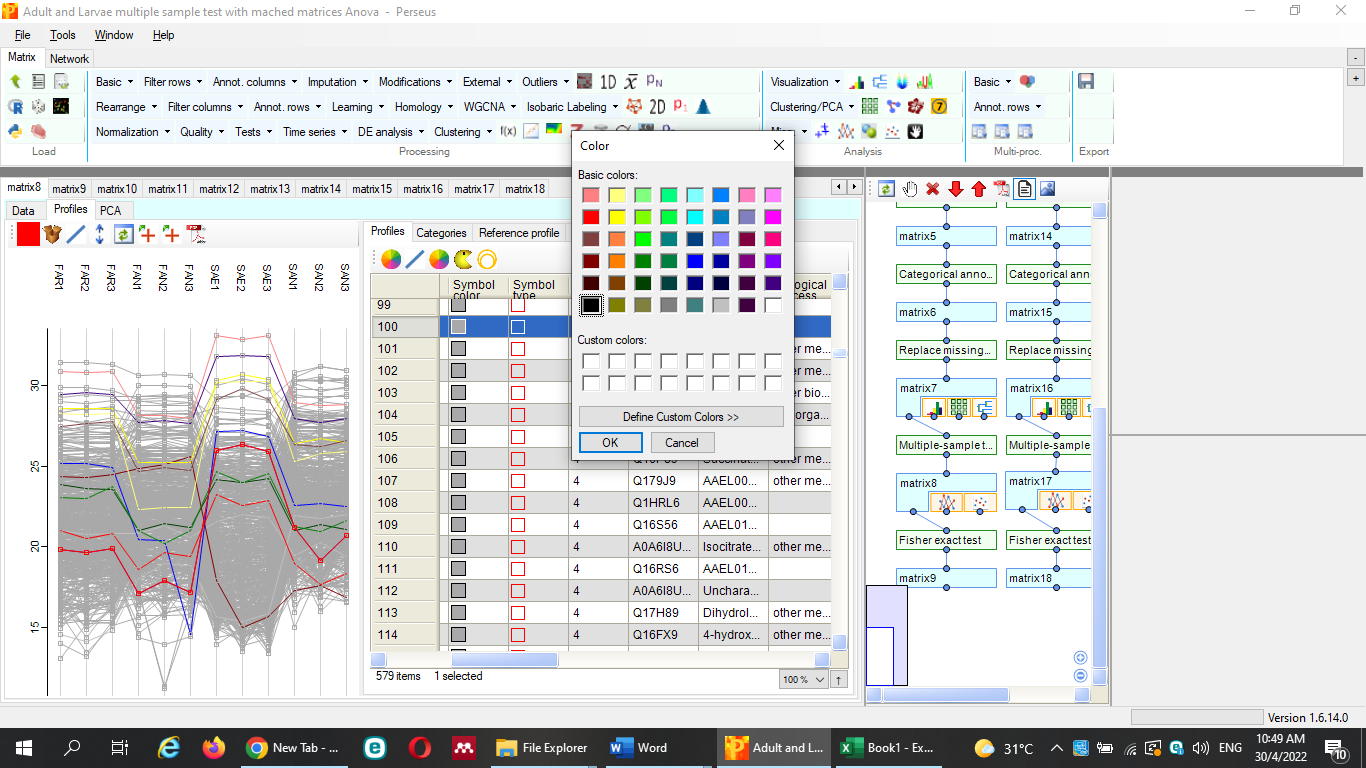 | GSTD1 (J9HHL7) | 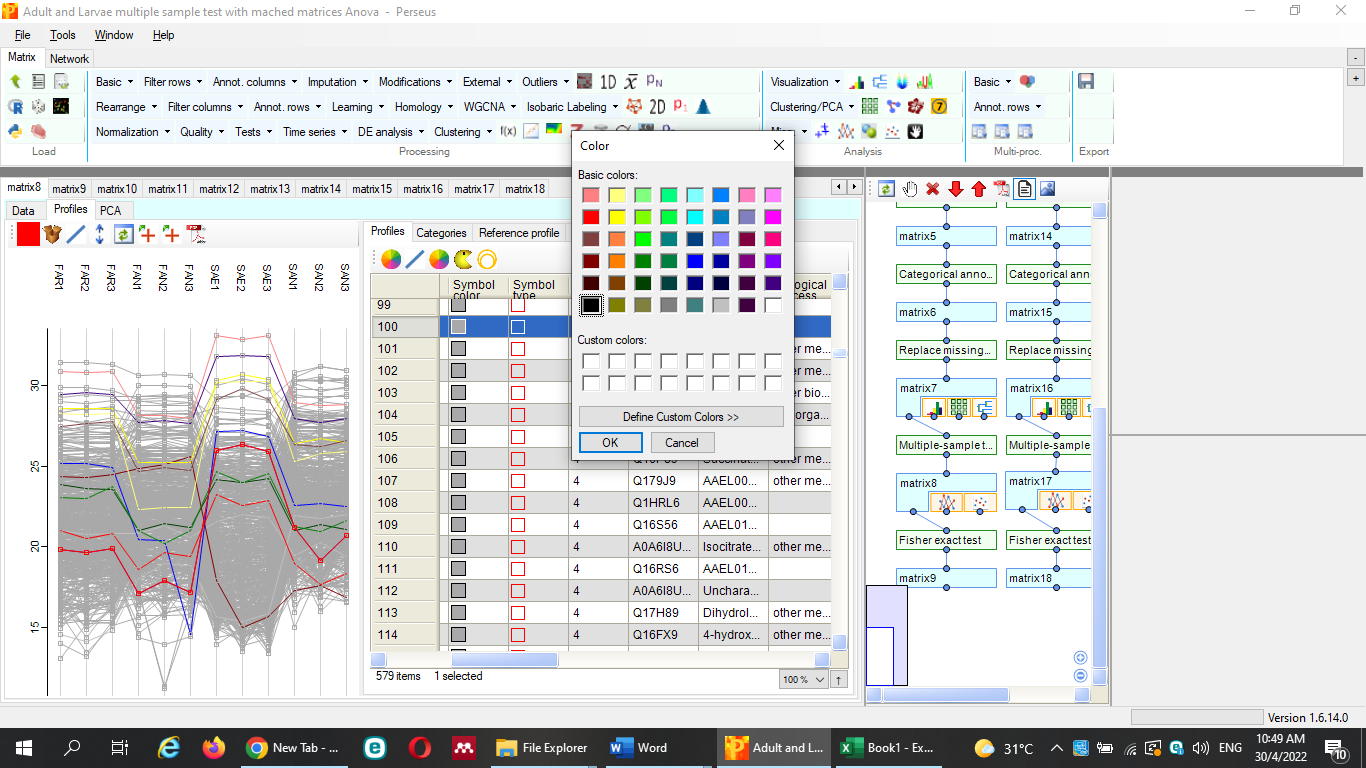 | GSTD1 (A0A1S4EXN8) |
| --- | --- | --- | --- |
| 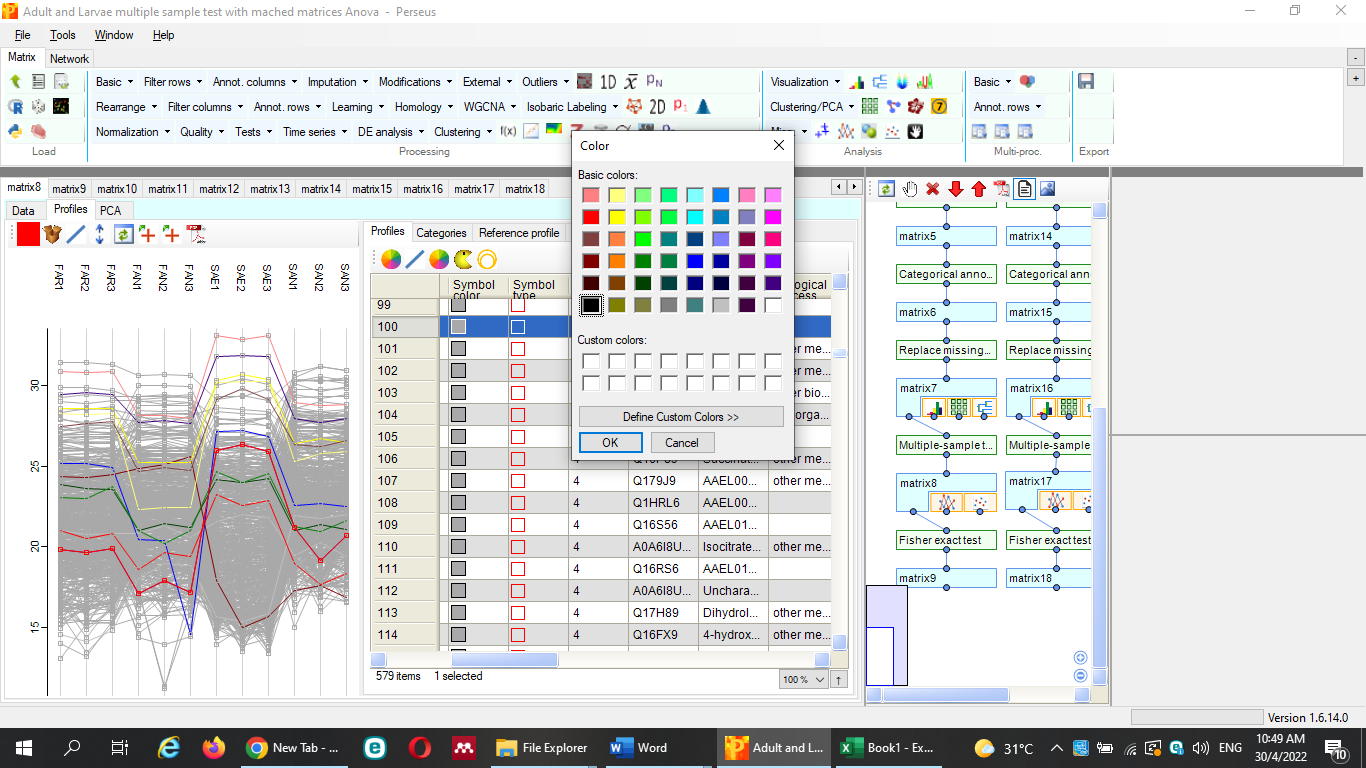 | GST (A0A6I8TLA6) | 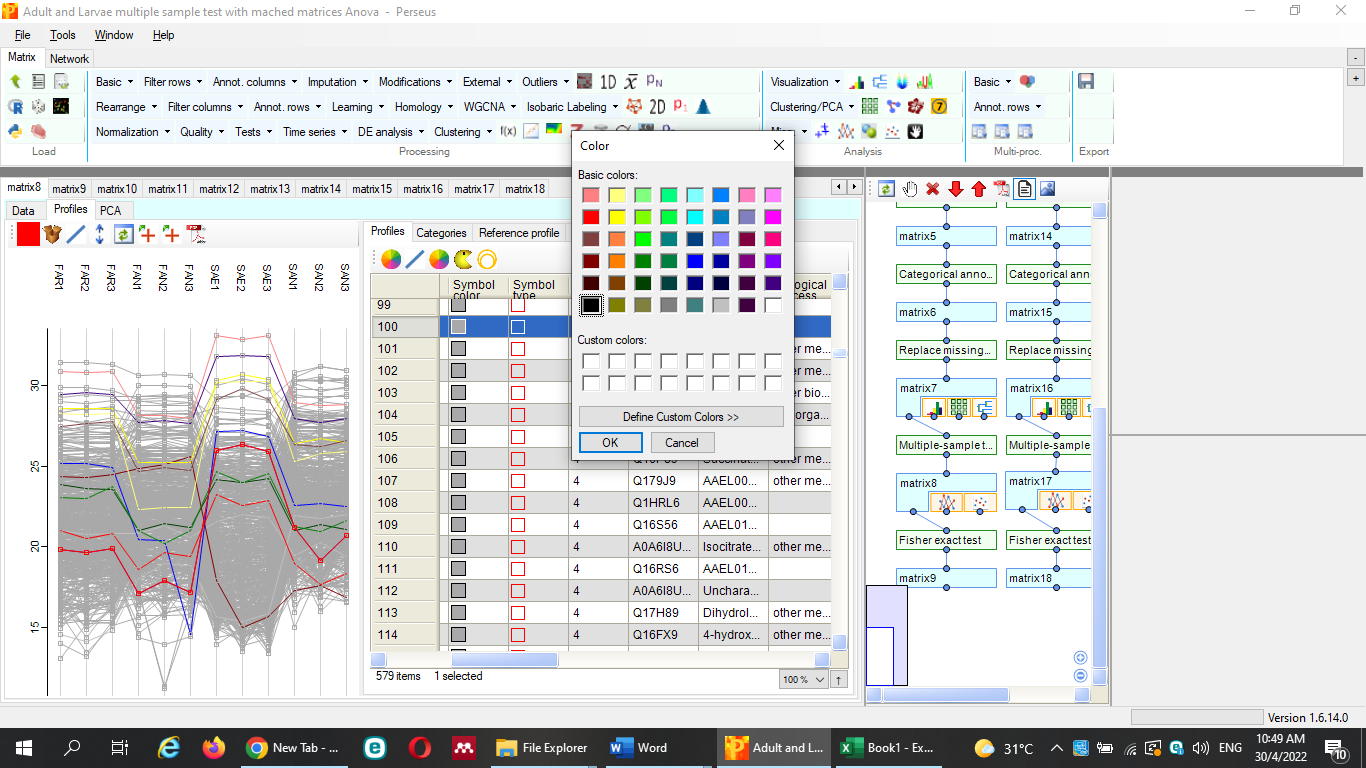 | GST (A0A6I8TL57) |
| 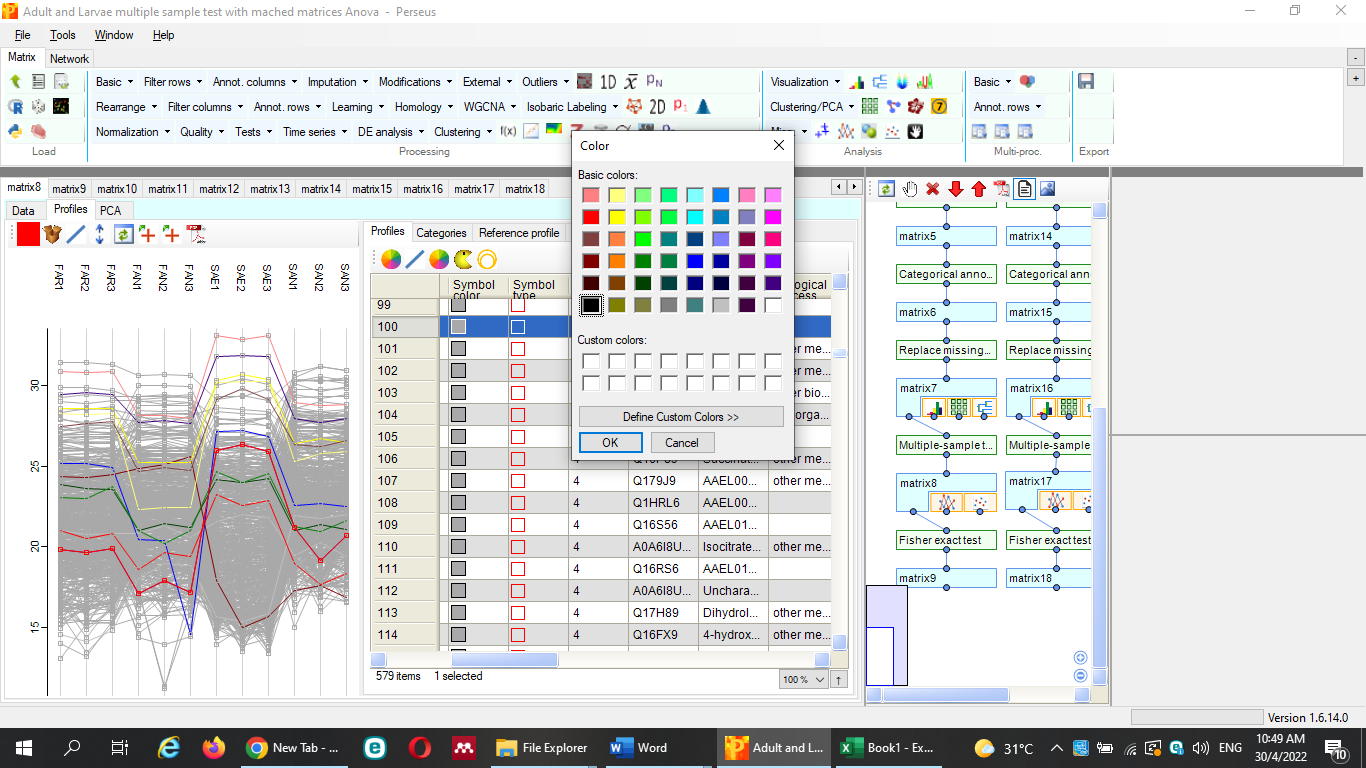 | GSTe4 (Q5PY78) | 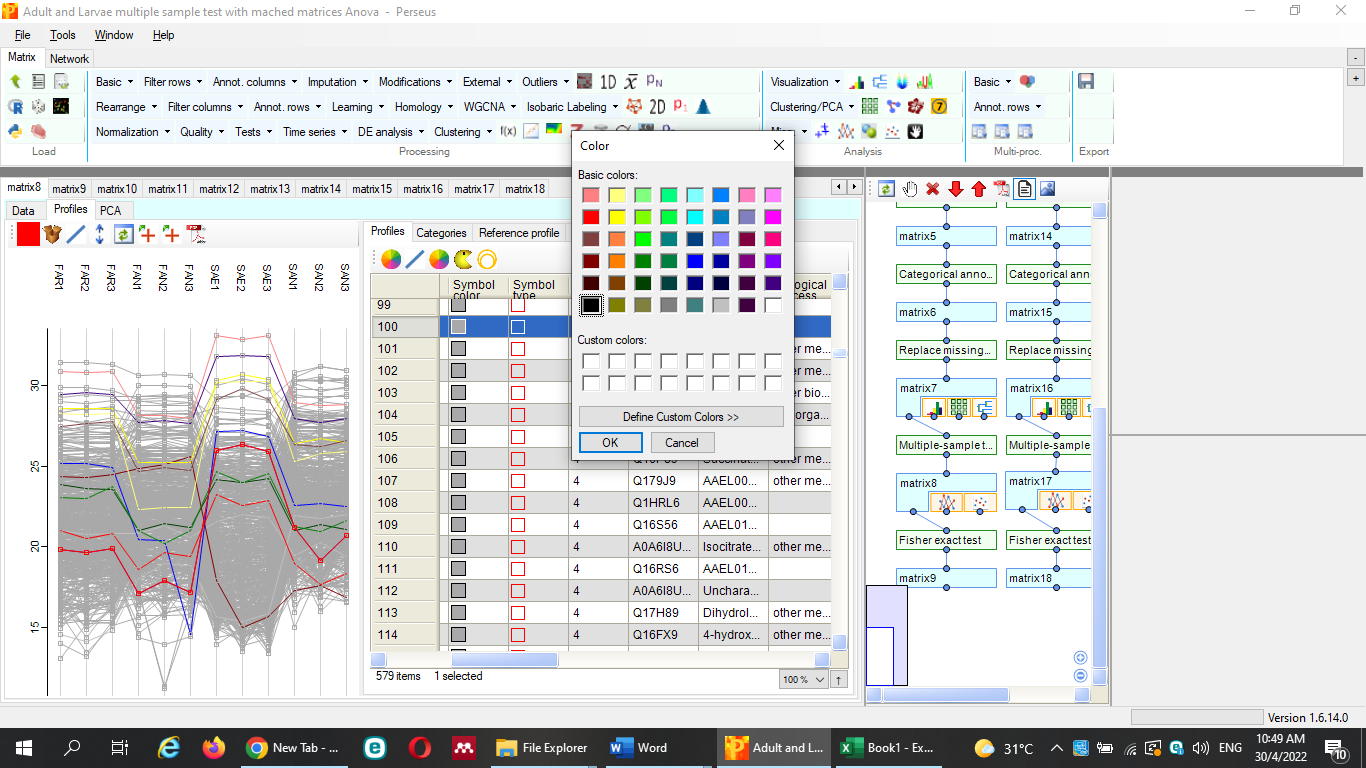 | Glutathione synthetase (Q0IEN8) |


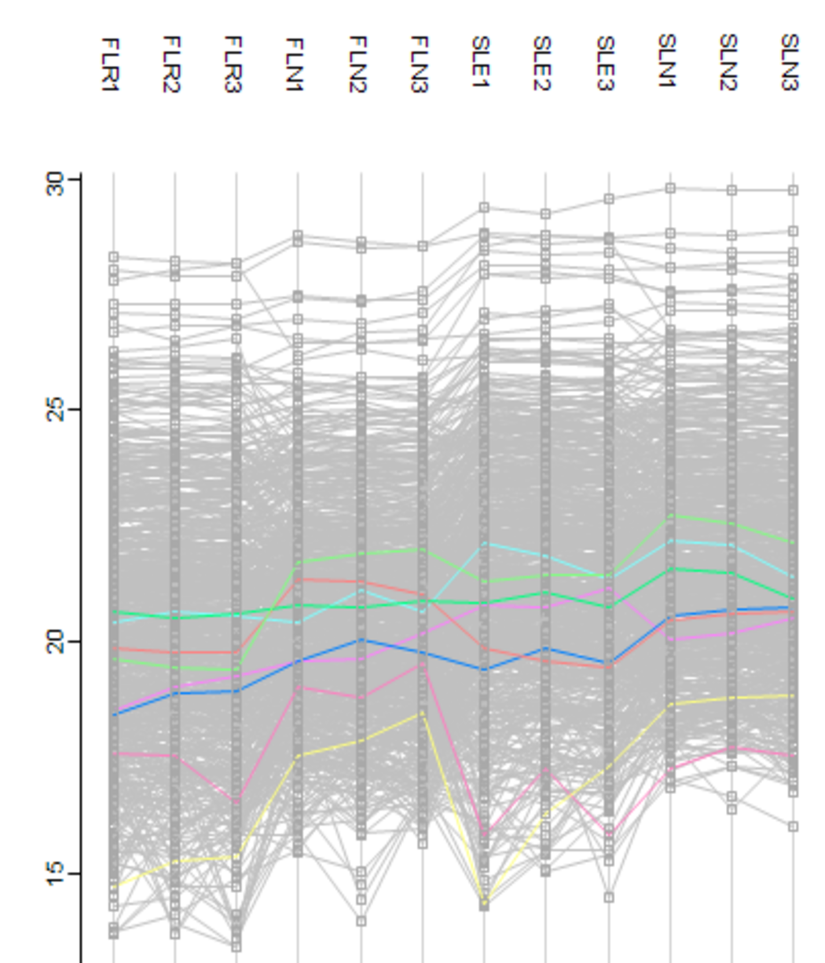


**S Fig D. GST proteins identified by ANOVA in *Ae*. *aegypti* larvae temephos resistant strain (q-value <0.05).** Notes: FLR: Field strain *Ae. aegypti* larvae temephos resistant. FLN: Field strain *Ae. aegypti* larvae not exposed to temephos. SLE: Laboratory strain *Ae. aegypti* larvae exposed to temephos. SLN: Laboratory strain *Ae. aegypti* larvae not exposed to temephos.

**Notes:**

| 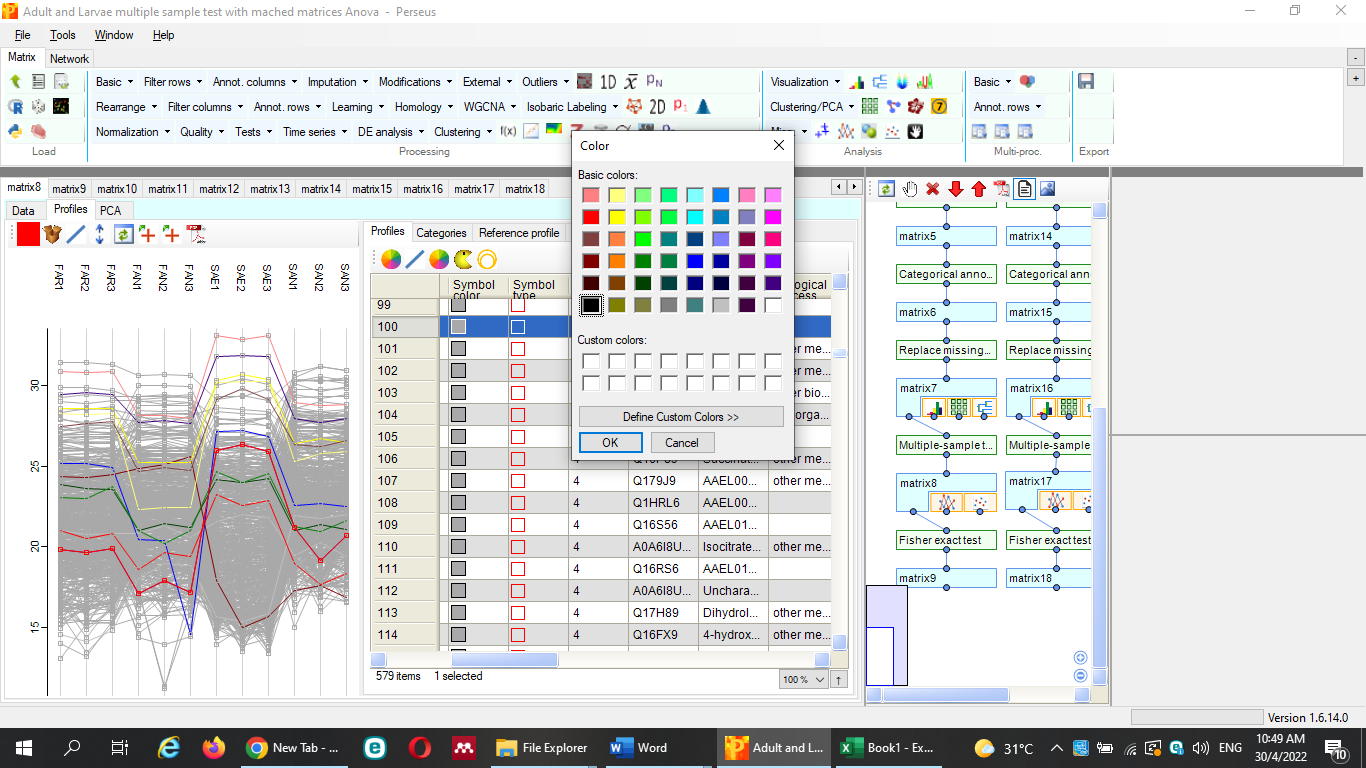 | GSTD1 (J9HHL7) | 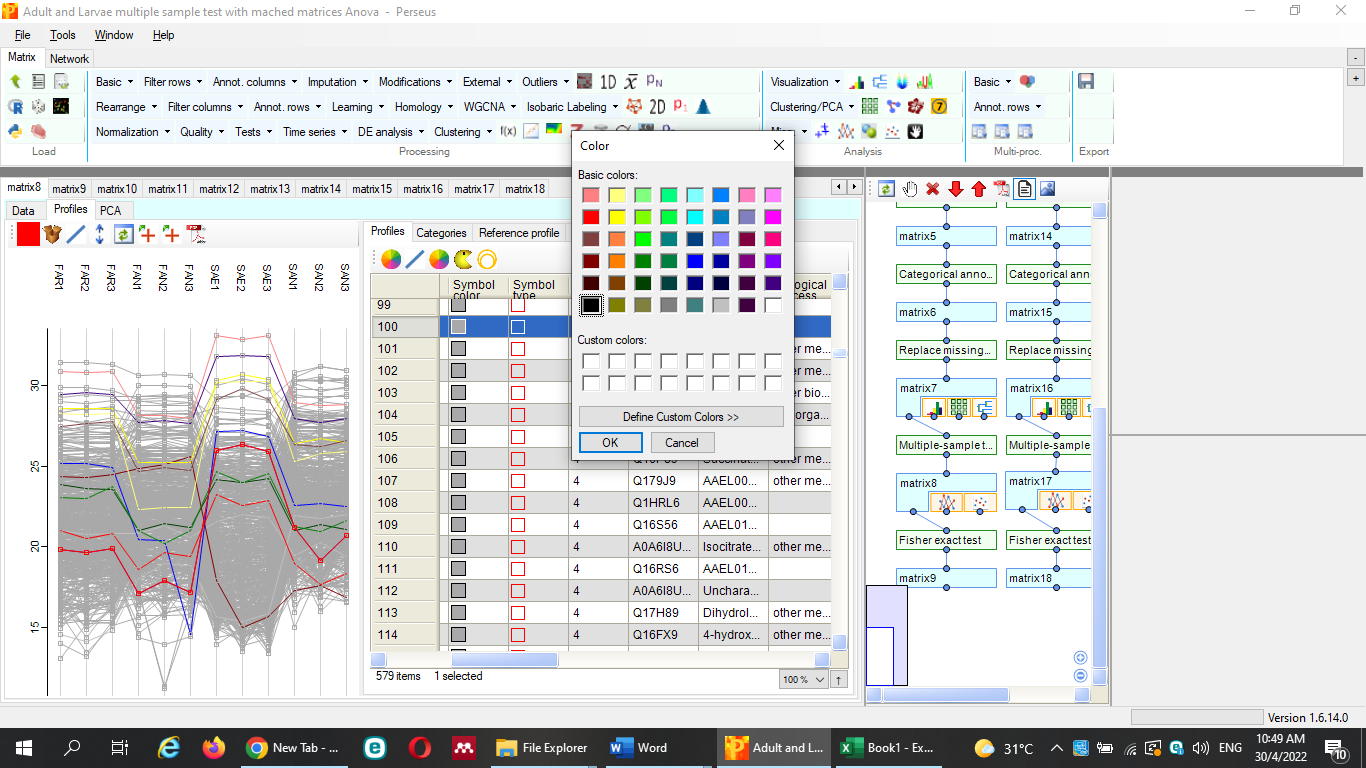 | GSTD1 (A0A1S4EXN8) |
| --- | --- | --- | --- |
| 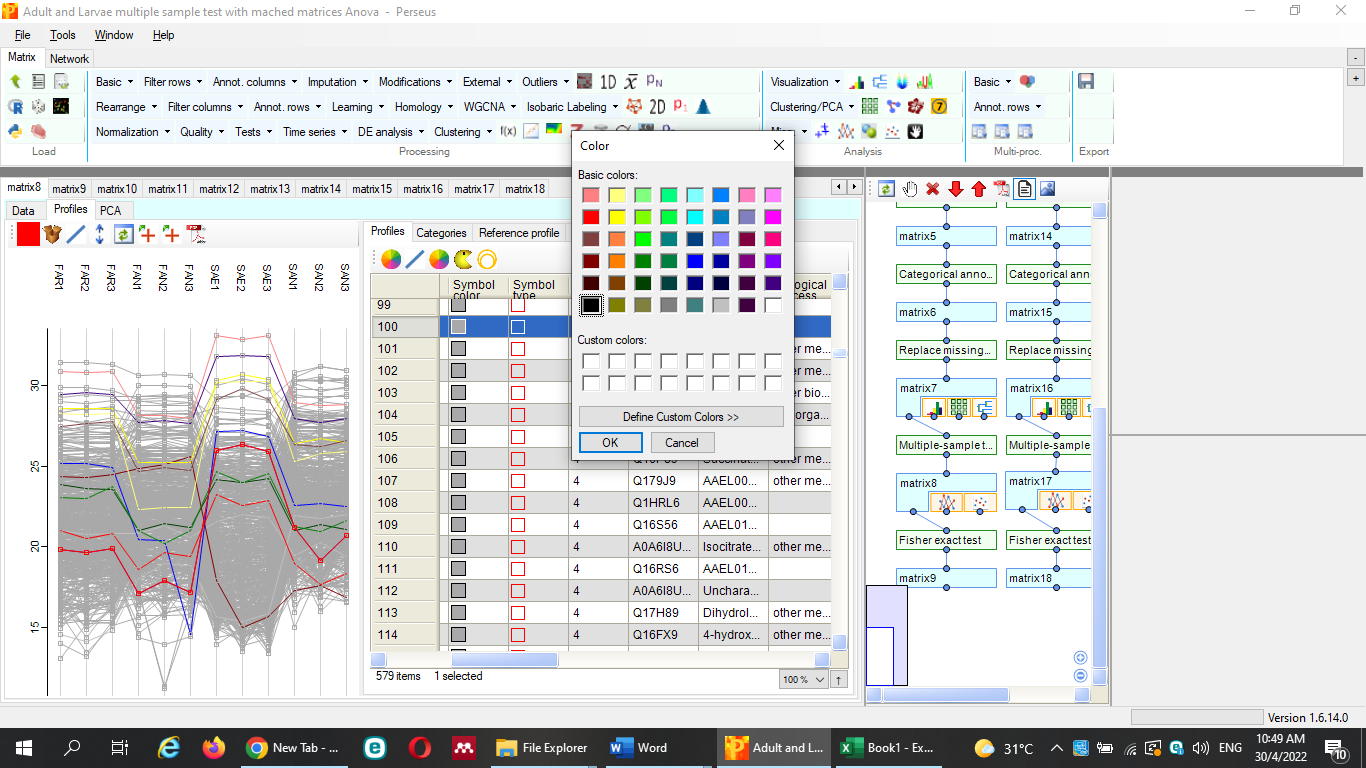 | GSTS1 (Q16P79) | 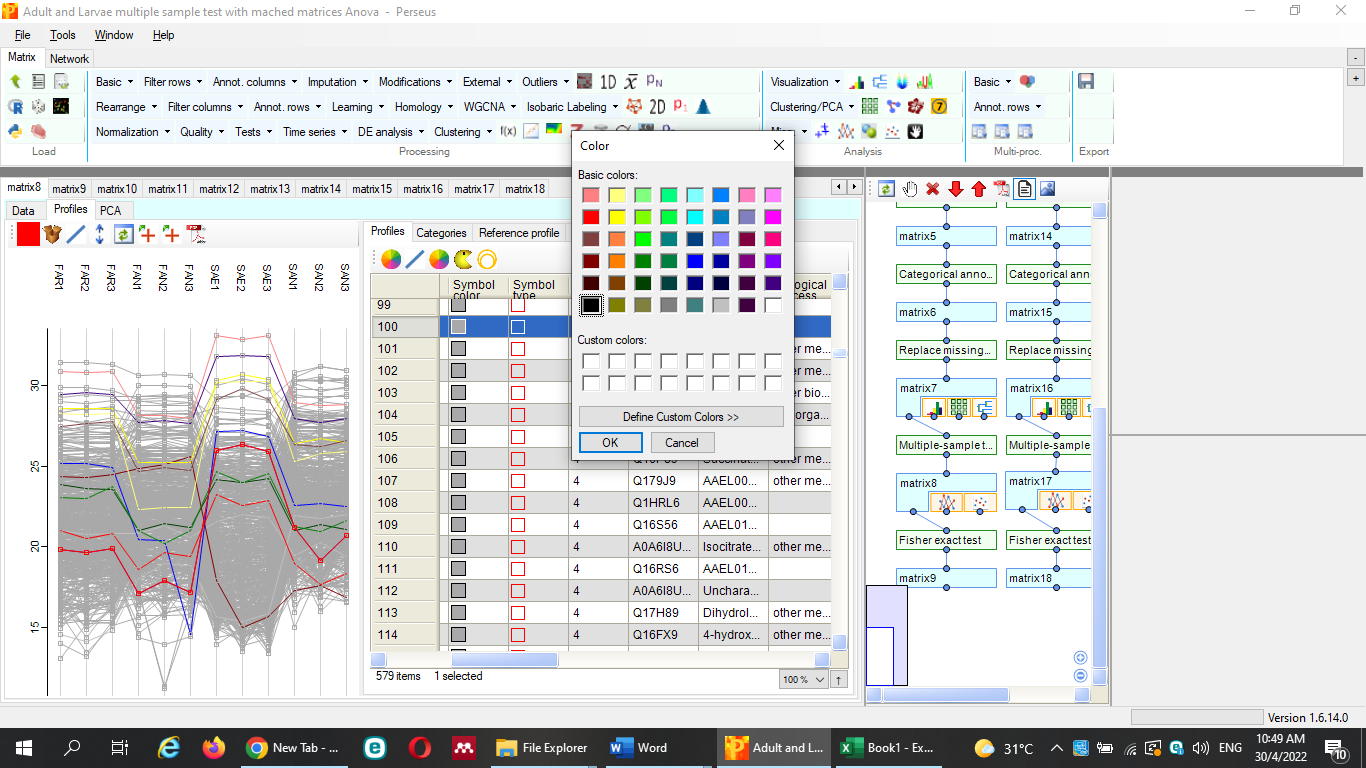 | Glutathione peroxidase (A0A6I8TLR0) |
| 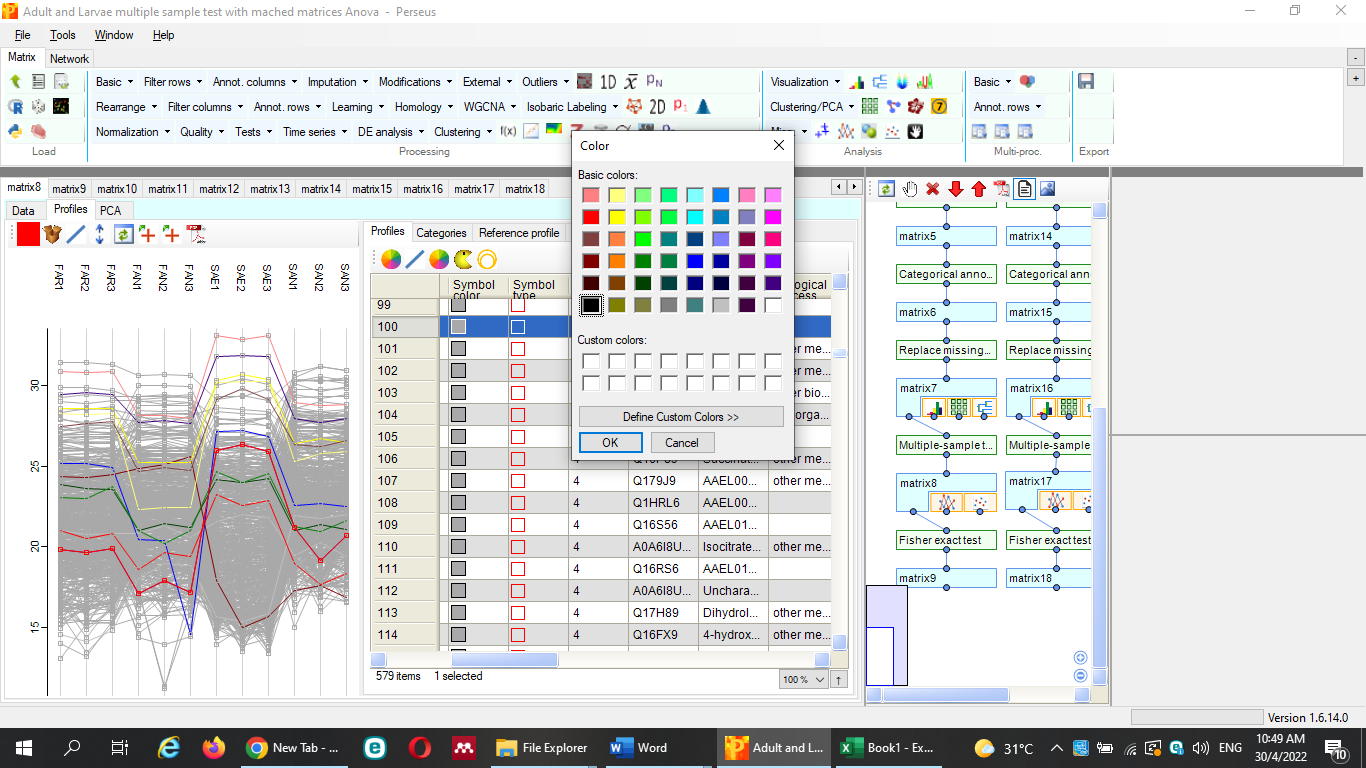 | S-glutathione dehydrogenase (Q176A6) | 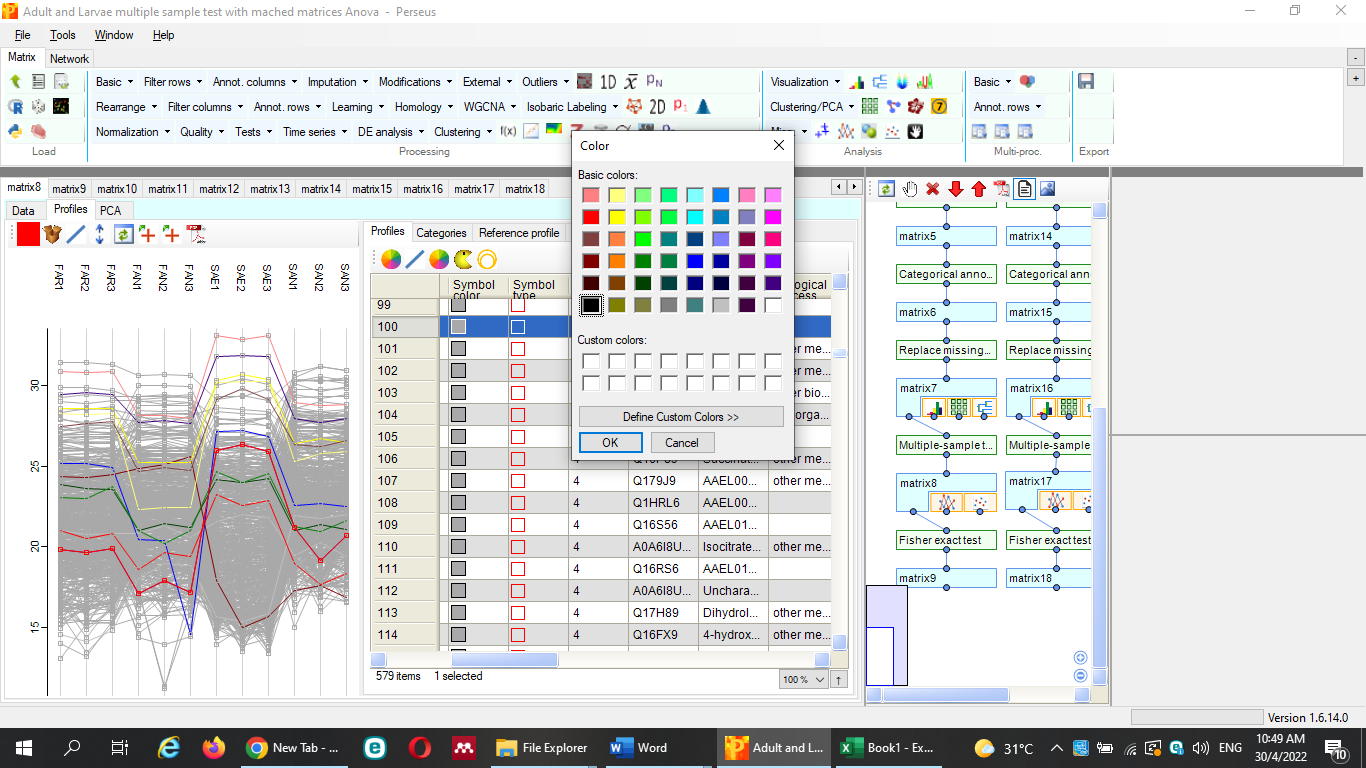 | GSTD4(Q17MB8) |
| 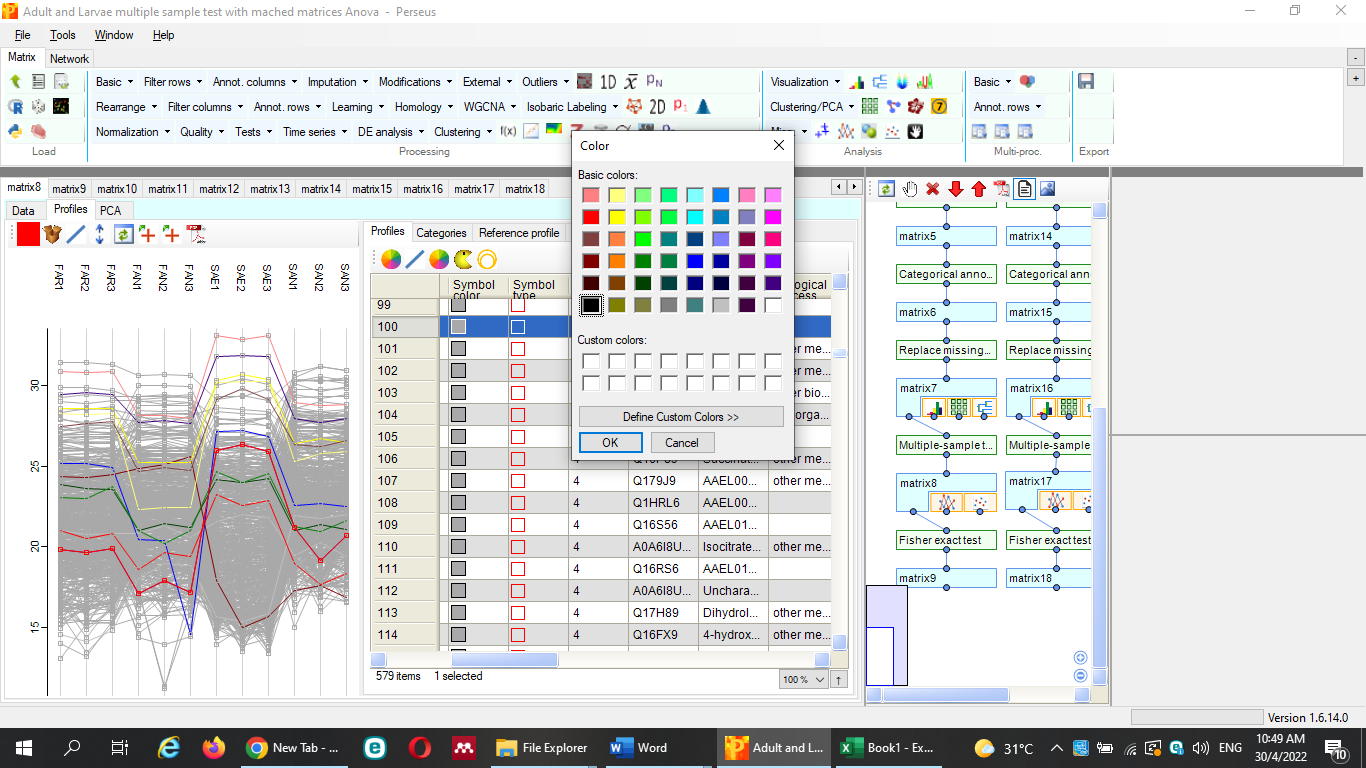 | GSTd4 (Q0C791) | 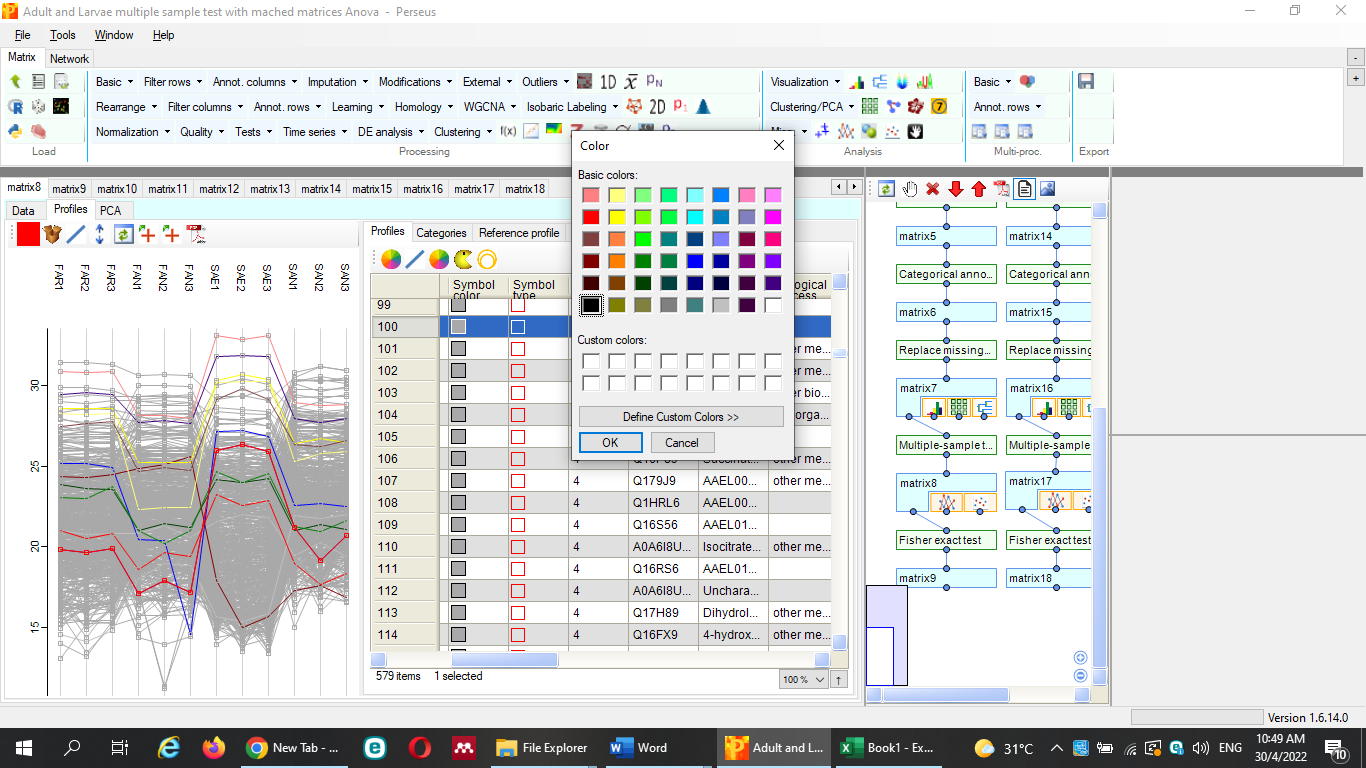 | GSTD11 (Q16SH7) |


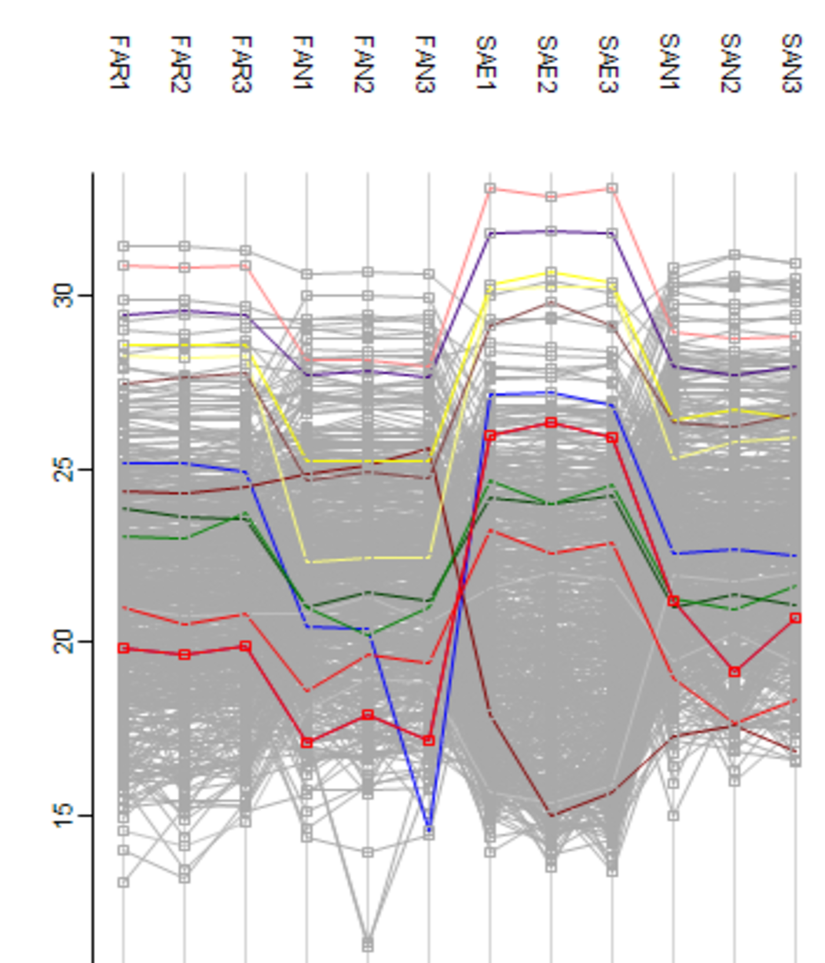


**S Fig E. Motor proteins identified by ANOVA in adult *Ae*. *aegypti* permethrin-resistant strain (q-value <0.05).** Notes: FAR: Field strain adult *Ae. aegypti* permethrin resistant. FAN: Field strain adult *Ae. aegypti* not exposed to permethrin. SAE: Laboratory strain adult *Ae. aegypti* exposed to permethrin. SAN: Laboratory strain adult *Ae. aegypti* not exposed to permethrin.

**Notes:**

| 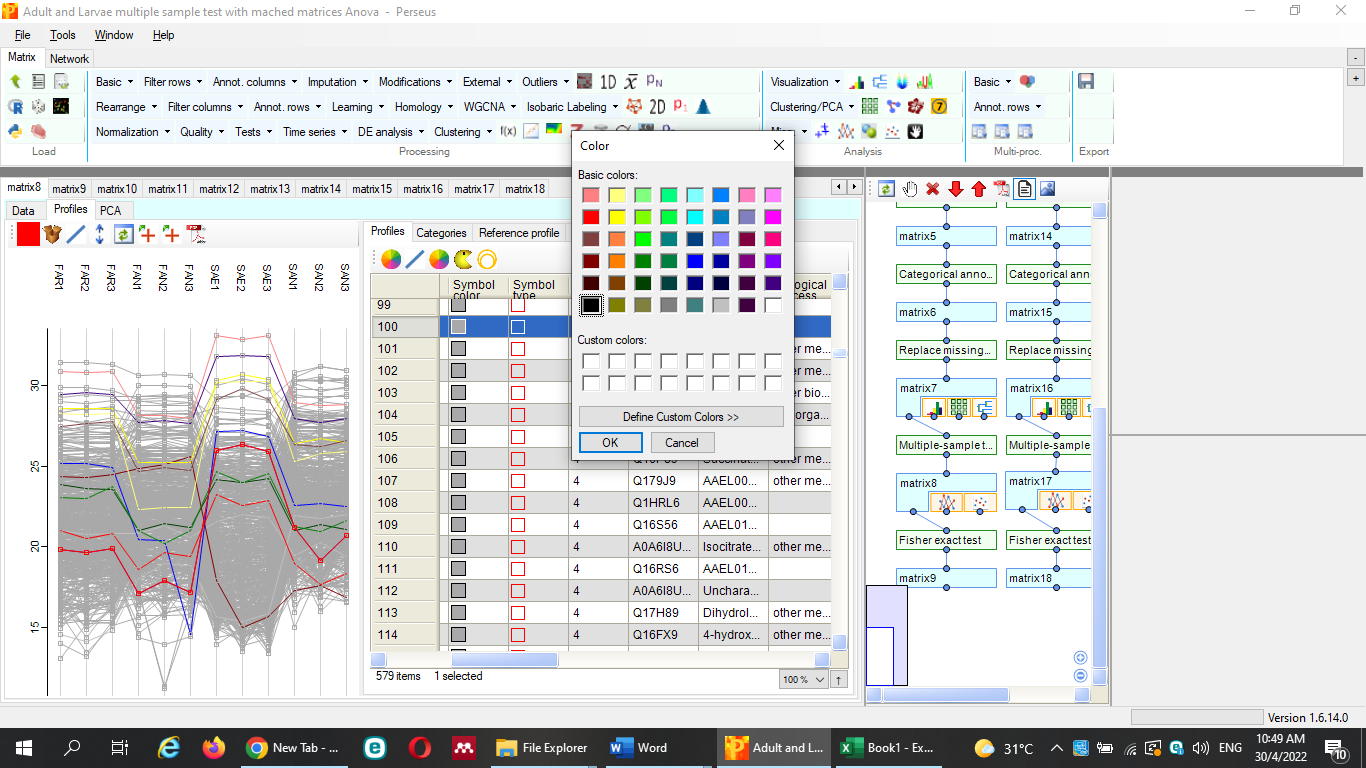 | Myosin heavy chain, non-muscle (Q179E8) | 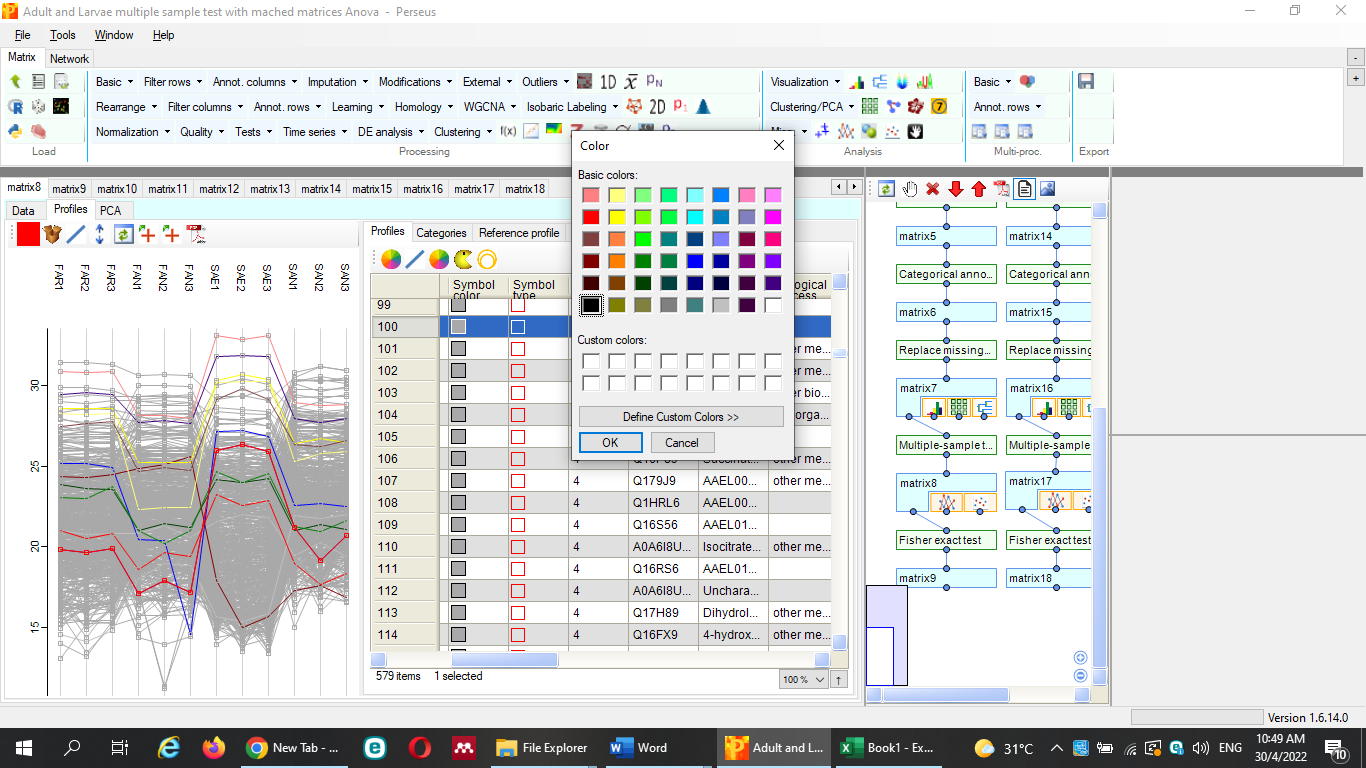 | Myosin heavy chain(W0FUL2) |
| --- | --- | --- | --- |
| 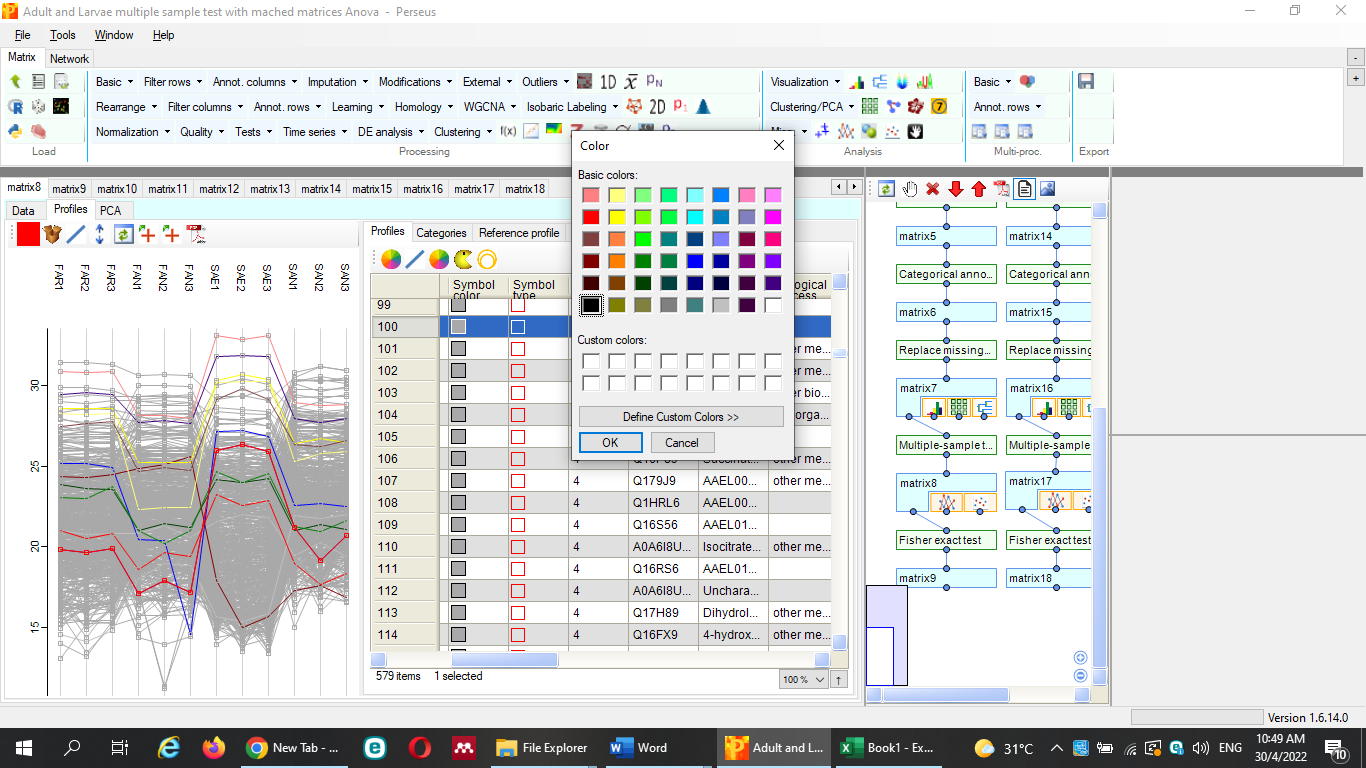 | Myosin regulatory light chain 2 (Q17HX1) | 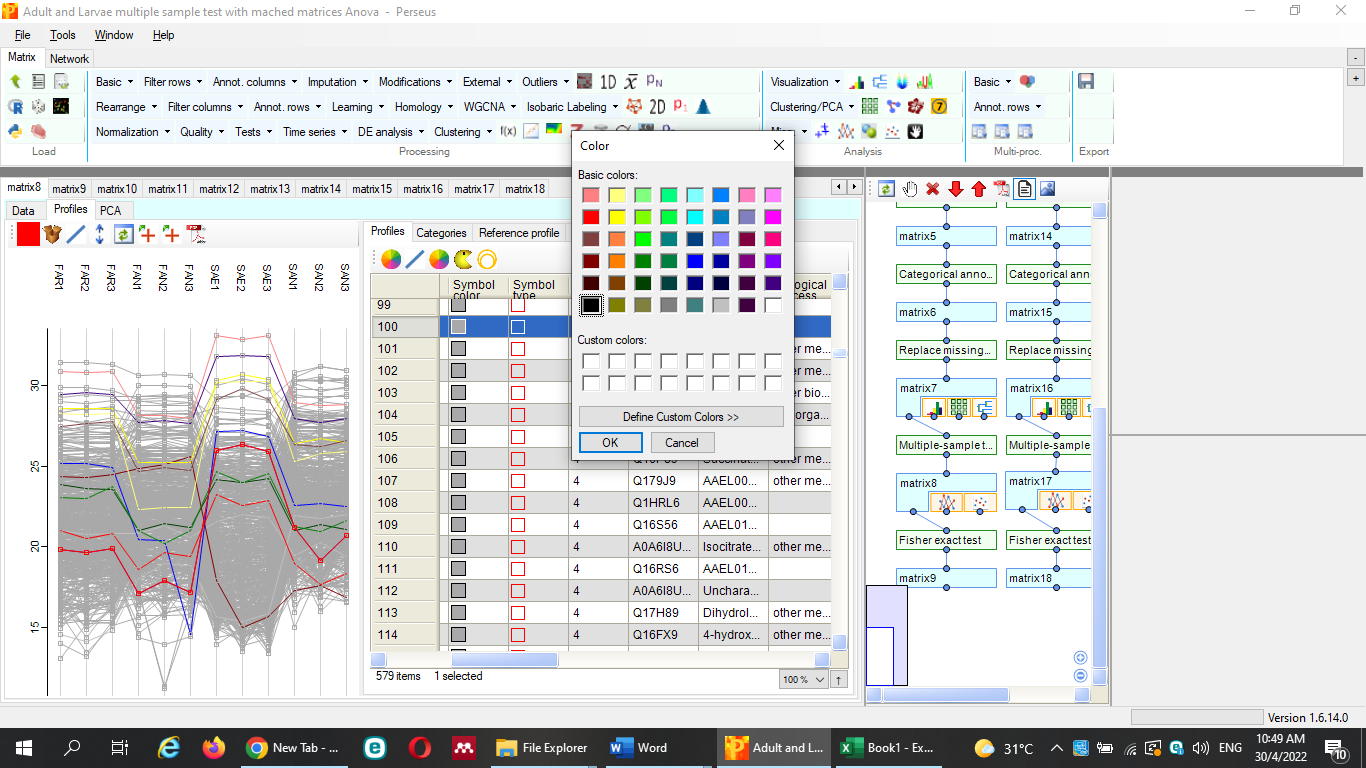 | Myosin light chain alkali (Q16MS5) |
| 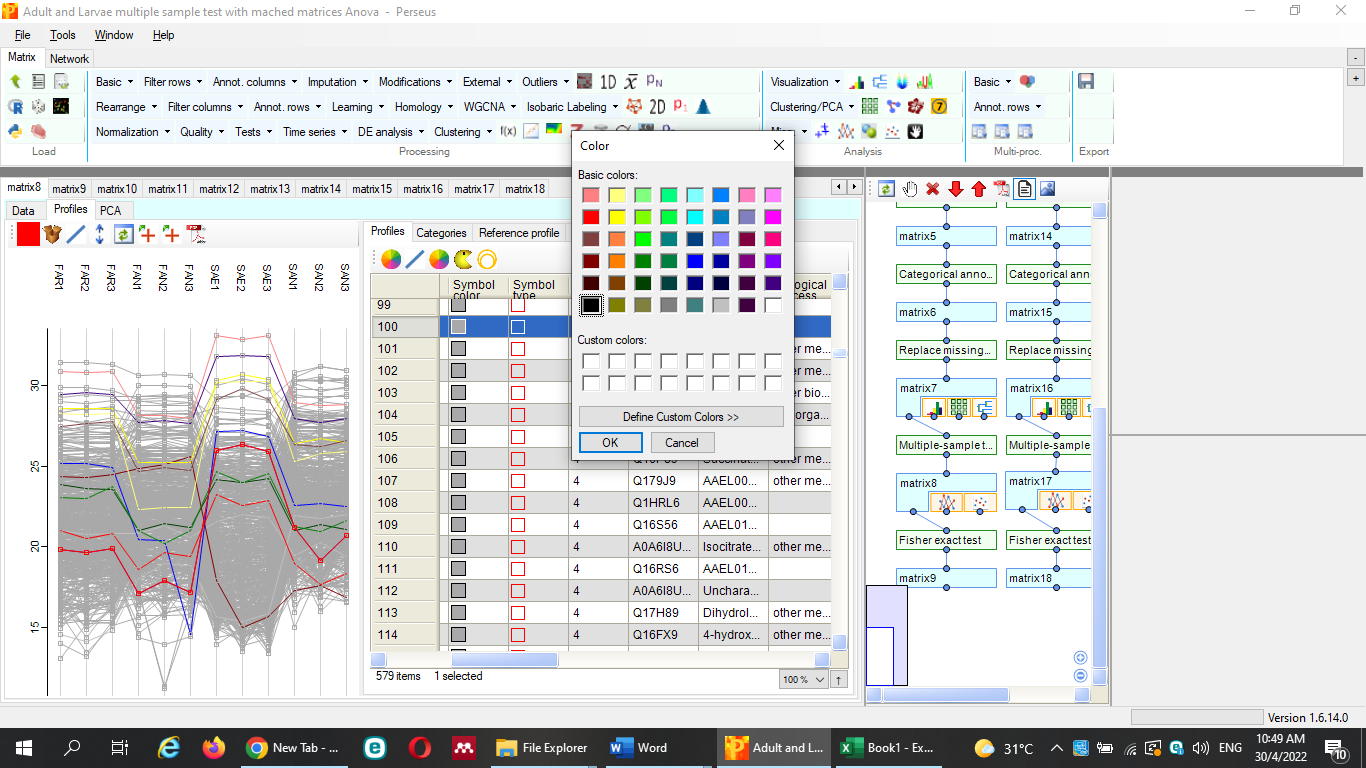 | Troponin t, invertebrate (Q17I94) | 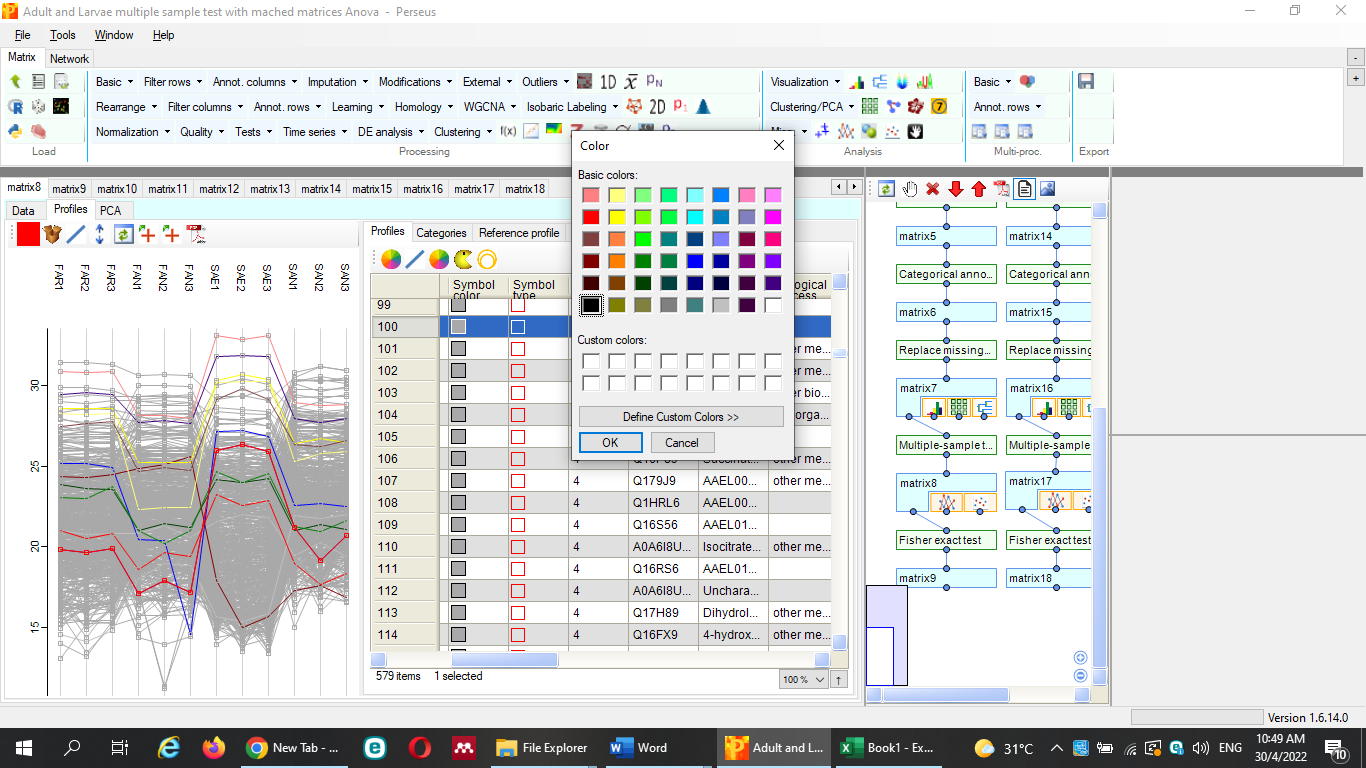 | Troponin I (Q16RS5) |
| 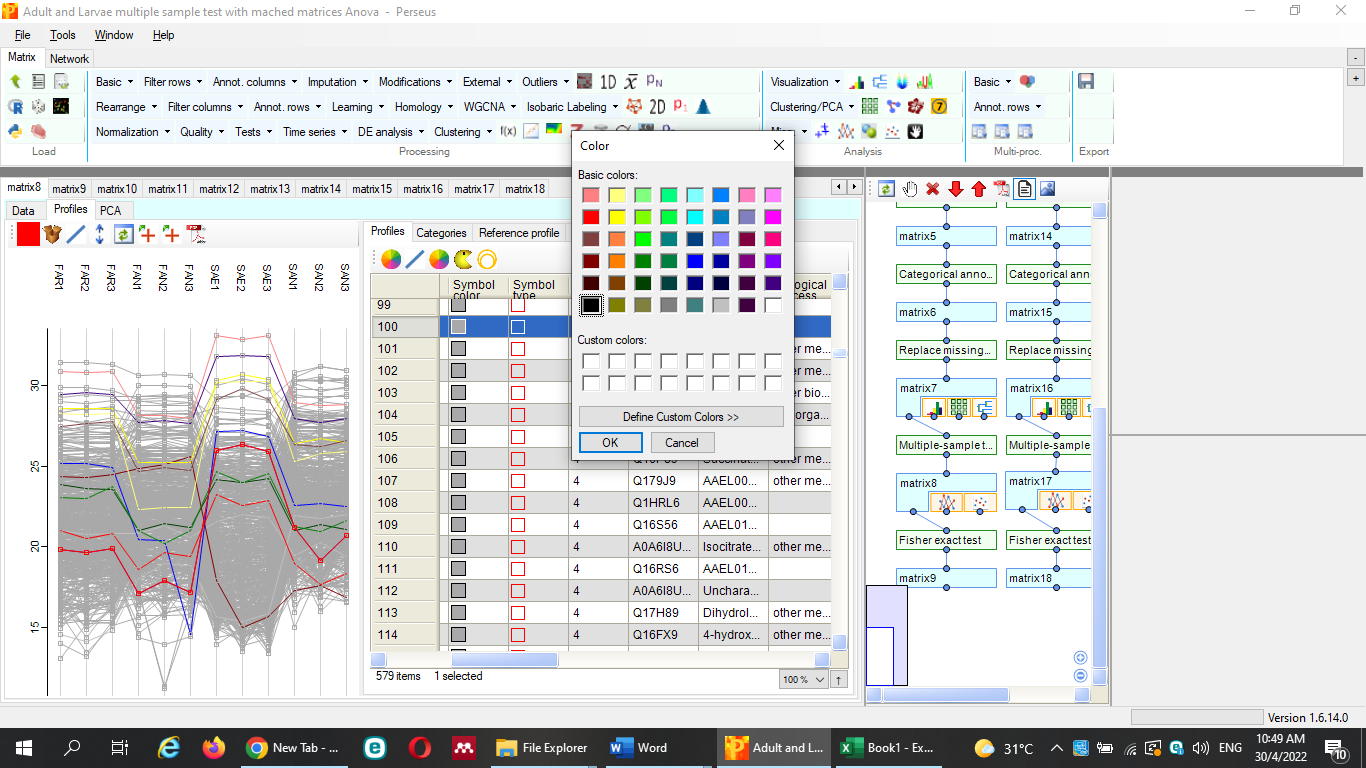 | Troponin I (Q16RS6) | 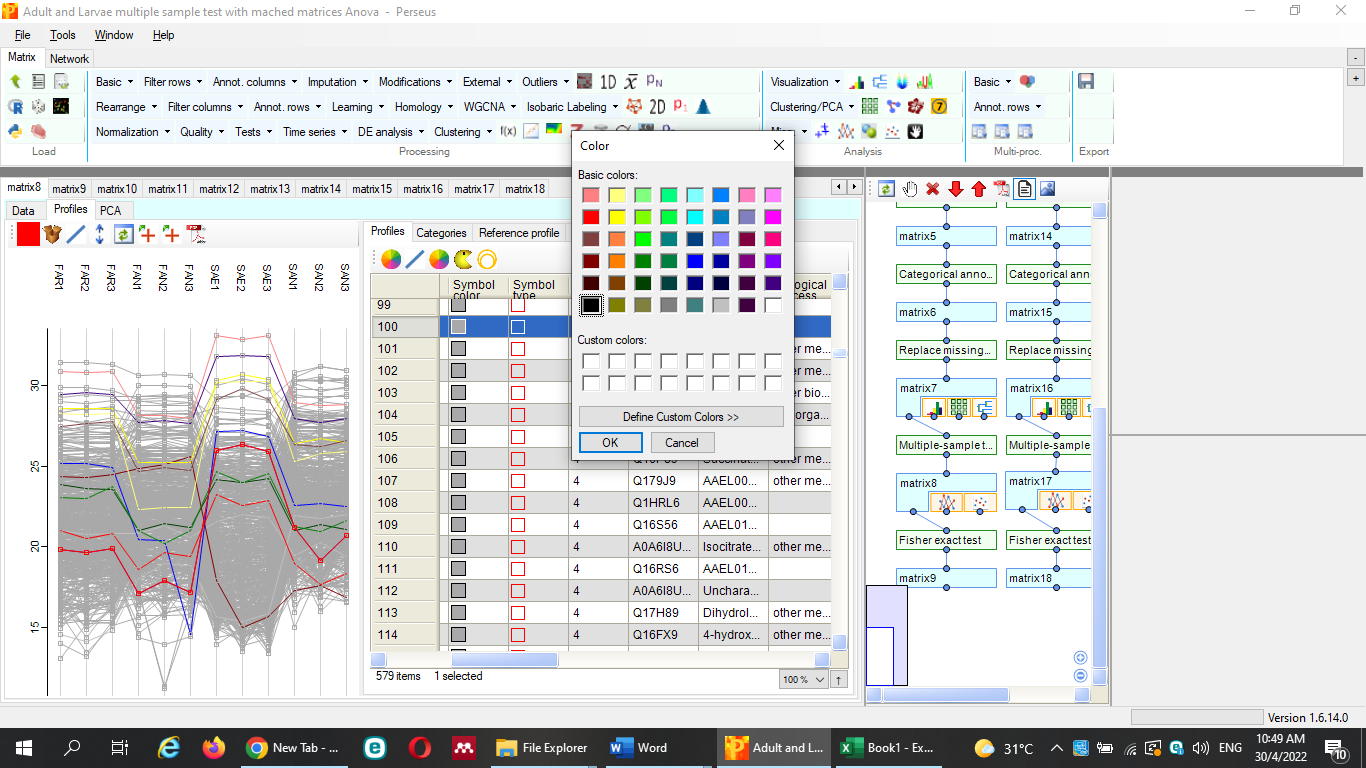 | Troponin I (Q16RS8) |
| 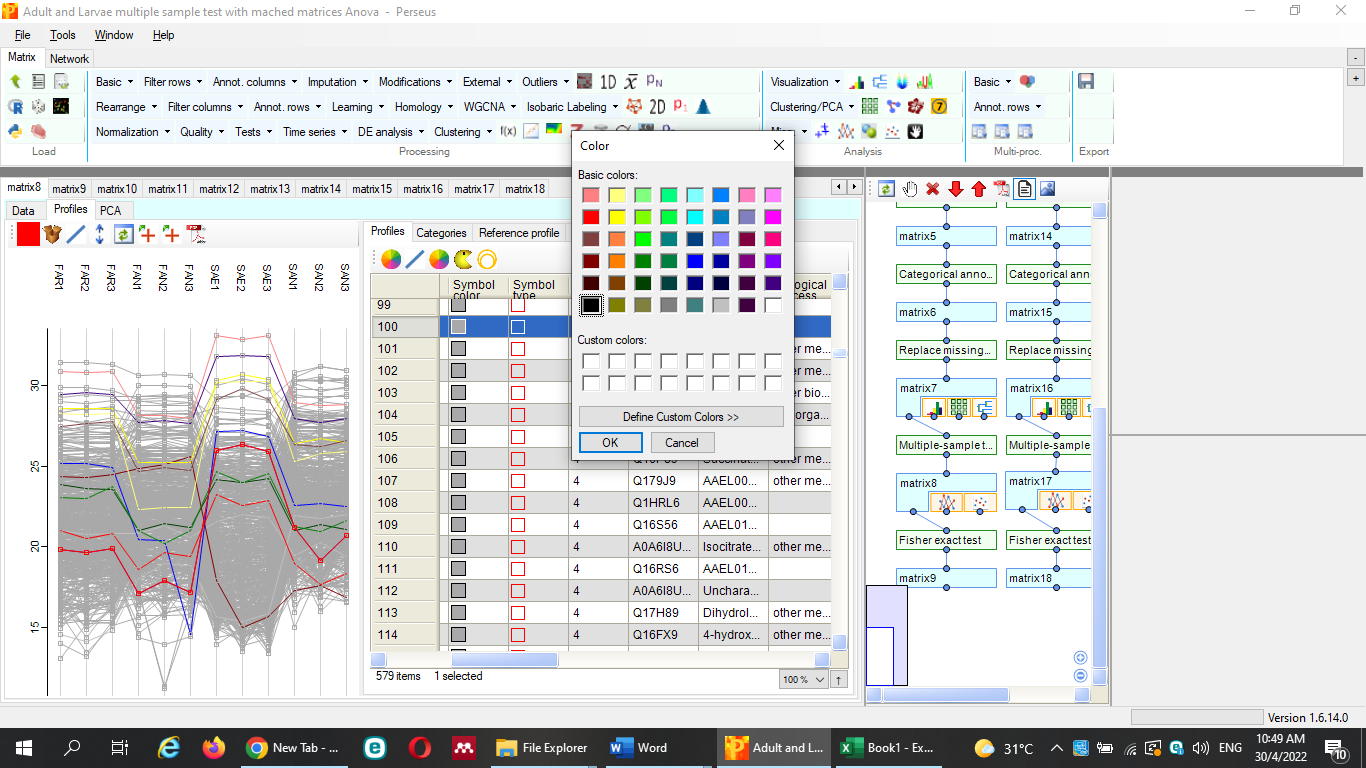 | Troponin I (Q16RS3) | 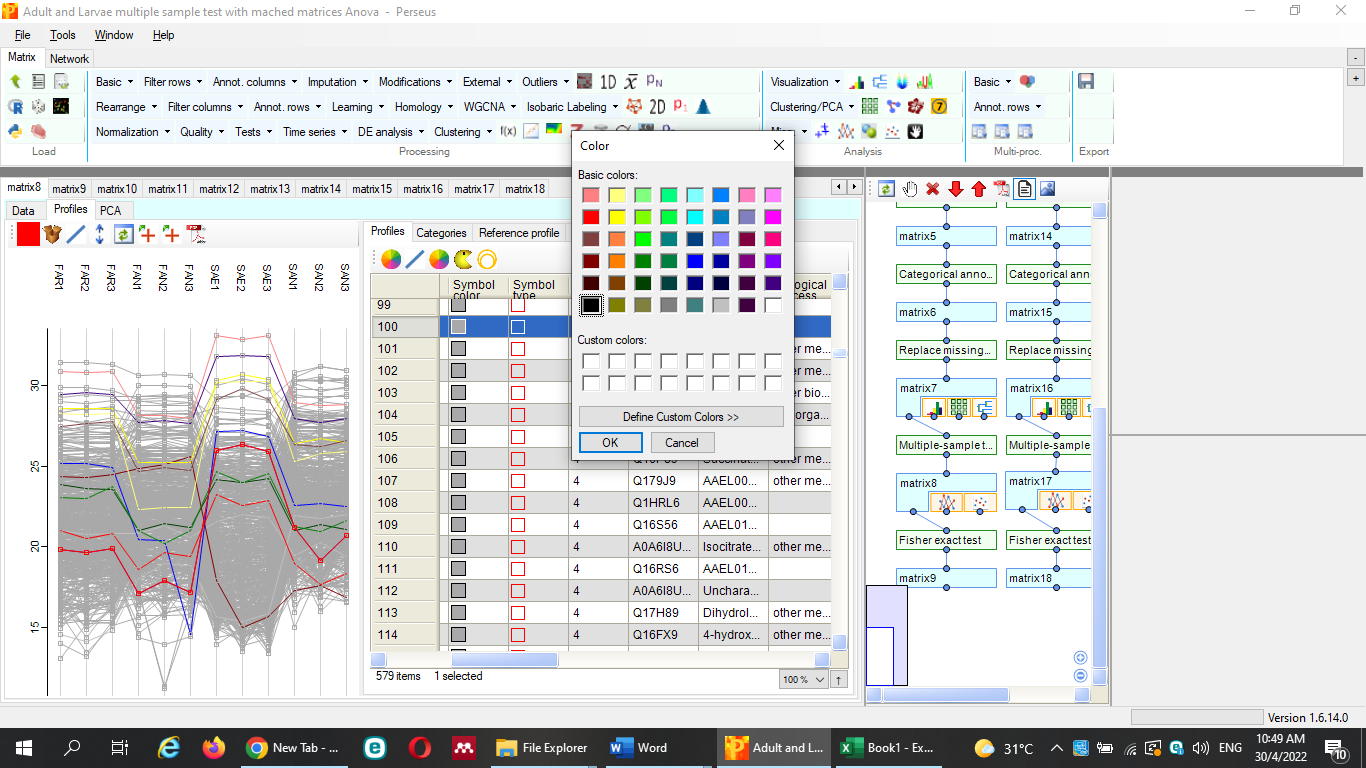 | Tropomyosin invertebrate (Q17H80) |
| 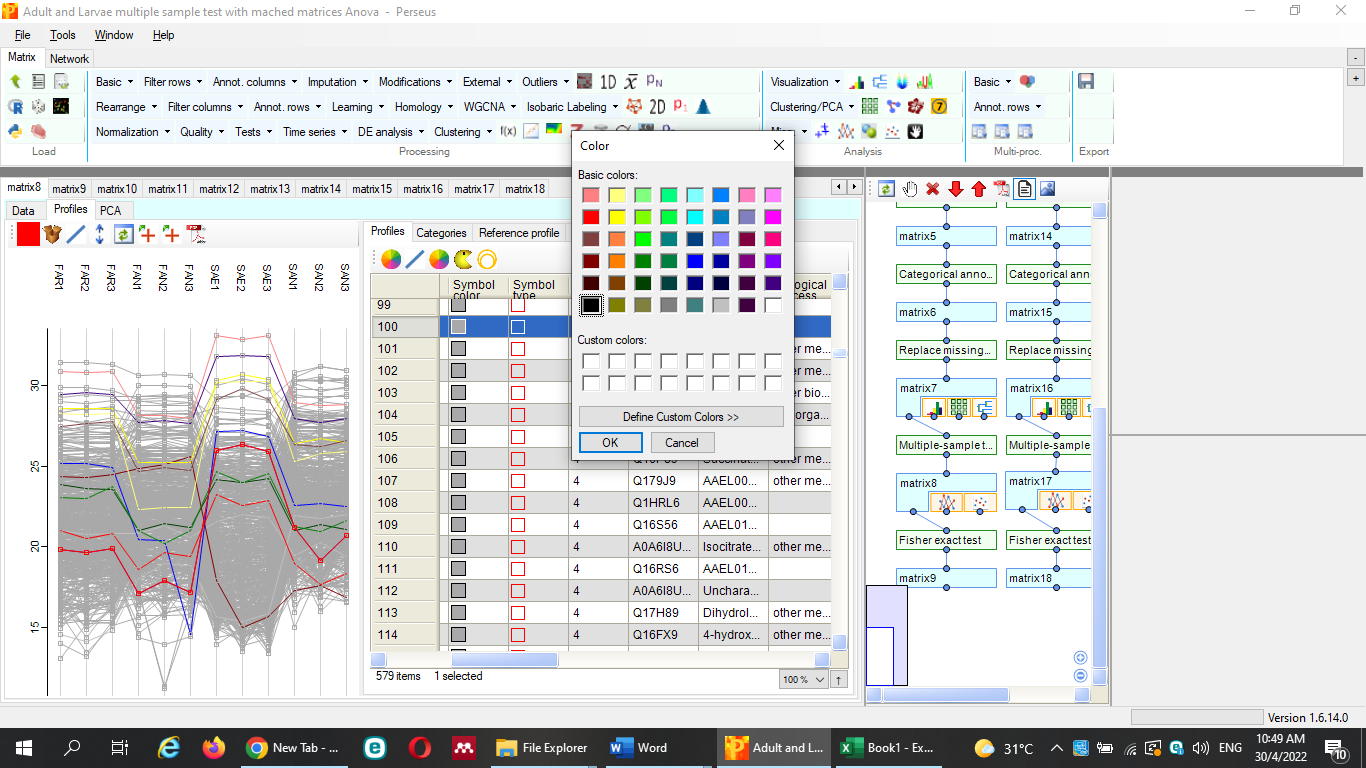 | Tropomyosin invertebrate(Q17H74) |  |  |


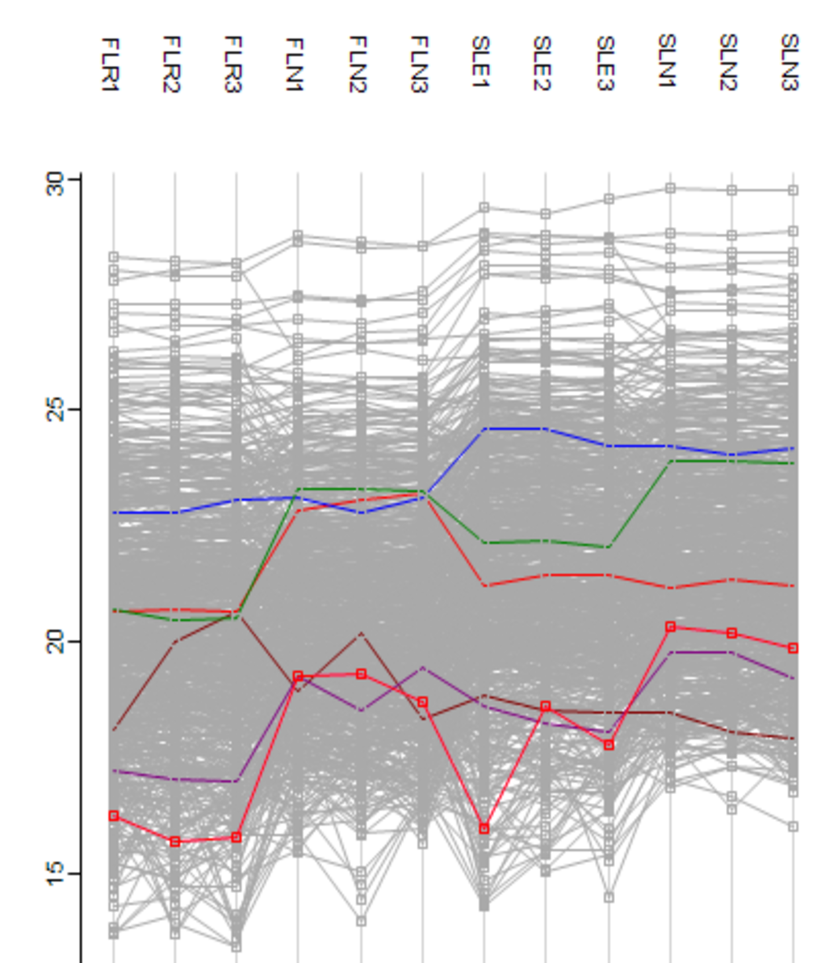


**S Fig F. Motor proteins identified by ANOVA in *Ae*. *aegypti* larvae temephos resistant strain (q-value <0.05).** Notes: FLR: Field strain *Ae. aegypti* larvae temephos resistant. FLN: Field strain *Ae. aegypti* larvae not exposed to temephos. SLE: Laboratory strain *Ae. aegypti* larvae exposed to temephos. SLN: Laboratory strain *Ae. aegypti* larvae not exposed to temephos.

**Notes:**

| 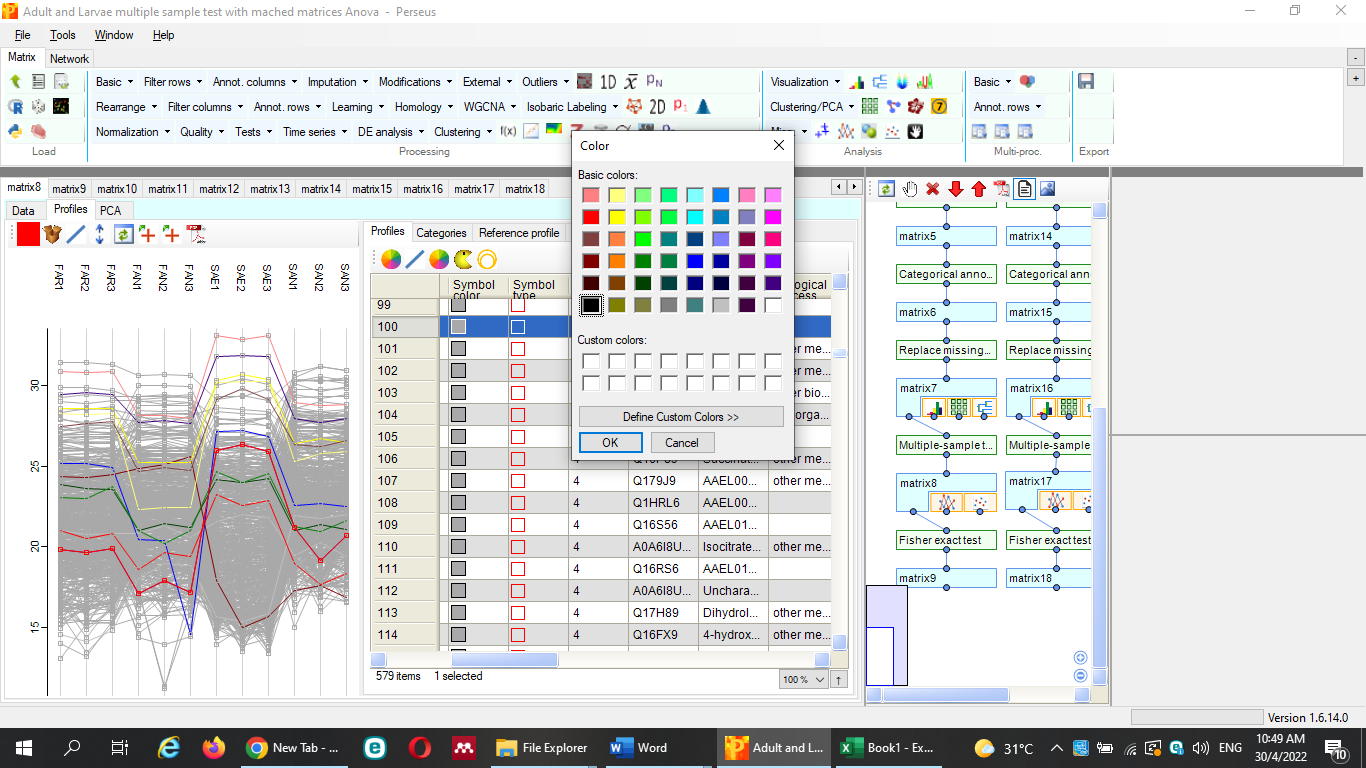 | Troponin t, invertebrate (A0A6I8T582) | 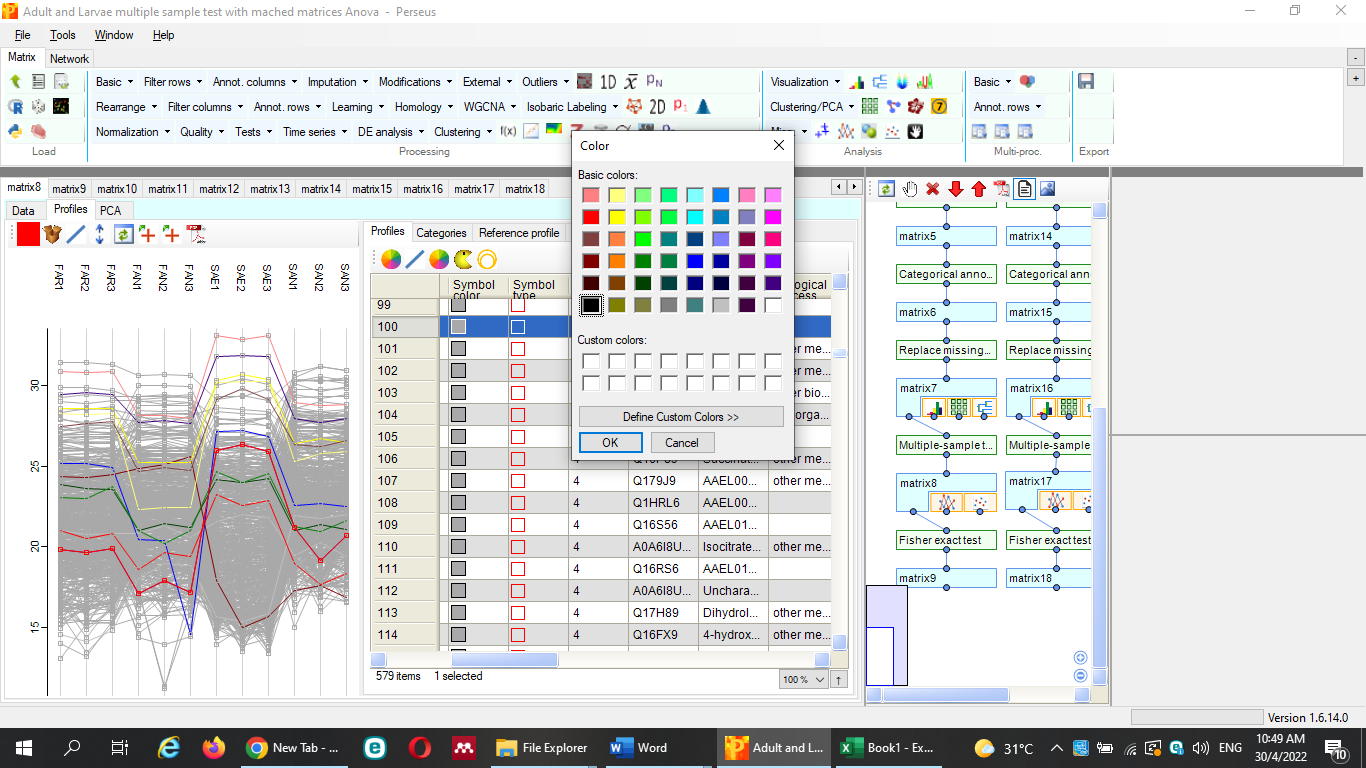 | Troponin I (Q16RS5) |
| --- | --- | --- | --- |
| 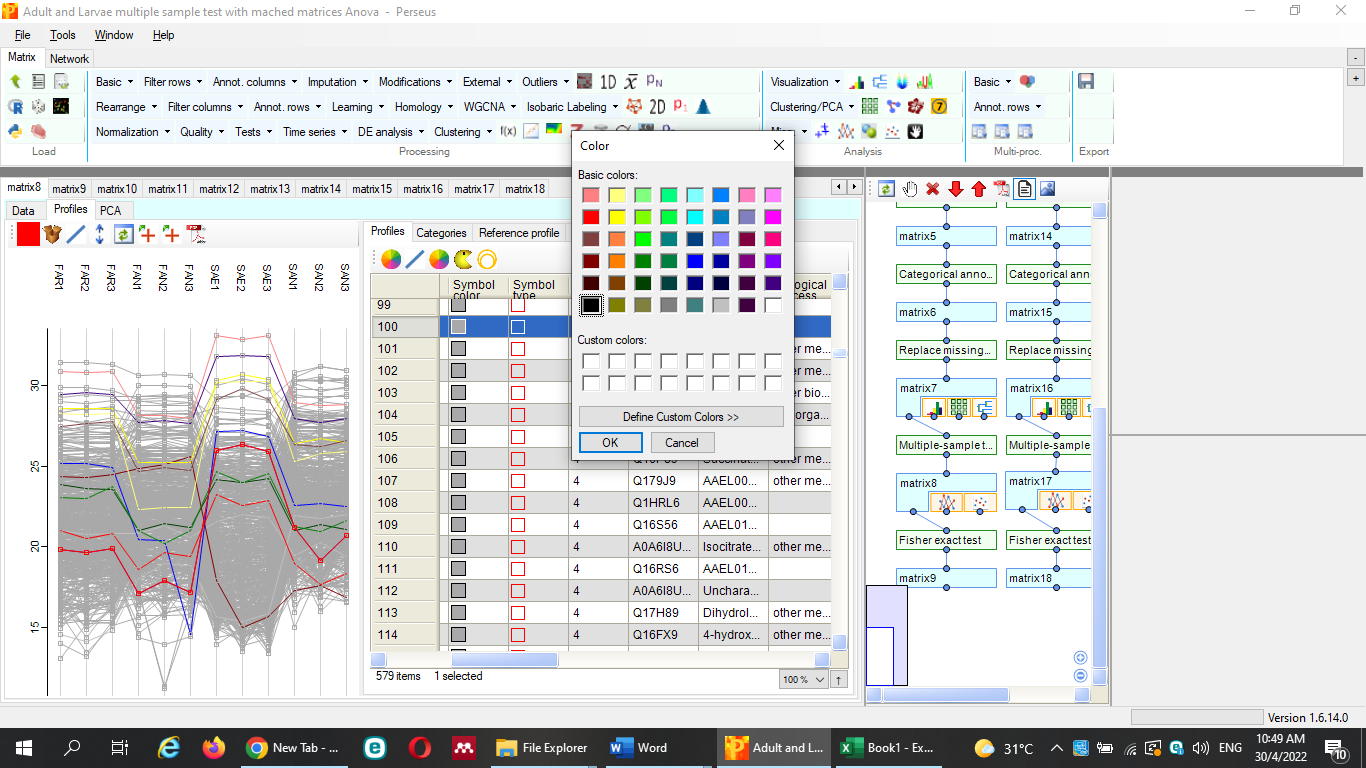 | Paramyosin, long-form(Q16RF4) | 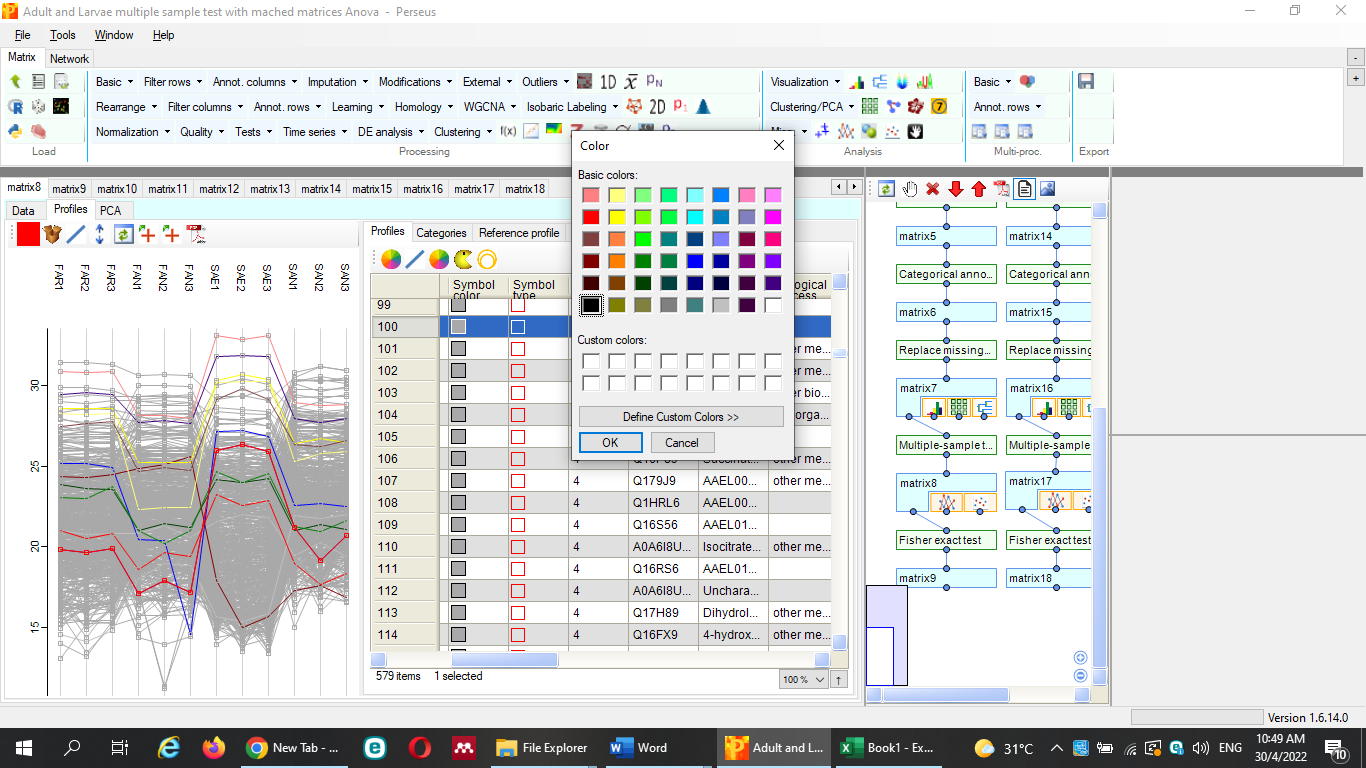 | Myosin regulatory light chain 2 (mlc-2)( Q17HX1) |
| 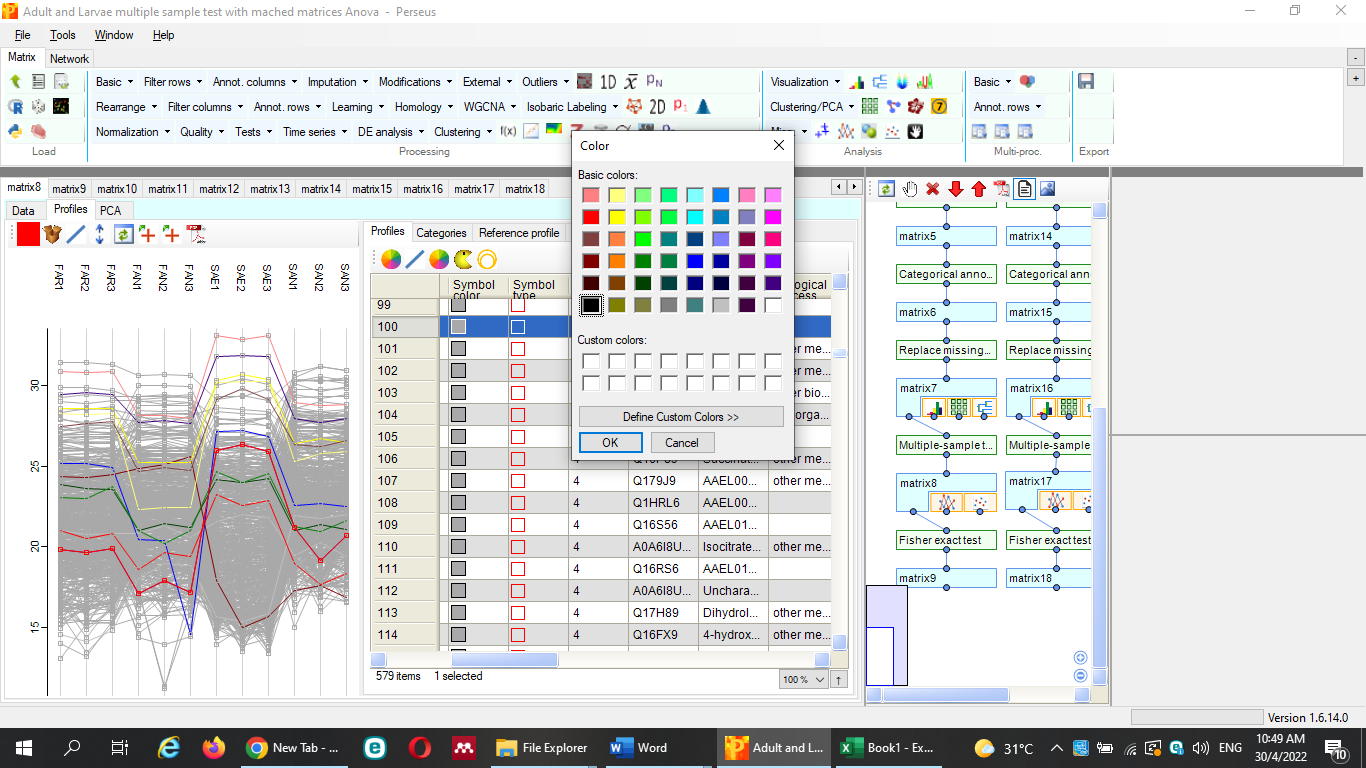 | Paramyosin, long form (A0A6I8TKE6) | 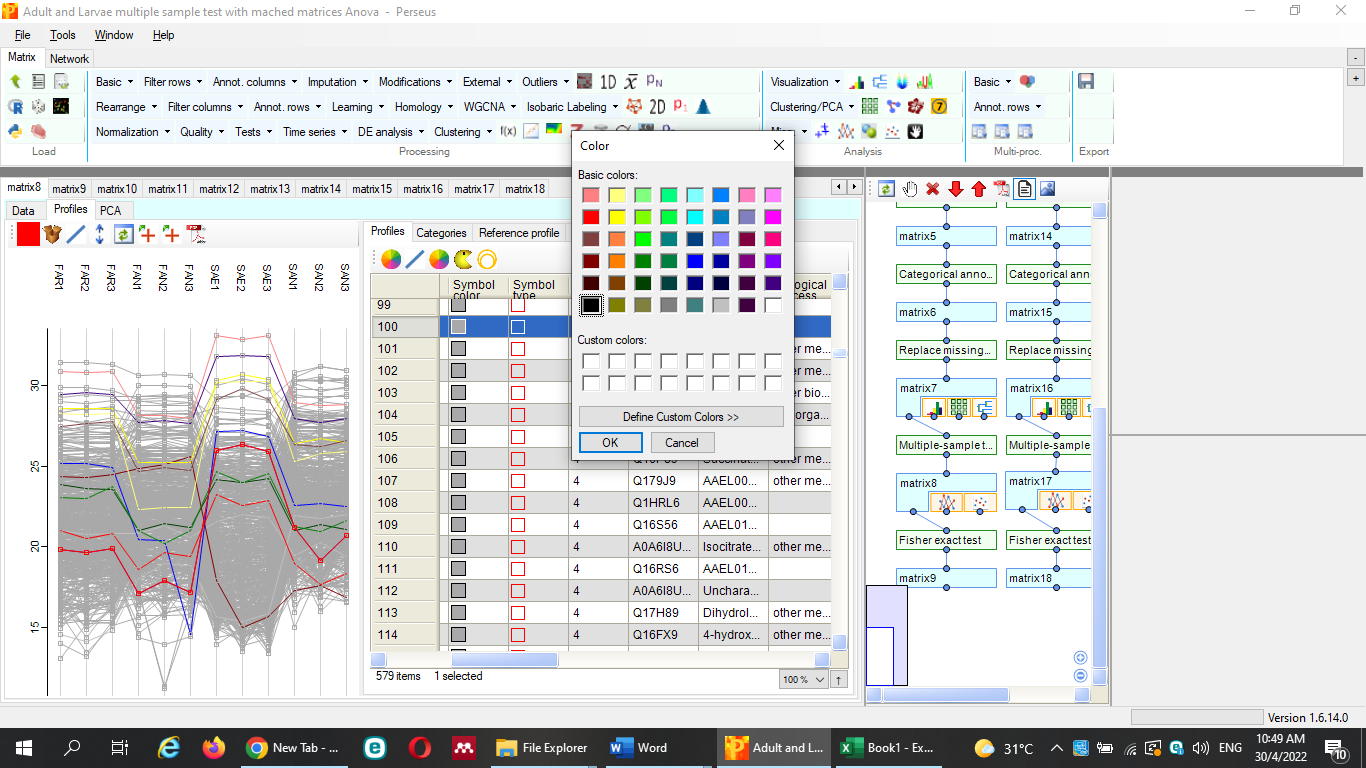 | Myosin light chain alkali (Q16MS5) |


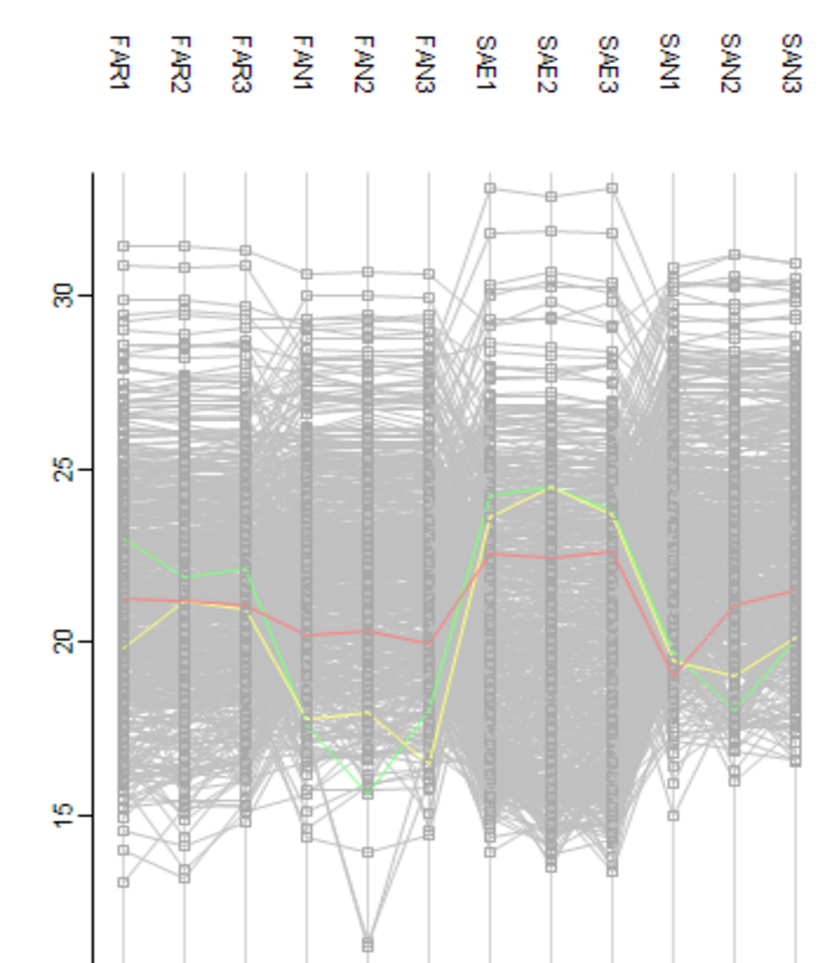


**S Fig G. Cuticle proteins identified by ANOVA in adult *Ae*. *aegypti* permethrin-resistant strain (q-value <0.05).** Notes: FAR: Field strain adult *Ae. aegypti* permethrin resistant. FAN: Field strain adult *Ae. aegypti* not exposed to permethrin. SAE: Laboratory strain adult *Ae. aegypti* exposed to permethrin. SAN: Laboratory strain adult *Ae. aegypti* not exposed to permethrin.

**Notes:**

| 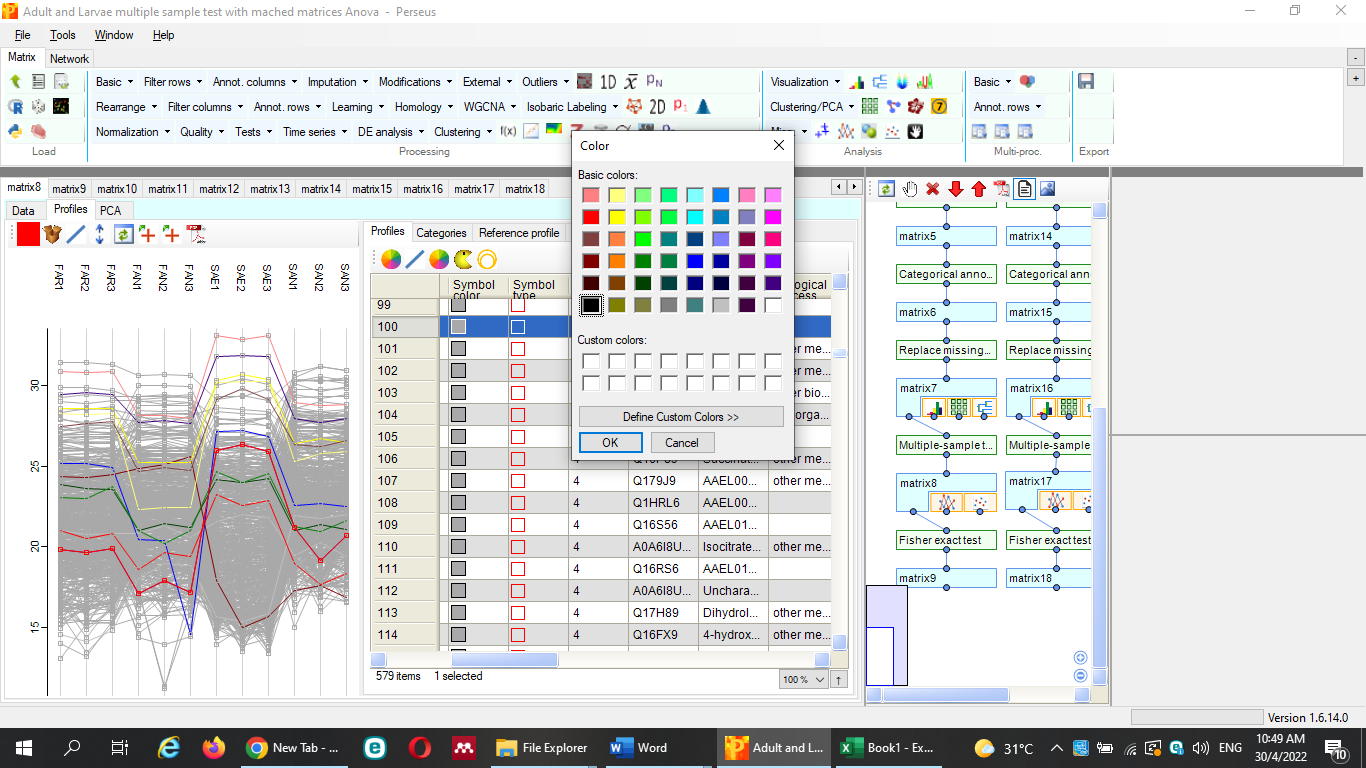 | Pupal cuticle protein, putative (A0A1S4FZ80) | 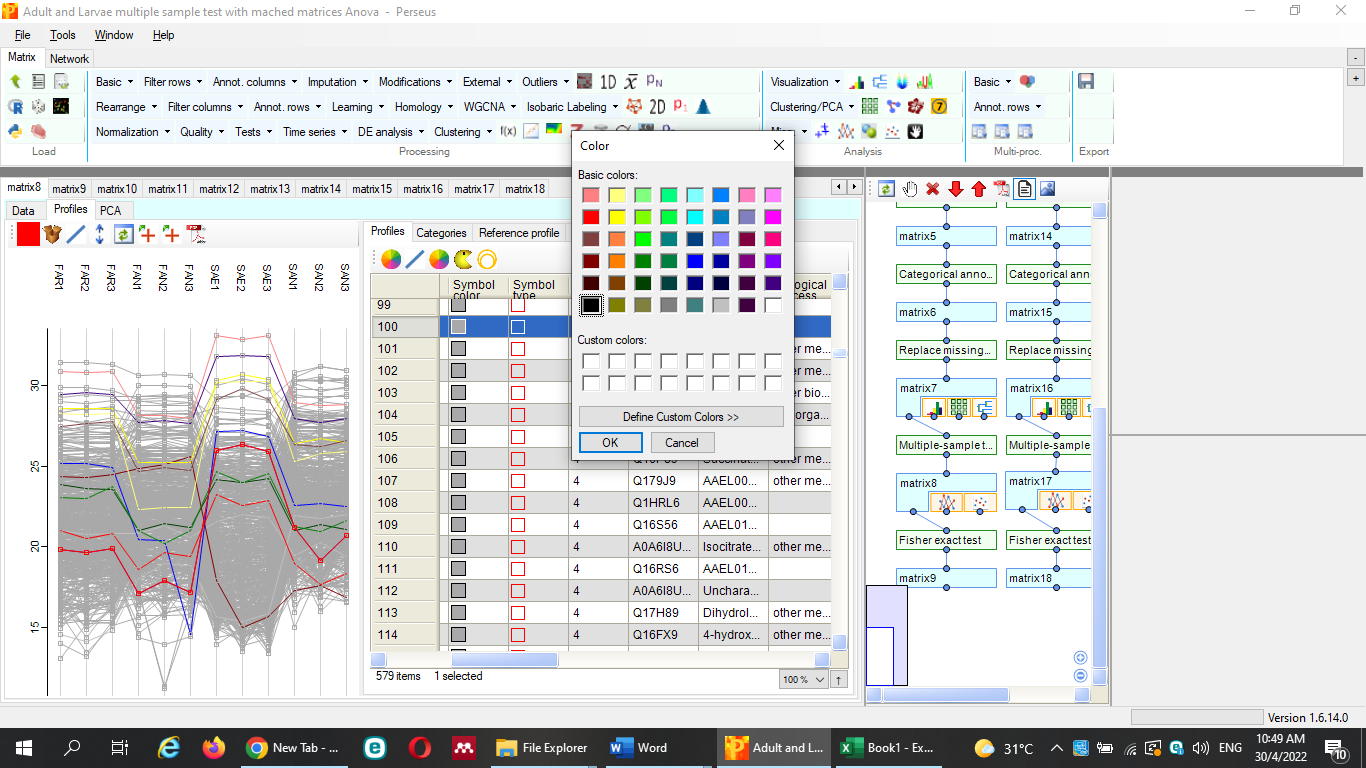 | Cuticle protein 2 (Q17LN8) |
| --- | --- | --- | --- |
| 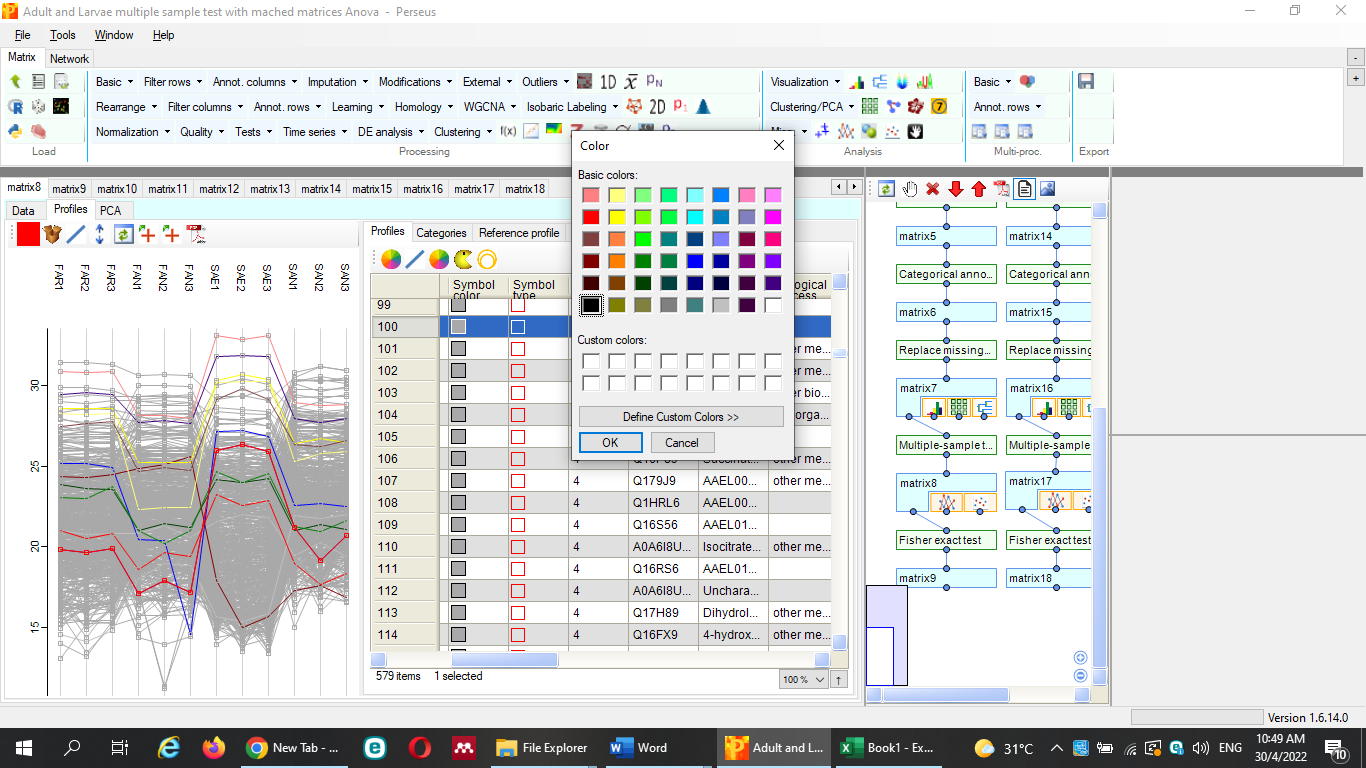 | Cuticle protein 5 (Q16I56) |  |  |


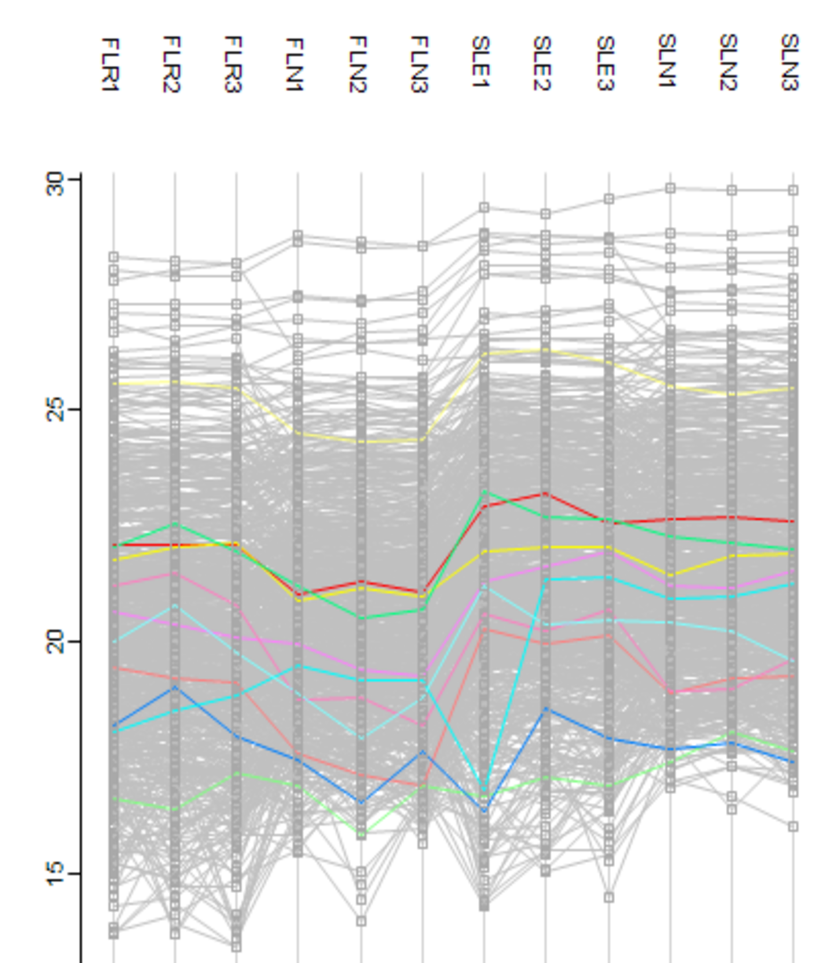


**S Fig H. Cuticle proteins identified by ANOVA in *Ae*. *aegypti* larvae temephos resistant strain (q-value <0.05).** Notes: FLR: Field strain *Ae. aegypti* larvae temephos resistant. FLN: Field strain *Ae. aegypti* larvae not exposed to temephos. SLE: Laboratory strain *Ae. aegypti* larvae exposed to temephos. SLN: Laboratory strain *Ae. aegypti* larvae not exposed to temephos.

**Notes:**

| 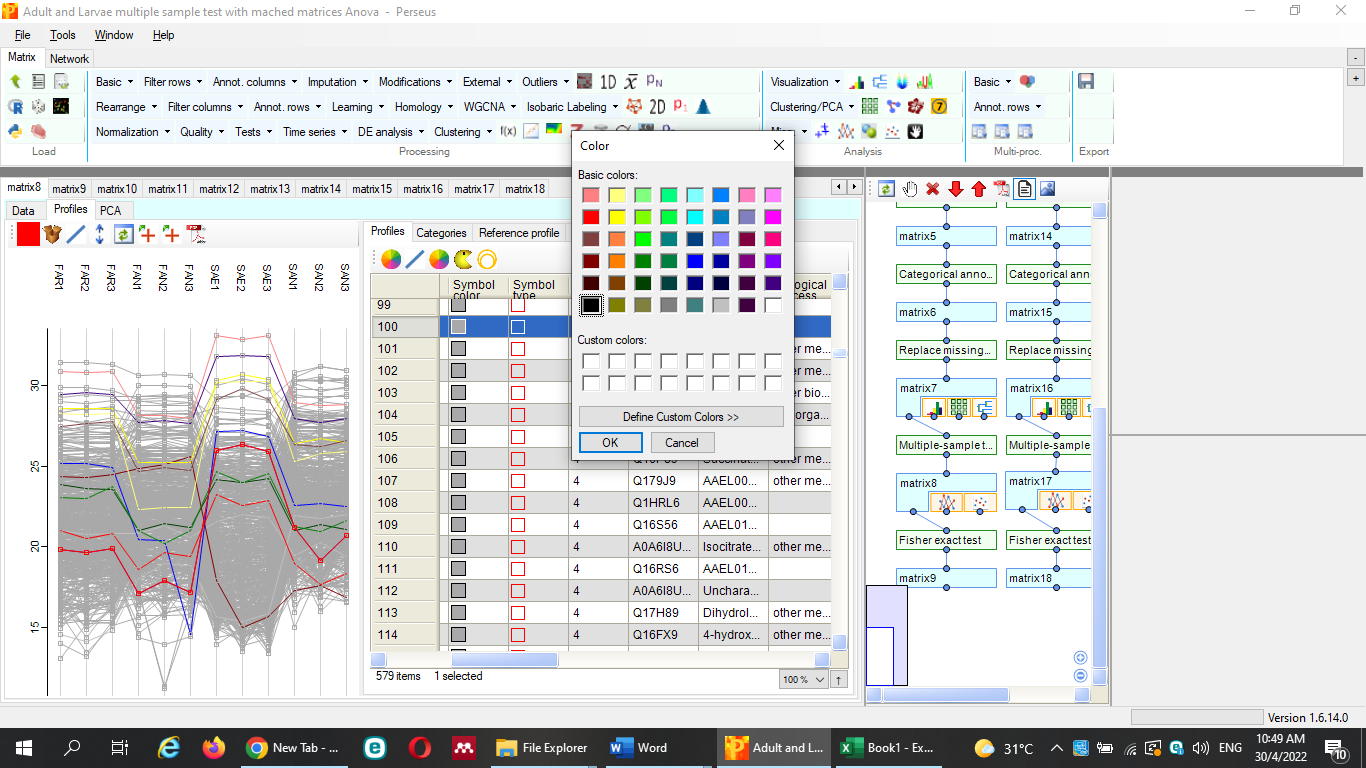 | Pupal cuticle protein 78E, putative (Q0IGD5) | 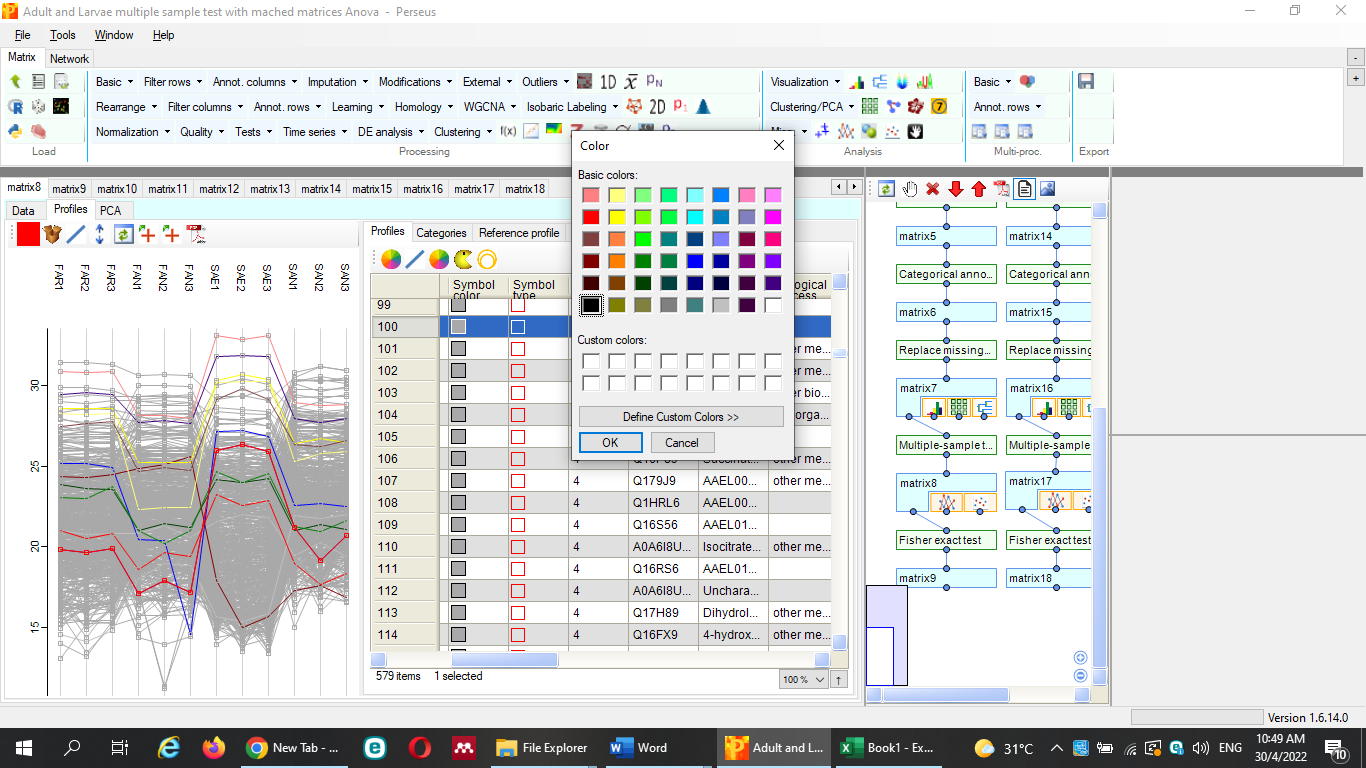 | Pupal cuticle protein 36 (Q17G24) |
| --- | --- | --- | --- |
| 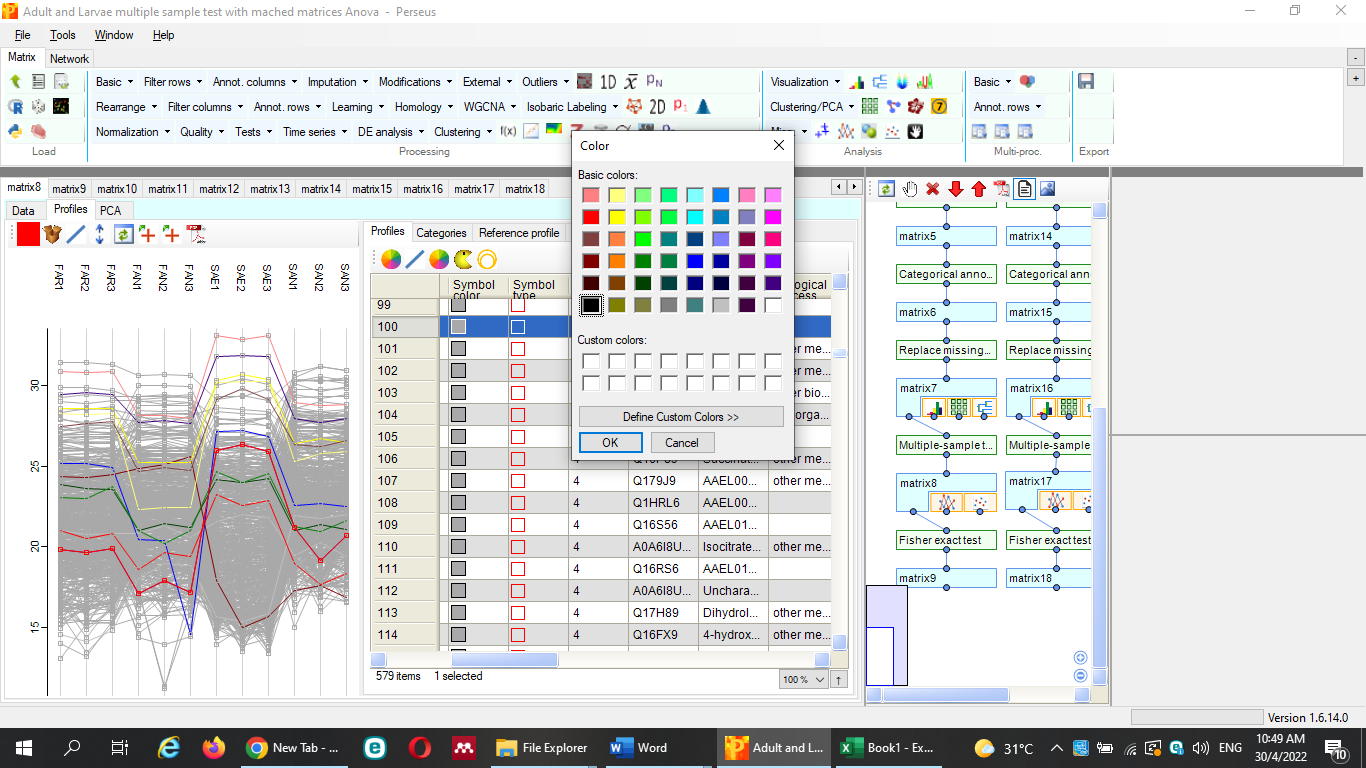 | Pupal cuticle protein, putative (Q17FX9) | 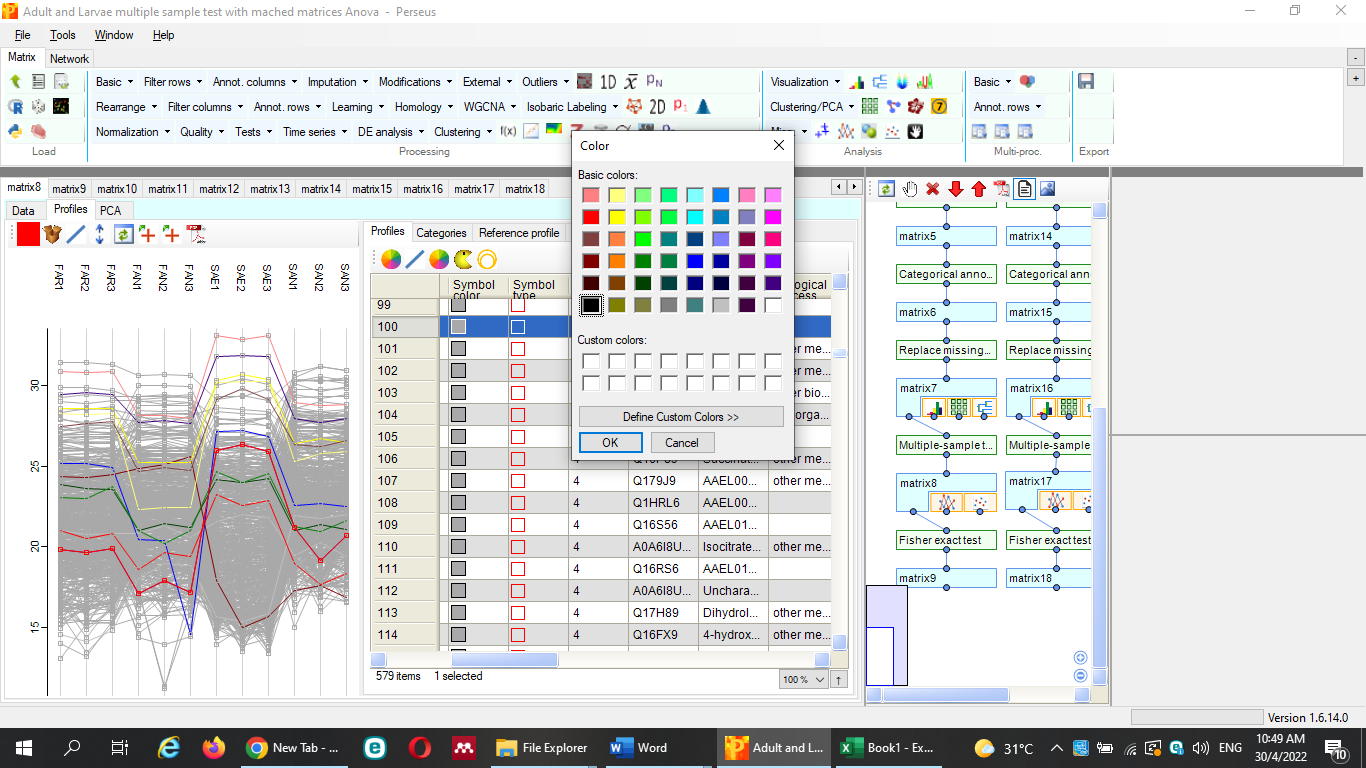 | Cuticle protein, putative (Q16E70) |
| 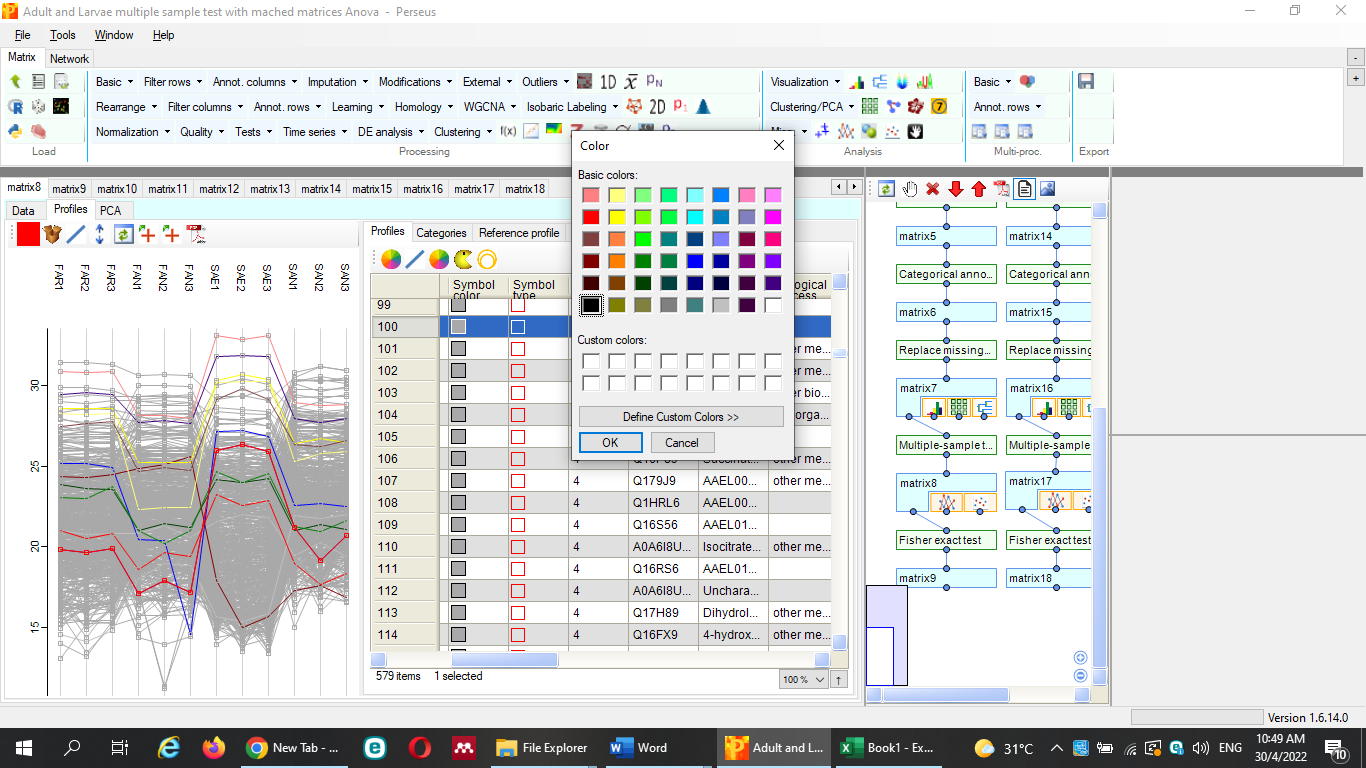 | Cuticle protein, putative (Q16UU3) | 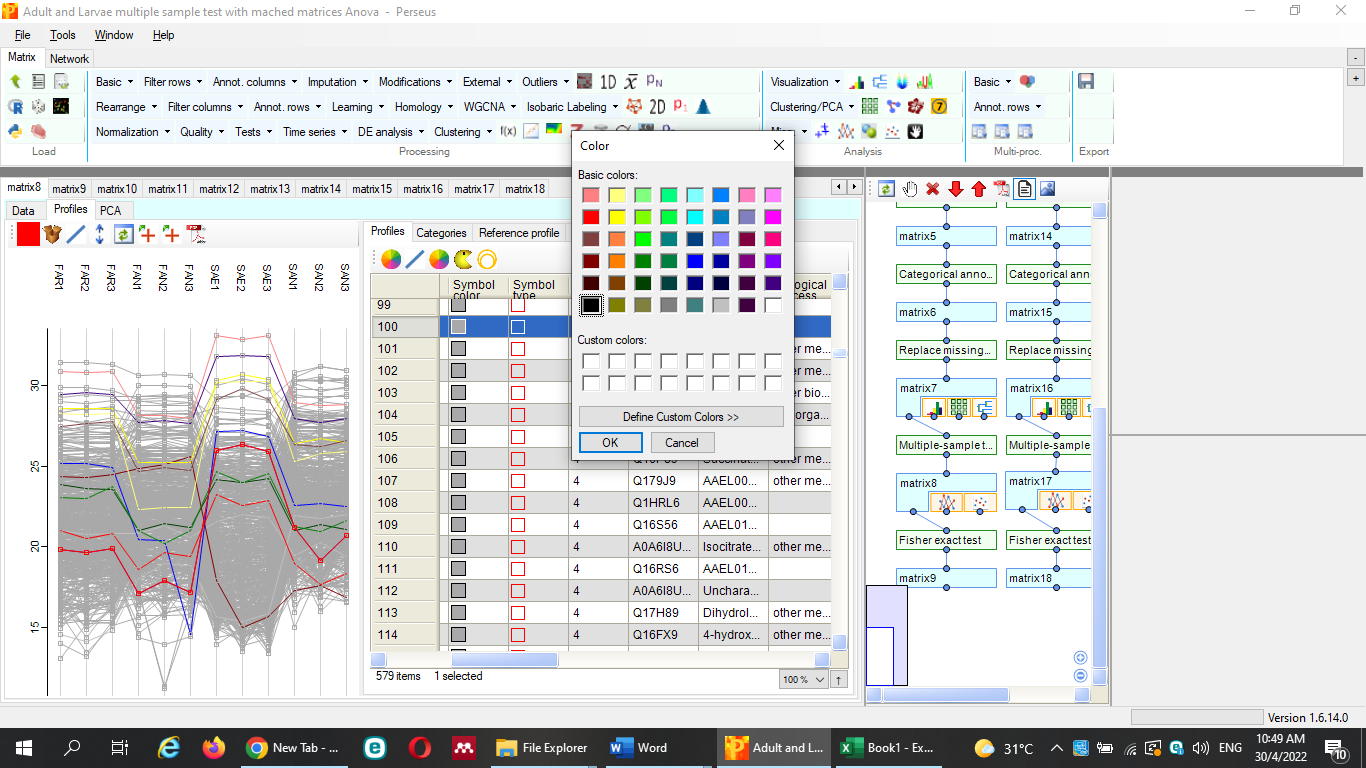 | Cuticle protein, putative (Q16UU3) |
| 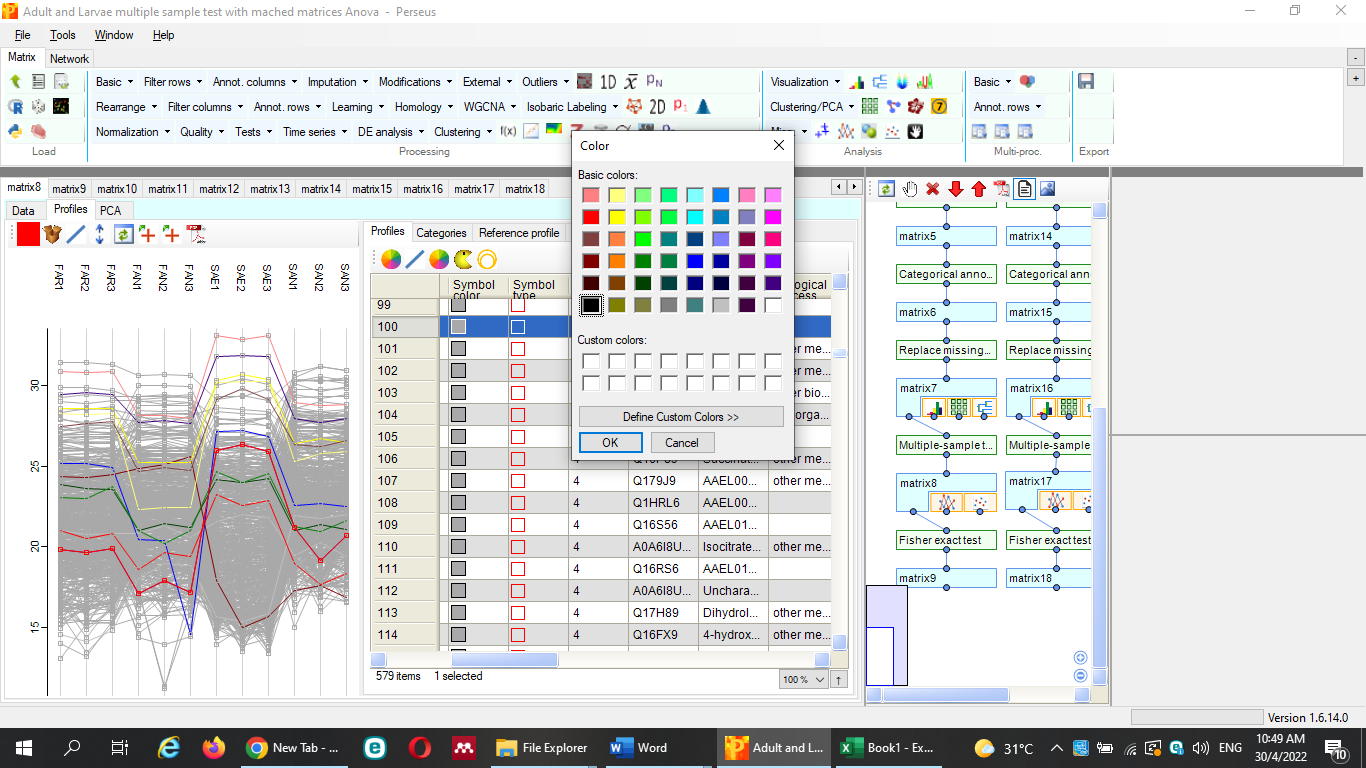 | Cuticle protein, putative (Q16EH6) | 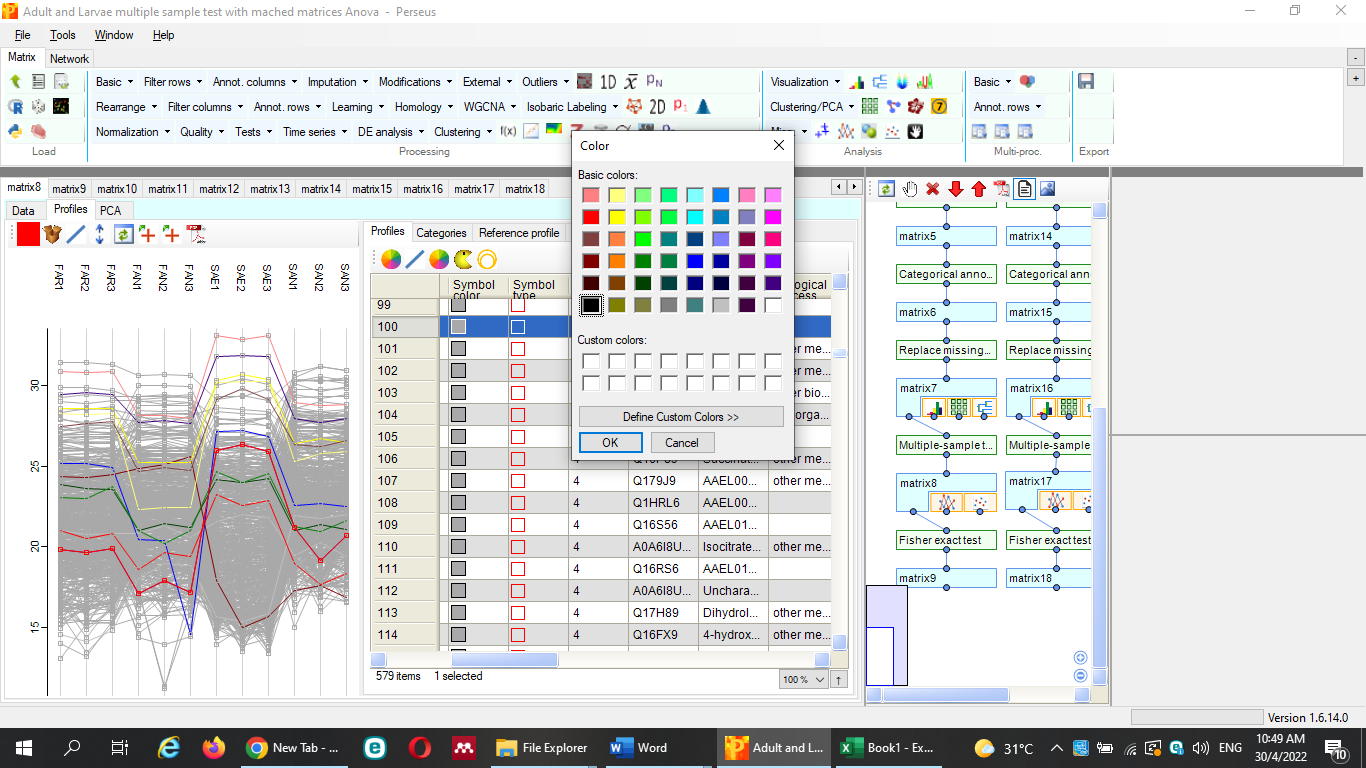 | Cuticle protein, putative (Q16UU4) |
| 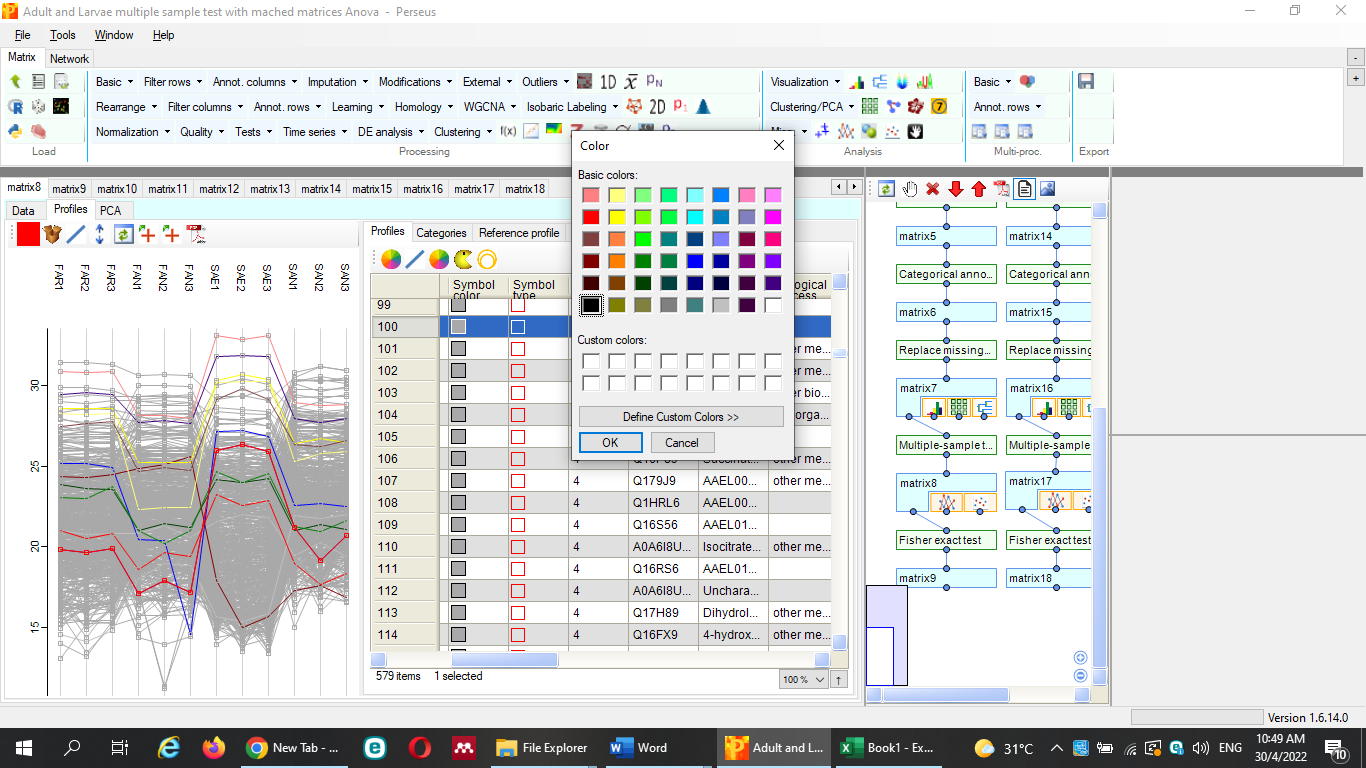 | Cuticle protein (Q17KI1) | 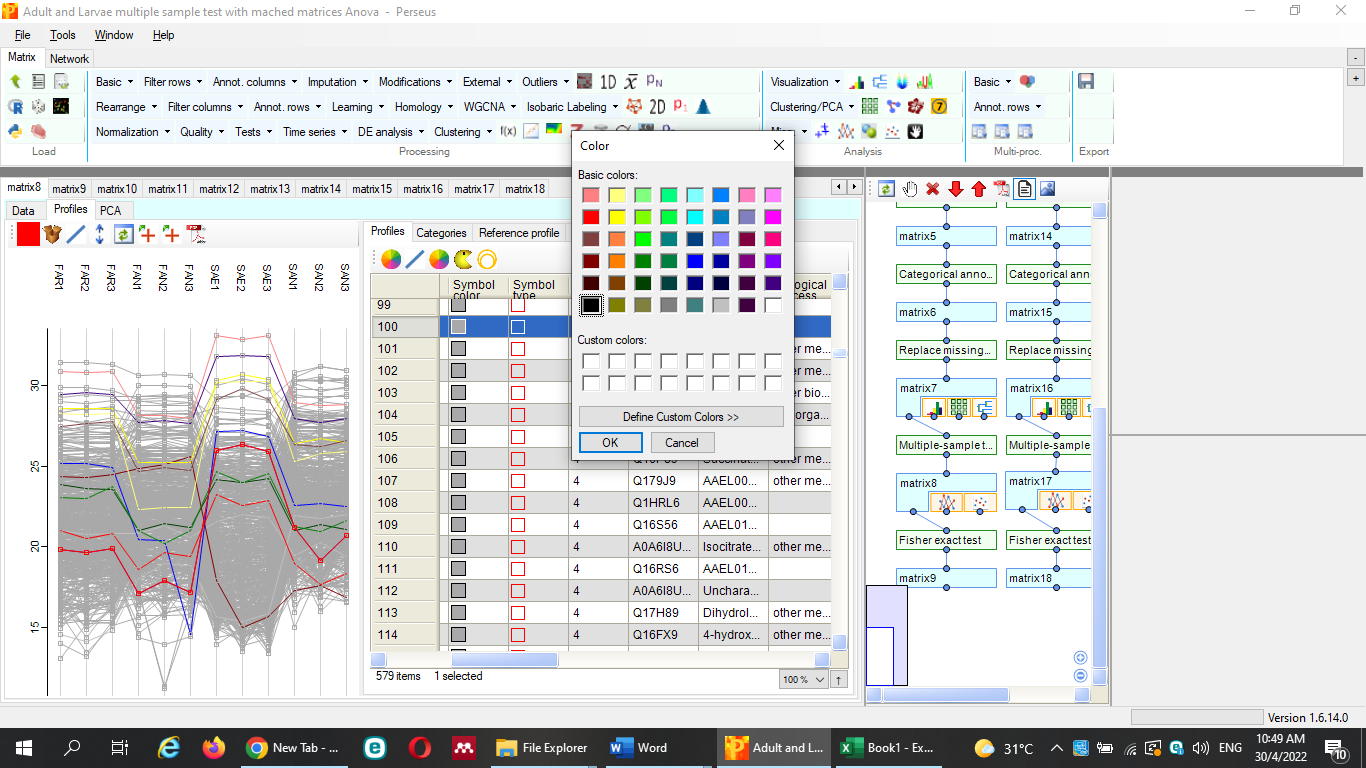 | Larval cuticle protein -30 isoform x1 (Q17G21) |
| 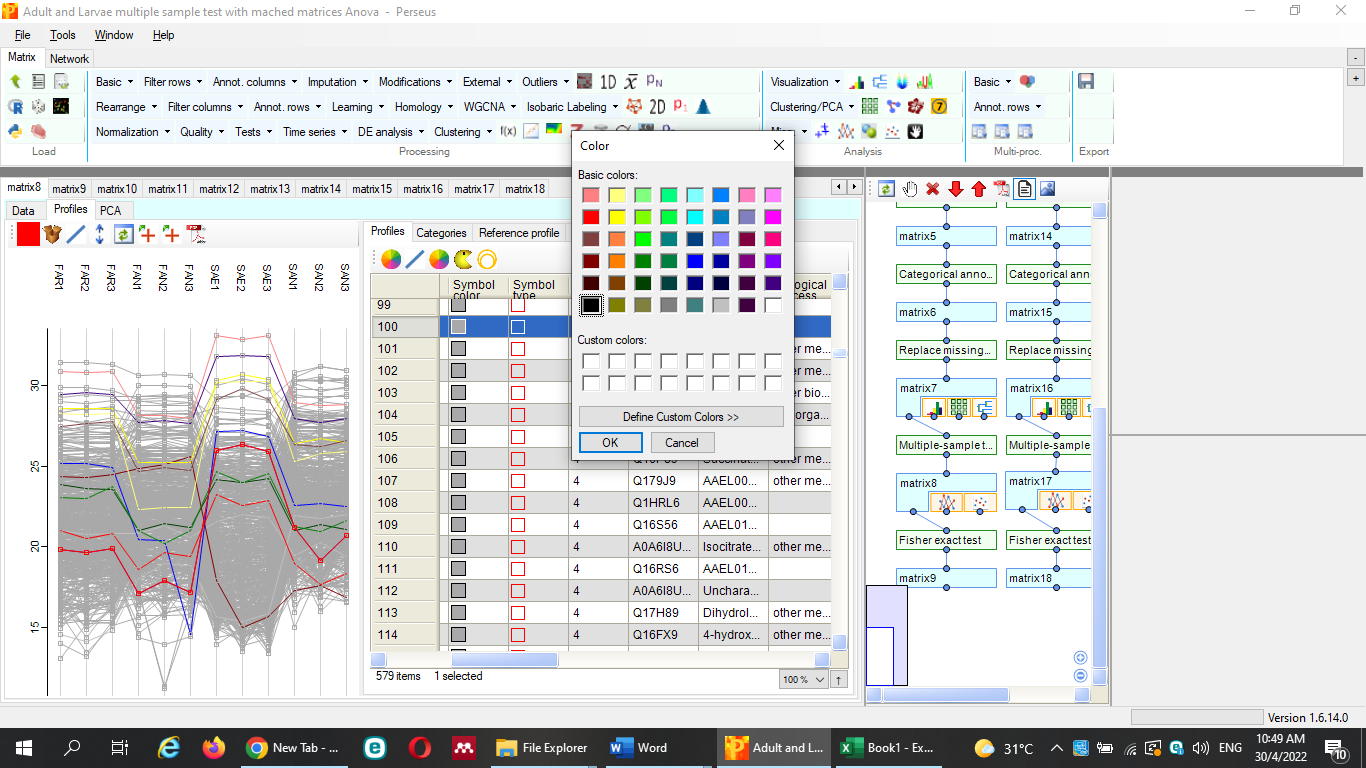 | Larval/pupal cuticle protein h1c (Q17MT9) |  |  |


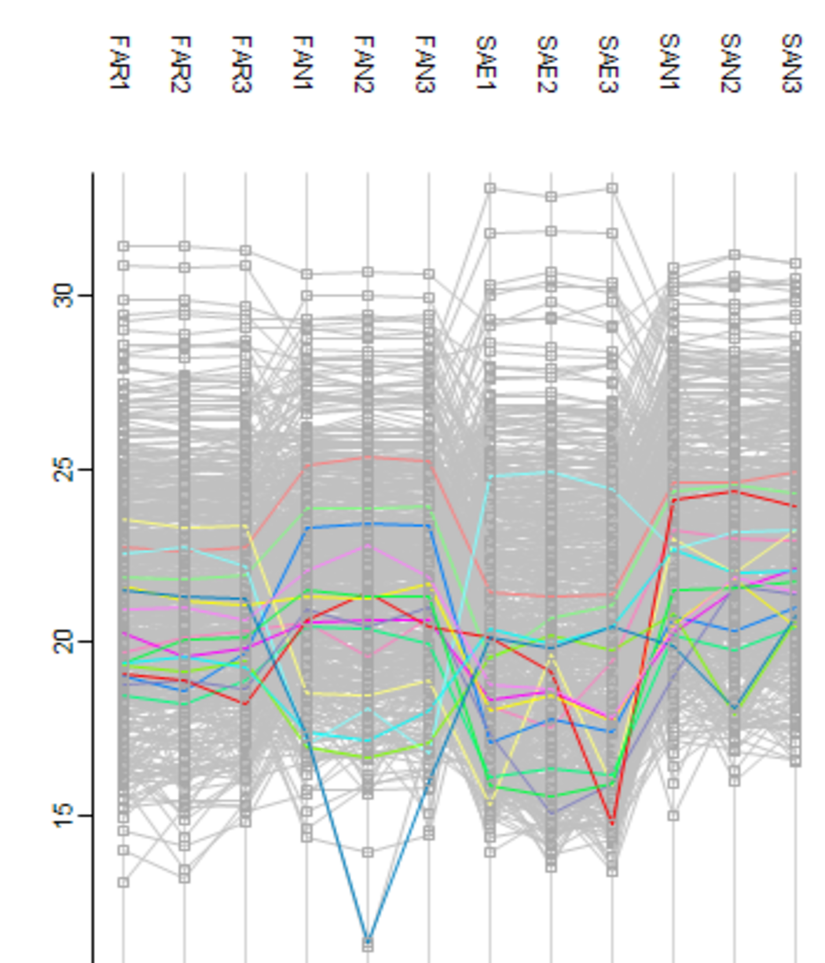


**S Fig I. Ribosomal proteins identified by ANOVA in adult *Ae*. *aegypti* permethrin-resistant strain (q-value <0.05)**. Notes: FAR: Field strain adult *Ae. aegypti* permethrin resistant. FAN: Field strain adult *Ae. aegypti* not exposed to permethrin. SAE: Laboratory strain adult *Ae. aegypti* exposed to permethrin. SAN: Laboratory strain adult *Ae. aegypti* not exposed to permethrin.

**Notes:**

| 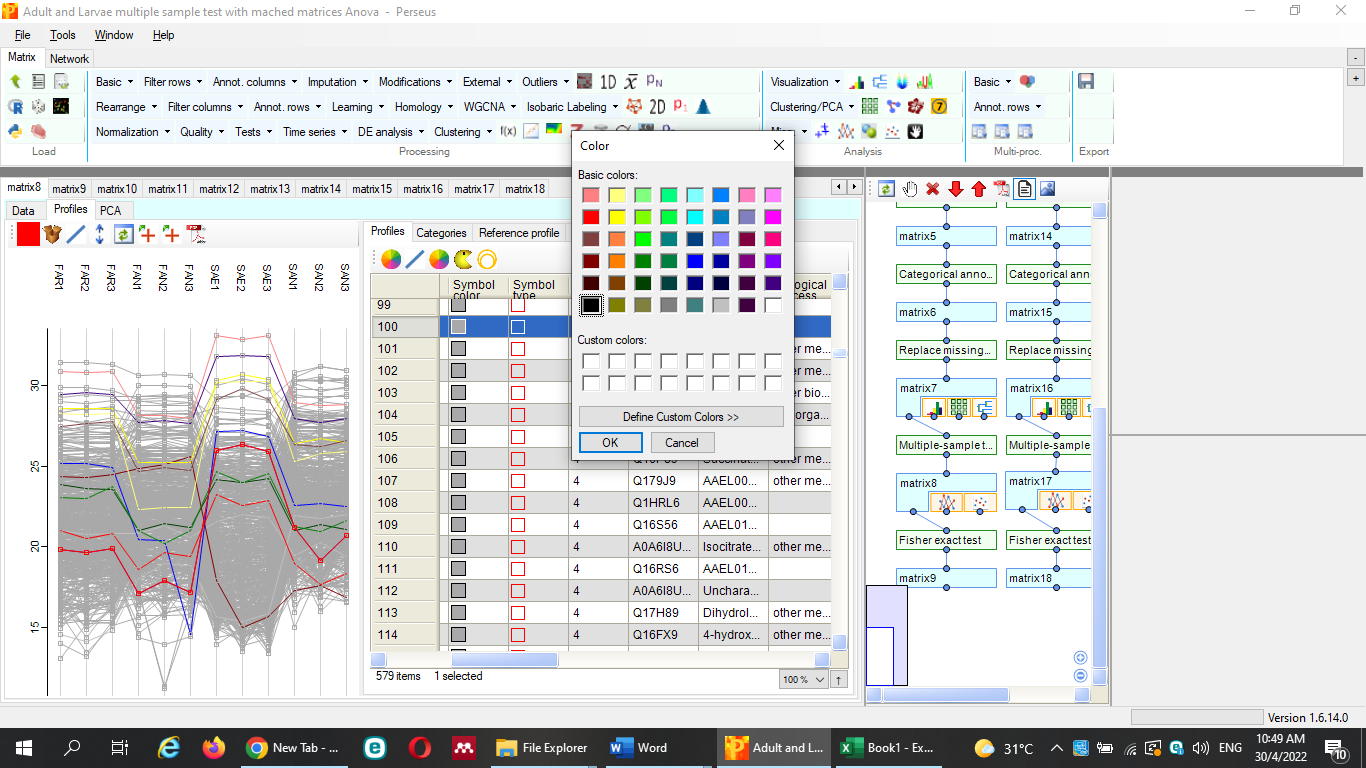 | 40S ribosomal protein S3 (Q4F6X0) | 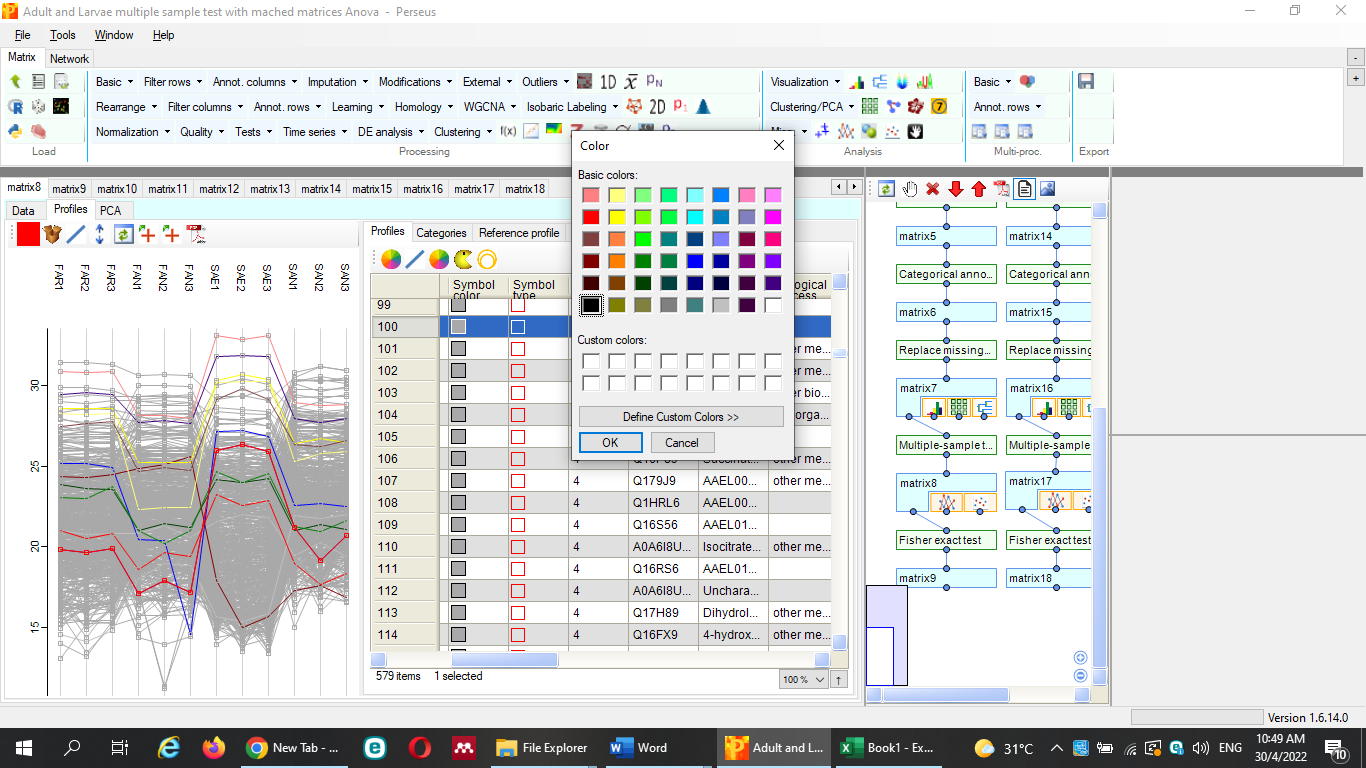 | 60S ribosomal protein L12 (Q1HRI6) |
| --- | --- | --- | --- |
| 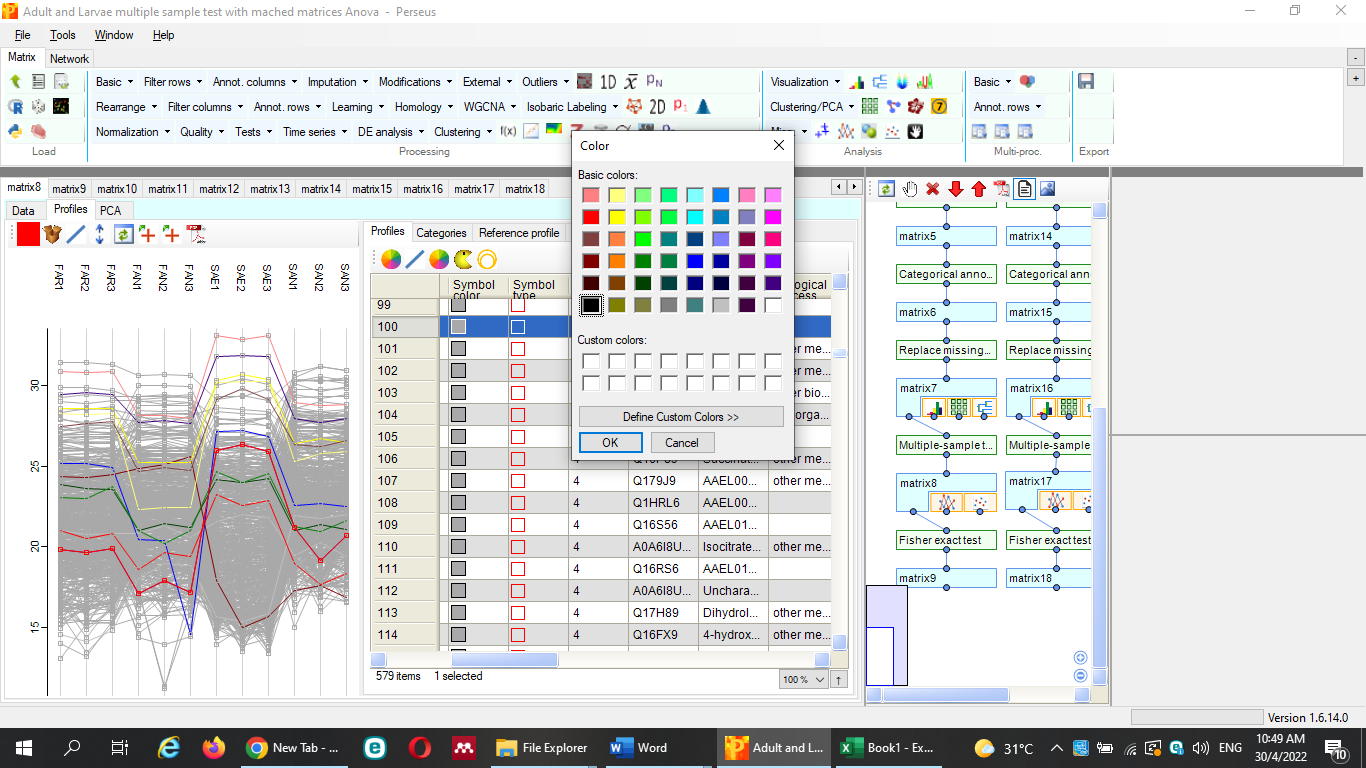 | 40S ribosomal protein S18 (Q1HRL8) | 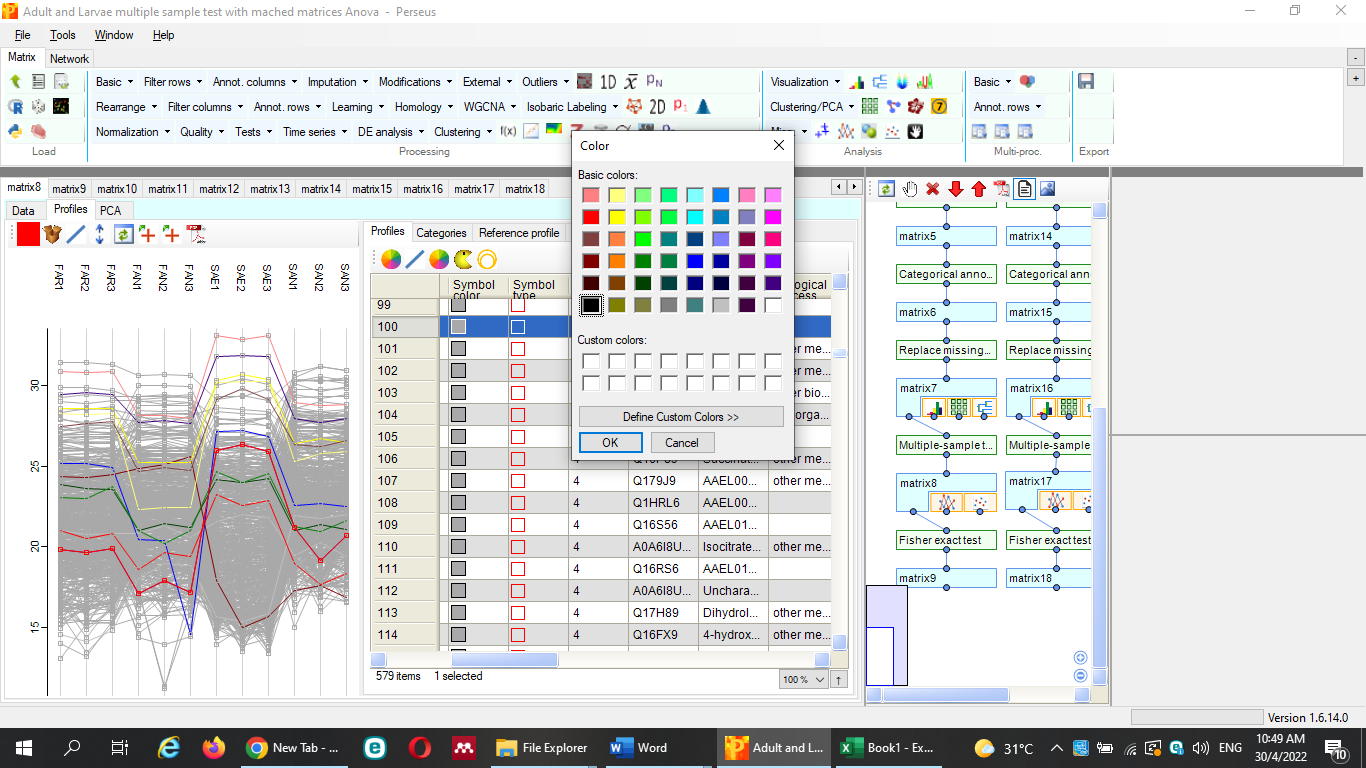 | 60S ribosomal protein L4 (Q1HQJ0) |
| 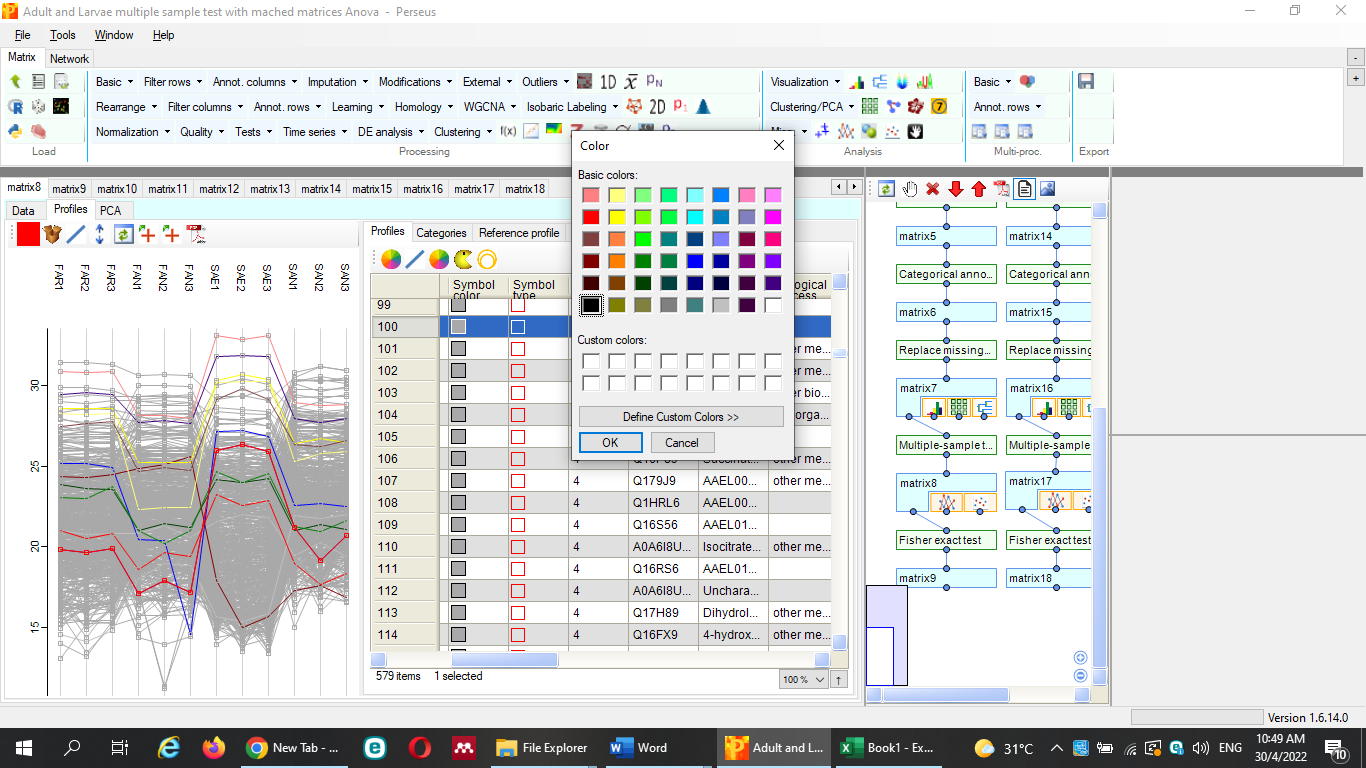 | 40S ribosomal protein S12 (Q1HRM3) | 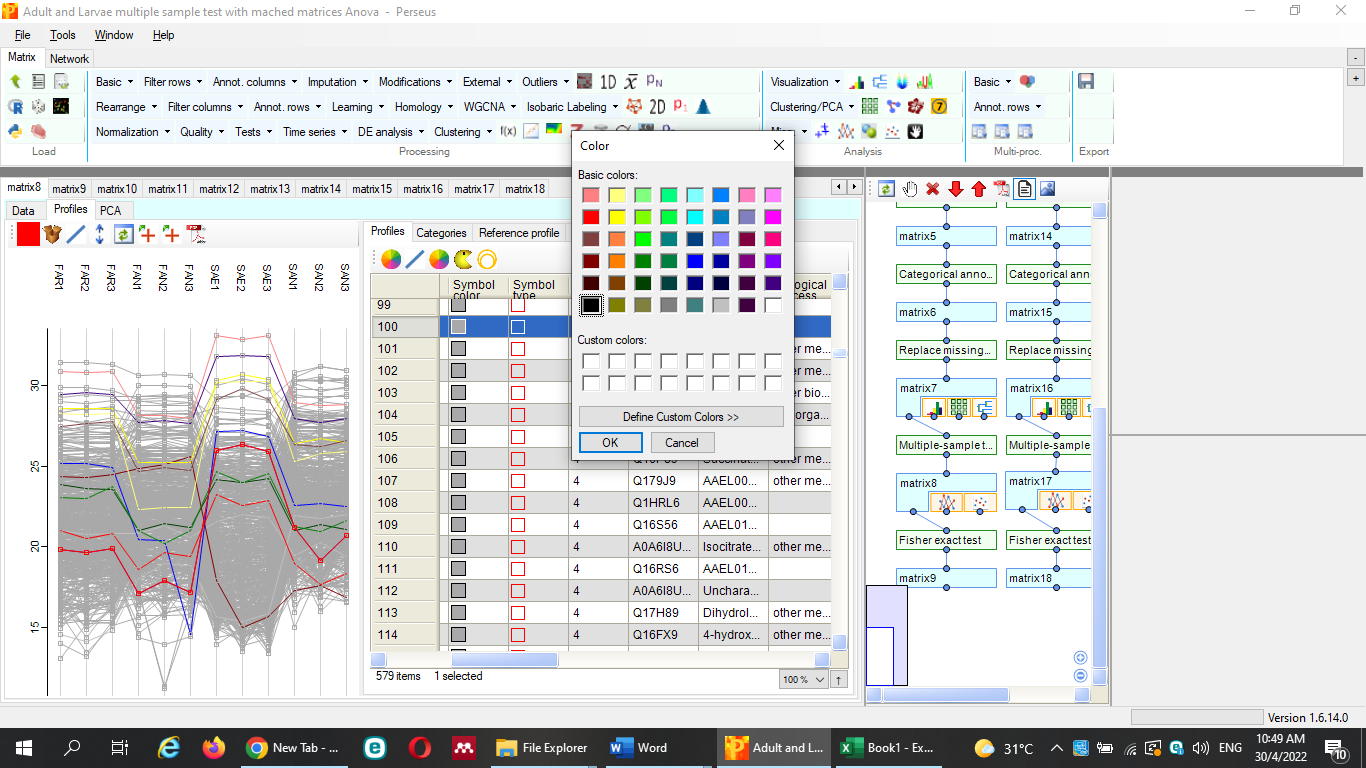 | 60S ribosomal protein L10 (Q1HRT6) |
| 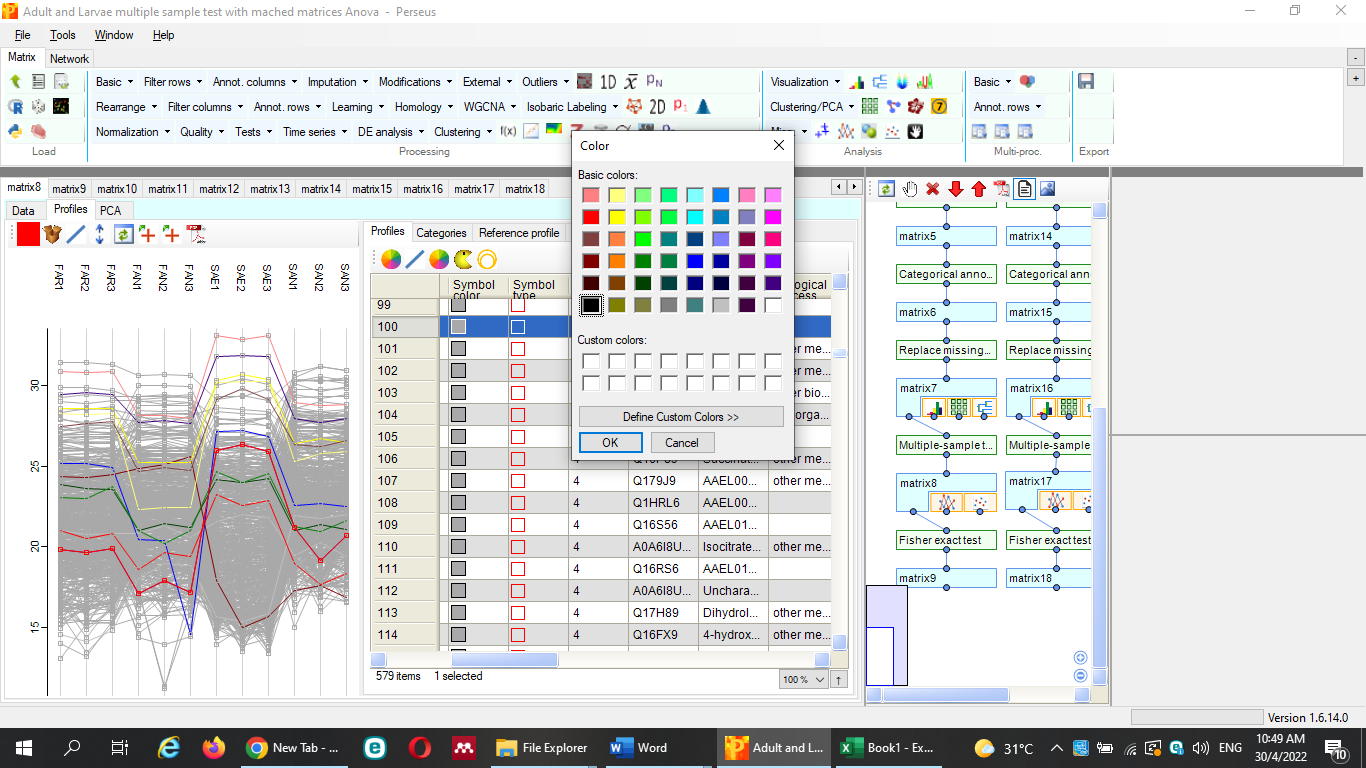 | 60S acidic ribosomal protein P0 (Q1HR99) | 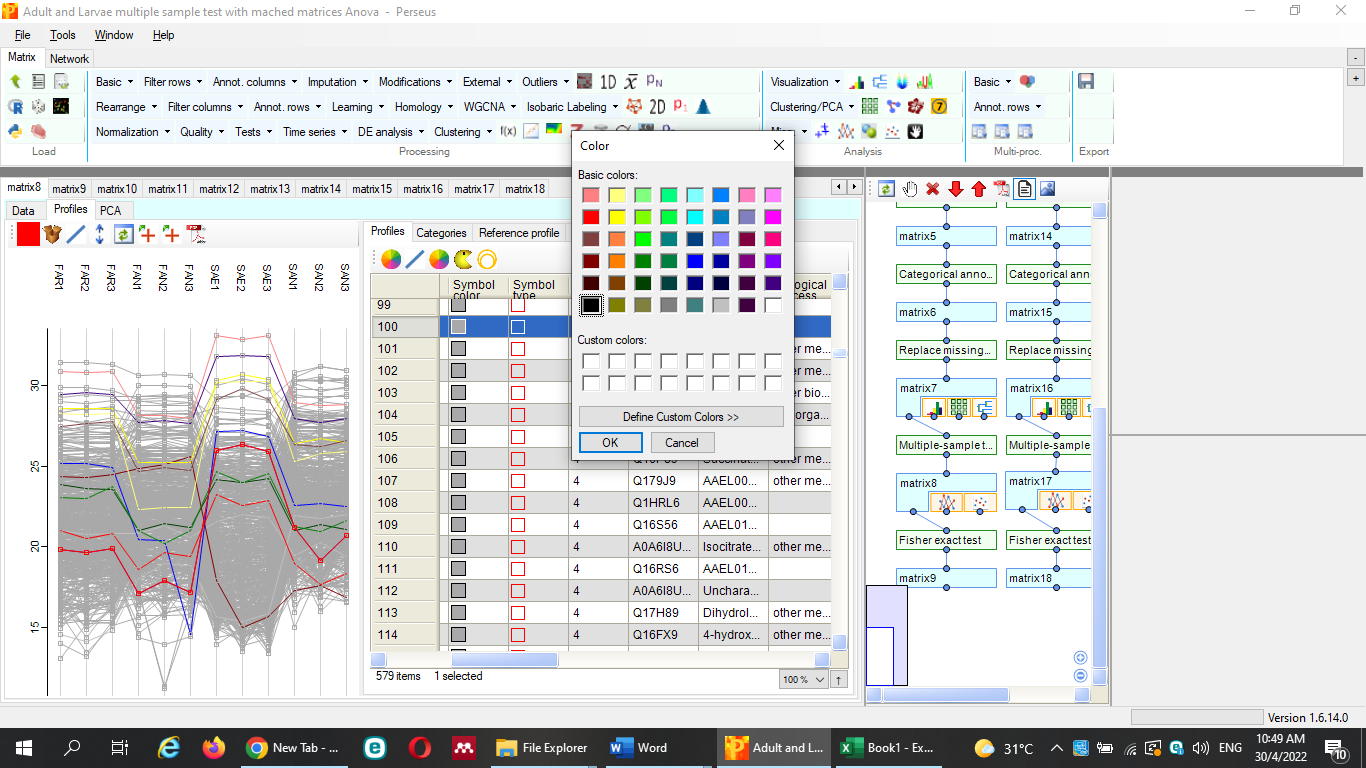 | 60S ribosomal protein L9 (Q1HR72) |
| 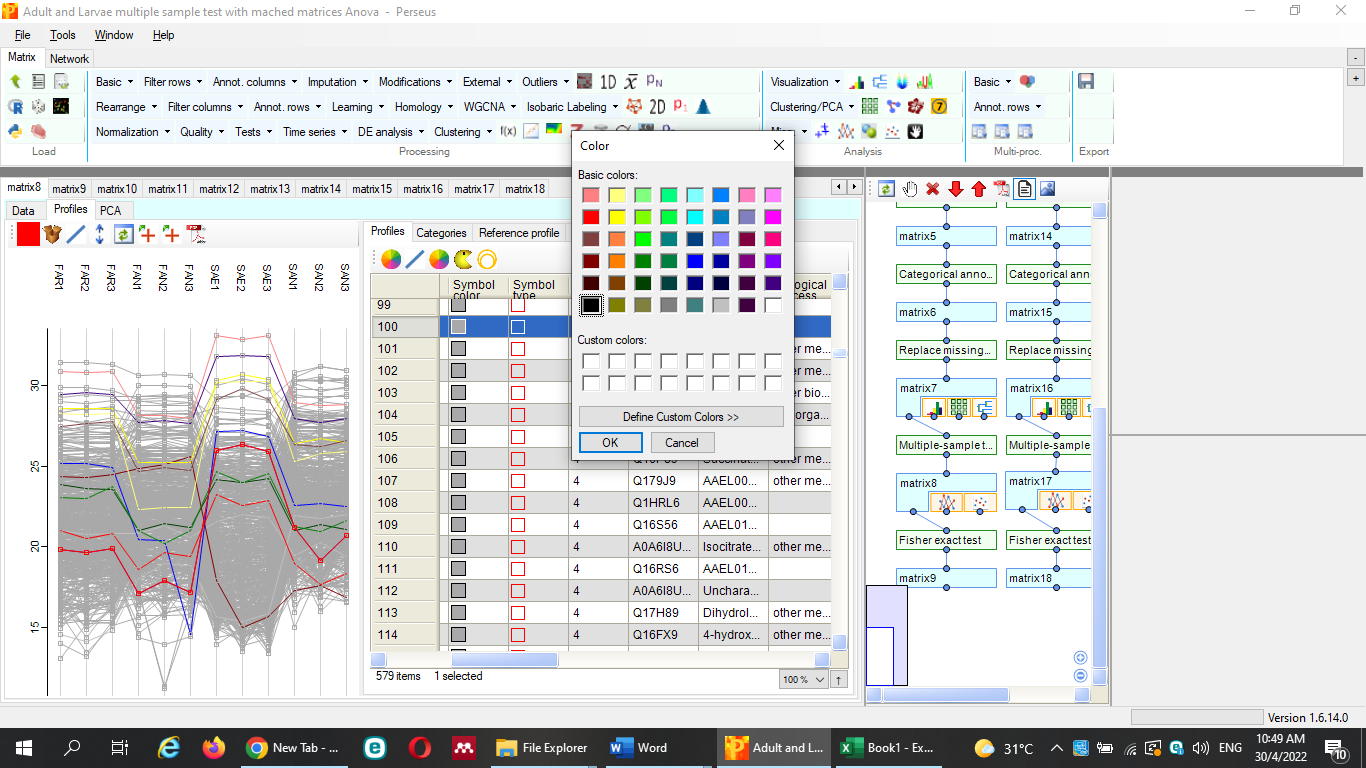 | 40S ribosomal protein S19 (Q1HRR8) | 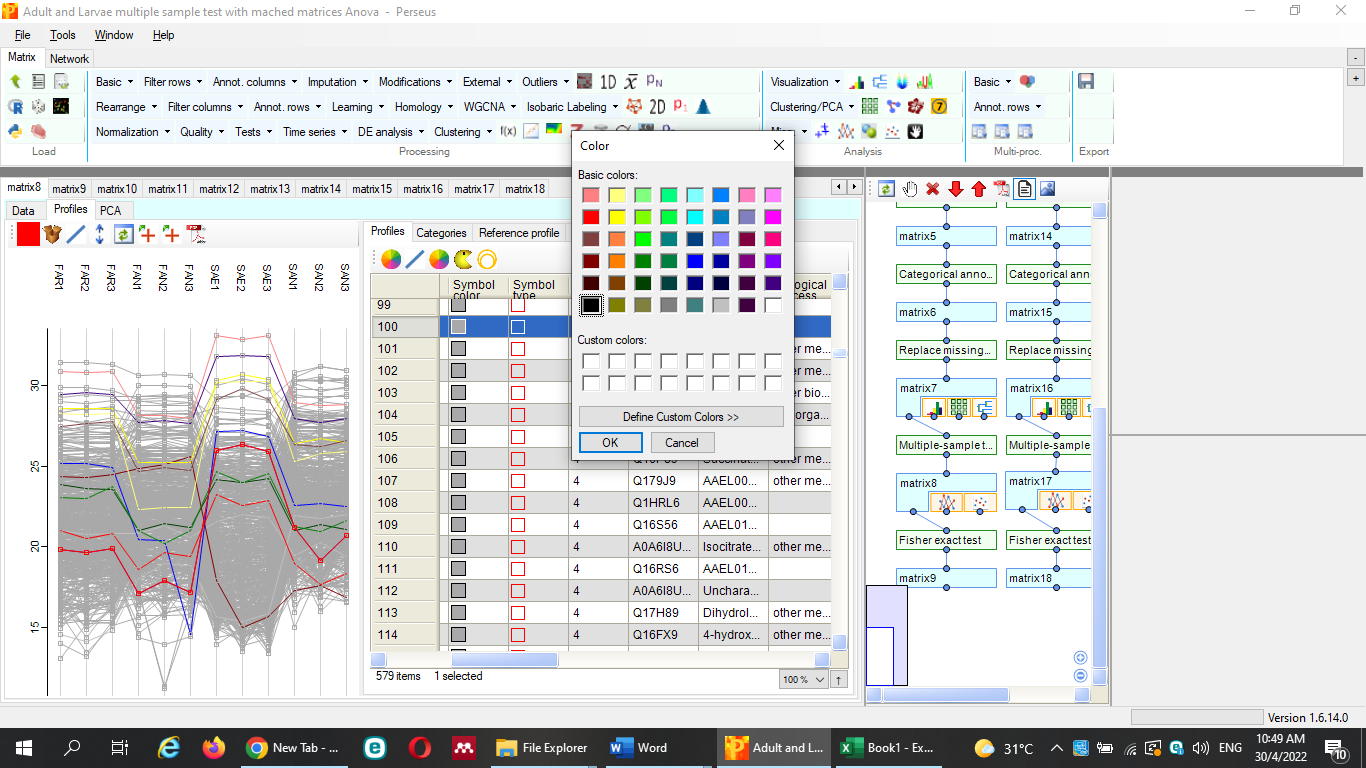 | 60S ribosomal protein L22 (Q1HRP2) |
| 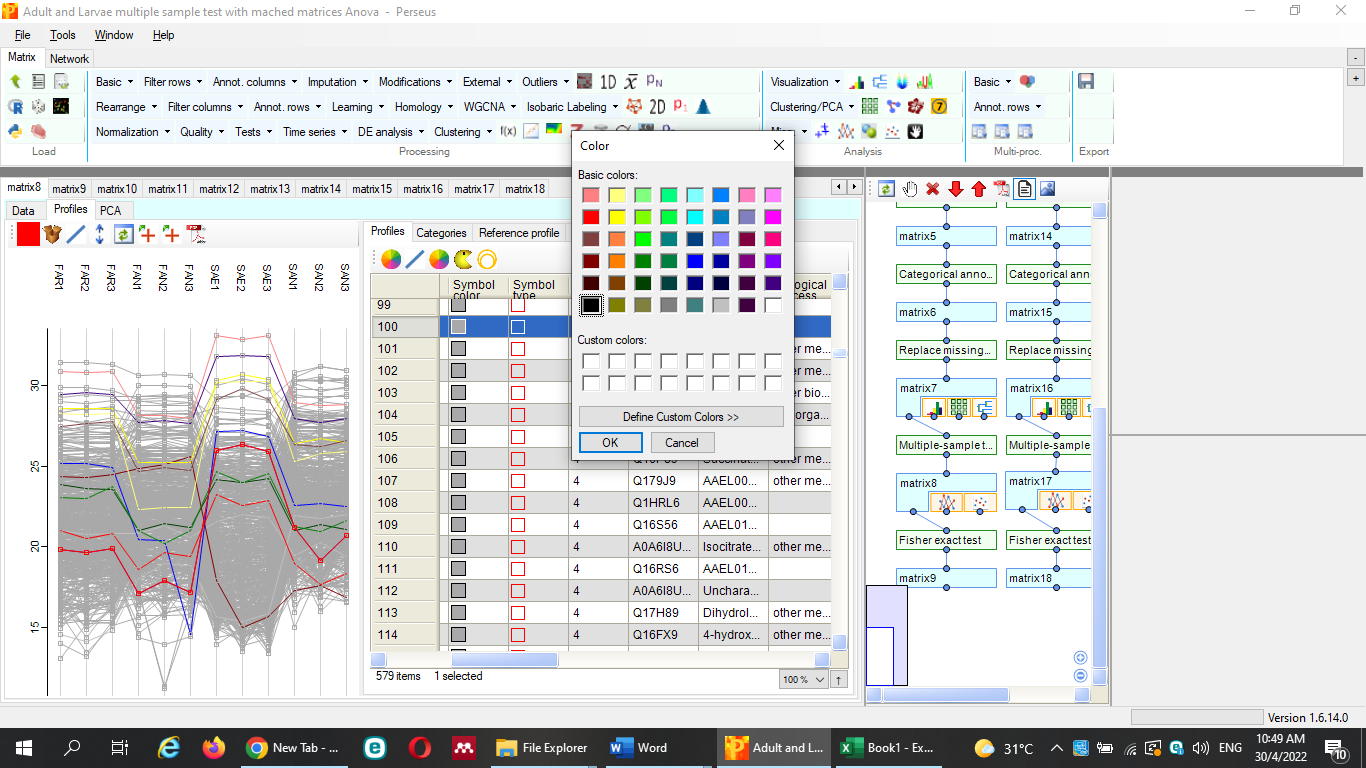 | Acidic ribosomal protein P1, putative (Q17BC2) | 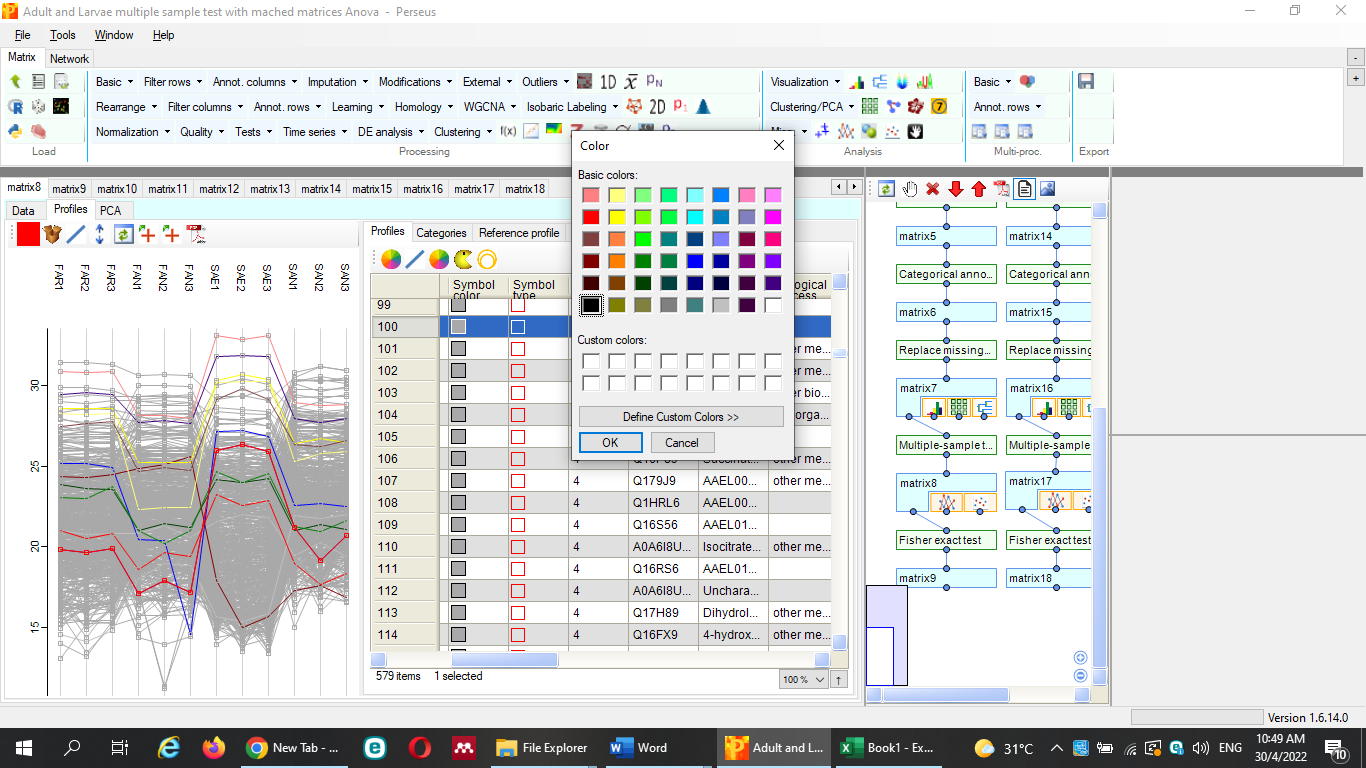 | 40S ribosomal protein S10 (Q1HR41) |
| 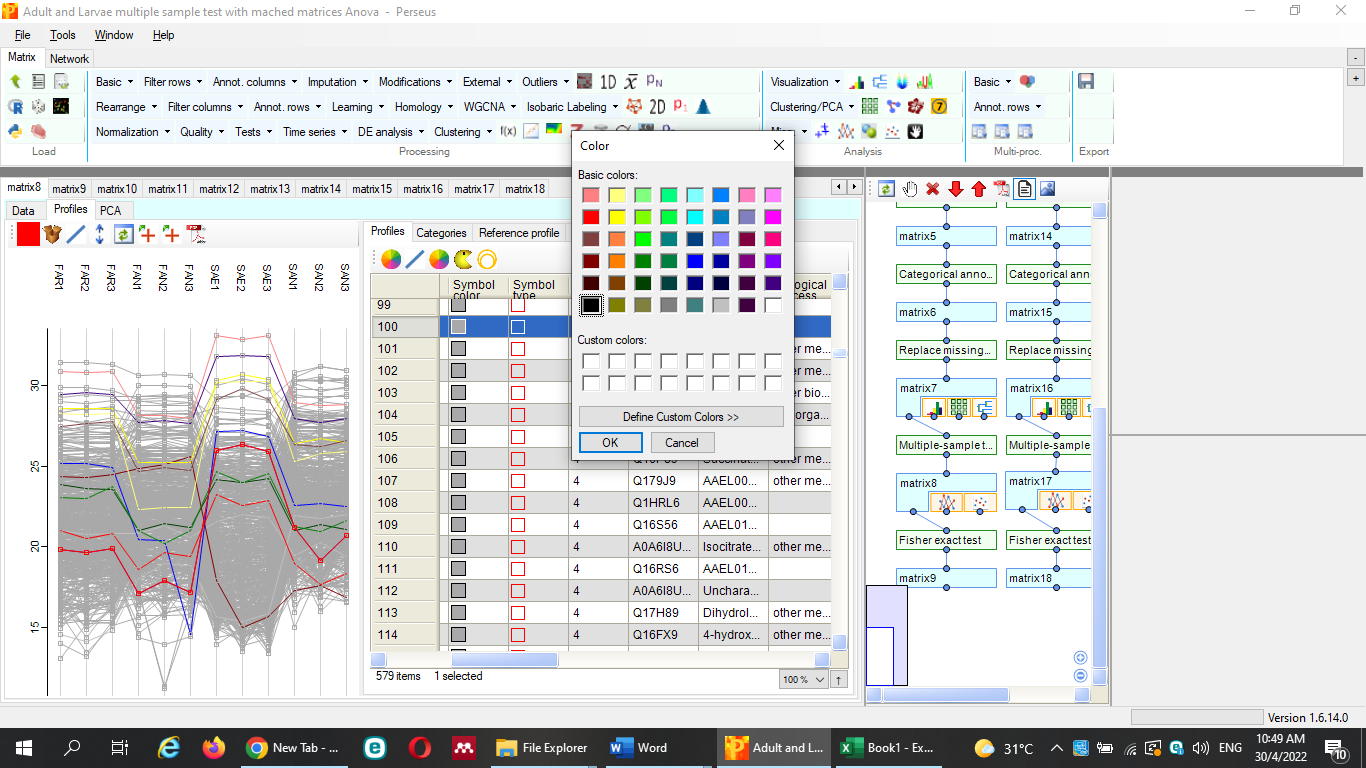 | 60S ribosomal protein L28 (Q1HRJ1) | 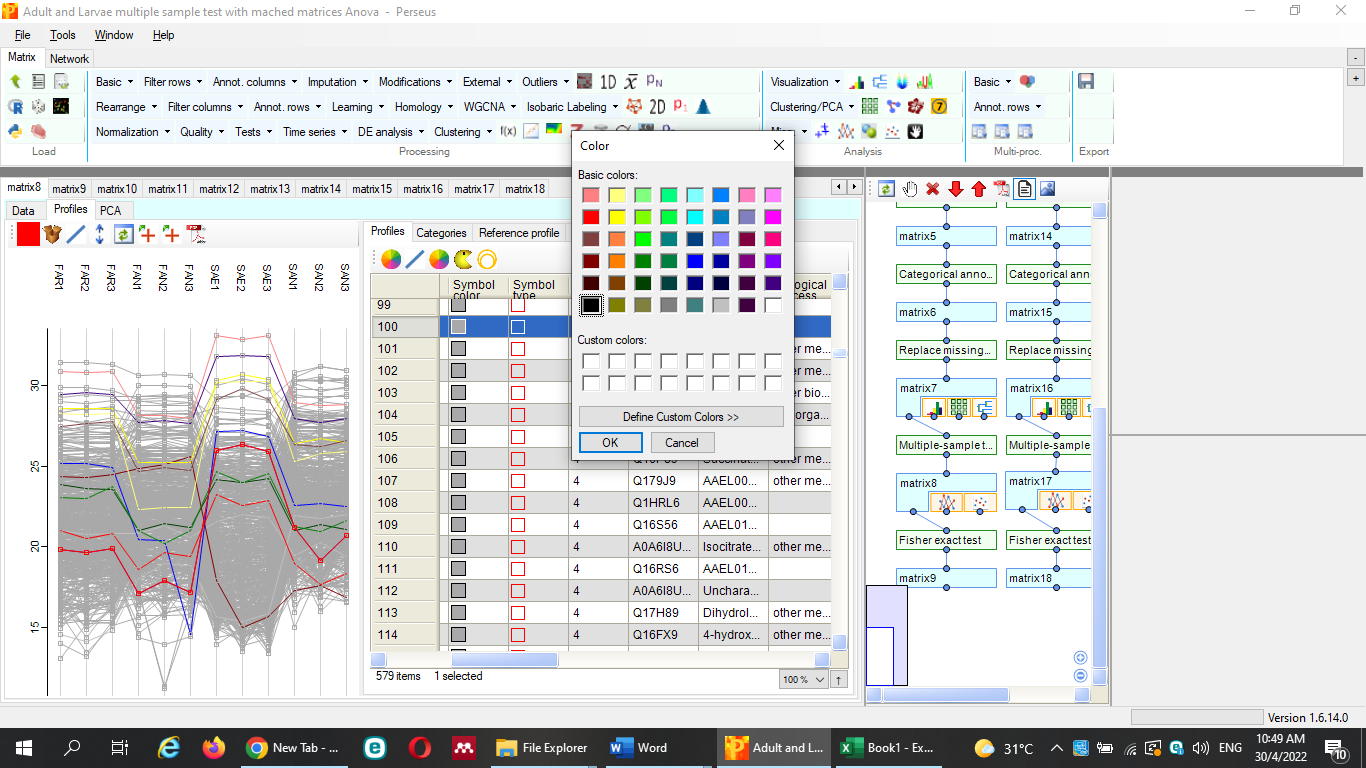 | Large subunit ribosomal protein l23ae (J9HFM9) |
| 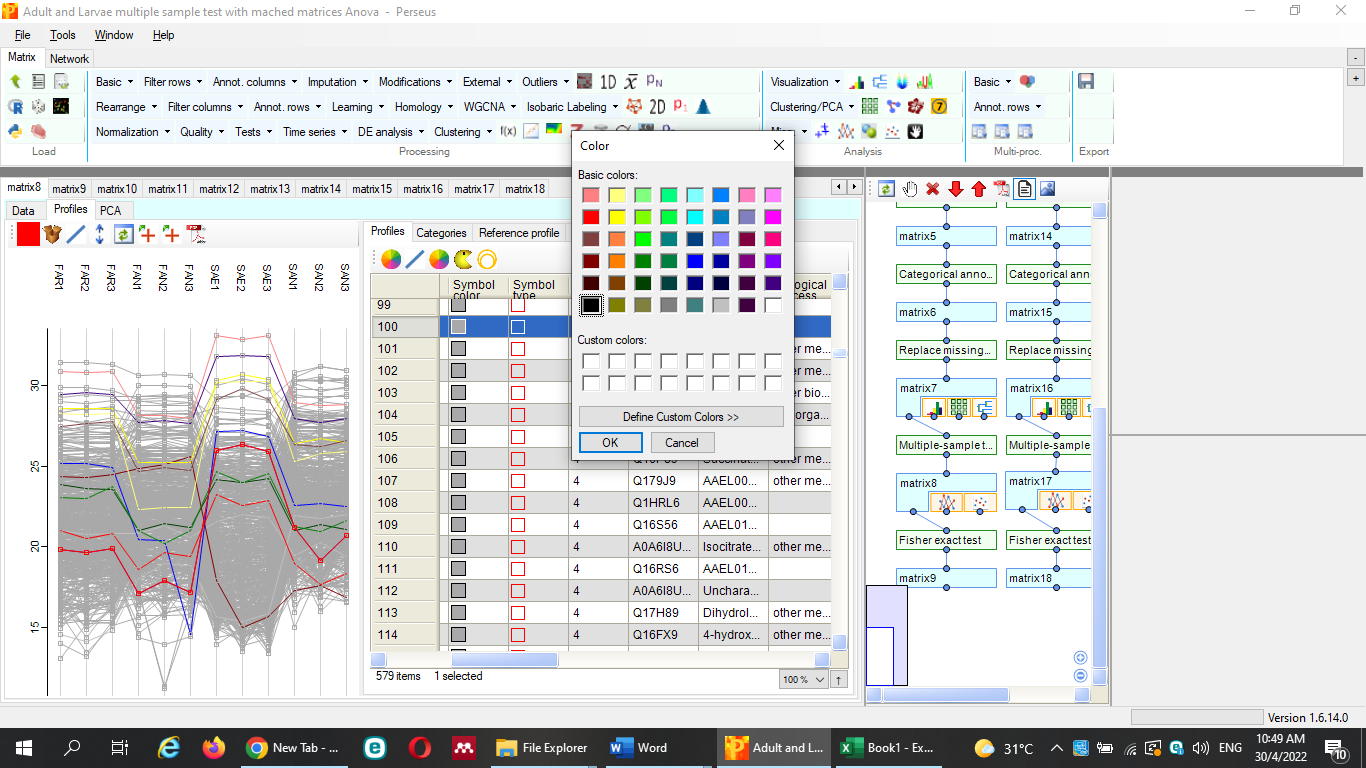 | 60S ribosomal protein L6 (Q16ZH3) | 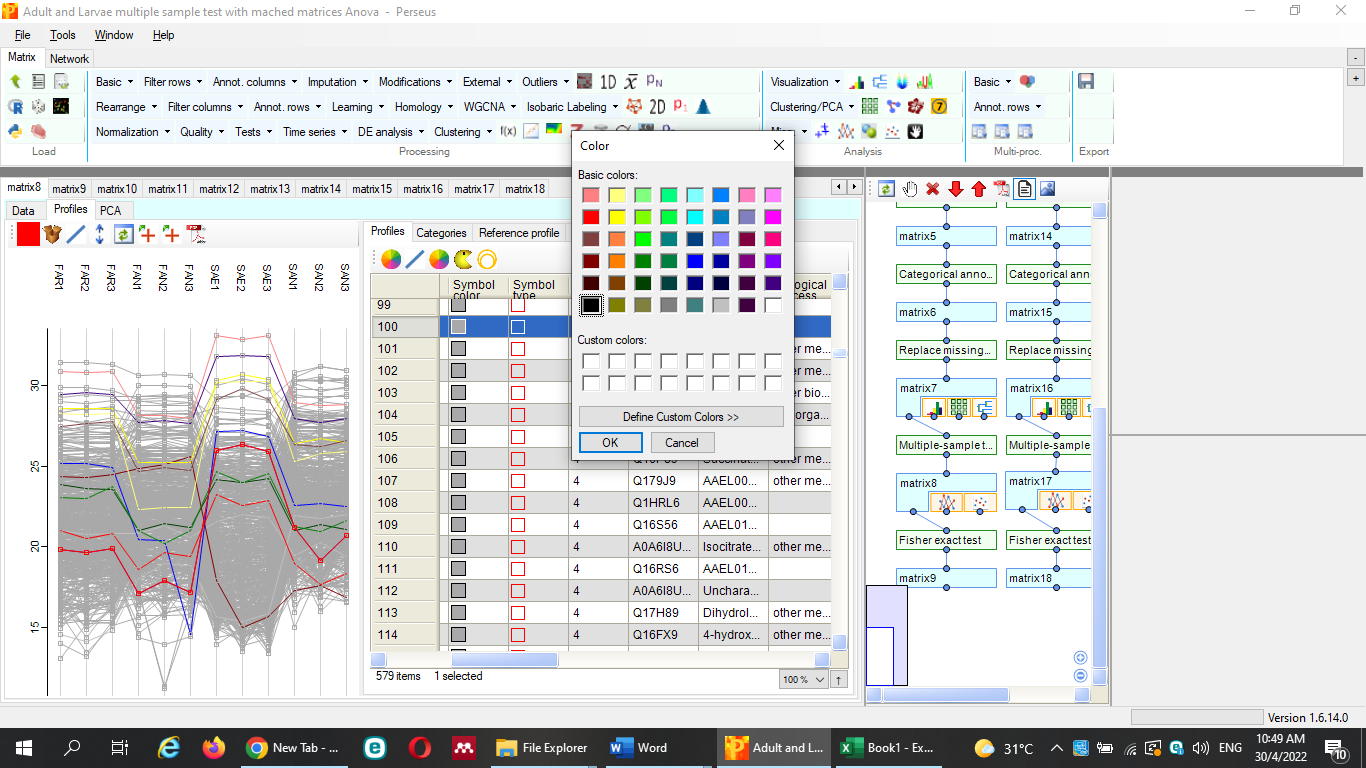 | 60S ribosomal protein L21(Q1HRN4) |


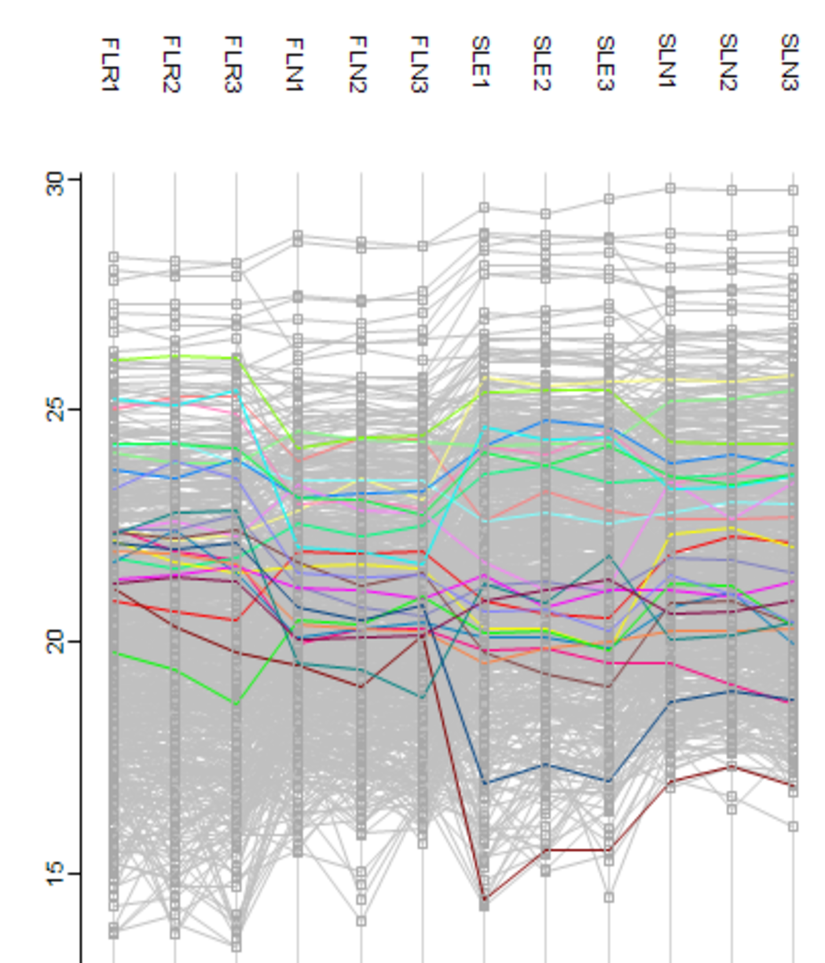


**S Fig J. Ribosomal proteins identified by ANOVA in *Ae*. *aegypti* larvae temephos resistant strain (q-value <0.05).** Notes: FLR: Field strain *Ae. aegypti* larvae temephos resistant. FLN: Field strain *Ae. aegypti* larvae not exposed to temephos. SLE: Laboratory strain *Ae. aegypti* larvae exposed to temephos. SLN: Laboratory strain *Ae. aegypti* larvae not exposed to temephos.

**Notes:**

| 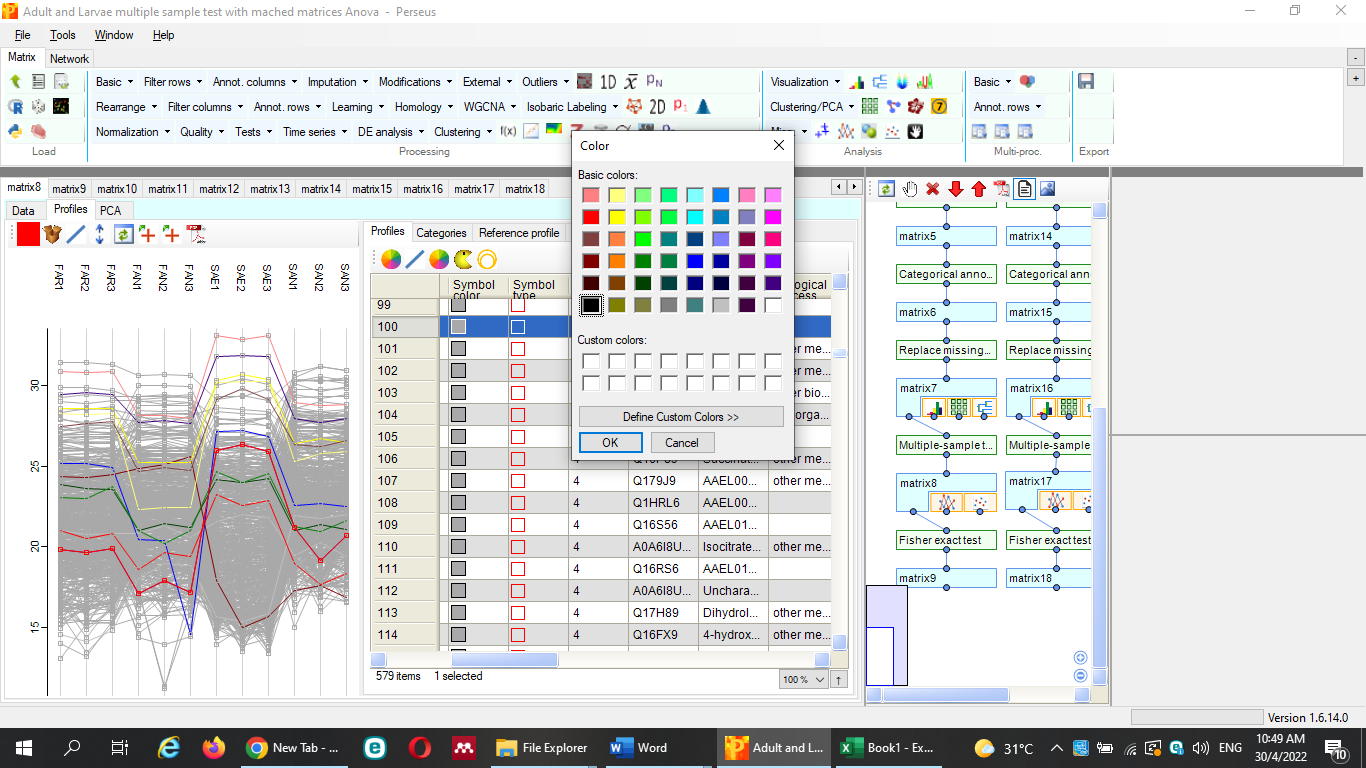 | Large subunit ribosomal protein l23ae (J9HFM9) | 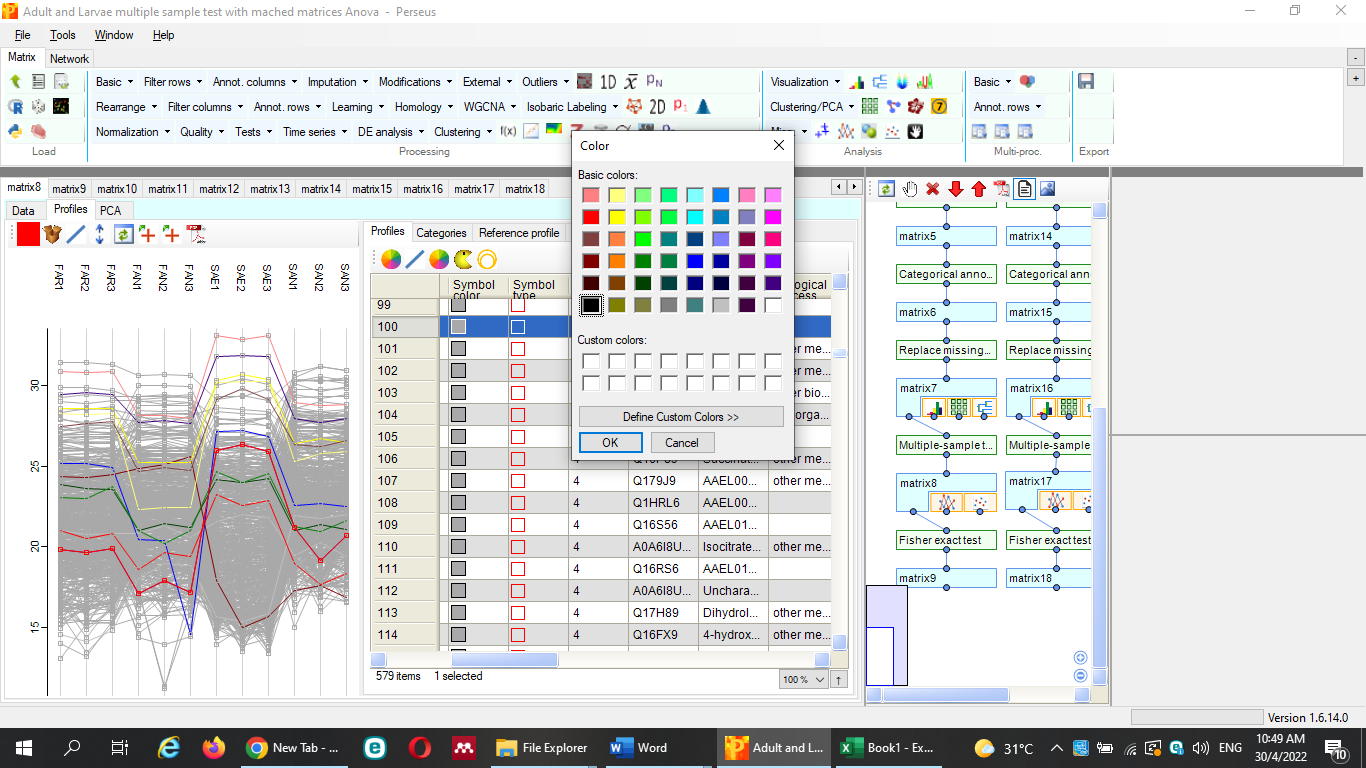 | 60S acidic ribosomal protein P0 (Q1HR99) |
| --- | --- | --- | --- |
| 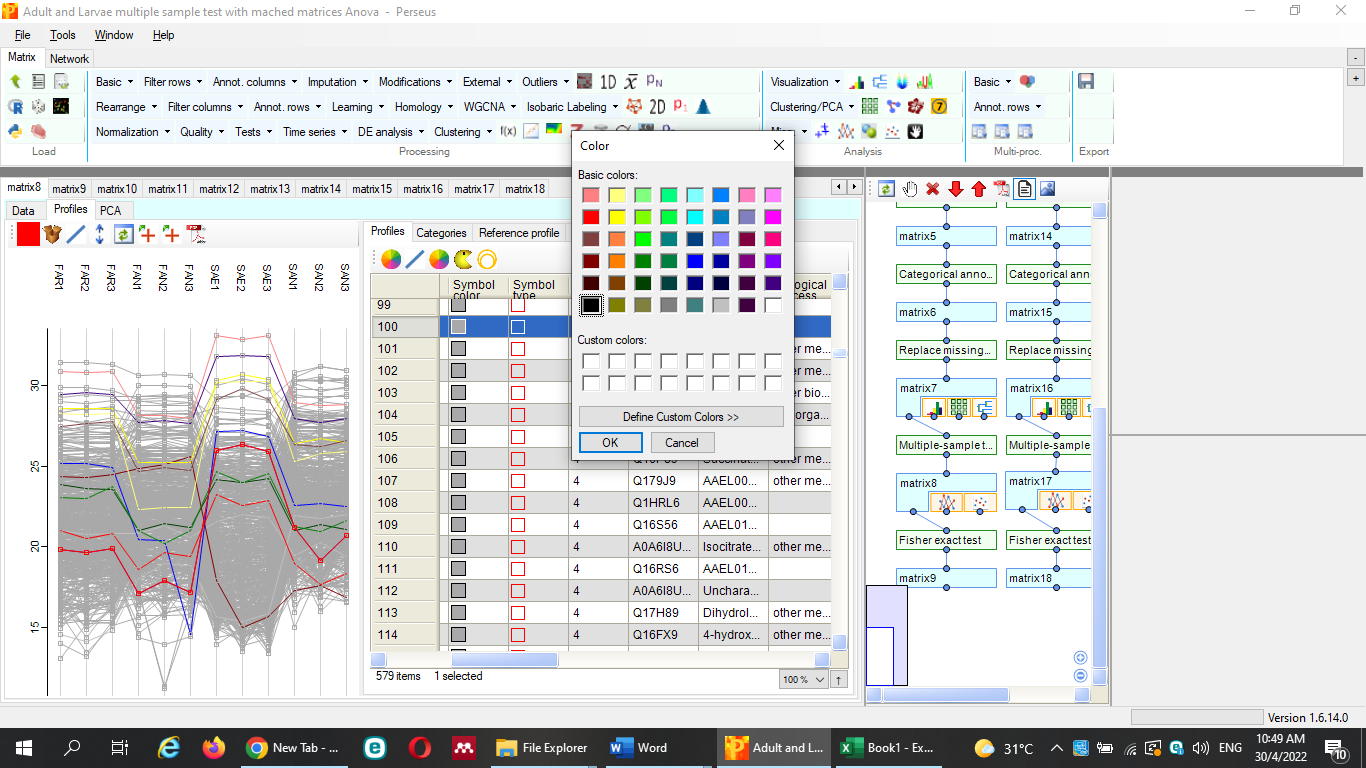 | 40S ribosomal protein S3 (Q4F6X0) |  | 40S ribosomal protein SA (Q16ZR8) |
|  | 60S ribosomal protein L8 (Q1HR32) |  | 60S ribosomal protein L12 (Q1HRI6) |
|  | 40S ribosomal protein S18 (Q1HRL8) |  | 60S ribosomal protein L9 (Q1HR72) |
|  | 40S ribosomal protein S15 (Q16PF7) |  | 60S ribosomal protein L4 (Q1HQJ0) |
|  | 60S ribosomal protein L35a (Q17N60) |  | 60S ribosomal protein L30 (Q1HR35) |
|  | 60S ribosomal protein L28 (Q1HRJ1) |  | 60S ribosomal protein L22 (Q1HRP2) |
|  | 60S ribosomal protein L10 (Q1HRT6) |  | 40S ribosomal protein S19 (Q1HRR8) |
|  | 60S ribosomal protein L14 (Q1HRP1) |  | 60S ribosomal protein L27 (Q16FB1) |
|  | Acidic ribosomal protein P1 (Q17BC2) |  | 60S ribosomal protein L29 (Q1HRL5) |
|  | 60S ribosomal protein L6 (Q16ZH3) |  | 60S ribosomal protein L21 (Q1HRN4) |
|  | 60S ribosomal protein L37 (Q1HR76) |  | 40S ribosomal protein S11 (Q17IE1) |
|  | Ribosomal protein (Q16JT5) |  |  |

**S Fig K. Heat shock proteins identified by ANOVA in adult *Ae*. *aegypti* permethrin resistant strain (q-value <0.05).** Notes: FAR: Field strain adult *Ae. aegypti* permethrin resistant. FAN: Field strain adult *Ae. aegypti* not exposed to permethrin. SAE: Laboratory strain adult *Ae. aegypti* exposed to permethrin. SAN: Laboratory strain adult *Ae. aegypti* not exposed to permethrin.

**Notes:**

|  | Heat shock cognate 70 (Q1HQZ5) |  |  |
| --- | --- | --- | --- |

**S Fig L. Heat shock proteins identified by ANOVA in adult *Ae*. *aegypti* temephos resistant strain (q-value <0.05).** Notes: FLR: Field strain *Ae. aegypti* larvae temephos resistant. FLN: Field strain *Ae. aegypti* larvae not exposed to temephos. SLE: Laboratory strain *Ae. aegypti* larvae exposed to temephos. SLN: Laboratory strain *Ae. aegypti* larvae not exposed to temephos.

**Notes:**

|  | Heat shock cognate 70 (Q1HR69) |  | Heat shock cognate 70 (Q1HQZ5) |
| --- | --- | --- | --- |
|  | Heat shock protein 83 (Q16KZ2) |  |  |
